# Extracting Clinical Guideline Information Using Two Large Language Models: Evaluation Study

**DOI:** 10.2196/73486

**Published:** 2025-09-05

**Authors:** Hsing-Yu Hsu, Lu-Wen Chen, Wan-Tseng Hsu, Yow-Wen Hsieh, Shih-Sheng Chang

**Affiliations:** 1 Graduate Institute of Clinical Pharmacy, College of Medicine, National Taiwan University, Taipei, Taiwan; 2 Department of Pharmacy, China Medical University Hospital, Taichung, Taiwan; 3 Artificial Intelligence Center, China Medical University Hospital, 2, Yude Road, Taichung, 404327, Taiwan, 886 4-22052121; 4 School of Pharmacy, College of Pharmacy, China Medical University, Taichung, Taiwan; 5 Division of Cardiovascular Medicine, Department of Medicine, China Medical University Hospital, Taichung, Taiwan; 6 School of Medicine, China Medical University, Taichung, Taiwan

**Keywords:** large language models, reproducibility, reliability, Guideline Classification, Pharmacogenomics, Clinical Decision Support System

## Abstract

**Background:**

The effective implementation of personalized pharmacogenomics (PGx) requires the integration of released clinical guidelines into decision support systems to facilitate clinical applications. Large language models (LLMs) can be valuable tools for automating information extraction and updates.

**Objective:**

This study aimed to assess the effectiveness of repeated cross-comparisons and an agreement-threshold strategy in 2 advanced LLMs as supportive tools for updating information.

**Methods:**

The study evaluated the performance of 2 LLMs, GPT-4o and Gemini-1.5-Pro, in extracting PGx clinical guidelines and comparing their outputs with expert-annotated evaluations. The 2 LLMs classified 385 PGx clinical guidelines, with each recommendation tested 20 times per model. Accuracy was assessed by comparing the results with manually labeled data. Two prospectively defined strategies were used to identify inconsistent predictions. The first involved repeated cross-comparison, flagging discrepancies between the most frequent classifications from each model. The second used a consistency threshold strategy, which designated predictions appearing in less than 60% of the 40 combined outputs as unstable. Cases flagged by either strategy were subjected to manual review. This study also estimated the overall cost of model use and was conducted between October 1 and November 30, 2024.

**Results:**

GPT-4o and Gemini-1.5-Pro yielded reproducibility rates of 97.8% (7534/7700) and 98.9% (7612/7700), respectively, based on the most frequent classification for each query. Compared with expert labels, GPT-4o achieved 93.5% accuracy (Cohen κ=0.90; *P*<.001) and Gemini-1.5-Pro 92.7% accuracy (Cohen κ=0.89; *P*<.001). Both models demonstrated high overall performance, with comparable weighted average *F*_1_-scores (GPT-4o: 0.929; Gemini: 0.935). The models generated consistent predictions for 341 of 385 guideline items, reducing the need for manual review by 88.6%. Among these agreed-upon cases, only one (0.3%) diverged from expert labels. Applying a predefined agreement-threshold strategy further reduced the number of priority manual review cases to 2.9% (11/385), although the error rate slightly increased to 0.5% (2/374). The inconsistencies identified through these methods prompted the prioritization of manual review to minimize errors and enhance clinical applicability. The total combined cost of using both LLMs was only US $0.76.

**Conclusions:**

These findings suggest that using 2 LLMs can effectively streamline PGx guideline integration into clinical decision support systems while maintaining high performance and minimal cost. Although selective manual review remains necessary, this approach offers a practical and scalable solution for PGx guideline classification in clinical workflows.

## Introduction

As pharmacogenomic (PGx) testing becomes more widely available, enhancing decision-making and knowledge-sharing in clinical genetics is essential for integrating PGx data into routine clinical practice [1]. PGx knowledge is new, complex, and constantly evolving, making it inadequate to rely solely on clinicians’ expertise for clinical implementation [2]. We developed a comprehensive database of drug-gene interactions and incorporated it into clinical decision support systems (CDSS) to achieve truly personalized treatment, improve patient outcomes, and optimize treatment strategies.

However, maintaining CDSS knowledge bases is crucial to ensuring alignment with the evolving nature of medical practice and clinical guidelines [3,4]. These labor-intensive activities necessitate manual input and significantly contribute to burnout among pharmacists. Recent research has highlighted the transformative potential of generative artificial intelligence (AI), particularly in leveraging large language models (LLMs) for data analysis and text generation to make complex medical information more accessible to health care providers and patients [5-7]. However, applying PGx guidelines as structured input for AI models remains rare, and even fewer studies have explored how to summarize classification task results effectively [8].

We hypothesize that using cross-comparison methods with 2 state-of-the-art LLMs can facilitate real-time updates to CDSS knowledge bases in health care institutions. Our objective is to enhance the clinical applicability and reliability of medical information while improving the dissemination of PGx knowledge.

## Methods

### Study Design

This methodological study followed the enhanced Transparent Reporting of a Multivariable Prediction Model for Individual Prognosis or Diagnosis (TRIPOD)-LLM reporting guidelines [9].

### Ethical Considerations

This study did not involve human participants, identifiable personal data, or biological materials. Therefore, the Institutional Review Board of China Medical University Hospital determined that the study did not require ethical review or informed consent. All data used in the study were publicly available or derived from deidentified, nonhuman sources. No interventions, clinical recruitment, or patient-level interactions were conducted.

### Model Selection and Data Sources

GPT-4o and Gemini-1.5-Pro were selected for their stable and publicly available cloud-based application programming interfaces (APIs), which support integration into clinical workflows and system deployment. In addition to their practical usability, both models have demonstrated strong performance in medical language understanding and instruction adherence in prior biomedical natural language processing studies [10,11]. Accordingly, we used these 2 LLMs to perform the classification tasks, analyzing drug-gene interaction recommendations derived from 385 clinical guideline annotations in PharmGKB as of October 5, 2024. Details of the retrospective datasets from PharmGKB are provided in Table S1 in Multimedia Appendix 1. All experiments and analyses involving the tested LLMs were conducted in English between October 1 and November 30, 2024.

### Classification Process and Prompt Design

Researchers supplied LLMs (GPT-4o and Gemini-1.5-Pro) with pertinent information on drug-gene interactions. Using this data, LLMs were instructed to generate recommendations based on a predefined prompt. To enable rapid adaptation to complex tasks and efficient data processing, we used zero-shot prompting techniques. The prompt used was: “ Your task is to summarize the following content: Provide a treatment recommendation categorized into one of the following options: “No action needed,” “Consider dosage modification,” “Change medication,” or “Monitor adverse effects.” If none of these categories fit, you may respond with “Other.” If both dosage modification and adverse effect monitoring were recommended, priority was given to classifying the recommendation under dosage modification. If both dosage modification and changing medication were recommended, priority was given to classifying the recommendation under changing medication. Please directly give “No action needed,” “Consider dosage modification,” “Change medication,” or “Monitor adverse effects.” The classification tasks of these 4 options must comply with the CDSS system update requirements, and there is no need to explain the reasons.” For each input query, separate responses from GPT-4o and Gemini-1.5-Pro were recorded and are presented in Tables S2 and S3 in Multimedia Appendix 1.

### Evaluation of Reproducibility, Accuracy, and Human Validation

GPT-4o and Gemini-1.5-Pro were used to evaluate 385 drug-gene interaction recommendations. Each recommendation underwent 20 repeated tests, generating 7700 responses per model (15,400 in total). Reproducibility was assessed by calculating the proportion of consistent responses across the 20 repetitions for each recommendation, using the most frequent response per model as the reference. Accuracy was evaluated by comparing model outputs with human-labeled data (Table S4 in Multimedia Appendix 1).

To further enhance accuracy and mitigate misclassification and hallucination issues, we implemented 2 strategies: (1) repeated cross-comparisons and (2) agreement-threshold strategy.

#### Repeated Cross-Comparisons

Classifications were first assessed within each model. Recommendations with inconsistent outputs between models were flagged for manual review.

#### Agreement-Threshold Strategy

For each recommendation, we aggregated 40 outputs from both models. If a classification appeared in ≥60% of responses (at least 24 out of 40 occurrences), it was considered stable and accepted as the final result. Those below the threshold triggered manual review.

### Expert Validation

To facilitate expert validation, 5 pharmacists from hospitals of varying sizes participated in reviewing and classifying the guideline content. To establish a reference standard for comparison with LLM-generated outputs, each pharmacist independently reviewed the PGx guidelines and categorized the therapeutic recommendations using the same classification scheme as the LLMs. Recommendations were assigned to the “Other” category if none of the predefined options were applicable. A final consensus meeting was conducted to resolve discrepancies and determine the definitive reference labels.

### Technical Implementation and CDSS Integration

PGx guideline information was initially curated by clinical pharmacists and stored in JSON format. A Python-based pipeline was developed to parse drug names, gene symbols, and recommendation summaries, which were then standardized into 385 unique drug-gene pairs. Each pair was processed 20 times by both GPT-4o and Gemini-1.5-Pro using fixed prompts via publicly available cloud-based APIs. The outputs were recorded in a structured tabular format, where each column represented one model repetition, enabling reproducible and scalable classification. To integrate the results into clinical workflows, model outputs were reviewed for consistency. Cases with discrepancies across repetitions or between models were flagged for manual review. Verified results were then automatically formatted for incorporation into the existing CDSS database.

### Economic Evaluation

We conducted a preliminary evaluation of cost based on publicly available API pricing data. As of April 17, 2025, according to the pricing overview published on the Azure OpenAI Service website, the cost for GPT-4o is US $2.50 per million tokens for input and US $10.00 per million tokens for output. For Gemini 1.5 Pro, based on the pricing listed on Google Cloud’s Vertex AI Generative AI service, the input cost is US $0.3125 per million tokens, and the output cost is US $1.25 per million tokens.

### Statistical Analysis

Descriptive statistics were used to report the reproducibility results of the classification tool. We also evaluated the accuracy of GPT-4o and Gemini-1.5-Pro in comparison with human-labeled data. For each LLM, classifications that agreed with human-labeled data were coded as 1, while disagreements were coded as 0. Cohen κ was used to assess the level of agreement. The κ statistic measures the level of agreement between 2 raters, accounting for agreement expected by chance [12]. Cohen κ was computed using the irr package in R (version 4.4.0; R Foundation for Statistical Computing). A *P* value of less than .05 was considered statistically significant. We calculated category-specific metrics to comprehensively evaluate the performance of the 2 LLM models. These metrics included precision (true positive number / total positive number), recall (sensitivity, true positive number / total actual number), and *F*_1_-score (2×[precision / [precision+ recall]). All statistical analyses were conducted using R.

## Results

**Figure 1.**
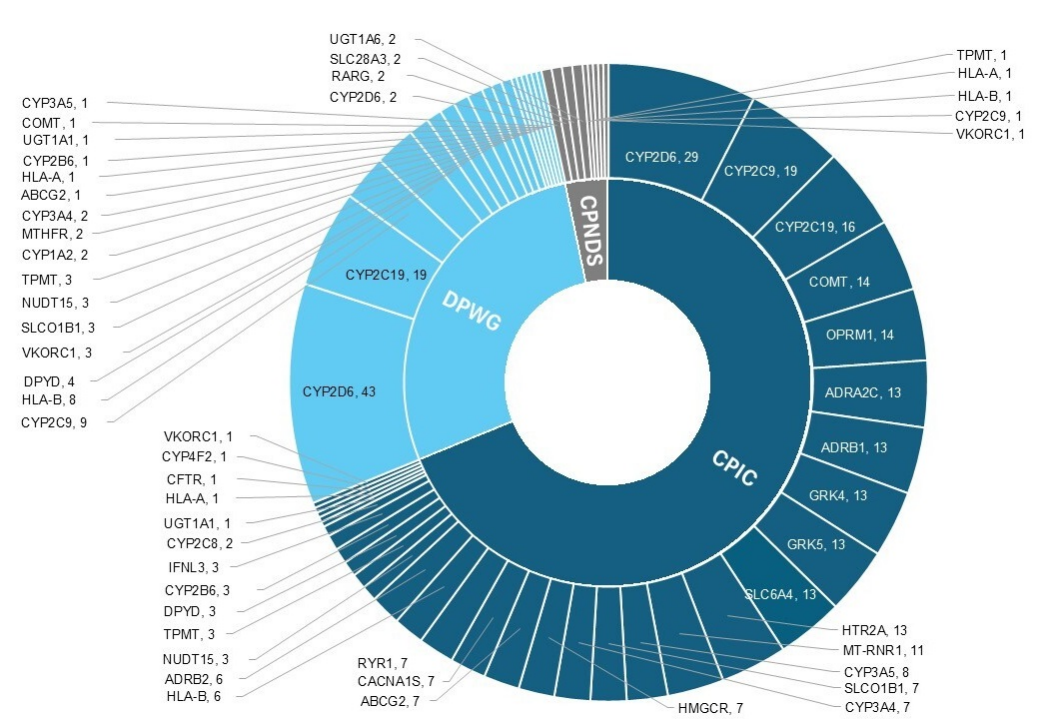
Distribution of pharmacogenomic guidelines by source and gene target. This sunburst chart illustrates the distribution of 385 PGx guideline recommendations across 3 major sources: CPIC, DPWG, and CPNDS. The inner ring represents the guideline sources, while the outer ring details the corresponding genes and the number of guidelines associated with each gene. CPIC: Clinical Pharmacogenetics Implementation Consortium; CPNDS: Canadian Pharmacogenomics Network for Drug Safety; DPWG: Dutch Pharmacogenetics Working Group.

Among 385 PGx guideline recommendations, the majority were provided by the Clinical Pharmacogenetics Implementation Consortium (265/385, 69%). Most PGx interactions involved *CYP2D6*, *CYP2C9*, and *CYP2C19*. The largest proportion of guidelines pertained to drugs targeting the nervous system (147/385, 38.2%) and the cardiovascular system (115/385, 29.9%). These were followed by antineoplastic and immunomodulating agents (37/385, 9.6%). Drugs targeting the musculoskeletal system, anti-infectives for systemic use, and the alimentary tract and metabolism each accounted for 20 (5.2%). Medications related to the blood and blood-forming organs represented 4.4% (n=17), while those targeting the respiratory system were the least represented, comprising 2.3% (n=9) (Figure 1; Table S1 in Multimedia Appendix 1).

**Figure 2.**
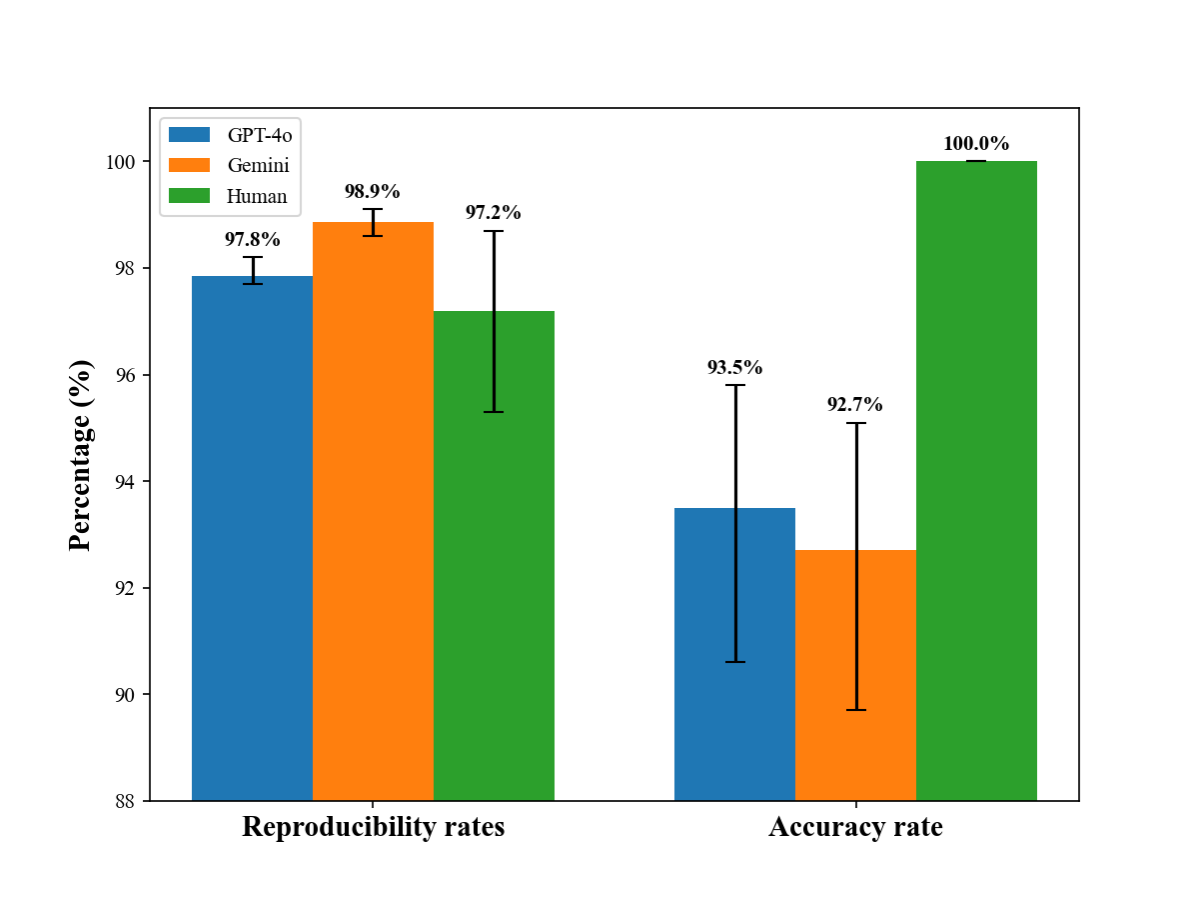
Reproducibility and accuracy rates of pharmacogenomic guideline classifications using GPT-4o, Gemini, and human review. This figure compares the reproducibility and accuracy rates of PGx guideline classifications using GPT-4o, Gemini, and human review. GPT-4o and Gemini showed high reproducibility rates (97.8% and 98.9%, respectively), and slightly lower accuracy rates (93.5% and 92.7%) when compared with human consensus labels. Human annotations, used as the gold standard for evaluation, yielded 100% accuracy. However, initial interrater reproducibility among human reviewers before consensus was 97.2% (see results section).

Among 7700 responses, GPT-4o produced 7534 consistent responses (97.8%; 95% CI 0.977‐0.982), while Gemini-1.5-Pro generated 7612 consistent responses (98.9%; 95% CI 0.986‐0.991) (Tables S2 and S3 in Multimedia Appendix 1). Human labeling consistency was 97.2% (95% CI 0.953‐0.987) (Table S4 in Multimedia Appendix 1). A total of 385 drug-gene interaction recommendations were compared with final classifications made by human experts. GPT-4o achieved agreement in 360 recommendations (accuracy: 93.5%; 95% CI 0.906‐0.958; Cohen κ=0.90; *P*<.001), while Gemini-1.5-Pro agreed on 357 recommendations (accuracy: 92.7%; 95% CI 0.897‐0.951; Cohen κ=0.89; *P*<.001) (Figure 2). The *F*_1_-scores of GPT-4o and Gemini ranged from 0.878 to 0.988 across different classes. The weighted average *F*_1_-scores were also very similar between the 2 models (GPT-4o: 0.929; Gemini: 0.935) (Table S5 in Multimedia Appendix 1).

Table 1 shows that the 2 models generated consistent predictions for 341 guideline classifications, while 44 classifications exhibited discrepancies. An analysis of the 44 guideline classifications with discrepancies revealed several recurring patterns. Specifically, 19 guidelines indicated that therapy should vary according to the patient’s metabolic type. Nine guidelines involved narrow therapeutic index drugs, for which dose adjustment should be carefully considered. Another 9 guidelines recommended monitoring for adverse events, even when the therapy was not explicitly advised. Five guidelines lacked sufficient clinical evidence to support a clear recommendation, and 2 provided dual options, advising avoidance of the drug or, if necessary, adjusting the dosage accordingly (Table S6 in Multimedia Appendix 1).

**Table 1.** Predictive performance and classification error/hallucination rates for repeated cross-comparisons and agreement-threshold strategy.

Variables	Repeated cross-comparisons (n=385), n (%)	Agreement-threshold strategy (n=385), n (%)
Predictive performance		
Classifiable guidelines	341 (88.6)	374 (97.1)
Unclassifiable guidelines (manual review rate)^a^	44 (11.4)	11 (2.9)
Classification error/hallucination rate		
Correctly classified	340 (99.7)	372 (99.5)
Incorrectly classified (hallucinations)	1 (0.3)	2 (0.5)

a Manual review rate (%) = (number of unclassifiable cases / total number of PGx guidelines)×100.

Applying a cross-comparison approach between the models reduced the proportion of guideline classifications requiring manual review to 11.4% (44/385). Furthermore, applying the predefined 60% agreement threshold (ie, at least 24 out of 40 classifications aligning with a single guideline question) further reduced the number of cases requiring prioritized manual review to 2.9% (11/385). However, this approach slightly increased the error rate, from 0.3% (1/341) to 0.5% (2/374). Table 2 lists the guideline questions where hallucinations persisted despite both AI approaches. For the 385 PGx clinical guideline entries, the total number of tokens processed in GPT-4o was 84,098 for input and 21,574 for output. In comparison, Gemini 1.5-Pro processed 446,284 input characters and 154,880 output characters. The combined total cost for running both LLMs was US $0.76 (Table 3).

**Table 2.** Pharmacogenomic interaction guidelines associated with error rates or hallucination generation. This table displays pharmacogenomic interaction guidelines linked to error rates or hallucination generation during classification. It details the specific guidelines, artificial intelligence (AI) tool thresholds used, large language model (LLM) answers, manual review results, and the reasons for discrepancies with human labels. Discrepancies were mainly due to conflicting classifications for different genotypes or model-generated information beyond the scope of the guidelines.

Pharmacogenomic interaction guidelines	AI tool threshold used	LLM answers	Manual review result	Guideline content	Reasons for discrepancies with human labels (hallucinations)
No.254 trimipramine - *CYP2D6*	Repeated cross-comparisons	Consider dosage modification	Change medication	CYP2D6 UM/PMs, CYP2C19 UM/RM/PMs: Use an alternative drug. CYP2D6 or CYP2C19 PMs (if used): Reduce dose by 50%. CYP2D6 IMs: Reduce dose by 25%.	Guideline prompts conflicting classifications for different genotypes
No.211 imipramine - *CYP2D6*	Agreement-threshold strategy	Consider dosage modification	Change medication	CYP2D6 UM/PMs, CYP2C19 UM/RM/PMs: Use an alternative drug. CYP2D6 or CYP2C19 PMs (if used): Reduce dose by 50%. CYP2D6 IMs: Reduce dose by 25%.	Guideline prompts conflicting classifications for different genotypes
No.278 daunorubicin - *SLC28A3*	Agreement-threshold strategy	Consider dosage modification	Other	Perform pharmacogenomic testing for RARG rs2229774, SLC28A3 rs7853758, and UGT1A6*4 (rs17863783)	Model-generated extraneous information beyond guideline content

**Table 3.** Cost comparison of GPT-4o and Gemini 1.5-Pro based on token and character use.

Cost type^a^	GPT-4o	Gemini 1.5-Pro
Input cost (US $)	0.21 (total input tokens: 84,098)	0.14 (total input characters: 446,284)
Output cost (US $)	0.22 (total output tokens: 21,574)	0.19 (total output characters: 154,880)
Total cost (US $)	0.43	0.33

a GPT-4o pricing is based on token use, whereas Gemini 1.5-Pro pricing is based on character count. Token-to-character conversions may vary depending on language structure.

## Discussion

### Principal Findings

To our knowledge, this is the first study to evaluate the accuracy and reproducibility of PGx guideline classification using 2 advanced LLMs simultaneously. Our approach was integrated into the existing CDSS workflow via automated guideline updates, supplemented by a manual review of inconsistent classifications. This reduces documentation burden and supports real-time updates. Although prior research suggests that LLM reproducibility may decline over time [13], our findings demonstrate that GPT-4o and Gemini-1.5-Pro consistently generated stable outputs across repeated classifications at the same time point, indicating high reliability. For simplified PGx recommendations, both models produced outputs comparable to expert annotations, particularly in classifying drug-gene interaction recommendations. The simultaneous use of 2 LLMs and repeated testing improved classification accuracy and reduced low-frequency hallucinations.

### Comparison to Prior Work

Research on LLM applications in medical genetics is limited. One study explored genetic education for BRCA1-related cancer syndrome, MLH1-related cancer syndrome, and HFE-related hemochromatosis [14]. Other studies have used chatbots to educate high-risk patients, provide cancer risk assessments, and promote preventive genetic testing [15,16]. Our study applies LLMs to clinical management, classifying PGx guidelines for integration into CDSS, enhancing health care education, and PGx application. Optimizing medication management requires understanding gene-drug interactions [17]. PGx-CDSS, a key clinical decision support tool, bridges knowledge gaps for health care providers and patients [18]. However, careful implementation is crucial to prevent alert fatigue and risks of liability [19].

Restricting output classifications also minimizes medical risks and enhances clinical decision support. Another potential application of this technology is enabling health care professionals to guide patients in precise pharmacotherapy and reduce adverse drug reactions. A study evaluated various publicly available LLMs for their ability to answer cancer-related questions. No clinically significant hallucinations were observed in simple queries [20]. Similar research fine-tuned Bidirectional Encoder Representations from Transformers models for classifying pharmacy publications to enhance clinical workflow applicability and reduce bias and hallucinations [21]. Our study confirms that leveraging zero-shot performance from 2 advanced LLMs provides a promising classification tool for simple tasks.

The classification system we adopted for PGx-drug interactions, “no action needed”, “consider dosage modification,” “change medication,” and “monitor adverse effects” was inspired by the structure of established drug-drug interaction databases, such as UpToDate Lexidrug, which typically provide a single recommended action for each interaction. It is worth noting that in certain clinical scenarios, multiple management strategies, such as dose adjustment and adverse effect monitoring, may both be appropriate. However, we prioritized recommendations involving active therapeutic changes (eg, dosage modification or medication substitution) over monitoring alone. This prioritization assumes that monitoring should naturally follow any therapeutic intervention, whereas recommending monitoring as the sole action may lead clinicians to overlook the need for proactive treatment adjustments. This complexity reflects real-world clinical decision-making, where nuanced judgment is often required. By clearly defining and prioritizing actionable strategies within our classification system, we aimed to enhance the clarity of both model training and output interpretation.

In this study, discrepancies in 44 guideline classifications between the 2 LLMs were used to identify cases warranting prioritized manual review. As described in Table S6 in Multimedia Appendix 1, an analysis of these inconsistencies reveals several recurring patterns. In some instances, treatment recommendations varied according to the patient’s metabolizer phenotype. In others, guidelines presented dual options, such as advising complete drug avoidance or, alternatively, recommending dose adjustment if use was deemed necessary. Certain guidelines did not offer explicit therapeutic recommendations but suggested monitoring for potential adverse effects. Dose adjustments were also frequently recommended for drugs with a narrow therapeutic index. Last, some discrepancies resulted from insufficient supporting evidence, leading to recommendations favoring alternative therapies. These patterns underscore the inherent complexity of guideline interpretation, particularly in contexts involving conditional recommendations or limited clinical data.

After verification, the manual review workload was reduced by 88.57%. A further examination of 341 consistent results from both LLMs revealed one (0.29%) discrepancy with human-labeled data, primarily due to a guideline prompting 2 different classification decisions for distinct genotypes, leading to inconsistent LLM responses. Many studies evaluating the capabilities of AI chatbots use a threshold of >60% as a benchmark for adequate knowledge and reasoning performance [22,23]. Notably, when applying an LLM agreement threshold of >60%, the manual review workload was reduced by 97.14% compared with human-reviewed guidelines. Among 374 guidelines, 2 (0.53%) showed discrepancies with human-labeled data. Interestingly, while the daunorubicin-SLC28A guideline recommends PGx testing for RARG rs2229774, SLC28A3 rs7853758, and UGT1A6*4 (rs17863783), it lacks specific treatment recommendations. Nonetheless, LLM responses suggested dose adjustments. Studies link hENT1 expression to increased Ara-C activity and altered drug metabolism, potentially affecting pharmacokinetics, though the evidence remains inconclusive [24]. Despite these inconsistencies, these errors likely do not compromise medical quality, as each classification partially aligns with guideline recommendations. Moreover, clinicians can review the original guideline recommendations if they have concerns about the classification summary. In deployment settings, guideline classifications with inconsistent outputs, either across repeated model runs or between models, would be automatically flagged and subjected to pharmacist validation before CDSS integration.

The challenge lies in the fact that even when the same prompts and models are used, slight randomness may lead to minor variations in the generated results [25]. Nevertheless, both LLMs demonstrated high accuracy and high reproducibility, suggesting that the observed discrepancies might merely reflect random fluctuations and that a majority-based approach considering the statistical distribution of outputs could be used. Simplifying text and classifying it did not compromise the quality of health information, as the LLM was explicitly directed to produce a particular output [26]. Further comparison revealed that both GPT-4o and Gemini performed well across the 3 major categories—no action needed, considered dosage modification, and changed medication—which together accounted for 97.7% of all samples. When considering class-specific metrics and class distribution, both models continued to demonstrate performance levels approaching that of expert pharmacists. In fact, these findings align with previous research, indicating that the quality of a model’s responses depends on the content of its input. A prior study showed that using an electronic health record–integrated generative AI chatbot to automatically draft responses to patient messages can streamline workflows and reduce burnout [27].

### Economic Estimation

We conducted a preliminary evaluation of cost and response time based on publicly available API pricing and average latency data. The economic estimation indicated that the total combined cost for running both LLMs was only US $0.76. This low cost is primarily attributable to the controlled volume of input and output tokens or characters. When managed appropriately, the combined use of both models demonstrates strong feasibility for real-world implementation, offering an excellent cost-performance balance. Both GPT-4o and Gemini 1.5-Pro operate through cloud-based APIs, with response times averaging between 2 and 4 seconds. The current results suggest that using repeated cross-comparisons and an agreement-threshold strategy can effectively narrow the scope of manual review. This approach may be a viable strategy and provides an important reference for the practical implementation of PGx CDSS in clinical settings.

### Limitations

This study has several limitations. First, the PGx guidelines we used were designed for personalized pharmacotherapy and may differ in structure or language from guidelines in other medical fields, potentially limiting generalizability. To mitigate this, we focused on clearly structured guideline recommendations and standardized the prompt format to ensure interpretability and consistency. Second, LLMs generate output based on probabilistic distributions, which can produce biased responses. However, repeated inputs and constrained target outputs can approximate the most likely distribution of responses. Third, determining the correct classification can be challenging. In some clinical scenarios, multiple management strategies, such as dose adjustment and adverse effect monitoring, may all be appropriate. However, we prioritized recommendations involving active therapeutic changes. Finally, we observed an inter-rater agreement of only 97.2% among human reviewers, highlighting the inherent heterogeneity in manual classification. Discrepancies were resolved through consensus discussion, ensuring a standardized reference for comparison with LLM-generated outputs. Future research should focus on how to automate further the integration of more complex and diverse clinical guideline updates into real-world clinical workflows.

### Conclusions

These results suggest that using 2 LLMs simultaneously can simplify PGx guidelines and generate classification outputs that enhance CDSS databases. However, manual review and cautious application remain necessary, as the models are not entirely infallible. Repeated cross-comparisons and an agreement-threshold strategy offer a promising approach for updating CDSS guidelines.

## Supplementary material

10.2196/73486 Multimedia Appendix 1 Online Supplements for " Extracting Clinical Guideline Information Using Two Large Language Models: An Evaluation Study Table S1 to Table S6.

## Abbreviations

AI: artificial intelligence
API: application programming interface
CDSS: clinical decision support system
LLM: large language model
PGx: pharmacogenomic
TRIPOD: Transparent Reporting of a Multivariable Prediction Model for Individual Prognosis or Diagnosis
