# Supplementary material for: Extracting Clinical Guideline Information Using Two Large Language Models: Evaluation Study
Source: J Med Internet Res. 2025 Sep 5;27:e73486. doi: 10.2196/73486 (PMC12413144; doi:10.2196/73486)
Supplement: Multimedia Appendix 1 [file jmir-v27-e73486-s001.docx]

**Online Supplements for "** **Extracting Clinical Guideline Information Using Two Large Language Models: An Evaluation Study "**

| **Title** | **Page Number** |
| --- | --- |
| **Table S1. Summary and Sources of Pharmacogenomic Guidelines** | 2 |
| **Table S2. Prediction Stability of GPT-4o Across 20 Iterations** | 62 |
| **Table S3. Prediction Stability of Gemini 1.5-Pro Across 20 Iterations** | 82 |
| **Table S4. Classification results by five pharmacists from different healthcare systems** | 102 |
| **Table S5. Class-Wise Evaluation Metrics for GPT-4o and Gemini-1.5-Pro on Pharmacogenomic Guideline Classification** | 114 |
| **Table S6. Error analysis of 44 pharmacogenomic guideline items with classification discrepancies between GPT-4o and Gemini-1.5-Pro** | 115 |

**Table S1. Summary and Sources of Pharmacogenomic Guidelines**

| No. | **Related Chemicals Name ^a^** | **Related Genes Symbol ^b^** | **Summary Markdown ^c^** | **Guideline** | **File ^d^** |
| --- | --- | --- | --- | --- | --- |
| 1 | alfentanil | COMT | There are currently no recommendations for dosing of alfentanil, buprenorphine, codeine, fentanyl, hydrocodone, hydromorphone, levomethadone, naltrexone, remifentanil, sufentanil or tramadol based on OPRM1 or COMT genotypes. | CPIC | Annotation_of_CPIC_Guideline_for_alfentanil_buprenorphine_codeine_fentanyl_hydrocodone_hydromorphone_levomethadone_morphine_naltrexone_remifent.json |
| 2 | alfentanil | OPRM1 | There are currently no recommendations for dosing of alfentanil, buprenorphine, codeine, fentanyl, hydrocodone, hydromorphone, levomethadone, naltrexone, remifentanil, sufentanil or tramadol based on OPRM1 or COMT genotypes. | CPIC | Annotation_of_CPIC_Guideline_for_alfentanil_buprenorphine_codeine_fentanyl_hydrocodone_hydromorphone_levomethadone_morphine_naltrexone_remifent.json |
| 3 | buprenorphine | COMT | There are currently no recommendations for dosing of alfentanil, buprenorphine, codeine, fentanyl, hydrocodone, hydromorphone, levomethadone, naltrexone, remifentanil, sufentanil or tramadol based on OPRM1 or COMT genotypes. | CPIC | Annotation_of_CPIC_Guideline_for_alfentanil_buprenorphine_codeine_fentanyl_hydrocodone_hydromorphone_levomethadone_morphine_naltrexone_remifent.json |
| 4 | buprenorphine | OPRM1 | There are currently no recommendations for dosing of alfentanil, buprenorphine, codeine, fentanyl, hydrocodone, hydromorphone, levomethadone, naltrexone, remifentanil, sufentanil or tramadol based on OPRM1 or COMT genotypes. | CPIC | Annotation_of_CPIC_Guideline_for_alfentanil_buprenorphine_codeine_fentanyl_hydrocodone_hydromorphone_levomethadone_morphine_naltrexone_remifent.json |
| 5 | codeine | COMT | There are currently no recommendations for dosing of alfentanil, buprenorphine, codeine, fentanyl, hydrocodone, hydromorphone, levomethadone, naltrexone, remifentanil, sufentanil or tramadol based on OPRM1 or COMT genotypes. | CPIC | Annotation_of_CPIC_Guideline_for_alfentanil_buprenorphine_codeine_fentanyl_hydrocodone_hydromorphone_levomethadone_morphine_naltrexone_remifent.json |
| 6 | codeine | OPRM1 | There are currently no recommendations for dosing of alfentanil, buprenorphine, codeine, fentanyl, hydrocodone, hydromorphone, levomethadone, naltrexone, remifentanil, sufentanil or tramadol based on OPRM1 or COMT genotypes. | CPIC | Annotation_of_CPIC_Guideline_for_alfentanil_buprenorphine_codeine_fentanyl_hydrocodone_hydromorphone_levomethadone_morphine_naltrexone_remifent.json |
| 7 | fentanyl | COMT | There are currently no recommendations for dosing of alfentanil, buprenorphine, codeine, fentanyl, hydrocodone, hydromorphone, levomethadone, naltrexone, remifentanil, sufentanil or tramadol based on OPRM1 or COMT genotypes. | CPIC | Annotation_of_CPIC_Guideline_for_alfentanil_buprenorphine_codeine_fentanyl_hydrocodone_hydromorphone_levomethadone_morphine_naltrexone_remifent.json |
| 8 | fentanyl | OPRM1 | There are currently no recommendations for dosing of alfentanil, buprenorphine, codeine, fentanyl, hydrocodone, hydromorphone, levomethadone, naltrexone, remifentanil, sufentanil or tramadol based on OPRM1 or COMT genotypes. | CPIC | Annotation_of_CPIC_Guideline_for_alfentanil_buprenorphine_codeine_fentanyl_hydrocodone_hydromorphone_levomethadone_morphine_naltrexone_remifent.json |
| 9 | hydrocodone | COMT | There are currently no recommendations for dosing of alfentanil, buprenorphine, codeine, fentanyl, hydrocodone, hydromorphone, levomethadone, naltrexone, remifentanil, sufentanil or tramadol based on OPRM1 or COMT genotypes. | CPIC | Annotation_of_CPIC_Guideline_for_alfentanil_buprenorphine_codeine_fentanyl_hydrocodone_hydromorphone_levomethadone_morphine_naltrexone_remifent.json |
| 10 | hydrocodone | OPRM1 | There are currently no recommendations for dosing of alfentanil, buprenorphine, codeine, fentanyl, hydrocodone, hydromorphone, levomethadone, naltrexone, remifentanil, sufentanil or tramadol based on OPRM1 or COMT genotypes. | CPIC | Annotation_of_CPIC_Guideline_for_alfentanil_buprenorphine_codeine_fentanyl_hydrocodone_hydromorphone_levomethadone_morphine_naltrexone_remifent.json |
| 11 | hydromorphone | COMT | There are currently no recommendations for dosing of alfentanil, buprenorphine, codeine, fentanyl, hydrocodone, hydromorphone, levomethadone, naltrexone, remifentanil, sufentanil or tramadol based on OPRM1 or COMT genotypes. | CPIC | Annotation_of_CPIC_Guideline_for_alfentanil_buprenorphine_codeine_fentanyl_hydrocodone_hydromorphone_levomethadone_morphine_naltrexone_remifent.json |
| 12 | hydromorphone | OPRM1 | There are currently no recommendations for dosing of alfentanil, buprenorphine, codeine, fentanyl, hydrocodone, hydromorphone, levomethadone, naltrexone, remifentanil, sufentanil or tramadol based on OPRM1 or COMT genotypes. | CPIC | Annotation_of_CPIC_Guideline_for_alfentanil_buprenorphine_codeine_fentanyl_hydrocodone_hydromorphone_levomethadone_morphine_naltrexone_remifent.json |
| 13 | levomethadone | COMT | There are currently no recommendations for dosing of alfentanil, buprenorphine, codeine, fentanyl, hydrocodone, hydromorphone, levomethadone, naltrexone, remifentanil, sufentanil or tramadol based on OPRM1 or COMT genotypes. | CPIC | Annotation_of_CPIC_Guideline_for_alfentanil_buprenorphine_codeine_fentanyl_hydrocodone_hydromorphone_levomethadone_morphine_naltrexone_remifent.json |
| 14 | levomethadone | OPRM1 | There are currently no recommendations for dosing of alfentanil, buprenorphine, codeine, fentanyl, hydrocodone, hydromorphone, levomethadone, naltrexone, remifentanil, sufentanil or tramadol based on OPRM1 or COMT genotypes. | CPIC | Annotation_of_CPIC_Guideline_for_alfentanil_buprenorphine_codeine_fentanyl_hydrocodone_hydromorphone_levomethadone_morphine_naltrexone_remifent.json |
| 15 | morphine | COMT | There are currently no recommendations for dosing of alfentanil, buprenorphine, codeine, fentanyl, hydrocodone, hydromorphone, levomethadone, naltrexone, remifentanil, sufentanil or tramadol based on OPRM1 or COMT genotypes. | CPIC | Annotation_of_CPIC_Guideline_for_alfentanil_buprenorphine_codeine_fentanyl_hydrocodone_hydromorphone_levomethadone_morphine_naltrexone_remifent.json |
| 16 | morphine | OPRM1 | There are currently no recommendations for dosing of alfentanil, buprenorphine, codeine, fentanyl, hydrocodone, hydromorphone, levomethadone, naltrexone, remifentanil, sufentanil or tramadol based on OPRM1 or COMT genotypes. | CPIC | Annotation_of_CPIC_Guideline_for_alfentanil_buprenorphine_codeine_fentanyl_hydrocodone_hydromorphone_levomethadone_morphine_naltrexone_remifent.json |
| 17 | naltrexone | COMT | There are currently no recommendations for dosing of alfentanil, buprenorphine, codeine, fentanyl, hydrocodone, hydromorphone, levomethadone, naltrexone, remifentanil, sufentanil or tramadol based on OPRM1 or COMT genotypes. | CPIC | Annotation_of_CPIC_Guideline_for_alfentanil_buprenorphine_codeine_fentanyl_hydrocodone_hydromorphone_levomethadone_morphine_naltrexone_remifent.json |
| 18 | naltrexone | OPRM1 | There are currently no recommendations for dosing of alfentanil, buprenorphine, codeine, fentanyl, hydrocodone, hydromorphone, levomethadone, naltrexone, remifentanil, sufentanil or tramadol based on OPRM1 or COMT genotypes. | CPIC | Annotation_of_CPIC_Guideline_for_alfentanil_buprenorphine_codeine_fentanyl_hydrocodone_hydromorphone_levomethadone_morphine_naltrexone_remifent.json |
| 19 | remifentanil | COMT | There are currently no recommendations for dosing of alfentanil, buprenorphine, codeine, fentanyl, hydrocodone, hydromorphone, levomethadone, naltrexone, remifentanil, sufentanil or tramadol based on OPRM1 or COMT genotypes. | CPIC | Annotation_of_CPIC_Guideline_for_alfentanil_buprenorphine_codeine_fentanyl_hydrocodone_hydromorphone_levomethadone_morphine_naltrexone_remifent.json |
| 20 | remifentanil | OPRM1 | There are currently no recommendations for dosing of alfentanil, buprenorphine, codeine, fentanyl, hydrocodone, hydromorphone, levomethadone, naltrexone, remifentanil, sufentanil or tramadol based on OPRM1 or COMT genotypes. | CPIC | Annotation_of_CPIC_Guideline_for_alfentanil_buprenorphine_codeine_fentanyl_hydrocodone_hydromorphone_levomethadone_morphine_naltrexone_remifent.json |
| 21 | sufentanil | COMT | There are currently no recommendations for dosing of alfentanil, buprenorphine, codeine, fentanyl, hydrocodone, hydromorphone, levomethadone, naltrexone, remifentanil, sufentanil or tramadol based on OPRM1 or COMT genotypes. | CPIC | Annotation_of_CPIC_Guideline_for_alfentanil_buprenorphine_codeine_fentanyl_hydrocodone_hydromorphone_levomethadone_morphine_naltrexone_remifent.json |
| 22 | sufentanil | OPRM1 | There are currently no recommendations for dosing of alfentanil, buprenorphine, codeine, fentanyl, hydrocodone, hydromorphone, levomethadone, naltrexone, remifentanil, sufentanil or tramadol based on OPRM1 or COMT genotypes. | CPIC | Annotation_of_CPIC_Guideline_for_alfentanil_buprenorphine_codeine_fentanyl_hydrocodone_hydromorphone_levomethadone_morphine_naltrexone_remifent.json |
| 23 | tramadol | COMT | There are currently no recommendations for dosing of alfentanil, buprenorphine, codeine, fentanyl, hydrocodone, hydromorphone, levomethadone, naltrexone, remifentanil, sufentanil or tramadol based on OPRM1 or COMT genotypes. | CPIC | Annotation_of_CPIC_Guideline_for_alfentanil_buprenorphine_codeine_fentanyl_hydrocodone_hydromorphone_levomethadone_morphine_naltrexone_remifent.json |
| 24 | tramadol | OPRM1 | There are currently no recommendations for dosing of alfentanil, buprenorphine, codeine, fentanyl, hydrocodone, hydromorphone, levomethadone, naltrexone, remifentanil, sufentanil or tramadol based on OPRM1 or COMT genotypes. | CPIC | Annotation_of_CPIC_Guideline_for_alfentanil_buprenorphine_codeine_fentanyl_hydrocodone_hydromorphone_levomethadone_morphine_naltrexone_remifent.json |
| 25 | allopurinol | HLA-B | Allopurinol is contraindicated in individuals with the HLA-B*58:01 variant allele ("HLA-B*58:01-positive") due to significantly increased risk of allopurinol-induced SCAR. | CPIC | Annotation_of_CPIC_Guideline_for_allopurinol_and_HLA_B.json |
| 26 | amitriptyline | CYP2C19 | The CPIC Dosing Guideline update for amitriptyline recommends an alternative drug for CYP2D6 ultrarapid or poor metabolizers and CYP2C19 ultrarapid, rapid or poor metabolizers. If amitriptyline is warranted, consider a 50% dose reduction in CYP2D6 or CYP2C19 poor metabolizers. For CYP2D6 intermediate metabolizers, a 25% dose reduction should be considered. | CPIC | Annotation_of_CPIC_Guideline_for_amitriptyline_and_CYP2C19_CYP2D6.json |
| 27 | amitriptyline | CYP2D6 | The CPIC Dosing Guideline update for amitriptyline recommends an alternative drug for CYP2D6 ultrarapid or poor metabolizers and CYP2C19 ultrarapid, rapid or poor metabolizers. If amitriptyline is warranted, consider a 50% dose reduction in CYP2D6 or CYP2C19 poor metabolizers. For CYP2D6 intermediate metabolizers, a 25% dose reduction should be considered. | CPIC | Annotation_of_CPIC_Guideline_for_amitriptyline_and_CYP2C19_CYP2D6.json |
| 28 | atazanavir | UGT1A1 | The CPIC dosing guideline recommends considering advising individuals who carry two decreased function UGT1A1 alleles about a substantial likelihood of developing jaundice, which may cause non-adherence. The dosing guideline recommends that alternative agents be considered if the risk of non-adherence due to jaundice is high. The risk of discontinuation is low and very low for individuals carrying one, or no decreased function UGT1A1 alleles, respectively. | CPIC | Annotation_of_CPIC_Guideline_for_atazanavir_and_UGT1A1.json |
| 29 | azathioprine | NUDT15 | Consider an alternate agent or extreme dose reduction of azathioprine for patients who are TPMT or NUDT15 poor metabolizers. Start at 30-80% of target dose for patients who are intermediate metabolizers of either TPMT or NUDT15. Start at 20-50% of target dose for patients who are intermediate metabolizers of both TPMT and NUDT15. | CPIC | Annotation_of_CPIC_Guideline_for_azathioprine_and_NUDT15_TPMT.json |
| 30 | azathioprine | TPMT | Consider an alternate agent or extreme dose reduction of azathioprine for patients who are TPMT or NUDT15 poor metabolizers. Start at 30-80% of target dose for patients who are intermediate metabolizers of either TPMT or NUDT15. Start at 20-50% of target dose for patients who are intermediate metabolizers of both TPMT and NUDT15. | CPIC | Annotation_of_CPIC_Guideline_for_azathioprine_and_NUDT15_TPMT.json |
| 31 | capecitabine | DPYD | The CPIC Dosing Guideline for 5-fluorouracil and capecitabine recommends an alternative drug for patients who are DPYD poor metabolizers with an activity score of 0. In those who are poor metabolizers with an activity score of 0.5, an alternative drug is also recommended, but if this is not considered a suitable therapeutic option, 5-fluorouracil or capecitabine should be administered at a strongly reduced dose with early therapeutic drug monitoring. Patients who are intermediate metabolizers with an activity score of 1 or 1.5 should receive a dose reduction of 50%. Patients with the c.[2846A>T];[2846A>T] genotype may require a >50% dose reduction. | CPIC | Annotation_of_CPIC_Guideline_for_capecitabine_and_DPYD.json |
| 32 | amikacin | MT-RNR1 | Administration of aminoglycoside antibiotics should be avoided in patients carrying certain MT-RNR1 variants due to the increased risk of developing aminoglycoside-induced hearing loss (AIHL). | CPIC | Annotation_of_CPIC_Guideline_for_amikacin_dibekacin_gentamicin_kanamycin_neomycin_netilmicin_paromomycin_plazomicin_ribostamycin_streptomycin_t.json |
| 33 | dibekacin | MT-RNR1 | Administration of aminoglycoside antibiotics should be avoided in patients carrying certain MT-RNR1 variants due to the increased risk of developing aminoglycoside-induced hearing loss (AIHL). | CPIC | Annotation_of_CPIC_Guideline_for_amikacin_dibekacin_gentamicin_kanamycin_neomycin_netilmicin_paromomycin_plazomicin_ribostamycin_streptomycin_t.json |
| 34 | gentamicin | MT-RNR1 | Administration of aminoglycoside antibiotics should be avoided in patients carrying certain MT-RNR1 variants due to the increased risk of developing aminoglycoside-induced hearing loss (AIHL). | CPIC | Annotation_of_CPIC_Guideline_for_amikacin_dibekacin_gentamicin_kanamycin_neomycin_netilmicin_paromomycin_plazomicin_ribostamycin_streptomycin_t.json |
| 35 | kanamycin | MT-RNR1 | Administration of aminoglycoside antibiotics should be avoided in patients carrying certain MT-RNR1 variants due to the increased risk of developing aminoglycoside-induced hearing loss (AIHL). | CPIC | Annotation_of_CPIC_Guideline_for_amikacin_dibekacin_gentamicin_kanamycin_neomycin_netilmicin_paromomycin_plazomicin_ribostamycin_streptomycin_t.json |
| 36 | neomycin | MT-RNR1 | Administration of aminoglycoside antibiotics should be avoided in patients carrying certain MT-RNR1 variants due to the increased risk of developing aminoglycoside-induced hearing loss (AIHL). | CPIC | Annotation_of_CPIC_Guideline_for_amikacin_dibekacin_gentamicin_kanamycin_neomycin_netilmicin_paromomycin_plazomicin_ribostamycin_streptomycin_t.json |
| 37 | netilmicin | MT-RNR1 | Administration of aminoglycoside antibiotics should be avoided in patients carrying certain MT-RNR1 variants due to the increased risk of developing aminoglycoside-induced hearing loss (AIHL). | CPIC | Annotation_of_CPIC_Guideline_for_amikacin_dibekacin_gentamicin_kanamycin_neomycin_netilmicin_paromomycin_plazomicin_ribostamycin_streptomycin_t.json |
| 38 | paromomycin | MT-RNR1 | Administration of aminoglycoside antibiotics should be avoided in patients carrying certain MT-RNR1 variants due to the increased risk of developing aminoglycoside-induced hearing loss (AIHL). | CPIC | Annotation_of_CPIC_Guideline_for_amikacin_dibekacin_gentamicin_kanamycin_neomycin_netilmicin_paromomycin_plazomicin_ribostamycin_streptomycin_t.json |
| 39 | plazomicin | MT-RNR1 | Administration of aminoglycoside antibiotics should be avoided in patients carrying certain MT-RNR1 variants due to the increased risk of developing aminoglycoside-induced hearing loss (AIHL). | CPIC | Annotation_of_CPIC_Guideline_for_amikacin_dibekacin_gentamicin_kanamycin_neomycin_netilmicin_paromomycin_plazomicin_ribostamycin_streptomycin_t.json |
| 40 | ribostamycin | MT-RNR1 | Administration of aminoglycoside antibiotics should be avoided in patients carrying certain MT-RNR1 variants due to the increased risk of developing aminoglycoside-induced hearing loss (AIHL). | CPIC | Annotation_of_CPIC_Guideline_for_amikacin_dibekacin_gentamicin_kanamycin_neomycin_netilmicin_paromomycin_plazomicin_ribostamycin_streptomycin_t.json |
| 41 | streptomycin | MT-RNR1 | Administration of aminoglycoside antibiotics should be avoided in patients carrying certain MT-RNR1 variants due to the increased risk of developing aminoglycoside-induced hearing loss (AIHL). | CPIC | Annotation_of_CPIC_Guideline_for_amikacin_dibekacin_gentamicin_kanamycin_neomycin_netilmicin_paromomycin_plazomicin_ribostamycin_streptomycin_t.json |
| 42 | tobramycin | MT-RNR1 | Administration of aminoglycoside antibiotics should be avoided in patients carrying certain MT-RNR1 variants due to the increased risk of developing aminoglycoside-induced hearing loss (AIHL). | CPIC | Annotation_of_CPIC_Guideline_for_amikacin_dibekacin_gentamicin_kanamycin_neomycin_netilmicin_paromomycin_plazomicin_ribostamycin_streptomycin_t.json |
| 43 | atomoxetine | CYP2D6 | The CPIC Dosing Guideline for atomoxetine provides therapeutic recommendations for CYP2D6 ultrarapid, normal, intermediate, and poor metabolizer, which includes guidance for plasma drug concentration testing, as a means to estimate atomoxetine exposure, if no clinical response and in the absence of adverse events after 2 weeks of therapy. | CPIC | Annotation_of_CPIC_Guideline_for_atomoxetine_and_CYP2D6.json |
| 44 | atorvastatin | SLCO1B1 | Prescribe ≤20mg for patients with SLCO1B1 poor function phenotype and ≤40mg for patients with SLCO1B1 decreased or possible decreased phenotype as a starting dose. Adjust doses of atorvastatin based on disease-specific guidelines. Prescriber should be aware of possible increased risk for myopathy especially for 40mg dose. | CPIC | Annotation_of_CPIC_Guideline_for_atorvastatin_and_SLCO1B1.json |
| 45 | acebutolol | ADRA2C | There are currently no recommendations for dosing of orally administered beta-blockers based on ADRB1, ADRA2C, GRK4 and/or GRK5 genotypes. | CPIC | Annotation_of_CPIC_Guideline_for_acebutolol_atenolol_betaxolol_bisoprolol_carvedilol_esmolol_labetalol_metoprolol_nadolol_nebivolol_pindolol_pr.json |
| 46 | acebutolol | ADRB1 | There are currently no recommendations for dosing of orally administered beta-blockers based on ADRB1, ADRA2C, GRK4 and/or GRK5 genotypes. | CPIC | Annotation_of_CPIC_Guideline_for_acebutolol_atenolol_betaxolol_bisoprolol_carvedilol_esmolol_labetalol_metoprolol_nadolol_nebivolol_pindolol_pr.json |
| 47 | acebutolol | GRK4 | There are currently no recommendations for dosing of orally administered beta-blockers based on ADRB1, ADRA2C, GRK4 and/or GRK5 genotypes. | CPIC | Annotation_of_CPIC_Guideline_for_acebutolol_atenolol_betaxolol_bisoprolol_carvedilol_esmolol_labetalol_metoprolol_nadolol_nebivolol_pindolol_pr.json |
| 48 | acebutolol | GRK5 | There are currently no recommendations for dosing of orally administered beta-blockers based on ADRB1, ADRA2C, GRK4 and/or GRK5 genotypes. | CPIC | Annotation_of_CPIC_Guideline_for_acebutolol_atenolol_betaxolol_bisoprolol_carvedilol_esmolol_labetalol_metoprolol_nadolol_nebivolol_pindolol_pr.json |
| 49 | atenolol | ADRA2C | There are currently no recommendations for dosing of orally administered beta-blockers based on ADRB1, ADRA2C, GRK4 and/or GRK5 genotypes. | CPIC | Annotation_of_CPIC_Guideline_for_acebutolol_atenolol_betaxolol_bisoprolol_carvedilol_esmolol_labetalol_metoprolol_nadolol_nebivolol_pindolol_pr.json |
| 50 | atenolol | ADRB1 | There are currently no recommendations for dosing of orally administered beta-blockers based on ADRB1, ADRA2C, GRK4 and/or GRK5 genotypes. | CPIC | Annotation_of_CPIC_Guideline_for_acebutolol_atenolol_betaxolol_bisoprolol_carvedilol_esmolol_labetalol_metoprolol_nadolol_nebivolol_pindolol_pr.json |
| 51 | atenolol | GRK4 | There are currently no recommendations for dosing of orally administered beta-blockers based on ADRB1, ADRA2C, GRK4 and/or GRK5 genotypes. | CPIC | Annotation_of_CPIC_Guideline_for_acebutolol_atenolol_betaxolol_bisoprolol_carvedilol_esmolol_labetalol_metoprolol_nadolol_nebivolol_pindolol_pr.json |
| 52 | atenolol | GRK5 | There are currently no recommendations for dosing of orally administered beta-blockers based on ADRB1, ADRA2C, GRK4 and/or GRK5 genotypes. | CPIC | Annotation_of_CPIC_Guideline_for_acebutolol_atenolol_betaxolol_bisoprolol_carvedilol_esmolol_labetalol_metoprolol_nadolol_nebivolol_pindolol_pr.json |
| 53 | betaxolol | ADRA2C | There are currently no recommendations for dosing of orally administered beta-blockers based on ADRB1, ADRA2C, GRK4 and/or GRK5 genotypes. | CPIC | Annotation_of_CPIC_Guideline_for_acebutolol_atenolol_betaxolol_bisoprolol_carvedilol_esmolol_labetalol_metoprolol_nadolol_nebivolol_pindolol_pr.json |
| 54 | betaxolol | ADRB1 | There are currently no recommendations for dosing of orally administered beta-blockers based on ADRB1, ADRA2C, GRK4 and/or GRK5 genotypes. | CPIC | Annotation_of_CPIC_Guideline_for_acebutolol_atenolol_betaxolol_bisoprolol_carvedilol_esmolol_labetalol_metoprolol_nadolol_nebivolol_pindolol_pr.json |
| 55 | betaxolol | GRK4 | There are currently no recommendations for dosing of orally administered beta-blockers based on ADRB1, ADRA2C, GRK4 and/or GRK5 genotypes. | CPIC | Annotation_of_CPIC_Guideline_for_acebutolol_atenolol_betaxolol_bisoprolol_carvedilol_esmolol_labetalol_metoprolol_nadolol_nebivolol_pindolol_pr.json |
| 56 | betaxolol | GRK5 | There are currently no recommendations for dosing of orally administered beta-blockers based on ADRB1, ADRA2C, GRK4 and/or GRK5 genotypes. | CPIC | Annotation_of_CPIC_Guideline_for_acebutolol_atenolol_betaxolol_bisoprolol_carvedilol_esmolol_labetalol_metoprolol_nadolol_nebivolol_pindolol_pr.json |
| 57 | bisoprolol | ADRA2C | There are currently no recommendations for dosing of orally administered beta-blockers based on ADRB1, ADRA2C, GRK4 and/or GRK5 genotypes. | CPIC | Annotation_of_CPIC_Guideline_for_acebutolol_atenolol_betaxolol_bisoprolol_carvedilol_esmolol_labetalol_metoprolol_nadolol_nebivolol_pindolol_pr.json |
| 58 | bisoprolol | ADRB1 | There are currently no recommendations for dosing of orally administered beta-blockers based on ADRB1, ADRA2C, GRK4 and/or GRK5 genotypes. | CPIC | Annotation_of_CPIC_Guideline_for_acebutolol_atenolol_betaxolol_bisoprolol_carvedilol_esmolol_labetalol_metoprolol_nadolol_nebivolol_pindolol_pr.json |
| 59 | bisoprolol | GRK4 | There are currently no recommendations for dosing of orally administered beta-blockers based on ADRB1, ADRA2C, GRK4 and/or GRK5 genotypes. | CPIC | Annotation_of_CPIC_Guideline_for_acebutolol_atenolol_betaxolol_bisoprolol_carvedilol_esmolol_labetalol_metoprolol_nadolol_nebivolol_pindolol_pr.json |
| 60 | bisoprolol | GRK5 | There are currently no recommendations for dosing of orally administered beta-blockers based on ADRB1, ADRA2C, GRK4 and/or GRK5 genotypes. | CPIC | Annotation_of_CPIC_Guideline_for_acebutolol_atenolol_betaxolol_bisoprolol_carvedilol_esmolol_labetalol_metoprolol_nadolol_nebivolol_pindolol_pr.json |
| 61 | carvedilol | ADRA2C | There are currently no recommendations for dosing of orally administered beta-blockers based on ADRB1, ADRA2C, GRK4 and/or GRK5 genotypes. | CPIC | Annotation_of_CPIC_Guideline_for_acebutolol_atenolol_betaxolol_bisoprolol_carvedilol_esmolol_labetalol_metoprolol_nadolol_nebivolol_pindolol_pr.json |
| 62 | carvedilol | ADRB1 | There are currently no recommendations for dosing of orally administered beta-blockers based on ADRB1, ADRA2C, GRK4 and/or GRK5 genotypes. | CPIC | Annotation_of_CPIC_Guideline_for_acebutolol_atenolol_betaxolol_bisoprolol_carvedilol_esmolol_labetalol_metoprolol_nadolol_nebivolol_pindolol_pr.json |
| 63 | carvedilol | GRK4 | There are currently no recommendations for dosing of orally administered beta-blockers based on ADRB1, ADRA2C, GRK4 and/or GRK5 genotypes. | CPIC | Annotation_of_CPIC_Guideline_for_acebutolol_atenolol_betaxolol_bisoprolol_carvedilol_esmolol_labetalol_metoprolol_nadolol_nebivolol_pindolol_pr.json |
| 64 | carvedilol | GRK5 | There are currently no recommendations for dosing of orally administered beta-blockers based on ADRB1, ADRA2C, GRK4 and/or GRK5 genotypes. | CPIC | Annotation_of_CPIC_Guideline_for_acebutolol_atenolol_betaxolol_bisoprolol_carvedilol_esmolol_labetalol_metoprolol_nadolol_nebivolol_pindolol_pr.json |
| 65 | esmolol | ADRA2C | There are currently no recommendations for dosing of orally administered beta-blockers based on ADRB1, ADRA2C, GRK4 and/or GRK5 genotypes. | CPIC | Annotation_of_CPIC_Guideline_for_acebutolol_atenolol_betaxolol_bisoprolol_carvedilol_esmolol_labetalol_metoprolol_nadolol_nebivolol_pindolol_pr.json |
| 66 | esmolol | ADRB1 | There are currently no recommendations for dosing of orally administered beta-blockers based on ADRB1, ADRA2C, GRK4 and/or GRK5 genotypes. | CPIC | Annotation_of_CPIC_Guideline_for_acebutolol_atenolol_betaxolol_bisoprolol_carvedilol_esmolol_labetalol_metoprolol_nadolol_nebivolol_pindolol_pr.json |
| 67 | esmolol | GRK4 | There are currently no recommendations for dosing of orally administered beta-blockers based on ADRB1, ADRA2C, GRK4 and/or GRK5 genotypes. | CPIC | Annotation_of_CPIC_Guideline_for_acebutolol_atenolol_betaxolol_bisoprolol_carvedilol_esmolol_labetalol_metoprolol_nadolol_nebivolol_pindolol_pr.json |
| 68 | esmolol | GRK5 | There are currently no recommendations for dosing of orally administered beta-blockers based on ADRB1, ADRA2C, GRK4 and/or GRK5 genotypes. | CPIC | Annotation_of_CPIC_Guideline_for_acebutolol_atenolol_betaxolol_bisoprolol_carvedilol_esmolol_labetalol_metoprolol_nadolol_nebivolol_pindolol_pr.json |
| 69 | labetalol | ADRA2C | There are currently no recommendations for dosing of orally administered beta-blockers based on ADRB1, ADRA2C, GRK4 and/or GRK5 genotypes. | CPIC | Annotation_of_CPIC_Guideline_for_acebutolol_atenolol_betaxolol_bisoprolol_carvedilol_esmolol_labetalol_metoprolol_nadolol_nebivolol_pindolol_pr.json |
| 70 | labetalol | ADRB1 | There are currently no recommendations for dosing of orally administered beta-blockers based on ADRB1, ADRA2C, GRK4 and/or GRK5 genotypes. | CPIC | Annotation_of_CPIC_Guideline_for_acebutolol_atenolol_betaxolol_bisoprolol_carvedilol_esmolol_labetalol_metoprolol_nadolol_nebivolol_pindolol_pr.json |
| 71 | labetalol | GRK4 | There are currently no recommendations for dosing of orally administered beta-blockers based on ADRB1, ADRA2C, GRK4 and/or GRK5 genotypes. | CPIC | Annotation_of_CPIC_Guideline_for_acebutolol_atenolol_betaxolol_bisoprolol_carvedilol_esmolol_labetalol_metoprolol_nadolol_nebivolol_pindolol_pr.json |
| 72 | labetalol | GRK5 | There are currently no recommendations for dosing of orally administered beta-blockers based on ADRB1, ADRA2C, GRK4 and/or GRK5 genotypes. | CPIC | Annotation_of_CPIC_Guideline_for_acebutolol_atenolol_betaxolol_bisoprolol_carvedilol_esmolol_labetalol_metoprolol_nadolol_nebivolol_pindolol_pr.json |
| 73 | metoprolol | ADRA2C | There are currently no recommendations for dosing of orally administered beta-blockers based on ADRB1, ADRA2C, GRK4 and/or GRK5 genotypes. | CPIC | Annotation_of_CPIC_Guideline_for_acebutolol_atenolol_betaxolol_bisoprolol_carvedilol_esmolol_labetalol_metoprolol_nadolol_nebivolol_pindolol_pr.json |
| 74 | metoprolol | ADRB1 | There are currently no recommendations for dosing of orally administered beta-blockers based on ADRB1, ADRA2C, GRK4 and/or GRK5 genotypes. | CPIC | Annotation_of_CPIC_Guideline_for_acebutolol_atenolol_betaxolol_bisoprolol_carvedilol_esmolol_labetalol_metoprolol_nadolol_nebivolol_pindolol_pr.json |
| 75 | metoprolol | GRK4 | There are currently no recommendations for dosing of orally administered beta-blockers based on ADRB1, ADRA2C, GRK4 and/or GRK5 genotypes. | CPIC | Annotation_of_CPIC_Guideline_for_acebutolol_atenolol_betaxolol_bisoprolol_carvedilol_esmolol_labetalol_metoprolol_nadolol_nebivolol_pindolol_pr.json |
| 76 | metoprolol | GRK5 | There are currently no recommendations for dosing of orally administered beta-blockers based on ADRB1, ADRA2C, GRK4 and/or GRK5 genotypes. | CPIC | Annotation_of_CPIC_Guideline_for_acebutolol_atenolol_betaxolol_bisoprolol_carvedilol_esmolol_labetalol_metoprolol_nadolol_nebivolol_pindolol_pr.json |
| 77 | nadolol | ADRA2C | There are currently no recommendations for dosing of orally administered beta-blockers based on ADRB1, ADRA2C, GRK4 and/or GRK5 genotypes. | CPIC | Annotation_of_CPIC_Guideline_for_acebutolol_atenolol_betaxolol_bisoprolol_carvedilol_esmolol_labetalol_metoprolol_nadolol_nebivolol_pindolol_pr.json |
| 78 | nadolol | ADRB1 | There are currently no recommendations for dosing of orally administered beta-blockers based on ADRB1, ADRA2C, GRK4 and/or GRK5 genotypes. | CPIC | Annotation_of_CPIC_Guideline_for_acebutolol_atenolol_betaxolol_bisoprolol_carvedilol_esmolol_labetalol_metoprolol_nadolol_nebivolol_pindolol_pr.json |
| 79 | nadolol | GRK4 | There are currently no recommendations for dosing of orally administered beta-blockers based on ADRB1, ADRA2C, GRK4 and/or GRK5 genotypes. | CPIC | Annotation_of_CPIC_Guideline_for_acebutolol_atenolol_betaxolol_bisoprolol_carvedilol_esmolol_labetalol_metoprolol_nadolol_nebivolol_pindolol_pr.json |
| 80 | nadolol | GRK5 | There are currently no recommendations for dosing of orally administered beta-blockers based on ADRB1, ADRA2C, GRK4 and/or GRK5 genotypes. | CPIC | Annotation_of_CPIC_Guideline_for_acebutolol_atenolol_betaxolol_bisoprolol_carvedilol_esmolol_labetalol_metoprolol_nadolol_nebivolol_pindolol_pr.json |
| 81 | nebivolol | ADRA2C | There are currently no recommendations for dosing of orally administered beta-blockers based on ADRB1, ADRA2C, GRK4 and/or GRK5 genotypes. | CPIC | Annotation_of_CPIC_Guideline_for_acebutolol_atenolol_betaxolol_bisoprolol_carvedilol_esmolol_labetalol_metoprolol_nadolol_nebivolol_pindolol_pr.json |
| 82 | nebivolol | ADRB1 | There are currently no recommendations for dosing of orally administered beta-blockers based on ADRB1, ADRA2C, GRK4 and/or GRK5 genotypes. | CPIC | Annotation_of_CPIC_Guideline_for_acebutolol_atenolol_betaxolol_bisoprolol_carvedilol_esmolol_labetalol_metoprolol_nadolol_nebivolol_pindolol_pr.json |
| 83 | nebivolol | GRK4 | There are currently no recommendations for dosing of orally administered beta-blockers based on ADRB1, ADRA2C, GRK4 and/or GRK5 genotypes. | CPIC | Annotation_of_CPIC_Guideline_for_acebutolol_atenolol_betaxolol_bisoprolol_carvedilol_esmolol_labetalol_metoprolol_nadolol_nebivolol_pindolol_pr.json |
| 84 | nebivolol | GRK5 | There are currently no recommendations for dosing of orally administered beta-blockers based on ADRB1, ADRA2C, GRK4 and/or GRK5 genotypes. | CPIC | Annotation_of_CPIC_Guideline_for_acebutolol_atenolol_betaxolol_bisoprolol_carvedilol_esmolol_labetalol_metoprolol_nadolol_nebivolol_pindolol_pr.json |
| 85 | pindolol | ADRA2C | There are currently no recommendations for dosing of orally administered beta-blockers based on ADRB1, ADRA2C, GRK4 and/or GRK5 genotypes. | CPIC | Annotation_of_CPIC_Guideline_for_acebutolol_atenolol_betaxolol_bisoprolol_carvedilol_esmolol_labetalol_metoprolol_nadolol_nebivolol_pindolol_pr.json |
| 86 | pindolol | ADRB1 | There are currently no recommendations for dosing of orally administered beta-blockers based on ADRB1, ADRA2C, GRK4 and/or GRK5 genotypes. | CPIC | Annotation_of_CPIC_Guideline_for_acebutolol_atenolol_betaxolol_bisoprolol_carvedilol_esmolol_labetalol_metoprolol_nadolol_nebivolol_pindolol_pr.json |
| 87 | pindolol | GRK4 | There are currently no recommendations for dosing of orally administered beta-blockers based on ADRB1, ADRA2C, GRK4 and/or GRK5 genotypes. | CPIC | Annotation_of_CPIC_Guideline_for_acebutolol_atenolol_betaxolol_bisoprolol_carvedilol_esmolol_labetalol_metoprolol_nadolol_nebivolol_pindolol_pr.json |
| 88 | pindolol | GRK5 | There are currently no recommendations for dosing of orally administered beta-blockers based on ADRB1, ADRA2C, GRK4 and/or GRK5 genotypes. | CPIC | Annotation_of_CPIC_Guideline_for_acebutolol_atenolol_betaxolol_bisoprolol_carvedilol_esmolol_labetalol_metoprolol_nadolol_nebivolol_pindolol_pr.json |
| 89 | propranolol | ADRA2C | There are currently no recommendations for dosing of orally administered beta-blockers based on ADRB1, ADRA2C, GRK4 and/or GRK5 genotypes. | CPIC | Annotation_of_CPIC_Guideline_for_acebutolol_atenolol_betaxolol_bisoprolol_carvedilol_esmolol_labetalol_metoprolol_nadolol_nebivolol_pindolol_pr.json |
| 90 | propranolol | ADRB1 | There are currently no recommendations for dosing of orally administered beta-blockers based on ADRB1, ADRA2C, GRK4 and/or GRK5 genotypes. | CPIC | Annotation_of_CPIC_Guideline_for_acebutolol_atenolol_betaxolol_bisoprolol_carvedilol_esmolol_labetalol_metoprolol_nadolol_nebivolol_pindolol_pr.json |
| 91 | propranolol | GRK4 | There are currently no recommendations for dosing of orally administered beta-blockers based on ADRB1, ADRA2C, GRK4 and/or GRK5 genotypes. | CPIC | Annotation_of_CPIC_Guideline_for_acebutolol_atenolol_betaxolol_bisoprolol_carvedilol_esmolol_labetalol_metoprolol_nadolol_nebivolol_pindolol_pr.json |
| 92 | propranolol | GRK5 | There are currently no recommendations for dosing of orally administered beta-blockers based on ADRB1, ADRA2C, GRK4 and/or GRK5 genotypes. | CPIC | Annotation_of_CPIC_Guideline_for_acebutolol_atenolol_betaxolol_bisoprolol_carvedilol_esmolol_labetalol_metoprolol_nadolol_nebivolol_pindolol_pr.json |
| 93 | sotalol | ADRA2C | There are currently no recommendations for dosing of orally administered beta-blockers based on ADRB1, ADRA2C, GRK4 and/or GRK5 genotypes. | CPIC | Annotation_of_CPIC_Guideline_for_acebutolol_atenolol_betaxolol_bisoprolol_carvedilol_esmolol_labetalol_metoprolol_nadolol_nebivolol_pindolol_pr.json |
| 94 | sotalol | ADRB1 | There are currently no recommendations for dosing of orally administered beta-blockers based on ADRB1, ADRA2C, GRK4 and/or GRK5 genotypes. | CPIC | Annotation_of_CPIC_Guideline_for_acebutolol_atenolol_betaxolol_bisoprolol_carvedilol_esmolol_labetalol_metoprolol_nadolol_nebivolol_pindolol_pr.json |
| 95 | sotalol | GRK4 | There are currently no recommendations for dosing of orally administered beta-blockers based on ADRB1, ADRA2C, GRK4 and/or GRK5 genotypes. | CPIC | Annotation_of_CPIC_Guideline_for_acebutolol_atenolol_betaxolol_bisoprolol_carvedilol_esmolol_labetalol_metoprolol_nadolol_nebivolol_pindolol_pr.json |
| 96 | sotalol | GRK5 | There are currently no recommendations for dosing of orally administered beta-blockers based on ADRB1, ADRA2C, GRK4 and/or GRK5 genotypes. | CPIC | Annotation_of_CPIC_Guideline_for_acebutolol_atenolol_betaxolol_bisoprolol_carvedilol_esmolol_labetalol_metoprolol_nadolol_nebivolol_pindolol_pr.json |
| 97 | aceclofenac | CYP2C9 | There are currently no recommendations for dosing of aceclofenac, aspirin, diclofenac, indomethacin, lumiracoxib, metamizole, nabumetone and naproxen based on CYP2C9 genotypes. | CPIC | Annotation_of_CPIC_Guideline_for_aceclofenac_aspirin_diclofenac_dipyrone_indomethacin_lumiracoxib_nabumetone_naproxen_and_CYP2C9.json |
| 98 | aspirin | CYP2C9 | There are currently no recommendations for dosing of aceclofenac, aspirin, diclofenac, indomethacin, lumiracoxib, metamizole, nabumetone and naproxen based on CYP2C9 genotypes. | CPIC | Annotation_of_CPIC_Guideline_for_aceclofenac_aspirin_diclofenac_dipyrone_indomethacin_lumiracoxib_nabumetone_naproxen_and_CYP2C9.json |
| 99 | diclofenac | CYP2C9 | There are currently no recommendations for dosing of aceclofenac, aspirin, diclofenac, indomethacin, lumiracoxib, metamizole, nabumetone and naproxen based on CYP2C9 genotypes. | CPIC | Annotation_of_CPIC_Guideline_for_aceclofenac_aspirin_diclofenac_dipyrone_indomethacin_lumiracoxib_nabumetone_naproxen_and_CYP2C9.json |
| 100 | dipyrone | CYP2C9 | There are currently no recommendations for dosing of aceclofenac, aspirin, diclofenac, indomethacin, lumiracoxib, metamizole, nabumetone and naproxen based on CYP2C9 genotypes. | CPIC | Annotation_of_CPIC_Guideline_for_aceclofenac_aspirin_diclofenac_dipyrone_indomethacin_lumiracoxib_nabumetone_naproxen_and_CYP2C9.json |
| 101 | indomethacin | CYP2C9 | There are currently no recommendations for dosing of aceclofenac, aspirin, diclofenac, indomethacin, lumiracoxib, metamizole, nabumetone and naproxen based on CYP2C9 genotypes. | CPIC | Annotation_of_CPIC_Guideline_for_aceclofenac_aspirin_diclofenac_dipyrone_indomethacin_lumiracoxib_nabumetone_naproxen_and_CYP2C9.json |
| 102 | lumiracoxib | CYP2C9 | There are currently no recommendations for dosing of aceclofenac, aspirin, diclofenac, indomethacin, lumiracoxib, metamizole, nabumetone and naproxen based on CYP2C9 genotypes. | CPIC | Annotation_of_CPIC_Guideline_for_aceclofenac_aspirin_diclofenac_dipyrone_indomethacin_lumiracoxib_nabumetone_naproxen_and_CYP2C9.json |
| 103 | nabumetone | CYP2C9 | There are currently no recommendations for dosing of aceclofenac, aspirin, diclofenac, indomethacin, lumiracoxib, metamizole, nabumetone and naproxen based on CYP2C9 genotypes. | CPIC | Annotation_of_CPIC_Guideline_for_aceclofenac_aspirin_diclofenac_dipyrone_indomethacin_lumiracoxib_nabumetone_naproxen_and_CYP2C9.json |
| 104 | naproxen | CYP2C9 | There are currently no recommendations for dosing of aceclofenac, aspirin, diclofenac, indomethacin, lumiracoxib, metamizole, nabumetone and naproxen based on CYP2C9 genotypes. | CPIC | Annotation_of_CPIC_Guideline_for_aceclofenac_aspirin_diclofenac_dipyrone_indomethacin_lumiracoxib_nabumetone_naproxen_and_CYP2C9.json |
| 105 | carbamazepine | HLA-A | The CPIC Dosing Guideline update for carbamazepine recommends an alternative drug for carbamazepine-naive patients carrying at least one copy of either HLA-B*15:02 or HLA-A*31:01 due to the association of those alleles with an increased risk of Stevens-Johnson syndrome (SJS) and toxic epidermal necrolysis (TEN). In addition, HLA-A*31:01 is associated with an increased risk of drug reaction with eosinophilia and systemic symptoms (DRESS) and maculopapular exanthema (MPE). | CPIC | Annotation_of_CPIC_Guideline_for_carbamazepine_and_HLA_A_HLA_B.json |
| 106 | carbamazepine | HLA-B | The CPIC Dosing Guideline update for carbamazepine recommends an alternative drug for carbamazepine-naive patients carrying at least one copy of either HLA-B*15:02 or HLA-A*31:01 due to the association of those alleles with an increased risk of Stevens-Johnson syndrome (SJS) and toxic epidermal necrolysis (TEN). In addition, HLA-A*31:01 is associated with an increased risk of drug reaction with eosinophilia and systemic symptoms (DRESS) and maculopapular exanthema (MPE). | CPIC | Annotation_of_CPIC_Guideline_for_carbamazepine_and_HLA_A_HLA_B.json |
| 107 | citalopram | SLC6A4 | There are currently no recommendations for dosing of escitalopram, citalopram, fluoxetine, fluvoxamine, paroxetine, sertraline, duloxetine, venlafaxine, desvenlafaxine, vilazodone, vortioxetine, levomilnacipran, or milnacipran based on SLC6A4 genotypes. | CPIC | Annotation_of_CPIC_Guideline_for_citalopram_desvenlafaxine_duloxetine_escitalopram_fluoxetine_fluvoxamine_levomilnacipran_milnacipran_paroxetin.json |
| 108 | desvenlafaxine | SLC6A4 | There are currently no recommendations for dosing of escitalopram, citalopram, fluoxetine, fluvoxamine, paroxetine, sertraline, duloxetine, venlafaxine, desvenlafaxine, vilazodone, vortioxetine, levomilnacipran, or milnacipran based on SLC6A4 genotypes. | CPIC | Annotation_of_CPIC_Guideline_for_citalopram_desvenlafaxine_duloxetine_escitalopram_fluoxetine_fluvoxamine_levomilnacipran_milnacipran_paroxetin.json |
| 109 | duloxetine | SLC6A4 | There are currently no recommendations for dosing of escitalopram, citalopram, fluoxetine, fluvoxamine, paroxetine, sertraline, duloxetine, venlafaxine, desvenlafaxine, vilazodone, vortioxetine, levomilnacipran, or milnacipran based on SLC6A4 genotypes. | CPIC | Annotation_of_CPIC_Guideline_for_citalopram_desvenlafaxine_duloxetine_escitalopram_fluoxetine_fluvoxamine_levomilnacipran_milnacipran_paroxetin.json |
| 110 | escitalopram | SLC6A4 | There are currently no recommendations for dosing of escitalopram, citalopram, fluoxetine, fluvoxamine, paroxetine, sertraline, duloxetine, venlafaxine, desvenlafaxine, vilazodone, vortioxetine, levomilnacipran, or milnacipran based on SLC6A4 genotypes. | CPIC | Annotation_of_CPIC_Guideline_for_citalopram_desvenlafaxine_duloxetine_escitalopram_fluoxetine_fluvoxamine_levomilnacipran_milnacipran_paroxetin.json |
| 111 | fluoxetine | SLC6A4 | There are currently no recommendations for dosing of escitalopram, citalopram, fluoxetine, fluvoxamine, paroxetine, sertraline, duloxetine, venlafaxine, desvenlafaxine, vilazodone, vortioxetine, levomilnacipran, or milnacipran based on SLC6A4 genotypes. | CPIC | Annotation_of_CPIC_Guideline_for_citalopram_desvenlafaxine_duloxetine_escitalopram_fluoxetine_fluvoxamine_levomilnacipran_milnacipran_paroxetin.json |
| 112 | fluvoxamine | SLC6A4 | There are currently no recommendations for dosing of escitalopram, citalopram, fluoxetine, fluvoxamine, paroxetine, sertraline, duloxetine, venlafaxine, desvenlafaxine, vilazodone, vortioxetine, levomilnacipran, or milnacipran based on SLC6A4 genotypes. | CPIC | Annotation_of_CPIC_Guideline_for_citalopram_desvenlafaxine_duloxetine_escitalopram_fluoxetine_fluvoxamine_levomilnacipran_milnacipran_paroxetin.json |
| 113 | levomilnacipran | SLC6A4 | There are currently no recommendations for dosing of escitalopram, citalopram, fluoxetine, fluvoxamine, paroxetine, sertraline, duloxetine, venlafaxine, desvenlafaxine, vilazodone, vortioxetine, levomilnacipran, or milnacipran based on SLC6A4 genotypes. | CPIC | Annotation_of_CPIC_Guideline_for_citalopram_desvenlafaxine_duloxetine_escitalopram_fluoxetine_fluvoxamine_levomilnacipran_milnacipran_paroxetin.json |
| 114 | milnacipran | SLC6A4 | There are currently no recommendations for dosing of escitalopram, citalopram, fluoxetine, fluvoxamine, paroxetine, sertraline, duloxetine, venlafaxine, desvenlafaxine, vilazodone, vortioxetine, levomilnacipran, or milnacipran based on SLC6A4 genotypes. | CPIC | Annotation_of_CPIC_Guideline_for_citalopram_desvenlafaxine_duloxetine_escitalopram_fluoxetine_fluvoxamine_levomilnacipran_milnacipran_paroxetin.json |
| 115 | paroxetine | SLC6A4 | There are currently no recommendations for dosing of escitalopram, citalopram, fluoxetine, fluvoxamine, paroxetine, sertraline, duloxetine, venlafaxine, desvenlafaxine, vilazodone, vortioxetine, levomilnacipran, or milnacipran based on SLC6A4 genotypes. | CPIC | Annotation_of_CPIC_Guideline_for_citalopram_desvenlafaxine_duloxetine_escitalopram_fluoxetine_fluvoxamine_levomilnacipran_milnacipran_paroxetin.json |
| 116 | sertraline | SLC6A4 | There are currently no recommendations for dosing of escitalopram, citalopram, fluoxetine, fluvoxamine, paroxetine, sertraline, duloxetine, venlafaxine, desvenlafaxine, vilazodone, vortioxetine, levomilnacipran, or milnacipran based on SLC6A4 genotypes. | CPIC | Annotation_of_CPIC_Guideline_for_citalopram_desvenlafaxine_duloxetine_escitalopram_fluoxetine_fluvoxamine_levomilnacipran_milnacipran_paroxetin.json |
| 117 | venlafaxine | SLC6A4 | There are currently no recommendations for dosing of escitalopram, citalopram, fluoxetine, fluvoxamine, paroxetine, sertraline, duloxetine, venlafaxine, desvenlafaxine, vilazodone, vortioxetine, levomilnacipran, or milnacipran based on SLC6A4 genotypes. | CPIC | Annotation_of_CPIC_Guideline_for_citalopram_desvenlafaxine_duloxetine_escitalopram_fluoxetine_fluvoxamine_levomilnacipran_milnacipran_paroxetin.json |
| 118 | vilazodone | SLC6A4 | There are currently no recommendations for dosing of escitalopram, citalopram, fluoxetine, fluvoxamine, paroxetine, sertraline, duloxetine, venlafaxine, desvenlafaxine, vilazodone, vortioxetine, levomilnacipran, or milnacipran based on SLC6A4 genotypes. | CPIC | Annotation_of_CPIC_Guideline_for_citalopram_desvenlafaxine_duloxetine_escitalopram_fluoxetine_fluvoxamine_levomilnacipran_milnacipran_paroxetin.json |
| 119 | vortioxetine | SLC6A4 | There are currently no recommendations for dosing of escitalopram, citalopram, fluoxetine, fluvoxamine, paroxetine, sertraline, duloxetine, venlafaxine, desvenlafaxine, vilazodone, vortioxetine, levomilnacipran, or milnacipran based on SLC6A4 genotypes. | CPIC | Annotation_of_CPIC_Guideline_for_citalopram_desvenlafaxine_duloxetine_escitalopram_fluoxetine_fluvoxamine_levomilnacipran_milnacipran_paroxetin.json |
| 120 | acebutolol | CYP2D6 | There are currently no recommendations for dosing of acebutolol, betaxolol, bisoprolol, carvedilol, nebivolol or propranolol based on CYP2D6 genotypes. | CPIC | Annotation_of_CPIC_Guideline_for_acebutolol_betaxolol_bisoprolol_carvedilol_nebivolol_propranolol_and_CYP2D6.json |
| 121 | betaxolol | CYP2D6 | There are currently no recommendations for dosing of acebutolol, betaxolol, bisoprolol, carvedilol, nebivolol or propranolol based on CYP2D6 genotypes. | CPIC | Annotation_of_CPIC_Guideline_for_acebutolol_betaxolol_bisoprolol_carvedilol_nebivolol_propranolol_and_CYP2D6.json |
| 122 | bisoprolol | CYP2D6 | There are currently no recommendations for dosing of acebutolol, betaxolol, bisoprolol, carvedilol, nebivolol or propranolol based on CYP2D6 genotypes. | CPIC | Annotation_of_CPIC_Guideline_for_acebutolol_betaxolol_bisoprolol_carvedilol_nebivolol_propranolol_and_CYP2D6.json |
| 123 | carvedilol | CYP2D6 | There are currently no recommendations for dosing of acebutolol, betaxolol, bisoprolol, carvedilol, nebivolol or propranolol based on CYP2D6 genotypes. | CPIC | Annotation_of_CPIC_Guideline_for_acebutolol_betaxolol_bisoprolol_carvedilol_nebivolol_propranolol_and_CYP2D6.json |
| 124 | nebivolol | CYP2D6 | There are currently no recommendations for dosing of acebutolol, betaxolol, bisoprolol, carvedilol, nebivolol or propranolol based on CYP2D6 genotypes. | CPIC | Annotation_of_CPIC_Guideline_for_acebutolol_betaxolol_bisoprolol_carvedilol_nebivolol_propranolol_and_CYP2D6.json |
| 125 | propranolol | CYP2D6 | There are currently no recommendations for dosing of acebutolol, betaxolol, bisoprolol, carvedilol, nebivolol or propranolol based on CYP2D6 genotypes. | CPIC | Annotation_of_CPIC_Guideline_for_acebutolol_betaxolol_bisoprolol_carvedilol_nebivolol_propranolol_and_CYP2D6.json |
| 126 | citalopram | CYP2C19 | The CPIC Dosing Guideline for the selective serotonin reuptake inhibitors citalopram and escitalopram recommends to consider a clinically appropriate antidepressant not predominantly metabolized by CYP2C19 for CYP2C19 ultrarapid, likely poor, and poor metabolizers. In case citalopram or escitalopram are clinically appropriate, dose alterations are recommended. | CPIC | Annotation_of_CPIC_Guideline_for_citalopram_escitalopram_and_CYP2C19.json |
| 127 | escitalopram | CYP2C19 | The CPIC Dosing Guideline for the selective serotonin reuptake inhibitors citalopram and escitalopram recommends to consider a clinically appropriate antidepressant not predominantly metabolized by CYP2C19 for CYP2C19 ultrarapid, likely poor, and poor metabolizers. In case citalopram or escitalopram are clinically appropriate, dose alterations are recommended. | CPIC | Annotation_of_CPIC_Guideline_for_citalopram_escitalopram_and_CYP2C19.json |
| 128 | citalopram | HTR2A | There are currently no recommendations for dosing of escitalopram and citalopram based on HTR2A genotypes. | CPIC | Annotation_of_CPIC_Guideline_for_citalopram_escitalopram_and_HTR2A.json |
| 129 | escitalopram | HTR2A | There are currently no recommendations for dosing of escitalopram and citalopram based on HTR2A genotypes. | CPIC | Annotation_of_CPIC_Guideline_for_citalopram_escitalopram_and_HTR2A.json |
| 130 | atorvastatin | CYP3A4 | There are currently no recommendations for atorvastatin, lovastatin, fluvastatin, pravastatin, pitavastatin, rosuvastatin and simvastatin dosing based on HMGCR, CYP3A4 or CYP3A5 genotypes. | CPIC | Annotation_of_CPIC_Guideline_for_atorvastatin_fluvastatin_lovastatin_pitavastatin_pravastatin_rosuvastatin_simvastatin_and_CYP3A4_CYP3A5_HMGCR.json |
| 131 | atorvastatin | CYP3A5 | There are currently no recommendations for atorvastatin, lovastatin, fluvastatin, pravastatin, pitavastatin, rosuvastatin and simvastatin dosing based on HMGCR, CYP3A4 or CYP3A5 genotypes. | CPIC | Annotation_of_CPIC_Guideline_for_atorvastatin_fluvastatin_lovastatin_pitavastatin_pravastatin_rosuvastatin_simvastatin_and_CYP3A4_CYP3A5_HMGCR.json |
| 132 | atorvastatin | HMGCR | There are currently no recommendations for atorvastatin, lovastatin, fluvastatin, pravastatin, pitavastatin, rosuvastatin and simvastatin dosing based on HMGCR, CYP3A4 or CYP3A5 genotypes. | CPIC | Annotation_of_CPIC_Guideline_for_atorvastatin_fluvastatin_lovastatin_pitavastatin_pravastatin_rosuvastatin_simvastatin_and_CYP3A4_CYP3A5_HMGCR.json |
| 133 | fluvastatin | CYP3A4 | There are currently no recommendations for atorvastatin, lovastatin, fluvastatin, pravastatin, pitavastatin, rosuvastatin and simvastatin dosing based on HMGCR, CYP3A4 or CYP3A5 genotypes. | CPIC | Annotation_of_CPIC_Guideline_for_atorvastatin_fluvastatin_lovastatin_pitavastatin_pravastatin_rosuvastatin_simvastatin_and_CYP3A4_CYP3A5_HMGCR.json |
| 134 | fluvastatin | CYP3A5 | There are currently no recommendations for atorvastatin, lovastatin, fluvastatin, pravastatin, pitavastatin, rosuvastatin and simvastatin dosing based on HMGCR, CYP3A4 or CYP3A5 genotypes. | CPIC | Annotation_of_CPIC_Guideline_for_atorvastatin_fluvastatin_lovastatin_pitavastatin_pravastatin_rosuvastatin_simvastatin_and_CYP3A4_CYP3A5_HMGCR.json |
| 135 | fluvastatin | HMGCR | There are currently no recommendations for atorvastatin, lovastatin, fluvastatin, pravastatin, pitavastatin, rosuvastatin and simvastatin dosing based on HMGCR, CYP3A4 or CYP3A5 genotypes. | CPIC | Annotation_of_CPIC_Guideline_for_atorvastatin_fluvastatin_lovastatin_pitavastatin_pravastatin_rosuvastatin_simvastatin_and_CYP3A4_CYP3A5_HMGCR.json |
| 136 | lovastatin | CYP3A4 | There are currently no recommendations for atorvastatin, lovastatin, fluvastatin, pravastatin, pitavastatin, rosuvastatin and simvastatin dosing based on HMGCR, CYP3A4 or CYP3A5 genotypes. | CPIC | Annotation_of_CPIC_Guideline_for_atorvastatin_fluvastatin_lovastatin_pitavastatin_pravastatin_rosuvastatin_simvastatin_and_CYP3A4_CYP3A5_HMGCR.json |
| 137 | lovastatin | CYP3A5 | There are currently no recommendations for atorvastatin, lovastatin, fluvastatin, pravastatin, pitavastatin, rosuvastatin and simvastatin dosing based on HMGCR, CYP3A4 or CYP3A5 genotypes. | CPIC | Annotation_of_CPIC_Guideline_for_atorvastatin_fluvastatin_lovastatin_pitavastatin_pravastatin_rosuvastatin_simvastatin_and_CYP3A4_CYP3A5_HMGCR.json |
| 138 | lovastatin | HMGCR | There are currently no recommendations for atorvastatin, lovastatin, fluvastatin, pravastatin, pitavastatin, rosuvastatin and simvastatin dosing based on HMGCR, CYP3A4 or CYP3A5 genotypes. | CPIC | Annotation_of_CPIC_Guideline_for_atorvastatin_fluvastatin_lovastatin_pitavastatin_pravastatin_rosuvastatin_simvastatin_and_CYP3A4_CYP3A5_HMGCR.json |
| 139 | pitavastatin | CYP3A4 | There are currently no recommendations for atorvastatin, lovastatin, fluvastatin, pravastatin, pitavastatin, rosuvastatin and simvastatin dosing based on HMGCR, CYP3A4 or CYP3A5 genotypes. | CPIC | Annotation_of_CPIC_Guideline_for_atorvastatin_fluvastatin_lovastatin_pitavastatin_pravastatin_rosuvastatin_simvastatin_and_CYP3A4_CYP3A5_HMGCR.json |
| 140 | pitavastatin | CYP3A5 | There are currently no recommendations for atorvastatin, lovastatin, fluvastatin, pravastatin, pitavastatin, rosuvastatin and simvastatin dosing based on HMGCR, CYP3A4 or CYP3A5 genotypes. | CPIC | Annotation_of_CPIC_Guideline_for_atorvastatin_fluvastatin_lovastatin_pitavastatin_pravastatin_rosuvastatin_simvastatin_and_CYP3A4_CYP3A5_HMGCR.json |
| 141 | pitavastatin | HMGCR | There are currently no recommendations for atorvastatin, lovastatin, fluvastatin, pravastatin, pitavastatin, rosuvastatin and simvastatin dosing based on HMGCR, CYP3A4 or CYP3A5 genotypes. | CPIC | Annotation_of_CPIC_Guideline_for_atorvastatin_fluvastatin_lovastatin_pitavastatin_pravastatin_rosuvastatin_simvastatin_and_CYP3A4_CYP3A5_HMGCR.json |
| 142 | pravastatin | CYP3A4 | There are currently no recommendations for atorvastatin, lovastatin, fluvastatin, pravastatin, pitavastatin, rosuvastatin and simvastatin dosing based on HMGCR, CYP3A4 or CYP3A5 genotypes. | CPIC | Annotation_of_CPIC_Guideline_for_atorvastatin_fluvastatin_lovastatin_pitavastatin_pravastatin_rosuvastatin_simvastatin_and_CYP3A4_CYP3A5_HMGCR.json |
| 143 | pravastatin | CYP3A5 | There are currently no recommendations for atorvastatin, lovastatin, fluvastatin, pravastatin, pitavastatin, rosuvastatin and simvastatin dosing based on HMGCR, CYP3A4 or CYP3A5 genotypes. | CPIC | Annotation_of_CPIC_Guideline_for_atorvastatin_fluvastatin_lovastatin_pitavastatin_pravastatin_rosuvastatin_simvastatin_and_CYP3A4_CYP3A5_HMGCR.json |
| 144 | pravastatin | HMGCR | There are currently no recommendations for atorvastatin, lovastatin, fluvastatin, pravastatin, pitavastatin, rosuvastatin and simvastatin dosing based on HMGCR, CYP3A4 or CYP3A5 genotypes. | CPIC | Annotation_of_CPIC_Guideline_for_atorvastatin_fluvastatin_lovastatin_pitavastatin_pravastatin_rosuvastatin_simvastatin_and_CYP3A4_CYP3A5_HMGCR.json |
| 145 | rosuvastatin | CYP3A4 | There are currently no recommendations for atorvastatin, lovastatin, fluvastatin, pravastatin, pitavastatin, rosuvastatin and simvastatin dosing based on HMGCR, CYP3A4 or CYP3A5 genotypes. | CPIC | Annotation_of_CPIC_Guideline_for_atorvastatin_fluvastatin_lovastatin_pitavastatin_pravastatin_rosuvastatin_simvastatin_and_CYP3A4_CYP3A5_HMGCR.json |
| 146 | rosuvastatin | CYP3A5 | There are currently no recommendations for atorvastatin, lovastatin, fluvastatin, pravastatin, pitavastatin, rosuvastatin and simvastatin dosing based on HMGCR, CYP3A4 or CYP3A5 genotypes. | CPIC | Annotation_of_CPIC_Guideline_for_atorvastatin_fluvastatin_lovastatin_pitavastatin_pravastatin_rosuvastatin_simvastatin_and_CYP3A4_CYP3A5_HMGCR.json |
| 147 | rosuvastatin | HMGCR | There are currently no recommendations for atorvastatin, lovastatin, fluvastatin, pravastatin, pitavastatin, rosuvastatin and simvastatin dosing based on HMGCR, CYP3A4 or CYP3A5 genotypes. | CPIC | Annotation_of_CPIC_Guideline_for_atorvastatin_fluvastatin_lovastatin_pitavastatin_pravastatin_rosuvastatin_simvastatin_and_CYP3A4_CYP3A5_HMGCR.json |
| 148 | simvastatin | CYP3A4 | There are currently no recommendations for atorvastatin, lovastatin, fluvastatin, pravastatin, pitavastatin, rosuvastatin and simvastatin dosing based on HMGCR, CYP3A4 or CYP3A5 genotypes. | CPIC | Annotation_of_CPIC_Guideline_for_atorvastatin_fluvastatin_lovastatin_pitavastatin_pravastatin_rosuvastatin_simvastatin_and_CYP3A4_CYP3A5_HMGCR.json |
| 149 | simvastatin | CYP3A5 | There are currently no recommendations for atorvastatin, lovastatin, fluvastatin, pravastatin, pitavastatin, rosuvastatin and simvastatin dosing based on HMGCR, CYP3A4 or CYP3A5 genotypes. | CPIC | Annotation_of_CPIC_Guideline_for_atorvastatin_fluvastatin_lovastatin_pitavastatin_pravastatin_rosuvastatin_simvastatin_and_CYP3A4_CYP3A5_HMGCR.json |
| 150 | simvastatin | HMGCR | There are currently no recommendations for atorvastatin, lovastatin, fluvastatin, pravastatin, pitavastatin, rosuvastatin and simvastatin dosing based on HMGCR, CYP3A4 or CYP3A5 genotypes. | CPIC | Annotation_of_CPIC_Guideline_for_atorvastatin_fluvastatin_lovastatin_pitavastatin_pravastatin_rosuvastatin_simvastatin_and_CYP3A4_CYP3A5_HMGCR.json |
| 151 | atorvastatin | ABCG2 | There are currently no recommendations for atorvastatin, lovastatin, fluvastatin, pravastatin, pitavastatin and simvastatin dosing based on ABCG2 genotypes. | CPIC | Annotation_of_CPIC_Guideline_for_atorvastatin_fluvastatin_lovastatin_pitavastatin_pravastatin_simvastatin_and_ABCG2.json |
| 152 | fluvastatin | ABCG2 | There are currently no recommendations for atorvastatin, lovastatin, fluvastatin, pravastatin, pitavastatin and simvastatin dosing based on ABCG2 genotypes. | CPIC | Annotation_of_CPIC_Guideline_for_atorvastatin_fluvastatin_lovastatin_pitavastatin_pravastatin_simvastatin_and_ABCG2.json |
| 153 | lovastatin | ABCG2 | There are currently no recommendations for atorvastatin, lovastatin, fluvastatin, pravastatin, pitavastatin and simvastatin dosing based on ABCG2 genotypes. | CPIC | Annotation_of_CPIC_Guideline_for_atorvastatin_fluvastatin_lovastatin_pitavastatin_pravastatin_simvastatin_and_ABCG2.json |
| 154 | pitavastatin | ABCG2 | There are currently no recommendations for atorvastatin, lovastatin, fluvastatin, pravastatin, pitavastatin and simvastatin dosing based on ABCG2 genotypes. | CPIC | Annotation_of_CPIC_Guideline_for_atorvastatin_fluvastatin_lovastatin_pitavastatin_pravastatin_simvastatin_and_ABCG2.json |
| 155 | pravastatin | ABCG2 | There are currently no recommendations for atorvastatin, lovastatin, fluvastatin, pravastatin, pitavastatin and simvastatin dosing based on ABCG2 genotypes. | CPIC | Annotation_of_CPIC_Guideline_for_atorvastatin_fluvastatin_lovastatin_pitavastatin_pravastatin_simvastatin_and_ABCG2.json |
| 156 | simvastatin | ABCG2 | There are currently no recommendations for atorvastatin, lovastatin, fluvastatin, pravastatin, pitavastatin and simvastatin dosing based on ABCG2 genotypes. | CPIC | Annotation_of_CPIC_Guideline_for_atorvastatin_fluvastatin_lovastatin_pitavastatin_pravastatin_simvastatin_and_ABCG2.json |
| 157 | celecoxib | CYP2C9 | The CPIC Dosing Guideline for celecoxib, flurbiprofen, ibuprofen and lornoxicam recommends initiating therapy with 25-50% of the lowest recommended starting dose for CYP2C9 poor metabolizers and initiating therapy with lowest recommended starting dose for CYP2C9 intermediate metabolizers with activity score of 1. See full guideline for further details and supporting evidence. | CPIC | Annotation_of_CPIC_Guideline_for_celecoxib_flurbiprofen_ibuprofen_lornoxicam_and_CYP2C9.json |
| 158 | flurbiprofen | CYP2C9 | The CPIC Dosing Guideline for celecoxib, flurbiprofen, ibuprofen and lornoxicam recommends initiating therapy with 25-50% of the lowest recommended starting dose for CYP2C9 poor metabolizers and initiating therapy with lowest recommended starting dose for CYP2C9 intermediate metabolizers with activity score of 1. See full guideline for further details and supporting evidence. | CPIC | Annotation_of_CPIC_Guideline_for_celecoxib_flurbiprofen_ibuprofen_lornoxicam_and_CYP2C9.json |
| 159 | ibuprofen | CYP2C9 | The CPIC Dosing Guideline for celecoxib, flurbiprofen, ibuprofen and lornoxicam recommends initiating therapy with 25-50% of the lowest recommended starting dose for CYP2C9 poor metabolizers and initiating therapy with lowest recommended starting dose for CYP2C9 intermediate metabolizers with activity score of 1. See full guideline for further details and supporting evidence. | CPIC | Annotation_of_CPIC_Guideline_for_celecoxib_flurbiprofen_ibuprofen_lornoxicam_and_CYP2C9.json |
| 160 | lornoxicam | CYP2C9 | The CPIC Dosing Guideline for celecoxib, flurbiprofen, ibuprofen and lornoxicam recommends initiating therapy with 25-50% of the lowest recommended starting dose for CYP2C9 poor metabolizers and initiating therapy with lowest recommended starting dose for CYP2C9 intermediate metabolizers with activity score of 1. See full guideline for further details and supporting evidence. | CPIC | Annotation_of_CPIC_Guideline_for_celecoxib_flurbiprofen_ibuprofen_lornoxicam_and_CYP2C9.json |
| 161 | desvenlafaxine | HTR2A | There are currently no recommendations for dosing of fluoxetine, fluvoxamine, paroxetine, sertraline, duloxetine, venlafaxine, desvenlafaxine, vilazodone, vortioxetine, levomilnacipran, or milnacipran based on HTR2A genotypes. | CPIC | Annotation_of_CPIC_Guideline_for_desvenlafaxine_duloxetine_fluoxetine_fluvoxamine_levomilnacipran_milnacipran_paroxetine_sertraline_venlafaxine.json |
| 162 | duloxetine | HTR2A | There are currently no recommendations for dosing of fluoxetine, fluvoxamine, paroxetine, sertraline, duloxetine, venlafaxine, desvenlafaxine, vilazodone, vortioxetine, levomilnacipran, or milnacipran based on HTR2A genotypes. | CPIC | Annotation_of_CPIC_Guideline_for_desvenlafaxine_duloxetine_fluoxetine_fluvoxamine_levomilnacipran_milnacipran_paroxetine_sertraline_venlafaxine.json |
| 163 | fluoxetine | HTR2A | There are currently no recommendations for dosing of fluoxetine, fluvoxamine, paroxetine, sertraline, duloxetine, venlafaxine, desvenlafaxine, vilazodone, vortioxetine, levomilnacipran, or milnacipran based on HTR2A genotypes. | CPIC | Annotation_of_CPIC_Guideline_for_desvenlafaxine_duloxetine_fluoxetine_fluvoxamine_levomilnacipran_milnacipran_paroxetine_sertraline_venlafaxine.json |
| 164 | fluvoxamine | HTR2A | There are currently no recommendations for dosing of fluoxetine, fluvoxamine, paroxetine, sertraline, duloxetine, venlafaxine, desvenlafaxine, vilazodone, vortioxetine, levomilnacipran, or milnacipran based on HTR2A genotypes. | CPIC | Annotation_of_CPIC_Guideline_for_desvenlafaxine_duloxetine_fluoxetine_fluvoxamine_levomilnacipran_milnacipran_paroxetine_sertraline_venlafaxine.json |
| 165 | levomilnacipran | HTR2A | There are currently no recommendations for dosing of fluoxetine, fluvoxamine, paroxetine, sertraline, duloxetine, venlafaxine, desvenlafaxine, vilazodone, vortioxetine, levomilnacipran, or milnacipran based on HTR2A genotypes. | CPIC | Annotation_of_CPIC_Guideline_for_desvenlafaxine_duloxetine_fluoxetine_fluvoxamine_levomilnacipran_milnacipran_paroxetine_sertraline_venlafaxine.json |
| 166 | milnacipran | HTR2A | There are currently no recommendations for dosing of fluoxetine, fluvoxamine, paroxetine, sertraline, duloxetine, venlafaxine, desvenlafaxine, vilazodone, vortioxetine, levomilnacipran, or milnacipran based on HTR2A genotypes. | CPIC | Annotation_of_CPIC_Guideline_for_desvenlafaxine_duloxetine_fluoxetine_fluvoxamine_levomilnacipran_milnacipran_paroxetine_sertraline_venlafaxine.json |
| 167 | paroxetine | HTR2A | There are currently no recommendations for dosing of fluoxetine, fluvoxamine, paroxetine, sertraline, duloxetine, venlafaxine, desvenlafaxine, vilazodone, vortioxetine, levomilnacipran, or milnacipran based on HTR2A genotypes. | CPIC | Annotation_of_CPIC_Guideline_for_desvenlafaxine_duloxetine_fluoxetine_fluvoxamine_levomilnacipran_milnacipran_paroxetine_sertraline_venlafaxine.json |
| 168 | sertraline | HTR2A | There are currently no recommendations for dosing of fluoxetine, fluvoxamine, paroxetine, sertraline, duloxetine, venlafaxine, desvenlafaxine, vilazodone, vortioxetine, levomilnacipran, or milnacipran based on HTR2A genotypes. | CPIC | Annotation_of_CPIC_Guideline_for_desvenlafaxine_duloxetine_fluoxetine_fluvoxamine_levomilnacipran_milnacipran_paroxetine_sertraline_venlafaxine.json |
| 169 | venlafaxine | HTR2A | There are currently no recommendations for dosing of fluoxetine, fluvoxamine, paroxetine, sertraline, duloxetine, venlafaxine, desvenlafaxine, vilazodone, vortioxetine, levomilnacipran, or milnacipran based on HTR2A genotypes. | CPIC | Annotation_of_CPIC_Guideline_for_desvenlafaxine_duloxetine_fluoxetine_fluvoxamine_levomilnacipran_milnacipran_paroxetine_sertraline_venlafaxine.json |
| 170 | vilazodone | HTR2A | There are currently no recommendations for dosing of fluoxetine, fluvoxamine, paroxetine, sertraline, duloxetine, venlafaxine, desvenlafaxine, vilazodone, vortioxetine, levomilnacipran, or milnacipran based on HTR2A genotypes. | CPIC | Annotation_of_CPIC_Guideline_for_desvenlafaxine_duloxetine_fluoxetine_fluvoxamine_levomilnacipran_milnacipran_paroxetine_sertraline_venlafaxine.json |
| 171 | vortioxetine | HTR2A | There are currently no recommendations for dosing of fluoxetine, fluvoxamine, paroxetine, sertraline, duloxetine, venlafaxine, desvenlafaxine, vilazodone, vortioxetine, levomilnacipran, or milnacipran based on HTR2A genotypes. | CPIC | Annotation_of_CPIC_Guideline_for_desvenlafaxine_duloxetine_fluoxetine_fluvoxamine_levomilnacipran_milnacipran_paroxetine_sertraline_venlafaxine.json |
| 172 | codeine | CYP2D6 | Alternate non-tramadol analgesics are recommended for CYP2D6 ultrarapid and poor metabolizers. A label recommended age- or weight-specific dose of codeine is warranted for CYP2D6 normal and intermediate metabolizers. | CPIC | Annotation_of_CPIC_Guideline_for_codeine_and_CYP2D6.json |
| 173 | carvedilol | ADRB2 | There are currently no recommendations for dosing of carvedilol, labetalol, nadolol, pindolol, propranolol or sotalol based on ADRB2 genotypes. | CPIC | Annotation_of_CPIC_Guideline_for_carvedilol_labetalol_nadolol_pindolol_propranolol_sotalol_and_ADRB2.json |
| 174 | labetalol | ADRB2 | There are currently no recommendations for dosing of carvedilol, labetalol, nadolol, pindolol, propranolol or sotalol based on ADRB2 genotypes. | CPIC | Annotation_of_CPIC_Guideline_for_carvedilol_labetalol_nadolol_pindolol_propranolol_sotalol_and_ADRB2.json |
| 175 | nadolol | ADRB2 | There are currently no recommendations for dosing of carvedilol, labetalol, nadolol, pindolol, propranolol or sotalol based on ADRB2 genotypes. | CPIC | Annotation_of_CPIC_Guideline_for_carvedilol_labetalol_nadolol_pindolol_propranolol_sotalol_and_ADRB2.json |
| 176 | pindolol | ADRB2 | There are currently no recommendations for dosing of carvedilol, labetalol, nadolol, pindolol, propranolol or sotalol based on ADRB2 genotypes. | CPIC | Annotation_of_CPIC_Guideline_for_carvedilol_labetalol_nadolol_pindolol_propranolol_sotalol_and_ADRB2.json |
| 177 | propranolol | ADRB2 | There are currently no recommendations for dosing of carvedilol, labetalol, nadolol, pindolol, propranolol or sotalol based on ADRB2 genotypes. | CPIC | Annotation_of_CPIC_Guideline_for_carvedilol_labetalol_nadolol_pindolol_propranolol_sotalol_and_ADRB2.json |
| 178 | sotalol | ADRB2 | There are currently no recommendations for dosing of carvedilol, labetalol, nadolol, pindolol, propranolol or sotalol based on ADRB2 genotypes. | CPIC | Annotation_of_CPIC_Guideline_for_carvedilol_labetalol_nadolol_pindolol_propranolol_sotalol_and_ADRB2.json |
| 179 | desipramine | CYP2D6 | Tricyclic antidepressants have comparable pharmacokinetic properties, it may be reasonable to apply the CPIC Dosing Guideline for amitriptyline/nortriptyline and CYP2C19, CYP2D6 to other tricyclics including desipramine. The CPIC Dosing Guideline update for nortriptyline recommends a 25% dose reduction for CYP2D6 intermediate metabolizers. For CYP2D6 ultrarapid or poor metabolizers, an alternative drug should be considered. If nortriptyline is warranted, consider a 50% dose reduction in CYP2D6 poor metabolizers. | CPIC | Annotation_of_CPIC_Guideline_for_desipramine_and_CYP2D6.json |
| 180 | clomipramine | CYP2C19 | Tricyclic antidepressants have comparable pharmacokinetic properties, it may be reasonable to apply the CPIC Dosing Guideline for amitriptyline and CYP2C19, CYP2D6 to other tricyclics including clomipramine. The CPIC Dosing Guideline update for amitriptyline recommends an alternative drug for CYP2D6 ultrarapid or poor metabolizers and CYP2C19 ultrarapid, rapid or poor metabolizers. If amitriptyline is warranted, consider a 50% dose reduction in CYP2D6 or CYP2C19 poor metabolizers. For CYP2D6 intermediate metabolizers, a 25% dose reduction should be considered. | CPIC | Annotation_of_CPIC_Guideline_for_clomipramine_and_CYP2C19_CYP2D6.json |
| 181 | clomipramine | CYP2D6 | Tricyclic antidepressants have comparable pharmacokinetic properties, it may be reasonable to apply the CPIC Dosing Guideline for amitriptyline and CYP2C19, CYP2D6 to other tricyclics including clomipramine. The CPIC Dosing Guideline update for amitriptyline recommends an alternative drug for CYP2D6 ultrarapid or poor metabolizers and CYP2C19 ultrarapid, rapid or poor metabolizers. If amitriptyline is warranted, consider a 50% dose reduction in CYP2D6 or CYP2C19 poor metabolizers. For CYP2D6 intermediate metabolizers, a 25% dose reduction should be considered. | CPIC | Annotation_of_CPIC_Guideline_for_clomipramine_and_CYP2C19_CYP2D6.json |
| 182 | clopidogrel | CYP2C19 | The CPIC Dosing Guideline for clopidogrel recommends an alternative antiplatelet therapy for CYP2C19 poor or intermediate metabolizers (cardiovascular indications: prasugrel or ticagrelor if no contraindication; neurovascular indications: alternative P2Y12 inhibitor if clinically indicated and no contraindication.) | CPIC | Annotation_of_CPIC_Guideline_for_clopidogrel_and_CYP2C19.json |
| 183 | desflurane | CACNA1S | The CPIC Dosing Guideline recommends that halogenated volatile anesthetics such as desflurane, enflurane, halothane, isoflurane, methoxyflurane, sevoflurane and the depolarizing muscle relaxants succinylcholine are relatively contraindicated in persons with malignant hyperthermia susceptibility (MHS). See full guideline for disclaimers, further details and supporting evidence. | CPIC | Annotation_of_CPIC_Guideline_for_desflurane_enflurane_halothane_isoflurane_methoxyflurane_sevoflurane_succinylcholine_and_CACNA1S_RYR1.json |
| 184 | desflurane | RYR1 | The CPIC Dosing Guideline recommends that halogenated volatile anesthetics such as desflurane, enflurane, halothane, isoflurane, methoxyflurane, sevoflurane and the depolarizing muscle relaxants succinylcholine are relatively contraindicated in persons with malignant hyperthermia susceptibility (MHS). See full guideline for disclaimers, further details and supporting evidence. | CPIC | Annotation_of_CPIC_Guideline_for_desflurane_enflurane_halothane_isoflurane_methoxyflurane_sevoflurane_succinylcholine_and_CACNA1S_RYR1.json |
| 185 | enflurane | CACNA1S | The CPIC Dosing Guideline recommends that halogenated volatile anesthetics such as desflurane, enflurane, halothane, isoflurane, methoxyflurane, sevoflurane and the depolarizing muscle relaxants succinylcholine are relatively contraindicated in persons with malignant hyperthermia susceptibility (MHS). See full guideline for disclaimers, further details and supporting evidence. | CPIC | Annotation_of_CPIC_Guideline_for_desflurane_enflurane_halothane_isoflurane_methoxyflurane_sevoflurane_succinylcholine_and_CACNA1S_RYR1.json |
| 186 | enflurane | RYR1 | The CPIC Dosing Guideline recommends that halogenated volatile anesthetics such as desflurane, enflurane, halothane, isoflurane, methoxyflurane, sevoflurane and the depolarizing muscle relaxants succinylcholine are relatively contraindicated in persons with malignant hyperthermia susceptibility (MHS). See full guideline for disclaimers, further details and supporting evidence. | CPIC | Annotation_of_CPIC_Guideline_for_desflurane_enflurane_halothane_isoflurane_methoxyflurane_sevoflurane_succinylcholine_and_CACNA1S_RYR1.json |
| 187 | halothane | CACNA1S | The CPIC Dosing Guideline recommends that halogenated volatile anesthetics such as desflurane, enflurane, halothane, isoflurane, methoxyflurane, sevoflurane and the depolarizing muscle relaxants succinylcholine are relatively contraindicated in persons with malignant hyperthermia susceptibility (MHS). See full guideline for disclaimers, further details and supporting evidence. | CPIC | Annotation_of_CPIC_Guideline_for_desflurane_enflurane_halothane_isoflurane_methoxyflurane_sevoflurane_succinylcholine_and_CACNA1S_RYR1.json |
| 188 | halothane | RYR1 | The CPIC Dosing Guideline recommends that halogenated volatile anesthetics such as desflurane, enflurane, halothane, isoflurane, methoxyflurane, sevoflurane and the depolarizing muscle relaxants succinylcholine are relatively contraindicated in persons with malignant hyperthermia susceptibility (MHS). See full guideline for disclaimers, further details and supporting evidence. | CPIC | Annotation_of_CPIC_Guideline_for_desflurane_enflurane_halothane_isoflurane_methoxyflurane_sevoflurane_succinylcholine_and_CACNA1S_RYR1.json |
| 189 | isoflurane | CACNA1S | The CPIC Dosing Guideline recommends that halogenated volatile anesthetics such as desflurane, enflurane, halothane, isoflurane, methoxyflurane, sevoflurane and the depolarizing muscle relaxants succinylcholine are relatively contraindicated in persons with malignant hyperthermia susceptibility (MHS). See full guideline for disclaimers, further details and supporting evidence. | CPIC | Annotation_of_CPIC_Guideline_for_desflurane_enflurane_halothane_isoflurane_methoxyflurane_sevoflurane_succinylcholine_and_CACNA1S_RYR1.json |
| 190 | isoflurane | RYR1 | The CPIC Dosing Guideline recommends that halogenated volatile anesthetics such as desflurane, enflurane, halothane, isoflurane, methoxyflurane, sevoflurane and the depolarizing muscle relaxants succinylcholine are relatively contraindicated in persons with malignant hyperthermia susceptibility (MHS). See full guideline for disclaimers, further details and supporting evidence. | CPIC | Annotation_of_CPIC_Guideline_for_desflurane_enflurane_halothane_isoflurane_methoxyflurane_sevoflurane_succinylcholine_and_CACNA1S_RYR1.json |
| 191 | methoxyflurane | CACNA1S | The CPIC Dosing Guideline recommends that halogenated volatile anesthetics such as desflurane, enflurane, halothane, isoflurane, methoxyflurane, sevoflurane and the depolarizing muscle relaxants succinylcholine are relatively contraindicated in persons with malignant hyperthermia susceptibility (MHS). See full guideline for disclaimers, further details and supporting evidence. | CPIC | Annotation_of_CPIC_Guideline_for_desflurane_enflurane_halothane_isoflurane_methoxyflurane_sevoflurane_succinylcholine_and_CACNA1S_RYR1.json |
| 192 | methoxyflurane | RYR1 | The CPIC Dosing Guideline recommends that halogenated volatile anesthetics such as desflurane, enflurane, halothane, isoflurane, methoxyflurane, sevoflurane and the depolarizing muscle relaxants succinylcholine are relatively contraindicated in persons with malignant hyperthermia susceptibility (MHS). See full guideline for disclaimers, further details and supporting evidence. | CPIC | Annotation_of_CPIC_Guideline_for_desflurane_enflurane_halothane_isoflurane_methoxyflurane_sevoflurane_succinylcholine_and_CACNA1S_RYR1.json |
| 193 | sevoflurane | CACNA1S | The CPIC Dosing Guideline recommends that halogenated volatile anesthetics such as desflurane, enflurane, halothane, isoflurane, methoxyflurane, sevoflurane and the depolarizing muscle relaxants succinylcholine are relatively contraindicated in persons with malignant hyperthermia susceptibility (MHS). See full guideline for disclaimers, further details and supporting evidence. | CPIC | Annotation_of_CPIC_Guideline_for_desflurane_enflurane_halothane_isoflurane_methoxyflurane_sevoflurane_succinylcholine_and_CACNA1S_RYR1.json |
| 194 | sevoflurane | RYR1 | The CPIC Dosing Guideline recommends that halogenated volatile anesthetics such as desflurane, enflurane, halothane, isoflurane, methoxyflurane, sevoflurane and the depolarizing muscle relaxants succinylcholine are relatively contraindicated in persons with malignant hyperthermia susceptibility (MHS). See full guideline for disclaimers, further details and supporting evidence. | CPIC | Annotation_of_CPIC_Guideline_for_desflurane_enflurane_halothane_isoflurane_methoxyflurane_sevoflurane_succinylcholine_and_CACNA1S_RYR1.json |
| 195 | succinylcholine | CACNA1S | The CPIC Dosing Guideline recommends that halogenated volatile anesthetics such as desflurane, enflurane, halothane, isoflurane, methoxyflurane, sevoflurane and the depolarizing muscle relaxants succinylcholine are relatively contraindicated in persons with malignant hyperthermia susceptibility (MHS). See full guideline for disclaimers, further details and supporting evidence. | CPIC | Annotation_of_CPIC_Guideline_for_desflurane_enflurane_halothane_isoflurane_methoxyflurane_sevoflurane_succinylcholine_and_CACNA1S_RYR1.json |
| 196 | succinylcholine | RYR1 | The CPIC Dosing Guideline recommends that halogenated volatile anesthetics such as desflurane, enflurane, halothane, isoflurane, methoxyflurane, sevoflurane and the depolarizing muscle relaxants succinylcholine are relatively contraindicated in persons with malignant hyperthermia susceptibility (MHS). See full guideline for disclaimers, further details and supporting evidence. | CPIC | Annotation_of_CPIC_Guideline_for_desflurane_enflurane_halothane_isoflurane_methoxyflurane_sevoflurane_succinylcholine_and_CACNA1S_RYR1.json |
| 197 | duloxetine | CYP2D6 | There are currently no recommendations for dosing of duloxetine based on CYP2D6 genotypes. | CPIC | Annotation_of_CPIC_Guideline_for_duloxetine_and_CYP2D6.json |
| 198 | doxepin | CYP2C19 | Tricyclic antidepressants have comparable pharmacokinetic properties, it may be reasonable to apply the CPIC Dosing Guideline for amitriptyline and CYP2C19, CYP2D6 to other tricyclics including doxepin. The CPIC Dosing Guideline update for amitriptyline recommends an alternative drug for CYP2D6 ultrarapid or poor metabolizers and CYP2C19 ultrarapid, rapid or poor metabolizers. If amitriptyline is warranted, consider a 50% dose reduction in CYP2D6 or CYP2C19 poor metabolizers. For CYP2D6 intermediate metabolizers, a 25% dose reduction should be considered. | CPIC | Annotation_of_CPIC_Guideline_for_doxepin_and_CYP2C19_CYP2D6.json |
| 199 | doxepin | CYP2D6 | Tricyclic antidepressants have comparable pharmacokinetic properties, it may be reasonable to apply the CPIC Dosing Guideline for amitriptyline and CYP2C19, CYP2D6 to other tricyclics including doxepin. The CPIC Dosing Guideline update for amitriptyline recommends an alternative drug for CYP2D6 ultrarapid or poor metabolizers and CYP2C19 ultrarapid, rapid or poor metabolizers. If amitriptyline is warranted, consider a 50% dose reduction in CYP2D6 or CYP2C19 poor metabolizers. For CYP2D6 intermediate metabolizers, a 25% dose reduction should be considered. | CPIC | Annotation_of_CPIC_Guideline_for_doxepin_and_CYP2C19_CYP2D6.json |
| 200 | fluoxetine | CYP2D6 | There are currently no recommendations for dosing of fluoxetine based on CYP2D6 genotypes. | CPIC | Annotation_of_CPIC_Guideline_for_fluoxetine_and_CYP2D6.json |
| 201 | fluorouracil | DPYD | The CPIC Dosing Guideline for 5-fluorouracil and capecitabine recommends an alternative drug for patients who are DPYD poor metabolizers with an activity score of 0. In those who are poor metabolizers with an activity score of 0.5, an alternative drug is also recommended, but if this is not considered a suitable therapeutic option, 5-fluorouracil or capecitabine should be administered at a strongly reduced dose with early therapeutic drug monitoring. Patients who are intermediate metabolizers with an activity score of 1 or 1.5 should receive a dose reduction of 50%. Patients with the c.[2846A>T];[2846A>T] genotype may require a >50% dose reduction. | CPIC | Annotation_of_CPIC_Guideline_for_fluorouracil_and_DPYD.json |
| 202 | fluvoxamine | CYP2D6 | The CPIC Dosing Guideline for the selective serotonin reuptake inhibitor fluvoxamine recommends to consider a 25-50% reduction of recommended starting dose and slower titration schedule or use an alternative drug not metabolized by CYP2D6 for CYP2D6 poor metabolizers. | CPIC | Annotation_of_CPIC_Guideline_for_fluvoxamine_and_CYP2D6.json |
| 203 | hydrocodone | CYP2D6 | CYP2D6 intermediate and poor metabolizers should initiate hydrocodone therapy using the label recommended age- or weight-specific dosing. However, if there is no response to hydrocodone in these patients, an alternative analgesic should be considered. There is insufficient evidence to provide a dosing recommendation for hydrocodone in CYP2D6 ultrarapid metabolizers. | CPIC | Annotation_of_CPIC_Guideline_for_hydrocodone_and_CYP2D6.json |
| 204 | dexlansoprazole | CYP2C19 | The CPIC Dosing Guideline recommendations for dexlansoprazole are based on the similarity in its metabolism and lansoprazole and extrapolated from the first-generation PPIs. The guideline recommends to increase the starting daily dose and to monitor efficacy in CYP2C19 ultrarapid metabolizer. For CYP2C19 rapid and normal metabolizers in the treatment of H. pylori infection and erosive esophagitis increasing the dose might be considered after initiation with the standard starting daily dose. The recommendations for intermediate and poor metabolizers for chronic therapy (>12 weeks) and efficacy achieved is to consider 50% reduction in daily dose. See full guideline for further details and supporting evidence. | CPIC | Annotation_of_CPIC_Guideline_for_dexlansoprazole_and_CYP2C19.json |
| 205 | fosphenytoin | CYP2C9 | Phenytoin/fosphenytoin is contraindicated in individuals with the HLA-B*15:02 variant allele ("HLA-B*15:02-positive") due to significantly increased risk of phenytoin-induced cutaneous adverse reactions of Stevens-Johnson syndrome (SJS) and toxic epidermal necrolysis (TEN). Additionally, patients with the CYP2C9 poor metabolizer phenotype or with a CYP2C9 activity score of 1 may require reduced doses of phenytoin/fosphenytoin. | CPIC | Annotation_of_CPIC_Guideline_for_fosphenytoin_phenytoin_and_CYP2C9_HLA_B.json |
| 206 | fosphenytoin | HLA-B | Phenytoin/fosphenytoin is contraindicated in individuals with the HLA-B*15:02 variant allele ("HLA-B*15:02-positive") due to significantly increased risk of phenytoin-induced cutaneous adverse reactions of Stevens-Johnson syndrome (SJS) and toxic epidermal necrolysis (TEN). Additionally, patients with the CYP2C9 poor metabolizer phenotype or with a CYP2C9 activity score of 1 may require reduced doses of phenytoin/fosphenytoin. | CPIC | Annotation_of_CPIC_Guideline_for_fosphenytoin_phenytoin_and_CYP2C9_HLA_B.json |
| 207 | phenytoin | CYP2C9 | Phenytoin/fosphenytoin is contraindicated in individuals with the HLA-B*15:02 variant allele ("HLA-B*15:02-positive") due to significantly increased risk of phenytoin-induced cutaneous adverse reactions of Stevens-Johnson syndrome (SJS) and toxic epidermal necrolysis (TEN). Additionally, patients with the CYP2C9 poor metabolizer phenotype or with a CYP2C9 activity score of 1 may require reduced doses of phenytoin/fosphenytoin. | CPIC | Annotation_of_CPIC_Guideline_for_fosphenytoin_phenytoin_and_CYP2C9_HLA_B.json |
| 208 | phenytoin | HLA-B | Phenytoin/fosphenytoin is contraindicated in individuals with the HLA-B*15:02 variant allele ("HLA-B*15:02-positive") due to significantly increased risk of phenytoin-induced cutaneous adverse reactions of Stevens-Johnson syndrome (SJS) and toxic epidermal necrolysis (TEN). Additionally, patients with the CYP2C9 poor metabolizer phenotype or with a CYP2C9 activity score of 1 may require reduced doses of phenytoin/fosphenytoin. | CPIC | Annotation_of_CPIC_Guideline_for_fosphenytoin_phenytoin_and_CYP2C9_HLA_B.json |
| 209 | efavirenz | CYP2B6 | Consider initiating efavirenz with a decreased dose of either 400 or 200 mg/day for patients who are CYP2B6 poor metabolizers. Consider initiating efavirenz with a decreased dose of 400 mg/day for patients who are CYP2B6 intermediate metabolizers. | CPIC | Annotation_of_CPIC_Guideline_for_efavirenz_and_CYP2B6.json |
| 210 | imipramine | CYP2C19 | Tricyclic antidepressants have comparable pharmacokinetic properties, it may be reasonable to apply the CPIC Dosing Guideline for amitriptyline and CYP2C19, CYP2D6 to other tricyclics including imipramine. The CPIC Dosing Guideline update for amitriptyline recommends an alternative drug for CYP2D6 ultrarapid or poor metabolizers and CYP2C19 ultrarapid, rapid or poor metabolizers. If amitriptyline is warranted, consider a 50% dose reduction in CYP2D6 or CYP2C19 poor metabolizers. For CYP2D6 intermediate metabolizers, a 25% dose reduction should be considered. | CPIC | Annotation_of_CPIC_Guideline_for_imipramine_and_CYP2C19_CYP2D6.json |
| 211 | imipramine | CYP2D6 | Tricyclic antidepressants have comparable pharmacokinetic properties, it may be reasonable to apply the CPIC Dosing Guideline for amitriptyline and CYP2C19, CYP2D6 to other tricyclics including imipramine. The CPIC Dosing Guideline update for amitriptyline recommends an alternative drug for CYP2D6 ultrarapid or poor metabolizers and CYP2C19 ultrarapid, rapid or poor metabolizers. If amitriptyline is warranted, consider a 50% dose reduction in CYP2D6 or CYP2C19 poor metabolizers. For CYP2D6 intermediate metabolizers, a 25% dose reduction should be considered. | CPIC | Annotation_of_CPIC_Guideline_for_imipramine_and_CYP2C19_CYP2D6.json |
| 212 | diclofenac | CYP2C8 | There are currently no recommendations for NSAIDs dosing based on CYP2C8 genotypes. | CPIC | Annotation_of_CPIC_Guideline_for_diclofenac_ibuprofen_and_CYP2C8.json |
| 213 | ibuprofen | CYP2C8 | There are currently no recommendations for NSAIDs dosing based on CYP2C8 genotypes. | CPIC | Annotation_of_CPIC_Guideline_for_diclofenac_ibuprofen_and_CYP2C8.json |
| 214 | esomeprazole | CYP2C19 | The CPIC Dosing Guideline for CYP2C19 and Proton Pump Inhibitor Dosing states that inconsistent findings regarding the effect of CYP2C19 genotype on the pharmacokinetics and therapeutic response to esomeprazole and rabeprazole preclude making recommendations for these proton pump inhibitors. | CPIC | Annotation_of_CPIC_Guideline_for_esomeprazole_rabeprazole_and_CYP2C19.json |
| 215 | rabeprazole | CYP2C19 | The CPIC Dosing Guideline for CYP2C19 and Proton Pump Inhibitor Dosing states that inconsistent findings regarding the effect of CYP2C19 genotype on the pharmacokinetics and therapeutic response to esomeprazole and rabeprazole preclude making recommendations for these proton pump inhibitors. | CPIC | Annotation_of_CPIC_Guideline_for_esomeprazole_rabeprazole_and_CYP2C19.json |
| 216 | methadone | COMT | There are currently no recommendations for dosing of methadone or oxycodone based on CYP2D6, OPRM1 or COMT genotypes. | CPIC | Annotation_of_CPIC_Guideline_for_methadone_oxycodone_and_COMT_CYP2D6_OPRM1.json |
| 217 | methadone | CYP2D6 | There are currently no recommendations for dosing of methadone or oxycodone based on CYP2D6, OPRM1 or COMT genotypes. | CPIC | Annotation_of_CPIC_Guideline_for_methadone_oxycodone_and_COMT_CYP2D6_OPRM1.json |
| 218 | methadone | OPRM1 | There are currently no recommendations for dosing of methadone or oxycodone based on CYP2D6, OPRM1 or COMT genotypes. | CPIC | Annotation_of_CPIC_Guideline_for_methadone_oxycodone_and_COMT_CYP2D6_OPRM1.json |
| 219 | oxycodone | COMT | There are currently no recommendations for dosing of methadone or oxycodone based on CYP2D6, OPRM1 or COMT genotypes. | CPIC | Annotation_of_CPIC_Guideline_for_methadone_oxycodone_and_COMT_CYP2D6_OPRM1.json |
| 220 | oxycodone | CYP2D6 | There are currently no recommendations for dosing of methadone or oxycodone based on CYP2D6, OPRM1 or COMT genotypes. | CPIC | Annotation_of_CPIC_Guideline_for_methadone_oxycodone_and_COMT_CYP2D6_OPRM1.json |
| 221 | oxycodone | OPRM1 | There are currently no recommendations for dosing of methadone or oxycodone based on CYP2D6, OPRM1 or COMT genotypes. | CPIC | Annotation_of_CPIC_Guideline_for_methadone_oxycodone_and_COMT_CYP2D6_OPRM1.json |
| 222 | lansoprazole | CYP2C19 | The CPIC Dosing Guideline for omeprazole, lansoprazole, pantoprazole recommends to increase the starting daily dose and to monitor efficacy in CYP2C19 ultrarapid metabolizer. For CYP2C19 rapid and normal metabolizers in the treatment of H. pylori infection and erosive esophagitis increasing the dose might be considered after initiation with the standard starting daily dose. The recommendations for intermediate and poor metabolizer for chronic therapy (>12 weeks) and efficacy achieved is to consider 50% reduction in daily dose. See full guideline for further details and supporting evidence. | CPIC | Annotation_of_CPIC_Guideline_for_lansoprazole_omeprazole_pantoprazole_and_CYP2C19.json |
| 223 | omeprazole | CYP2C19 | The CPIC Dosing Guideline for omeprazole, lansoprazole, pantoprazole recommends to increase the starting daily dose and to monitor efficacy in CYP2C19 ultrarapid metabolizer. For CYP2C19 rapid and normal metabolizers in the treatment of H. pylori infection and erosive esophagitis increasing the dose might be considered after initiation with the standard starting daily dose. The recommendations for intermediate and poor metabolizer for chronic therapy (>12 weeks) and efficacy achieved is to consider 50% reduction in daily dose. See full guideline for further details and supporting evidence. | CPIC | Annotation_of_CPIC_Guideline_for_lansoprazole_omeprazole_pantoprazole_and_CYP2C19.json |
| 224 | pantoprazole | CYP2C19 | The CPIC Dosing Guideline for omeprazole, lansoprazole, pantoprazole recommends to increase the starting daily dose and to monitor efficacy in CYP2C19 ultrarapid metabolizer. For CYP2C19 rapid and normal metabolizers in the treatment of H. pylori infection and erosive esophagitis increasing the dose might be considered after initiation with the standard starting daily dose. The recommendations for intermediate and poor metabolizer for chronic therapy (>12 weeks) and efficacy achieved is to consider 50% reduction in daily dose. See full guideline for further details and supporting evidence. | CPIC | Annotation_of_CPIC_Guideline_for_lansoprazole_omeprazole_pantoprazole_and_CYP2C19.json |
| 225 | mercaptopurine | NUDT15 | Consider an alternate agent or extreme dose reduction of mercaptopurine for patients who are TPMT or NUDT15 poor metabolizers. Start at 30-80% of target dose for patients who are intermediate metabolizers of either TPMT or NUDT15. Start at 20-50% of target dose for patients who are intermediate metabolizers of both TPMT and NUDT15. | CPIC | Annotation_of_CPIC_Guideline_for_mercaptopurine_and_NUDT15_TPMT.json |
| 226 | mercaptopurine | TPMT | Consider an alternate agent or extreme dose reduction of mercaptopurine for patients who are TPMT or NUDT15 poor metabolizers. Start at 30-80% of target dose for patients who are intermediate metabolizers of either TPMT or NUDT15. Start at 20-50% of target dose for patients who are intermediate metabolizers of both TPMT and NUDT15. | CPIC | Annotation_of_CPIC_Guideline_for_mercaptopurine_and_NUDT15_TPMT.json |
| 227 | nortriptyline | CYP2D6 | The CPIC Dosing Guideline update for nortriptyline recommends a 25% dose reduction for CYP2D6 intermediate metabolizers. For CYP2D6 ultrarapid or poor metabolizers, an alternative drug should be considered. If nortriptyline is warranted, consider a 50% dose reduction in CYP2D6 poor metabolizers. | CPIC | Annotation_of_CPIC_Guideline_for_nortriptyline_and_CYP2D6.json |
| 228 | fluvastatin | CYP2C9 | CYP2C9 IMs should avoid fluvastatin doses greater than 40mg while CYP2C9 PMs should avoid doses greater than 20mg. If higher doses are required for desired efficacy, an alternative statin should be considered. Patients with SLCO1B1 poor function should also avoid fluvastatin doses greater than 40mg and and adjust doses of fluvastatin based on disease-specific guidelines. Patients with both SLCO1B1 poor function and CYP2C9 IM/PM should be prescribed an alternative statin depending on the desired potency. | CPIC | Annotation_of_CPIC_Guideline_for_fluvastatin_and_CYP2C9_SLCO1B1.json |
| 229 | fluvastatin | SLCO1B1 | CYP2C9 IMs should avoid fluvastatin doses greater than 40mg while CYP2C9 PMs should avoid doses greater than 20mg. If higher doses are required for desired efficacy, an alternative statin should be considered. Patients with SLCO1B1 poor function should also avoid fluvastatin doses greater than 40mg and and adjust doses of fluvastatin based on disease-specific guidelines. Patients with both SLCO1B1 poor function and CYP2C9 IM/PM should be prescribed an alternative statin depending on the desired potency. | CPIC | Annotation_of_CPIC_Guideline_for_fluvastatin_and_CYP2C9_SLCO1B1.json |
| 230 | lovastatin | SLCO1B1 | Prescribe an alternative statin depending on the desired potency for patients with SLCO1B1 decreased function, possible decreased function or poor function phenotype. If lovastatin therapy is warranted in patients with SLCO1B1 decreased or possible decreased phenotype, limit dose to <20mg/day. | CPIC | Annotation_of_CPIC_Guideline_for_lovastatin_and_SLCO1B1.json |
| 231 | ivacaftor | CFTR | Ivacaftor treatment is recommended only in cystic fibrosis (CF) patients that are either homozygous or heterozygous for certain CFTR variants. See full guideline for disclaimers, further details and supporting evidence. | CPIC | Annotation_of_CPIC_Guideline_for_ivacaftor_and_CFTR.json |
| 232 | ondansetron | CYP2D6 | The CPIC dosing guideline for ondansetron recommends selecting an alternate drug for CYP2D6 ultrarapid metabolizers. It is recommended that the alternate drug not be predominantly metabolized by CYP2D6 (eg. granisetron). | CPIC | Annotation_of_CPIC_Guideline_for_ondansetron_and_CYP2D6.json |
| 233 | meloxicam | CYP2C9 | The CPIC Dosing Guideline for meloxicam recommends alternative therapy for CYP2C9 poor metabolizers due to markedly prolonged half-life, and initiating therapy with 50% of the lowest recommended starting dose or choose an alternative therapy for CYP2C9 intermediate metabolizers with activity score of 1. See full guideline for further details and supporting evidence. | CPIC | Annotation_of_CPIC_Guideline_for_meloxicam_and_CYP2C9.json |
| 234 | oxcarbazepine | HLA-B | The CPIC Dosing Guideline for oxcarbazepine recommends an alternative drug for oxcarbazepine-naive patients carrying at least one copy of HLA-B*15:02 due to the association of this allele with an increased risk of Stevens-Johnson syndrome (SJS) and toxic epidermal necrolysis (TEN). | CPIC | Annotation_of_CPIC_Guideline_for_oxcarbazepine_and_HLA_B.json |
| 235 | peginterferon alfa-2a | IFNL3 | IFNL3 (IL28B) variation (rs12979860) is the strongest baseline predictor of response to PEG-interferon-alpha-containing regimens in HCV genotype 1 patients. Patients with the favorable response genotype (rs12979860 CC) have increased likelihood of response (higher SVR rate) to PEG-interferon-alpha-containing regimens as compared to patients with unfavorable response genotype (rs12979860 CT or TT). Consider implications before initiating PEG-IFN alpha and RBV containing regimens. | CPIC | Annotation_of_CPIC_Guideline_for_peginterferon_alfa_2a_peginterferon_alfa_2b_ribavirin_and_IFNL3.json |
| 236 | peginterferon alfa-2b | IFNL3 | IFNL3 (IL28B) variation (rs12979860) is the strongest baseline predictor of response to PEG-interferon-alpha-containing regimens in HCV genotype 1 patients. Patients with the favorable response genotype (rs12979860 CC) have increased likelihood of response (higher SVR rate) to PEG-interferon-alpha-containing regimens as compared to patients with unfavorable response genotype (rs12979860 CT or TT). Consider implications before initiating PEG-IFN alpha and RBV containing regimens. | CPIC | Annotation_of_CPIC_Guideline_for_peginterferon_alfa_2a_peginterferon_alfa_2b_ribavirin_and_IFNL3.json |
| 237 | ribavirin | IFNL3 | IFNL3 (IL28B) variation (rs12979860) is the strongest baseline predictor of response to PEG-interferon-alpha-containing regimens in HCV genotype 1 patients. Patients with the favorable response genotype (rs12979860 CC) have increased likelihood of response (higher SVR rate) to PEG-interferon-alpha-containing regimens as compared to patients with unfavorable response genotype (rs12979860 CT or TT). Consider implications before initiating PEG-IFN alpha and RBV containing regimens. | CPIC | Annotation_of_CPIC_Guideline_for_peginterferon_alfa_2a_peginterferon_alfa_2b_ribavirin_and_IFNL3.json |
| 238 | paroxetine | CYP2D6 | The CPIC Dosing Guideline for the selective serotonin reuptake inhibitor paroxetine recommends an alternative drug not predominantly metabolized by CYP2D6 for CYP2D6 ultrarapid metabolizers. For CYP2D6 poor metabolizers, consider a 50% reduction in recommended starting dose, slower titration schedule, and a 50% lower maintenance dose. | CPIC | Annotation_of_CPIC_Guideline_for_paroxetine_and_CYP2D6.json |
| 239 | tacrolimus | CYP3A5 | The CPIC dosing guideline for tacrolimus recommends increasing the starting dose by 1.5 to 2 times the recommended starting dose in patients who are CYP3A5 intermediate or extensive metabolizers, though total starting dose should not exceed 0.3 mg/kg/day. Therapeutic drug monitoring should also be used to guide dose adjustments. | CPIC | Annotation_of_CPIC_Guideline_for_tacrolimus_and_CYP3A5.json |
| 240 | tegafur | DPYD | As of the November 2017 update of CPIC guidelines regarding fluoropyrimidine dosing, there are no longer dosing recommendations for tegafur based on DPYD genotype. This is due to limited evidence regarding the impact of DPYD variants on tegafur toxicity risk. The guidelines currently only apply to 5-fluorouracil and capecitabine dosing. | CPIC | Annotation_of_CPIC_Guideline_for_tegafur_and_DPYD.json |
| 241 | metoprolol | CYP2D6 | Patients who are CYP2D6 poor metabolizers should initiate metoprolol therapy at the lowest recommended starting dose and carefully titrate upwards to clinical effect or guideline-recommended dose. Alternatively, a different beta-blocker may be selected. There is no recommendation for CYP2D6 ultrarapid metabolizers. | CPIC | Annotation_of_CPIC_Guideline_for_metoprolol_and_CYP2D6.json |
| 242 | methadone | CYP2B6 | CPIC does not currently provide CYP2B6 genotype-based prescribing recommendations for methadone. | CPIC | Annotation_of_CPIC_Guideline_for_methadone_and_CYP2B6.json |
| 243 | pitavastatin | SLCO1B1 | Prescribe ≤1mg as a starting dose for patients with SLCO1B1 poor function phenotype. Prescribe ≤2mg as a starting dose for patients with SLCO1B1 decreased or possible decreased phenotype. Adjust doses of pitavastatin based on disease-specific guidelines. Consider an alternative statin or combination therapy if higher doses are needed. | CPIC | Annotation_of_CPIC_Guideline_for_pitavastatin_and_SLCO1B1.json |
| 244 | sertraline | CYP2B6 | The CPIC Dosing Guideline for the selective serotonin reuptake inhibitor sertraline recommends to consider a 50% reduction of recommended starting dose and titrate to response or select alternative drug not predominantly metabolized by CYP2C19 for CYP2C19 poor metabolizers. | CPIC | Annotation_of_CPIC_Guideline_for_sertraline_and_CYP2B6_CYP2C19.json |
| 245 | sertraline | CYP2C19 | The CPIC Dosing Guideline for the selective serotonin reuptake inhibitor sertraline recommends to consider a 50% reduction of recommended starting dose and titrate to response or select alternative drug not predominantly metabolized by CYP2C19 for CYP2C19 poor metabolizers. | CPIC | Annotation_of_CPIC_Guideline_for_sertraline_and_CYP2B6_CYP2C19.json |
| 246 | pravastatin | SLCO1B1 | Prescribe ≤40mg as a starting dose and adjust doses of pravastatin based on disease-specific guidelines for patients with SLCO1B1 poor function phenotype. Prescribe desired starting dose and adjust doses of pravastatin based on disease-specific guidelines for patients with SLCO1B1 decreased or possible decreased phenotype. Prescriber should be aware of possible increased risk for myopathy with pravastatin especially with doses >40mg per day. | CPIC | Annotation_of_CPIC_Guideline_for_pravastatin_and_SLCO1B1.json |
| 247 | rosuvastatin | ABCG2 | Prescribe ≤20mg as a starting dose and adjust doses of rosuvastatin based on disease-specific and specific population guidelines for patients who are SLCO1B1 or ABCG2 poor function phenotype. If dose >20mg needed for desired efficacy, consider combination therapy (i.e. rosuvastatin plus non-statin guideline directed medical therapy). Patients with both ABCG2 poor function and SLCO1B1 poor/decreased function should be prescribed ≤10mg as a starting dose. | CPIC | Annotation_of_CPIC_Guideline_for_rosuvastatin_and_ABCG2_SLCO1B1.json |
| 248 | rosuvastatin | SLCO1B1 | Prescribe ≤20mg as a starting dose and adjust doses of rosuvastatin based on disease-specific and specific population guidelines for patients who are SLCO1B1 or ABCG2 poor function phenotype. If dose >20mg needed for desired efficacy, consider combination therapy (i.e. rosuvastatin plus non-statin guideline directed medical therapy). Patients with both ABCG2 poor function and SLCO1B1 poor/decreased function should be prescribed ≤10mg as a starting dose. | CPIC | Annotation_of_CPIC_Guideline_for_rosuvastatin_and_ABCG2_SLCO1B1.json |
| 249 | piroxicam | CYP2C9 | The CPIC Dosing Guideline for piroxicam recommends that CYP2C9 poor metabolizers and intermediate metabolizers with activity score of 1 should choose an alternative therapy not metabolized by CYP2C9 or not significantly impacted by CYP2C9 genetic variants in vivo or choose an NSAID metabolized by CYP2C9 but with a shorter half-life. See full guideline for further details and supporting evidence. | CPIC | Annotation_of_CPIC_Guideline_for_piroxicam_and_CYP2C9.json |
| 250 | tamoxifen | CYP2D6 | The CPIC Dosing Guideline for tamoxifen recommends the use of alternative hormonal therapy such as an aromatase inhibitor for postmenopausal women or aromatase inhibitor along with ovarian function suppression in premenopausal women for CYP2D6 poor metabolizer, if aromatase inhibitor use is not contraindicated. For CYP2D6 intermediate metabolizers and CYP2D6 allele combinations resulting in an activity score (AS) of 1 the recommendation is to consider the recommendations stated for the CYP2D6 poor metabolizer. If aromatase inhibitor use is contraindicated, consideration should be given to use a higher but FDA approved tamoxifen dose for CYP2D6 intermediate metabolizers and CYP2D6 allele combinations resulting in an AS of 1. For poor metabolizer, higher dose tamoxifen (40 mg/day) increases but does not normalize endoxifen concentrations and can be considered if there are contraindications to aromatase inhibitor therapy. | CPIC | Annotation_of_CPIC_Guideline_for_tamoxifen_and_CYP2D6.json |
| 251 | tramadol | CYP2D6 | Alternate non-codeine analgesics are recommended for CYP2D6 ultrarapid and poor metabolizers. A label recommended age- or weight-specific dose of tramadol is warranted for CYP2D6 normal and intermediate metabolizers. | CPIC | Annotation_of_CPIC_Guideline_for_tramadol_and_CYP2D6.json |
| 252 | simvastatin | SLCO1B1 | Prescribe an alternative statin depending on the desired potency for patients with SLCO1B1 decreased function, possible decreased function or poor function phenotype. If simvastatin therapy is warranted in patients with SLCO1B1 decreased or possible decreased phenotype, limit dose to <20mg/day. | CPIC | Annotation_of_CPIC_Guideline_for_simvastatin_and_SLCO1B1.json |
| 253 | trimipramine | CYP2C19 | Tricyclic antidepressants have comparable pharmacokinetic properties, it may be reasonable to apply the CPIC Dosing Guideline for amitriptyline and CYP2C19, CYP2D6 to other tricyclics including trimipramine. The CPIC Dosing Guideline update for amitriptyline recommends an alternative drug for CYP2D6 ultrarapid or poor metabolizers and CYP2C19 ultrarapid, rapid or poor metabolizers. If amitriptyline is warranted, consider a 50% dose reduction in CYP2D6 or CYP2C19 poor metabolizers. For CYP2D6 intermediate metabolizers, a 25% dose reduction should be considered. | CPIC | Annotation_of_CPIC_Guideline_for_trimipramine_and_CYP2C19_CYP2D6.json |
| 254 | trimipramine | CYP2D6 | Tricyclic antidepressants have comparable pharmacokinetic properties, it may be reasonable to apply the CPIC Dosing Guideline for amitriptyline and CYP2C19, CYP2D6 to other tricyclics including trimipramine. The CPIC Dosing Guideline update for amitriptyline recommends an alternative drug for CYP2D6 ultrarapid or poor metabolizers and CYP2C19 ultrarapid, rapid or poor metabolizers. If amitriptyline is warranted, consider a 50% dose reduction in CYP2D6 or CYP2C19 poor metabolizers. For CYP2D6 intermediate metabolizers, a 25% dose reduction should be considered. | CPIC | Annotation_of_CPIC_Guideline_for_trimipramine_and_CYP2C19_CYP2D6.json |
| 255 | thioguanine | NUDT15 | Consider an alternate agent or extreme dose reduction of thioguanine for patients who are TPMT or NUDT15 poor metabolizers. Start at 50-80% of target dose for patients who are intermediate metabolizers of either TPMT or NUDT15. Start at 20-50% of target dose for patients who are intermediate metabolizers of both TPMT and NUDT15. | CPIC | Annotation_of_CPIC_Guideline_for_thioguanine_and_NUDT15_TPMT.json |
| 256 | thioguanine | TPMT | Consider an alternate agent or extreme dose reduction of thioguanine for patients who are TPMT or NUDT15 poor metabolizers. Start at 50-80% of target dose for patients who are intermediate metabolizers of either TPMT or NUDT15. Start at 20-50% of target dose for patients who are intermediate metabolizers of both TPMT and NUDT15. | CPIC | Annotation_of_CPIC_Guideline_for_thioguanine_and_NUDT15_TPMT.json |
| 257 | tropisetron | CYP2D6 | The CPIC dosing guideline for tropisetron recommends selecting an alternate drug for CYP2D6 ultrarapid metabolizers. It is recommended that the alternate drug not be predominantly metabolized by CYP2D6 (eg. granisetron). | CPIC | Annotation_of_CPIC_Guideline_for_tropisetron_and_CYP2D6.json |
| 258 | voriconazole | CYP2C19 | The CPIC dosing guideline for voriconazole recommends selecting an alternative agent that is not dependent on CYP2C19 metabolism in adults who are CYP2C19 ultrarapid metabolizers, rapid metabolizers or poor metabolizers. In pediatric patients, an alternative agent should be used in patients who are ultrarapid metabolizers or poor metabolizers. In pediatric rapid metabolizers, therapy should be initiated at recommended standard case dosing, then therapeutic dosing monitoring should be used to titrate dose to therapeutic trough concentrations. | CPIC | Annotation_of_CPIC_Guideline_for_voriconazole_and_CYP2C19.json |
| 259 | vortioxetine | CYP2D6 | The CPIC Dosing Guideline for vortioxetine recommends for CYP2D6 ultrarapid metabolizers to select alternative drug not predominantly metabolized by CYP2D6. For poor metabolizer, initiate 50% of starting dose and titrate to the maximum recommended dose of 10 mg or consider a clinically appropriate alternative antidepressant not predominantly metabolized by CYP2D6. | CPIC | Annotation_of_CPIC_Guideline_for_vortioxetine_and_CYP2D6.json |
| 260 | venlafaxine | CYP2D6 | The CPIC Dosing Guideline for venlafaxine recommends to consider a clinically appropriate alternative antidepressant not predominantly metabolized by CYP2D6 for CYP2D6 poor metabolizers. | CPIC | Annotation_of_CPIC_Guideline_for_venlafaxine_and_CYP2D6.json |
| 261 | tenoxicam | CYP2C9 | The CPIC Dosing Guideline for tenoxicam recommends that CYP2C9 poor metabolizers and intermediate metabolizers with activity score of 1 should choose an alternative therapy not metabolized by CYP2C9 or not significantly impacted by CYP2C9 genetic variants in vivo or choose an NSAID metabolized by CYP2C9 but with a shorter half-life. See full guideline for further details and supporting evidence. | CPIC | Annotation_of_CPIC_Guideline_for_tenoxicam_and_CYP2C9.json |
| 262 | warfarin | CYP2C9 | The updated guideline for pharmacogenetics-guided warfarin dosing is published by the Clinical Pharmacogenetics Implementation Consortium. The recommendations for dosing are for adult and pediatric patients that are specific to continental ancestry, and are based on genotypes from CYP2C9, VKORC1, CYP4F2, and rs12777823. | CPIC | Annotation_of_CPIC_Guideline_for_warfarin_and_CYP2C9_CYP4F2_VKORC1.json |
| 263 | warfarin | CYP4F2 | The updated guideline for pharmacogenetics-guided warfarin dosing is published by the Clinical Pharmacogenetics Implementation Consortium. The recommendations for dosing are for adult and pediatric patients that are specific to continental ancestry, and are based on genotypes from CYP2C9, VKORC1, CYP4F2, and rs12777823. | CPIC | Annotation_of_CPIC_Guideline_for_warfarin_and_CYP2C9_CYP4F2_VKORC1.json |
| 264 | warfarin | VKORC1 | The updated guideline for pharmacogenetics-guided warfarin dosing is published by the Clinical Pharmacogenetics Implementation Consortium. The recommendations for dosing are for adult and pediatric patients that are specific to continental ancestry, and are based on genotypes from CYP2C9, VKORC1, CYP4F2, and rs12777823. | CPIC | Annotation_of_CPIC_Guideline_for_warfarin_and_CYP2C9_CYP4F2_VKORC1.json |
| 265 | tamoxifen | CYP2D6 | The Canadian Pharmacogenomics Network for Drug Safety (CPNDS) clinical recommendation group has published clinical practice guidelines for CYP2D6 as a treatment decision aid for ER-positive non-metastatic breast cancer patients. The guidelines recommend that alternatives to standard tamoxifen treatments may be considered in CYP2D6 poor or intermediate metabolizers with an emphasis on the use of comprehensive CYP2D6 genotyping panels in guiding treatment decisions. | CPNDS | Annotation_of_CPNDS_Guideline_for_tamoxifen_and_CYP2D6.json |
| 266 | cisplatin | TPMT | The Canadian Pharmacogenomics Network for Drug Safety (CPNDS) clinical recommendation group has published guidelines for the use of pharmacogenetic testing for TPMT gene variants when prescribing cisplatin in pediatric cancer patients. They recommend testing for the TPMT alleles *2, *3A, *3B or *3C in all pediatric cancer patients due to the association of these alleles with an increased risk of cisplatin-induced ototoxicity. | CPNDS | Annotation_of_CPNDS_Guideline_for_cisplatin_and_TPMT.json |
| 267 | abacavir | HLA-B | Avoid abacavir for patients who have the HLA-B*57:01 allele (patients who are "HLA-B*57:01-positive"). | DPWG | Annotation_of_DPWG_Guideline_for_abacavir_and_HLA_B.json |
| 268 | acenocoumarol | CYP2C9 | There are currently no recommendations for acenocoumarol dosing based on CYP2C9 genotypes. | DPWG | Annotation_of_DPWG_Guideline_for_acenocoumarol_and_CYP2C9.json |
| 269 | acenocoumarol | VKORC1 | Patients with the VKORC1 rs9923231 TT genotype (-1639 AA genotype) should be given 50% of the standard initial dose of acenocoumarol and undergo more frequent INR monitoring. There are no recommendation for patients with the VKORC1 rs9923231 CT genotype (-1639 AG genotype). | DPWG | Annotation_of_DPWG_Guideline_for_acenocoumarol_and_VKORC1.json |
| 270 | carbamazepine | HLA-A | The Canadian Pharmacogenomics Network for Drug Safety (CPNDS) clinical recommendation group has published guidelines for the use of HLA-A*31:01 or HLA-B*15:02 genotype when prescribing carbamazepine (CBZ). They recommend that carbamazepine not be prescribed for CBZ-naive patients who carry at least one HLA-A*31:01 or HLA-B*15:02 allele. | CPNDS | Annotation_of_CPNDS_Guideline_for_carbamazepine_and_HLA_A_HLA_B.json |
| 271 | carbamazepine | HLA-B | The Canadian Pharmacogenomics Network for Drug Safety (CPNDS) clinical recommendation group has published guidelines for the use of HLA-A*31:01 or HLA-B*15:02 genotype when prescribing carbamazepine (CBZ). They recommend that carbamazepine not be prescribed for CBZ-naive patients who carry at least one HLA-A*31:01 or HLA-B*15:02 allele. | CPNDS | Annotation_of_CPNDS_Guideline_for_carbamazepine_and_HLA_A_HLA_B.json |
| 272 | codeine | CYP2D6 | The Canadian Pharmacogenomics Network for Drug Safety (CPNDS) clinical recommendation group has published guidelines for the use of CYP2D6 genotype when prescribing codeine. They recommend that poor metabolizers of CYP2D6 should not receive codeine for pain relief, and ultrametabolizers of CYP2D6 should avoid codeine for pain relief. | CPNDS | Annotation_of_CPNDS_Guideline_for_codeine_and_CYP2D6.json |
| 273 | allopurinol | ABCG2 | Patients with the GT or TT genotypes at rs2231142 should be given an increased dose of allopurinol. | DPWG | Annotation_of_DPWG_Guideline_for_allopurinol_and_ABCG2.json |
| 274 | allopurinol | HLA-B | Individuals with the HLA-B*58:01 allele should be given an alternative drug or undergo induction of allopurinol tolerance. | DPWG | Annotation_of_DPWG_Guideline_for_allopurinol_and_HLA_B.json |
| 275 | warfarin | CYP2C9 | The Canadian Pharmacogenomics Network for Drug Safety (CPNDS) clinical recommendation group has published guidelines for the use of pharmacogenetic testing for variants in VKORC1 and CYP2C9 in adult and pediatric patients with an indication for warfarin. They recommend testing for the VKORC1 SNP -1639G>A (rs9923231) and the CYP2C9 alleles *2 and *3 in order to better guide warfarin dosage. | CPNDS | Annotation_of_CPNDS_Guideline_for_warfarin_and_CYP2C9_VKORC1.json |
| 276 | warfarin | VKORC1 | The Canadian Pharmacogenomics Network for Drug Safety (CPNDS) clinical recommendation group has published guidelines for the use of pharmacogenetic testing for variants in VKORC1 and CYP2C9 in adult and pediatric patients with an indication for warfarin. They recommend testing for the VKORC1 SNP -1639G>A (rs9923231) and the CYP2C9 alleles *2 and *3 in order to better guide warfarin dosage. | CPNDS | Annotation_of_CPNDS_Guideline_for_warfarin_and_CYP2C9_VKORC1.json |
| 277 | daunorubicin | RARG | The Canadian Pharmacogenomics Network for Drug Safety (CPNDS) clinical recommendation group has published guidelines for the use of RARG , SLC28A3 , and UGT1A6 when prescribing anthracyclines for pediatric cancer patients. They recommend that pharmacogenomic testing of RARG rs2229774, SLC28A3 rs7853758, and UGT1A6 *4 (rs17863783) should be performed in all pediatric cancer patients who are treated with daunorubicin or doxorubicin because the association of those genetic variants with anthracycline associated cardiotoxicity (ACT). | CPNDS | Annotation_of_CPNDS_Guideline_for_daunorubicin_doxorubicin_and_RARG_SLC28A3_UGT1A6.json |
| 278 | daunorubicin | SLC28A3 | The Canadian Pharmacogenomics Network for Drug Safety (CPNDS) clinical recommendation group has published guidelines for the use of RARG , SLC28A3 , and UGT1A6 when prescribing anthracyclines for pediatric cancer patients. They recommend that pharmacogenomic testing of RARG rs2229774, SLC28A3 rs7853758, and UGT1A6 *4 (rs17863783) should be performed in all pediatric cancer patients who are treated with daunorubicin or doxorubicin because the association of those genetic variants with anthracycline associated cardiotoxicity (ACT). | CPNDS | Annotation_of_CPNDS_Guideline_for_daunorubicin_doxorubicin_and_RARG_SLC28A3_UGT1A6.json |
| 279 | daunorubicin | UGT1A6 | The Canadian Pharmacogenomics Network for Drug Safety (CPNDS) clinical recommendation group has published guidelines for the use of RARG , SLC28A3 , and UGT1A6 when prescribing anthracyclines for pediatric cancer patients. They recommend that pharmacogenomic testing of RARG rs2229774, SLC28A3 rs7853758, and UGT1A6 *4 (rs17863783) should be performed in all pediatric cancer patients who are treated with daunorubicin or doxorubicin because the association of those genetic variants with anthracycline associated cardiotoxicity (ACT). | CPNDS | Annotation_of_CPNDS_Guideline_for_daunorubicin_doxorubicin_and_RARG_SLC28A3_UGT1A6.json |
| 280 | doxorubicin | RARG | The Canadian Pharmacogenomics Network for Drug Safety (CPNDS) clinical recommendation group has published guidelines for the use of RARG , SLC28A3 , and UGT1A6 when prescribing anthracyclines for pediatric cancer patients. They recommend that pharmacogenomic testing of RARG rs2229774, SLC28A3 rs7853758, and UGT1A6 *4 (rs17863783) should be performed in all pediatric cancer patients who are treated with daunorubicin or doxorubicin because the association of those genetic variants with anthracycline associated cardiotoxicity (ACT). | CPNDS | Annotation_of_CPNDS_Guideline_for_daunorubicin_doxorubicin_and_RARG_SLC28A3_UGT1A6.json |
| 281 | doxorubicin | SLC28A3 | The Canadian Pharmacogenomics Network for Drug Safety (CPNDS) clinical recommendation group has published guidelines for the use of RARG , SLC28A3 , and UGT1A6 when prescribing anthracyclines for pediatric cancer patients. They recommend that pharmacogenomic testing of RARG rs2229774, SLC28A3 rs7853758, and UGT1A6 *4 (rs17863783) should be performed in all pediatric cancer patients who are treated with daunorubicin or doxorubicin because the association of those genetic variants with anthracycline associated cardiotoxicity (ACT). | CPNDS | Annotation_of_CPNDS_Guideline_for_daunorubicin_doxorubicin_and_RARG_SLC28A3_UGT1A6.json |
| 282 | doxorubicin | UGT1A6 | The Canadian Pharmacogenomics Network for Drug Safety (CPNDS) clinical recommendation group has published guidelines for the use of RARG , SLC28A3 , and UGT1A6 when prescribing anthracyclines for pediatric cancer patients. They recommend that pharmacogenomic testing of RARG rs2229774, SLC28A3 rs7853758, and UGT1A6 *4 (rs17863783) should be performed in all pediatric cancer patients who are treated with daunorubicin or doxorubicin because the association of those genetic variants with anthracycline associated cardiotoxicity (ACT). | CPNDS | Annotation_of_CPNDS_Guideline_for_daunorubicin_doxorubicin_and_RARG_SLC28A3_UGT1A6.json |
| 283 | amitriptyline | CYP2D6 | The Dutch Pharmacogenetics Working Group Guideline for amitriptyline recommends decreasing the dose for CYP2D6 intermediate and CYP2D6 poor metabolizers and increasing the dose or using an alternative drug for CYP2D6 ultra-rapid metabolizers. | DPWG | Annotation_of_DPWG_Guideline_for_amitriptyline_and_CYP2D6.json |
| 284 | aripiprazole | CYP2D6 | The Royal Dutch Pharmacists Association - Pharmacogenetics Working Group (DPWG) recommends reducing maximum dose of aripiprazole for patients carrying poor metabolizer alleles of CYP2D6. | DPWG | Annotation_of_DPWG_Guideline_for_aripiprazole_and_CYP2D6.json |
| 285 | atorvastatin | SLCO1B1 | Choose an alternative for patients with the SLCO1B1 521 CC or TC (rs4149056) genotype and with ADDITIONAL SIGNIFICANT RISK FACTORS for statin-induced myopathy. For patients without additional significant risk factors for statin-induced myopathy, advise the patients to contact their doctor in the event of muscle symptoms. | DPWG | Annotation_of_DPWG_Guideline_for_atorvastatin_and_SLCO1B1.json |
| 286 | amiodarone | CYP2D6 | There are currently no dosing recommendations for amiodarone based on CYP2D6 genotype. | DPWG | Annotation_of_DPWG_Guideline_for_amiodarone_and_CYP2D6.json |
| 287 | atomoxetine | CYP2D6 | The Dutch Pharmacogenetics Working Group Guideline for atomoxetine states for CYP2D6 ultrarapid metabolizers, to be alert to reduced efficacy of atomoxetine or select an alternative drug as a precaution. Be alert to side effects in CYP2D6 poor metabolizers. | DPWG | Annotation_of_DPWG_Guideline_for_atomoxetine_and_CYP2D6.json |
| 288 | amitriptyline | CYP2C19 | There are currently no dosing recommendations for amitriptyline based on CYP2C19 genotype. | DPWG | Annotation_of_DPWG_Guideline_for_amitriptyline_and_CYP2C19.json |
| 289 | atenolol | CYP2D6 | There are currently no dosing recommendations for atenolol based on CYP2D6 genotype. | DPWG | Annotation_of_DPWG_Guideline_for_atenolol_and_CYP2D6.json |
| 290 | azathioprine | NUDT15 | Select an alternative drug or reduce the initial dose of azathioprine for patients that are NUDT15 poor metabolizers and reduce initial dose for patients that are NUDT15 intermediate metabolizers. | DPWG | Annotation_of_DPWG_Guideline_for_azathioprine_and_NUDT15.json |
| 291 | azathioprine | TPMT | Select an alternative drug or reduce the initial dose of azathioprine for patients that are TPMT poor metabolizers and reduce initial dose for patients that are TPMT intermediate metabolizers. | DPWG | Annotation_of_DPWG_Guideline_for_azathioprine_and_TPMT.json |
| 292 | brexpiprazole | CYP2D6 | The Royal Dutch Pharmacists Association - Pharmacogenetics Working Group (DPWG) recommends to use half of the normal dose of brexpiprazole for poor metabolizers of CYP2D6. | DPWG | Annotation_of_DPWG_Guideline_for_brexpiprazole_and_CYP2D6.json |
| 293 | bisoprolol | CYP2D6 | There are currently no dosing recommendations for bisoprolol based on CYP2D6 genotype. | DPWG | Annotation_of_DPWG_Guideline_for_bisoprolol_and_CYP2D6.json |
| 294 | capecitabine | DPYD | An alternative drug to capecitabine is recommended for patients with a DPYD activity score of 0; if an alternative drug is not possible, the DPD activity should be determined and the initial dose adjusted accordingly. For patients with a DPYD activity score of 1 or 1.5, start with 50% of the standard dose or choose an alternative drug; adjustment of subsequent doses should be guided by toxicity and effectiveness. Patients with two partially functional alleles or one non-functional and one partially functional allele or two gene variants leading to partially functional alleles or a gene variant leading to a non-functional allele and a gene variant leading to a partially functional allele should have their DPD activity determined or should avoid fluorouracil/capecitabine. Tegafur is not an alternative for capecitabine, as it is also metabolized by DPD. The DPWG evaluated DPYD genotyping as "essential" and recommend DPYD testing prior to initiating fluoropyrimidines. | DPWG | Annotation_of_DPWG_Guideline_for_capecitabine_and_DPYD.json |
| 295 | carvedilol | CYP2D6 | There are currently no dosing recommendations for carvedilol based on CYP2D6 genotype. | DPWG | Annotation_of_DPWG_Guideline_for_carvedilol_and_CYP2D6.json |
| 296 | clomipramine | CYP2C19 | The Royal Dutch Pharmacists Association - Pharmacogenetics Working Group (DPWG) recommends to avoid clomipramine in CYP2C19 ultrarapid metabolizer (UM) for Indication OBSESSIVE COMPULSIVE DISORDER or ANXIETY DISORDERS. | DPWG | Annotation_of_DPWG_Guideline_for_clomipramine_and_CYP2C19.json |
| 297 | carbamazepine | HLA-B | Consider an alternative drug to carbamazepine in patients with the HLA-B*15:02 or HLA-B*15:11 alleles. | DPWG | Annotation_of_DPWG_Guideline_for_carbamazepine_and_HLA_B.json |
| 298 | clopidogrel | CYP2C19 | Avoid clopidogrel use in patients who are CYP2C19 poor metabolizers and are undergoing percutaneous coronary intervention, stroke or TIA. For CYP2C19 intermediate metabolizers who are undergoing percutaneous coronary intervention, stroke, or TIA, choose an alternative drug or double the dose to 150 mg/day (600 mg loading dose). No action is required for patients who are CYP2C19 ultra-rapid metabolizers. | DPWG | Annotation_of_DPWG_Guideline_for_clopidogrel_and_CYP2C19.json |
| 299 | citalopram | CYP2C19 | The Dutch Pharmacogenetics Working Group Guideline for citalopram recommends for intermediate and poor metabolizers of CYP2C19 to not exceed the in the DPWG document specified daily doses. | DPWG | Annotation_of_DPWG_Guideline_for_citalopram_and_CYP2C19.json |
| 300 | clomipramine | CYP2D6 | The Dutch Pharmacogenetics Working Group Guideline for clomipramine recommends dose changes and to monitor the effect and side effects and the plasma concentrations to set the maintenance dose for CYP2D6 poor (PM), and intermediate (IM) metabolizer or to avoid clomipramine in PM and ultrarapid (UM) metabolizer. | DPWG | Annotation_of_DPWG_Guideline_for_clomipramine_and_CYP2D6.json |
| 301 | carbamazepine | HLA-A | Consider an alternative drug to carbamazepine in patients with the HLA-A*3101 allele. | DPWG | Annotation_of_DPWG_Guideline_for_carbamazepine_and_HLA_A.json |
| 302 | citalopram | CYP2D6 | There are currently no dosing recommendations for citalopram/escitalopram based on CYP2D6 genotype. | DPWG | Annotation_of_DPWG_Guideline_for_citalopram_escitalopram_and_CYP2D6.json |
| 303 | escitalopram | CYP2D6 | There are currently no dosing recommendations for citalopram/escitalopram based on CYP2D6 genotype. | DPWG | Annotation_of_DPWG_Guideline_for_citalopram_escitalopram_and_CYP2D6.json |
| 304 | clonidine | CYP2D6 | There are currently no dosing recommendations for clonidine based on CYP2D6 genotype and DPWG suggest clonidine as possible alternative for atomoxetine in variant CYP2D6 metabolisers. | DPWG | Annotation_of_DPWG_Guideline_for_clonidine_and_CYP2D6.json |
| 305 | clozapine | CYP1A2 | There is no gene-drug interaction between CYP1A2 and clozapine. | DPWG | Annotation_of_DPWG_Guideline_for_clozapine_and_CYP1A2.json |
| 306 | clozapine | CYP2D6 | There are currently no dosing recommendations for clozapine based on CYP2D6 genotypes. | DPWG | Annotation_of_DPWG_Guideline_for_clozapine_and_CYP2D6.json |
| 307 | codeine | CYP2D6 | The Pharmacogenetics Working Group Guideline for codeine includes individual recommendations for cough or pain for CYP2D6 poor, intermediate, and ultrarapid metabolizer. In addition, for ultrarapid metabolizer, higher or lower doses and additional risk factors are taken into consideration. | DPWG | Annotation_of_DPWG_Guideline_for_codeine_and_CYP2D6.json |
| 308 | duloxetine | CYP2D6 | There are currently no dosing recommendations for duloxetine based on CYP2D6 genotype. | DPWG | Annotation_of_DPWG_Guideline_for_duloxetine_and_CYP2D6.json |
| 309 | doxepin | CYP2D6 | The Dutch Pharmacogenetics Working Group Guideline for doxepin recommends dose changes and to monitor the effect and side effects or the plasma concentrations to set the maintenance dose for CYP2D6 poor (PM), intermediate (IM), and ultrarapid (UM) metabolizer or to avoid doxepin in UM. | DPWG | Annotation_of_DPWG_Guideline_for_doxepin_and_CYP2D6.json |
| 310 | disopyramide | CYP2D6 | There are currently no dosing recommendations for disopyramide based on CYP2D6 genotype. | DPWG | Annotation_of_DPWG_Guideline_for_disopyramide_and_CYP2D6.json |
| 311 | doxepin | CYP2C19 | There are currently no dosing recommendations for doxepin based on CYP2C19 genotype. | DPWG | Annotation_of_DPWG_Guideline_for_doxepin_and_CYP2C19.json |
| 312 | eliglustat | CYP2D6 | The Dutch Pharmacogenetics Working Group Guideline for eliglustat recommends to use an alternative in CYP2D6 ultrarapid metabolizer. For CYP2D6 poor metabolizer in combination with CYP3A inhibitors and strong inducers, the guideline recommends to choose an alternative if possible. For intermediate metabolizers recommendations are provided for co-medication with CYP2D6 and/or CYP3A inhibitors and CYP3A inducers. | DPWG | Annotation_of_DPWG_Guideline_for_eliglustat_and_CYP2D6.json |
| 313 | efavirenz | CYP2B6 | Adjust the initial efavirenz dose for patients with the CYP2B6 PM phenotype along with the consideration of age, weight and BMI and titrate the dose to plasma concentration if needed. For patients with the CYP2B6 IM phenotype, determine the efavirenz plasma concentration if side effects occur and reduce the dose if needed. | DPWG | Annotation_of_DPWG_Guideline_for_efavirenz_and_CYP2B6.json |
| 314 | escitalopram | CYP2C19 | The Dutch Pharmacogenetics Working Group Guideline for escitalopram recommends for intermediate and poor metabolizers of CYP2C19 to not exceed the in the DPWG document specified doses and for CYP2C19 ultrarapid metabolizer to avoid escitalopram. | DPWG | Annotation_of_DPWG_Guideline_for_escitalopram_and_CYP2C19.json |
| 315 | esomeprazole | CYP2C19 | No action is needed for this gene-drug interaction. | DPWG | Annotation_of_DPWG_Guideline_for_esomeprazole_and_CYP2C19.json |
| 316 | flucloxacillin | HLA-B | Monitor for liver function and select an alternative drug instead of flucloxacillin if liver enzymes or bilirubin is elevated, for patients that are HLA-B*57:01. | DPWG | Annotation_of_DPWG_Guideline_for_flucloxacillin_and_HLA_B.json |
| 317 | flecainide | CYP2D6 | Reduce flecainide dose by 50% for CYP2D6 poor metabolizer (PM) and record an ECG and monitor the plasma concentration. Reduce flecainide dose to 75% of the standard dose for CYP2D6 intermediate metabolizer (IM) patients with indications other than diagnosis of Brugada syndrome and record an ECG and monitor the plasma concentration. | DPWG | Annotation_of_DPWG_Guideline_for_flecainide_and_CYP2D6.json |
| 318 | flucytosine | DPYD | The Dutch Pharmacogenetics Working Group Guideline for flucytosine states that patients with a DPYD activity score of 0 should avoid flucytosine. Patients with a DPYD activity score of 1 or 1.5 should be alert to the occurrence of severe side effects and flucytosine should be stopped if severe side effects occur. | DPWG | Annotation_of_DPWG_Guideline_for_flucytosine_and_DPYD.json |
| 319 | fluorouracil | DPYD | An alternative drug to fluorouracil is recommended for patients with a DPYD activity score of 0; if an alternative drug is not possible, the DPD activity should be determined and the initial dose adjusted accordingly. For patients with a DPYD activity score of 1 or 1.5, start with 50% of the standard dose or choose an alternative drug; adjustment of subsequent doses should be guided by toxicity and effectiveness. Patients with two partially functional alleles or one non-functional and one partially functional allele or two gene variants leading to partially functional alleles or a gene variant leading to a non-functional allele and a gene variant leading to a partially functional allele should have their DPD activity determined or should avoid fluorouracil/capecitabine. Tegafur is not an alternative for fluorouracil, as it is also metabolized by DPD. The DPWG evaluated DPYD genotyping as "essential" and recommend DPYD testing prior to initiating fluoropyrimidines. | DPWG | Annotation_of_DPWG_Guideline_for_fluorouracil_and_DPYD.json |
| 320 | flupenthixol | CYP2D6 | There are currently no dosing recommendations for flupenthixol based on CYP2D6 genotype. | DPWG | Annotation_of_DPWG_Guideline_for_flupenthixol_and_CYP2D6.json |
| 321 | fluoxetine | CYP2D6 | There are currently no dosing recommendations for fluoxetine based on CYP2D6 genotype. | DPWG | Annotation_of_DPWG_Guideline_for_fluoxetine_and_CYP2D6.json |
| 322 | fluvoxamine | CYP2C19 | There are currently no dosing recommendations for fluvoxamine based on CYP2C19 genotypes. | DPWG | Annotation_of_DPWG_Guideline_for_fluvoxamine_and_CYP2C19.json |
| 323 | fluvastatin | SLCO1B1 | There are currently no recommendations for fluvastatin dosing based on SLCO1B1 genotypes. | DPWG | Annotation_of_DPWG_Guideline_for_fluvastatin_and_SLCO1B1.json |
| 324 | fluvoxamine | CYP2D6 | There are currently no dosing recommendations for fluvoxamine based on CYP2D6 genotype. | DPWG | Annotation_of_DPWG_Guideline_for_fluvoxamine_and_CYP2D6.json |
| 325 | folic acid | MTHFR | There are currently no recommendations for folic acid dosing based on MTHFR rs1801133 genotypes. | DPWG | Annotation_of_DPWG_Guideline_for_folic_acid_and_MTHFR.json |
| 326 | gefitinib | CYP2D6 | There are currently no dosing recommendations for gefitinib based on CYP2D6 genotype. | DPWG | Annotation_of_DPWG_Guideline_for_gefitinib_and_CYP2D6.json |
| 327 | glimepiride | CYP2C9 | There are currently no dosing recommendations for glimepiride based on CYP2C9 genotype. | DPWG | Annotation_of_DPWG_Guideline_for_glimepiride_and_CYP2C9.json |
| 328 | gliclazide | CYP2C9 | There are currently no dosing recommendations for gliclazide based on CYP2C9 genotype. | DPWG | Annotation_of_DPWG_Guideline_for_gliclazide_and_CYP2C9.json |
| 329 | glyburide | CYP2C9 | There are currently no dosing recommendations for glibenclamide based on CYP2C9 genotype. | DPWG | Annotation_of_DPWG_Guideline_for_glyburide_and_CYP2C9.json |
| 330 | haloperidol | CYP2D6 | The recommendation for CYP2D6 poor metabolizers is to use 60% of the normal dose of haloperidol and for CYP2D6 ultrarapid metabolizers to use 1.5 times the normal dose or to choose an alternative to haloperidol. | DPWG | Annotation_of_DPWG_Guideline_for_haloperidol_and_CYP2D6.json |
| 331 | imipramine | CYP2C19 | CYP2C19 poor metabolizers should receive 70% of the standard dose of imipramine, or imipramine should be avoided in these patients. Patients should be monitored for the effect and side effects or the plasma concentrations of imipramine and desipramine in order to set the maintenance dose. | DPWG | Annotation_of_DPWG_Guideline_for_imipramine_and_CYP2C19.json |
| 332 | imipramine | CYP2D6 | CYP2D6 poor metabolizers should receive 30% of the standard dose of imipramine, CYP2D6 intermediate metabolizers should receive 70% of the standard dose, and CYP2D6 ultra-rapid metabolizers should receive 1.7 times the standard dose. Patients should be monitored for the effect and side effects or the plasma concentrations of imipramine and desipramine in order to set the maintenance dose. | DPWG | Annotation_of_DPWG_Guideline_for_imipramine_and_CYP2D6.json |
| 333 | irinotecan | UGT1A1 | Dose reductions are recommended for irinotecan for patients who are UGT1A1 *28/*28 or UGT1A1 PM, starting with 70% of starting dose and increasing as tolerated, guided by neutrophil count. | DPWG | Annotation_of_DPWG_Guideline_for_irinotecan_and_UGT1A1.json |
| 334 | lamotrigine | HLA-B | Avoid lamotrigine in patients with HLA-B*15:02 if an alternative is available. If avoidance is not possible, advise to report any rash immediately. | DPWG | Annotation_of_DPWG_Guideline_for_lamotrigine_and_HLA_B.json |
| 335 | lansoprazole | CYP2C19 | For CYP2C19 ultrarapid metabolizers who are undergoing H. pylori eradication therapy, use a 4-fold higher dose. For CYP2C19 ultrarapid metabolizers with other indications, be alert to reduced effectiveness and, if necessary, use a 4-fold higher dose. | DPWG | Annotation_of_DPWG_Guideline_for_lansoprazole_and_CYP2C19.json |
| 336 | mercaptopurine | NUDT15 | Select an alternative drug or reduce the initial dose of mercaptopurine for patients that are NUDT15 poor metabolizers and reduce initial dose for patients that are NUDT15 intermediate metabolizers. | DPWG | Annotation_of_DPWG_Guideline_for_mercaptopurine_and_NUDT15.json |
| 337 | mercaptopurine | TPMT | Select an alternative drug or reduce the initial dose of mercaptopurine for patients that are TPMT poor metabolizers and reduce initial dose for patients that are TPMT intermediate metabolizers. | DPWG | Annotation_of_DPWG_Guideline_for_mercaptopurine_and_TPMT.json |
| 338 | methotrexate | MTHFR | There are currently no recommendations for methotrexate dosing based on MTHFR rs1801133 genotypes. | DPWG | Annotation_of_DPWG_Guideline_for_methotrexate_and_MTHFR.json |
| 339 | methylphenidate | COMT | There are currently no dosing recommendations for methylphenidate based on COMT genotype. | DPWG | Annotation_of_DPWG_Guideline_for_methylphenidate_and_COMT.json |
| 340 | methylphenidate | CYP2D6 | There are currently no dosing recommendations for methylphenidate based on CYP2D6 genotype. | DPWG | Annotation_of_DPWG_Guideline_for_methylphenidate_and_CYP2D6.json |
| 341 | mirtazapine | CYP2C19 | There are currently no dosing recommendations for mirtazapine based on CYP2C19 genotypes. | DPWG | Annotation_of_DPWG_Guideline_for_mirtazapine_and_CYP2C19.json |
| 342 | metoprolol | CYP2D6 | For CYP2D6 poor and intermediate metabolizer patients, if a GRADUAL REDUCTION in HEART RATE is desired, or in the event of SYMPTOMATIC BRADYCARDIA, use smaller steps in dose titration and/or prescribe no more than 25% or 50% of the standard dose, respectively. For CYP2D6 ultra metabolizers, use the maximum dose for the relevant indication as a target dose, and if the effectiveness is still insufficient: increase the dose based on effectiveness and side effects to 2.5 times the standard dose or select an alternative drug. | DPWG | Annotation_of_DPWG_Guideline_for_metoprolol_and_CYP2D6.json |
| 343 | mirtazapine | CYP2D6 | There are currently no dosing recommendations for mirtazapine based on CYP2D6 genotype. | DPWG | Annotation_of_DPWG_Guideline_for_mirtazapine_and_CYP2D6.json |
| 344 | moclobemide | CYP2C19 | No action is needed for this gene-drug interaction. | DPWG | Annotation_of_DPWG_Guideline_for_moclobemide_and_CYP2C19.json |
| 345 | nortriptyline | CYP2D6 | The Dutch Pharmacogenetics Working Group Guideline for nortriptyline recommends a dose reduction for CYP2D6 poor or intermediate metabolizer patients. For CYP2D6 ultrarapid metabolizers, select an alternative drug or use 1.7 times the standard dose. Monitoring of nortriptyline and 10-hydroxynortriptyline plasma concentrations is recommended. | DPWG | Annotation_of_DPWG_Guideline_for_nortriptyline_and_CYP2D6.json |
| 346 | olanzapine | CYP1A2 | There are currently no recommendations for olanzapine dosing based on CYP1A2 genotypes. | DPWG | Annotation_of_DPWG_Guideline_for_olanzapine_and_CYP1A2.json |
| 347 | olanzapine | CYP2D6 | There are currently no dosing recommendations for olanzapine based on CYP2D6 genotype. | DPWG | Annotation_of_DPWG_Guideline_for_olanzapine_and_CYP2D6.json |
| 348 | omeprazole | CYP2C19 | For CYP2C19 ultrarapid metabolizers who are undergoing H. pylori eradication therapy, use a 3-fold higher dose. For CYP2C19 ultrarapid metabolizers with other indications, be alert to reduced effectiveness and, if necessary, use a 3-fold higher dose. | DPWG | Annotation_of_DPWG_Guideline_for_omeprazole_and_CYP2C19.json |
| 349 | oxcarbazepine | HLA-B | Oxcarbazepine should be avoided if an alternative is available in patients with the HLA-B*15:02 allele. | DPWG | Annotation_of_DPWG_Guideline_for_oxcarbazepine_and_HLA_B.json |
| 350 | oxycodone | CYP2D6 | There are currently no dosing recommendations for oxycodone based on CYP2D6 genotypes. | DPWG | Annotation_of_DPWG_Guideline_for_oxycodone_and_CYP2D6.json |
| 351 | paclitaxel | CYP3A4 | There are currently no recommendations for paclitaxel dosing based on CYP3A4 genotypes. | DPWG | Annotation_of_DPWG_Guideline_for_paclitaxel_and_CYP3A4.json |
| 352 | pantoprazole | CYP2C19 | For CYP2C19 ultrarapid metabolizers who are undergoing H. pylori eradication therapy, use a 5-fold higher dose. For CYP2C19 ultrarapid metabolizers with other indications, be alert to reduced effectiveness and, if necessary, use a 5-fold higher dose. | DPWG | Annotation_of_DPWG_Guideline_for_pantoprazole_and_CYP2C19.json |
| 353 | paroxetine | CYP2D6 | Select an alternative drug rather than paroxetine for CYP2D6 ultrarapid metabolizer patients. | DPWG | Annotation_of_DPWG_Guideline_for_paroxetine_and_CYP2D6.json |
| 354 | phenprocoumon | CYP2C9 | There are currently no recommendations for phenprocoumon dosing based on CYP2C9 genotypes. | DPWG | Annotation_of_DPWG_Guideline_for_phenprocoumon_and_CYP2C9.json |
| 355 | phenprocoumon | VKORC1 | Patients with the VKORC1 rs9923231 TT genotype (-1639 AA genotype) is recommended to be given 50% of the standard initial dose of phenprocoumon and more frequent monitoring of INR. The genotype-specific initial dose and maintenance dose can be calculated using an algorithm. There is no recommendation for patients with the VKORC1 rs9923231 CT genotype (-1639 AG genotype). | DPWG | Annotation_of_DPWG_Guideline_for_phenprocoumon_and_VKORC1.json |
| 356 | phenytoin | CYP2C9 | Use the standard starting dose of phenytoin and reduce the maintenance dose based on CYP2C9 genotype; monitor response and serum concentrations and be aware of ADEs. | DPWG | Annotation_of_DPWG_Guideline_for_phenytoin_and_CYP2C9.json |
| 357 | phenytoin | HLA-B | Phenytoin should be avoided if possible in patients with the HLA-B*15:02 allele. | DPWG | Annotation_of_DPWG_Guideline_for_phenytoin_and_HLA_B.json |
| 358 | pimozide | CYP2D6 | Patients who are CYP2D6 intermediate metabolizers should be given no more than 80% of the standard maximum dose of pimozide while patients who are CYP2D6 poor metabolizers should be given no more than 50% of the standard maximum dose. | DPWG | Annotation_of_DPWG_Guideline_for_pimozide_and_CYP2D6.json |
| 359 | prasugrel | CYP2C19 | There are currently no dosing recommendations for prasugrel based on CYP2C19 genotypes. | DPWG | Annotation_of_DPWG_Guideline_for_prasugrel_and_CYP2C19.json |
| 360 | propafenone | CYP2D6 | Reduce the dose of propafenone by 70% for CYP2D6 poor metabolizers, and monitor propafenone plasma concentrations or use an alternative drug for CYP2D6 intermediate and ultrarapid metabolizers. | DPWG | Annotation_of_DPWG_Guideline_for_propafenone_and_CYP2D6.json |
| 361 | quetiapine | CYP2D6 | There are currently no dosing recommendations for quetiapine based on CYP2D6 genotype. | DPWG | Annotation_of_DPWG_Guideline_for_quetiapine_and_CYP2D6.json |
| 362 | quetiapine | CYP3A4 | Patients who are CYP3A4 poor metabolizers and being treated for depression should be given an alternative drug. Patients being treated for other indications and who are CYP3A4 poor metabolizers should be given a reduced dose of quetiapine. No action is needed for patients who are CYP3A4 intermediate metabolizers. PharmGKB uses CYP3A4 allele nomenclature as defined by PharmVar, while the current version of this guideline issued by the DPWG uses retired allele names. Further details are given on the DPWG curation page. | DPWG | Annotation_of_DPWG_Guideline_for_quetiapine_and_CYP3A4.json |
| 363 | quinidine | CYP2D6 | There are currently no dosing recommendations for quinidine based on CYP2D6 genotypes. | DPWG | Annotation_of_DPWG_Guideline_for_quinidine_and_CYP2D6.json |
| 364 | rabeprazole | CYP2C19 | No action is needed for this gene-drug interaction. | DPWG | Annotation_of_DPWG_Guideline_for_rabeprazole_and_CYP2C19.json |
| 365 | ribavirin | HLA-B | There are no dosing recommendations for ribavirin in HLA-B*44 positive patients at this time. | DPWG | Annotation_of_DPWG_Guideline_for_ribavirin_and_HLA_B.json |
| 366 | risperidone | CYP2D6 | The Dutch Pharmacogenetics Working Group Guideline for risperidone recommends decreasing the dose for CYP2D6 poor metabolizers and using an alternative drug or titrate the dose according to the maximum dose for the active metabolite for CYP2D6 ultrarapid metabolizers. | DPWG | Annotation_of_DPWG_Guideline_for_risperidone_and_CYP2D6.json |
| 367 | sertraline | CYP2C19 | Do not give doses exceeding 75 mg/day in patients with CYP2C19 poor metabolizer genotypes, and guide the dose by response and side effects and/or sertraline plasma concentration. | DPWG | Annotation_of_DPWG_Guideline_for_sertraline_and_CYP2C19.json |
| 368 | sertraline | CYP2D6 | There are currently no dosing recommendations for sertraline based on CYP2D6 genotypes. | DPWG | Annotation_of_DPWG_Guideline_for_sertraline_and_CYP2D6.json |
| 369 | simvastatin | SLCO1B1 | Choose an alternative for patients with the SLCO1B1 521 CC or TC (rs4149056) genotype and consider any additional risk factors for statin-induced myopathy. If an alternative is not an option for patients with the 521 TC genotype, avoid simvastatin doses exceeding 40 mg/day and advise the patient to contact their doctor in the event of muscle symptoms. | DPWG | Annotation_of_DPWG_Guideline_for_simvastatin_and_SLCO1B1.json |
| 370 | siponimod | CYP2C9 | The Dutch Pharmacogenetics Working Group Guideline for siponimod recommends decreasing the dose for CYP2C9 *1/*3, *2/*3 genotypes and to avoid siponimod for the CYP2C9 *3/*3 genotype. | DPWG | Annotation_of_DPWG_Guideline_for_siponimod_and_CYP2C9.json |
| 371 | tacrolimus | CYP3A5 | Increased doses of tacrolimus are recommended for CYP3A5 heterozygous and homozygous expressors and adjusted as needed. Instructions for liver transplantation patients include genotypes of both patient and donor. | DPWG | Annotation_of_DPWG_Guideline_for_tacrolimus_and_CYP3A5.json |
| 372 | sotalol | CYP2D6 | There are currently no dosing recommendations for sotalol based on CYP2D6 genotype. | DPWG | Annotation_of_DPWG_Guideline_for_sotalol_and_CYP2D6.json |
| 373 | tamoxifen | CYP2D6 | For CYP2D6 poor and intermediate metabolizers, consider an alternative medication or a dose increase. For intermediate metabolizers, avoid concomitant CYP2D6 inhibitor use. | DPWG | Annotation_of_DPWG_Guideline_for_tamoxifen_and_CYP2D6.json |
| 374 | tegafur | DPYD | Choose an alternative drug to tegafur for patients with DPYD Activity Score of 0. For patients with a DPYD Activity Score of 1 or 1.5, avoid tegafur or start with a low dose and adjust the initial dose based on toxicity and efficacy. Fluorouracil and capecitabine are not alternatives for tegafur, as these are also metabolized by DPD. The DPWG evaluated DPYD genotyping as "essential" and recommend DPYD testing prior to initiating fluoropyrimidines. | DPWG | Annotation_of_DPWG_Guideline_for_tegafur_and_DPYD.json |
| 375 | thioguanine | TPMT | Select an alternative drug or reduce the initial dose of thioguanine for patients that are TPMT poor metabolizers and reduce initial dose for patients that are TPMT intermediate metabolizers. | DPWG | Annotation_of_DPWG_Guideline_for_thioguanine_and_TPMT.json |
| 376 | thioguanine | NUDT15 | Select an alternative drug or reduce the initial dose of thioguanine for patients that are NUDT15 poor metabolizers and reduce initial dose for patients that are NUDT15 intermediate metabolizers. | DPWG | Annotation_of_DPWG_Guideline_for_thioguanine_and_NUDT15.json |
| 377 | ticagrelor | CYP2C19 | There are currently no dosing recommendations for ticagrelor based on CYP2C19 genotypes. | DPWG | Annotation_of_DPWG_Guideline_for_ticagrelor_and_CYP2C19.json |
| 378 | tolbutamide | CYP2C9 | There are currently no dosing recommendations for tolbutamide based on CYP2C9 genotype. | DPWG | Annotation_of_DPWG_Guideline_for_tolbutamide_and_CYP2C9.json |
| 379 | tramadol | CYP2D6 | Be alert to a reduced efficacy of tramadol in CYP2D6 intermediate or poor metabolizers. If tramadol is not effective in these patients, try a dose increase or select an alternative to tramadol (not codeine) and be alert for symptoms of insufficient pain relief. For CYP2D6 ultrarapid metabolizers, use an alternative to tramadol (not codeine) or use 40% of the standard dose and be alert to side effects. | DPWG | Annotation_of_DPWG_Guideline_for_tramadol_and_CYP2D6.json |
| 380 | venlafaxine | CYP2D6 | For CYP2D6 poor (PM) and intermediate metabolizers (IM), select an alternative to venlafaxine or reduce the dose and monitor patient's plasma metabolite level. For CYP2D6 ultrarapid metabolizers (UM), therapy adjustment is not required or beneficial for this phenotype-drug combination. | DPWG | Annotation_of_DPWG_Guideline_for_venlafaxine_and_CYP2D6.json |
| 381 | voriconazole | CYP2C19 | Patients who are CYP2C19 poor metabolizers should receive 50% of the standard dose, and CYP2C19 ultrarapid metabolizers should receive a 1.5 times higher initial dose. Monitor voriconazole plasma concentrations for CYP2C19 poor, intermediate and ultrarapid metabolizers. | DPWG | Annotation_of_DPWG_Guideline_for_voriconazole_and_CYP2C19.json |
| 382 | warfarin | CYP2C9 | Reduce warfarin dose in CYP2C9 poor and intermediate metabolizers (PM and IM) and patients with CYP2C9*1/*3, *2/*3, *2/*2 or *3/*3 genotype. The genotype-specific initial dose and maintenance dose can be calculated using an algorithm, as used in EU-PACT. | DPWG | Annotation_of_DPWG_Guideline_for_warfarin_and_CYP2C9.json |
| 383 | warfarin | VKORC1 | Patients with the VKORC1 rs9923231 TT genotype (-1639 AA genotype) should be given 60% of the standard initial dose of warfarin. The genotype-specific initial dose and maintenance dose can be calculated using an algorithm. There are no recommendation for patients with the VKORC1 rs9923231 CT genotype (-1639 AG genotype). | DPWG | Annotation_of_DPWG_Guideline_for_warfarin_and_VKORC1.json |
| 384 | zuclopenthixol | CYP2D6 | For CYP2D6 poor and intermediate metabolizers, reduce zuclopenthixol dose. For ultrarapid metabolizers, if the effectiveness is insufficient a dose increase not exceeding 1.5x normal dose is suggested. | DPWG | Annotation_of_DPWG_Guideline_for_zuclopenthixol_and_CYP2D6.json |
| 385 | abacavir | HLA-B | In individuals with the HLA-B*57:01 variant allele ("HLA-B*57:01-positive"), abacavir is not recommended and should be considered only under exceptional circumstances. See full guideline for disclaimers, further details and supporting evidence. | CPIC | Annotation_of_CPIC_Guideline_for_abacavir_and_HLA_B.json |

1. Related Chemicals Name: Associated drug names linked to gene-drug interactions.
2. Related Genes Symbol: Genes affecting drug metabolism or efficacy.
3. Summary Markdown: Summary of pharmacogenomic guidelines.
4. File: Source or reference files for guidelines. The supplementary material is provided as a JSON file containing detailed clinical guidelines. Data preprocessing was conducted using R and Excel. From the JSON file, the **Chemical Names** under the "Related" field were extracted as specific genes, the **Gene Symbols** under the "Related" field were extracted as the corresponding drugs, and the **Summary Markdown** served as the source for gene-drug interaction recommendations to facilitate further analysis. Pharmacists thoroughly reviewed the guidelines and compiled a comprehensive list of drug-gene interaction pairs. These included recommendations sourced from clinical guideline annotations available in PharmGKB, such as specific genes, corresponding drugs, gene-associated phenotypes, and details of gene-drug interaction

**Table S2. Prediction Stability of GPT-4o Across 20 Iterations**

| **No.** | **Base value ^a^** | **Individual results of 20 predictions by GPT-4o ^b^** | | | | | | | | | | | | | | | | | | | |
| --- | --- | --- | --- | --- | --- | --- | --- | --- | --- | --- | --- | --- | --- | --- | --- | --- | --- | --- | --- | --- | --- |
|  |  | **1** | **2** | **3** | **4** | **5** | **6** | **7** | **8** | **9** | **10** | **11** | **12** | **13** | **14** | **15** | **16** | **17** | **18** | **19** | **20** |
| 1 | No action needed | 1 | 1 | 1 | 1 | 1 | 1 | 1 | 1 | 1 | 1 | 1 | 1 | 1 | 1 | 1 | 1 | 1 | 1 | 1 | 1 |
| 2 | No action needed | 1 | 1 | 1 | 1 | 1 | 1 | 1 | 1 | 1 | 1 | 1 | 1 | 1 | 1 | 1 | 1 | 1 | 1 | 1 | 1 |
| 3 | No action needed | 1 | 1 | 1 | 1 | 1 | 1 | 1 | 1 | 1 | 1 | 1 | 1 | 1 | 1 | 1 | 1 | 1 | 1 | 1 | 1 |
| 4 | No action needed | 1 | 1 | 1 | 1 | 1 | 1 | 1 | 1 | 1 | 1 | 1 | 1 | 1 | 1 | 1 | 1 | 1 | 1 | 1 | 1 |
| 5 | No action needed | 1 | 1 | 1 | 1 | 1 | 1 | 1 | 1 | 1 | 1 | 1 | 1 | 1 | 1 | 1 | 1 | 1 | 1 | 1 | 1 |
| 6 | No action needed | 1 | 1 | 1 | 1 | 1 | 1 | 1 | 1 | 1 | 1 | 1 | 1 | 1 | 1 | 1 | 1 | 1 | 1 | 1 | 1 |
| 7 | No action needed | 1 | 1 | 1 | 1 | 1 | 1 | 1 | 1 | 1 | 1 | 1 | 1 | 1 | 1 | 1 | 1 | 1 | 1 | 1 | 1 |
| 8 | No action needed | 1 | 1 | 1 | 1 | 1 | 1 | 1 | 1 | 1 | 1 | 1 | 1 | 1 | 1 | 1 | 1 | 1 | 1 | 1 | 1 |
| 9 | No action needed | 1 | 1 | 1 | 1 | 1 | 1 | 1 | 1 | 1 | 1 | 1 | 1 | 1 | 1 | 1 | 1 | 1 | 1 | 1 | 1 |
| 10 | No action needed | 1 | 1 | 1 | 1 | 1 | 1 | 1 | 1 | 1 | 1 | 1 | 1 | 1 | 1 | 1 | 1 | 1 | 1 | 1 | 1 |
| 11 | No action needed | 1 | 1 | 1 | 1 | 1 | 1 | 1 | 1 | 1 | 1 | 1 | 1 | 1 | 1 | 1 | 1 | 1 | 1 | 1 | 1 |
| 12 | No action needed | 1 | 1 | 1 | 1 | 1 | 1 | 1 | 1 | 1 | 1 | 1 | 1 | 1 | 1 | 1 | 1 | 1 | 1 | 1 | 1 |
| 13 | No action needed | 1 | 1 | 1 | 1 | 1 | 1 | 1 | 1 | 1 | 1 | 1 | 1 | 1 | 1 | 1 | 1 | 1 | 1 | 1 | 1 |
| 14 | No action needed | 1 | 1 | 1 | 1 | 1 | 1 | 1 | 1 | 1 | 1 | 1 | 1 | 1 | 1 | 1 | 1 | 1 | 1 | 1 | 1 |
| 15 | No action needed | 1 | 1 | 1 | 1 | 1 | 1 | 1 | 1 | 1 | 1 | 1 | 1 | 1 | 1 | 1 | 1 | 1 | 1 | 1 | 1 |
| 16 | No action needed | 1 | 1 | 1 | 1 | 1 | 1 | 1 | 1 | 1 | 1 | 1 | 1 | 1 | 1 | 1 | 1 | 1 | 1 | 1 | 1 |
| 17 | No action needed | 1 | 1 | 1 | 1 | 1 | 1 | 1 | 1 | 1 | 1 | 1 | 1 | 1 | 1 | 1 | 1 | 1 | 1 | 1 | 1 |
| 18 | No action needed. | 1 | 1 | 1 | 1 | 1 | 1 | 1 | 1 | 1 | 1 | 1 | 1 | 1 | 1 | 1 | 1 | 1 | 1 | 1 | 1 |
| 19 | No action needed | 1 | 1 | 1 | 1 | 1 | 1 | 1 | 1 | 1 | 1 | 1 | 1 | 1 | 1 | 1 | 1 | 1 | 1 | 1 | 1 |
| 20 | No action needed | 1 | 1 | 1 | 1 | 1 | 1 | 1 | 1 | 1 | 1 | 1 | 1 | 1 | 1 | 1 | 1 | 1 | 1 | 1 | 1 |
| 21 | No action needed | 1 | 1 | 1 | 1 | 1 | 1 | 1 | 1 | 1 | 1 | 1 | 1 | 1 | 1 | 1 | 1 | 1 | 1 | 1 | 1 |
| 22 | No action needed. | 1 | 1 | 1 | 1 | 1 | 1 | 1 | 1 | 1 | 1 | 1 | 1 | 1 | 1 | 1 | 1 | 1 | 1 | 1 | 1 |
| 23 | No action needed | 1 | 1 | 1 | 1 | 1 | 1 | 1 | 1 | 1 | 1 | 1 | 1 | 1 | 1 | 1 | 1 | 1 | 1 | 1 | 1 |
| 24 | No action needed | 1 | 1 | 1 | 1 | 1 | 1 | 1 | 1 | 1 | 1 | 1 | 1 | 1 | 1 | 1 | 1 | 1 | 1 | 1 | 1 |
| 25 | Change medication | 1 | 1 | 1 | 1 | 1 | 1 | 1 | 1 | 1 | 1 | 1 | 1 | 1 | 1 | 1 | 1 | 1 | 1 | 1 | 1 |
| 26 | Consider dosage modification | 1 | 1 | 1 | 0 | 1 | 0 | 1 | 1 | 1 | 1 | 1 | 1 | 0 | 1 | 1 | 1 | 0 | 1 | 0 | 1 |
| 27 | Consider dosage modification | 0 | 0 | 1 | 1 | 1 | 1 | 1 | 1 | 0 | 1 | 1 | 1 | 1 | 1 | 1 | 1 | 0 | 1 | 1 | 1 |
| 28 | Change medication | 1 | 1 | 1 | 1 | 1 | 1 | 1 | 1 | 1 | 1 | 1 | 1 | 1 | 1 | 1 | 1 | 1 | 1 | 1 | 1 |
| 29 | Consider dosage modification | 1 | 1 | 1 | 1 | 1 | 1 | 1 | 1 | 1 | 1 | 1 | 1 | 1 | 1 | 1 | 1 | 1 | 1 | 1 | 1 |
| 30 | Consider dosage modification | 1 | 1 | 1 | 1 | 1 | 1 | 1 | 1 | 1 | 1 | 1 | 1 | 1 | 1 | 1 | 1 | 1 | 1 | 1 | 1 |
| 31 | Change medication | 0 | 1 | 1 | 1 | 0 | 1 | 1 | 1 | 1 | 1 | 1 | 1 | 1 | 1 | 1 | 1 | 1 | 1 | 1 | 1 |
| 32 | Change medication | 1 | 1 | 1 | 1 | 1 | 1 | 1 | 1 | 1 | 1 | 1 | 1 | 1 | 1 | 1 | 1 | 1 | 1 | 1 | 1 |
| 33 | Change medication | 1 | 1 | 1 | 1 | 1 | 1 | 1 | 1 | 1 | 1 | 1 | 1 | 1 | 1 | 1 | 1 | 1 | 1 | 1 | 1 |
| 34 | Change medication | 1 | 1 | 1 | 1 | 1 | 1 | 1 | 1 | 1 | 1 | 1 | 1 | 1 | 1 | 1 | 1 | 1 | 1 | 1 | 1 |
| 35 | Change medication | 1 | 1 | 1 | 1 | 1 | 1 | 1 | 1 | 1 | 1 | 1 | 1 | 1 | 1 | 1 | 1 | 1 | 1 | 1 | 1 |
| 36 | Change medication | 1 | 1 | 1 | 1 | 1 | 1 | 1 | 1 | 1 | 1 | 1 | 1 | 1 | 1 | 1 | 1 | 1 | 1 | 1 | 1 |
| 37 | Change medication | 1 | 1 | 1 | 1 | 1 | 1 | 1 | 1 | 1 | 1 | 1 | 1 | 1 | 1 | 1 | 1 | 1 | 1 | 1 | 1 |
| 38 | Change medication | 1 | 1 | 1 | 1 | 1 | 1 | 1 | 1 | 1 | 1 | 1 | 1 | 1 | 1 | 1 | 1 | 1 | 1 | 1 | 1 |
| 39 | Change medication | 1 | 1 | 1 | 1 | 1 | 1 | 1 | 1 | 1 | 1 | 1 | 1 | 1 | 1 | 1 | 1 | 1 | 1 | 1 | 1 |
| 40 | Change medication | 1 | 1 | 1 | 1 | 1 | 1 | 1 | 1 | 1 | 1 | 1 | 1 | 1 | 1 | 1 | 1 | 1 | 1 | 1 | 1 |
| 41 | Change medication | 1 | 1 | 1 | 1 | 1 | 1 | 1 | 1 | 1 | 1 | 1 | 1 | 1 | 1 | 1 | 1 | 1 | 1 | 1 | 1 |
| 42 | Change medication | 1 | 1 | 1 | 1 | 1 | 1 | 1 | 1 | 1 | 1 | 1 | 1 | 1 | 1 | 1 | 1 | 1 | 1 | 1 | 1 |
| 43 | Consider dosage modification | 1 | 1 | 1 | 1 | 1 | 1 | 1 | 1 | 1 | 1 | 1 | 1 | 1 | 1 | 1 | 1 | 1 | 1 | 1 | 1 |
| 44 | Consider dosage modification | 1 | 1 | 1 | 1 | 1 | 1 | 1 | 1 | 1 | 1 | 1 | 1 | 1 | 1 | 1 | 1 | 1 | 1 | 1 | 1 |
| 45 | No action needed | 1 | 1 | 1 | 1 | 1 | 1 | 1 | 1 | 1 | 1 | 1 | 1 | 1 | 1 | 1 | 1 | 1 | 1 | 1 | 1 |
| 46 | No action needed | 1 | 1 | 1 | 1 | 1 | 1 | 1 | 1 | 1 | 1 | 1 | 1 | 1 | 1 | 1 | 1 | 1 | 1 | 1 | 1 |
| 47 | No action needed | 1 | 1 | 1 | 1 | 1 | 1 | 1 | 1 | 1 | 1 | 1 | 1 | 1 | 1 | 1 | 1 | 1 | 1 | 1 | 1 |
| 48 | No action needed | 1 | 1 | 1 | 1 | 1 | 1 | 1 | 1 | 1 | 1 | 1 | 1 | 1 | 1 | 1 | 1 | 1 | 1 | 1 | 1 |
| 49 | No action needed | 1 | 1 | 1 | 1 | 1 | 1 | 1 | 1 | 1 | 1 | 1 | 1 | 1 | 1 | 1 | 1 | 1 | 1 | 1 | 1 |
| 50 | No action needed | 1 | 1 | 1 | 1 | 1 | 1 | 1 | 1 | 1 | 1 | 1 | 1 | 1 | 1 | 1 | 1 | 1 | 1 | 1 | 1 |
| 51 | No action needed. | 1 | 1 | 1 | 1 | 1 | 1 | 1 | 1 | 1 | 1 | 1 | 1 | 1 | 1 | 1 | 1 | 1 | 1 | 1 | 1 |
| 52 | No action needed | 1 | 1 | 1 | 1 | 1 | 1 | 1 | 1 | 1 | 1 | 1 | 1 | 1 | 1 | 1 | 1 | 1 | 1 | 1 | 1 |
| 53 | No action needed | 1 | 1 | 1 | 1 | 1 | 1 | 1 | 1 | 1 | 1 | 1 | 1 | 1 | 1 | 1 | 1 | 1 | 1 | 1 | 1 |
| 54 | No action needed | 1 | 1 | 1 | 1 | 1 | 1 | 1 | 1 | 1 | 1 | 1 | 1 | 1 | 1 | 1 | 1 | 1 | 1 | 1 | 1 |
| 55 | No action needed | 1 | 1 | 1 | 1 | 1 | 1 | 1 | 1 | 1 | 1 | 1 | 1 | 1 | 1 | 1 | 1 | 1 | 1 | 1 | 1 |
| 56 | No action needed | 1 | 1 | 1 | 1 | 1 | 1 | 1 | 1 | 1 | 1 | 1 | 1 | 1 | 1 | 1 | 1 | 1 | 1 | 1 | 1 |
| 57 | No action needed | 1 | 1 | 1 | 1 | 1 | 1 | 1 | 1 | 1 | 1 | 1 | 1 | 1 | 1 | 1 | 1 | 1 | 1 | 1 | 1 |
| 58 | No action needed | 1 | 1 | 1 | 1 | 1 | 1 | 1 | 1 | 1 | 1 | 1 | 1 | 1 | 1 | 1 | 1 | 1 | 1 | 1 | 1 |
| 59 | No action needed | 1 | 1 | 1 | 1 | 1 | 1 | 1 | 1 | 1 | 1 | 1 | 1 | 1 | 1 | 1 | 1 | 1 | 1 | 1 | 1 |
| 60 | No action needed | 1 | 1 | 1 | 1 | 1 | 1 | 1 | 1 | 1 | 1 | 1 | 1 | 1 | 1 | 1 | 1 | 1 | 1 | 1 | 1 |
| 61 | No action needed | 1 | 1 | 1 | 1 | 1 | 1 | 1 | 1 | 1 | 1 | 1 | 1 | 1 | 1 | 1 | 0 | 1 | 1 | 1 | 1 |
| 62 | No action needed | 1 | 1 | 1 | 1 | 1 | 1 | 1 | 1 | 1 | 1 | 1 | 1 | 1 | 1 | 1 | 1 | 1 | 1 | 1 | 1 |
| 63 | No action needed | 1 | 1 | 1 | 1 | 1 | 1 | 1 | 1 | 1 | 1 | 1 | 1 | 1 | 1 | 1 | 1 | 1 | 1 | 1 | 1 |
| 64 | No action needed | 1 | 1 | 1 | 1 | 1 | 1 | 1 | 1 | 1 | 1 | 1 | 1 | 1 | 1 | 1 | 1 | 1 | 1 | 1 | 1 |
| 65 | No action needed | 1 | 1 | 1 | 1 | 1 | 1 | 1 | 1 | 1 | 1 | 1 | 1 | 1 | 1 | 1 | 1 | 1 | 1 | 1 | 1 |
| 66 | No action needed | 1 | 1 | 1 | 1 | 1 | 1 | 1 | 1 | 1 | 1 | 1 | 1 | 1 | 1 | 1 | 1 | 1 | 1 | 1 | 1 |
| 67 | No action needed | 1 | 1 | 1 | 1 | 1 | 1 | 1 | 1 | 1 | 1 | 1 | 1 | 1 | 1 | 1 | 1 | 1 | 1 | 1 | 1 |
| 68 | No action needed | 1 | 1 | 1 | 1 | 1 | 1 | 1 | 1 | 1 | 1 | 1 | 1 | 1 | 1 | 1 | 1 | 1 | 1 | 1 | 1 |
| 69 | No action needed | 1 | 1 | 1 | 1 | 1 | 1 | 1 | 1 | 1 | 1 | 1 | 1 | 1 | 1 | 1 | 1 | 1 | 1 | 1 | 1 |
| 70 | No action needed | 1 | 1 | 1 | 1 | 1 | 1 | 1 | 1 | 1 | 1 | 1 | 1 | 1 | 1 | 1 | 1 | 1 | 1 | 1 | 1 |
| 71 | No action needed | 1 | 1 | 1 | 1 | 1 | 1 | 1 | 1 | 1 | 1 | 1 | 1 | 1 | 1 | 1 | 1 | 1 | 1 | 1 | 1 |
| 72 | No action needed | 1 | 1 | 1 | 1 | 1 | 1 | 1 | 1 | 1 | 1 | 1 | 1 | 1 | 1 | 1 | 1 | 1 | 1 | 1 | 1 |
| 73 | No action needed | 1 | 1 | 1 | 1 | 1 | 1 | 1 | 1 | 1 | 1 | 1 | 1 | 1 | 1 | 1 | 1 | 1 | 1 | 1 | 1 |
| 74 | No action needed | 1 | 1 | 1 | 1 | 1 | 1 | 1 | 1 | 1 | 1 | 1 | 1 | 1 | 1 | 1 | 1 | 1 | 1 | 1 | 1 |
| 75 | No action needed | 1 | 1 | 1 | 1 | 1 | 1 | 1 | 1 | 1 | 1 | 1 | 1 | 1 | 1 | 1 | 1 | 1 | 1 | 1 | 1 |
| 76 | No action needed | 1 | 1 | 1 | 1 | 1 | 1 | 1 | 1 | 1 | 1 | 1 | 1 | 1 | 1 | 1 | 1 | 1 | 1 | 1 | 1 |
| 77 | No action needed | 1 | 1 | 1 | 1 | 1 | 1 | 1 | 1 | 1 | 1 | 1 | 1 | 1 | 1 | 1 | 1 | 1 | 1 | 1 | 1 |
| 78 | No action needed | 1 | 1 | 1 | 1 | 1 | 1 | 1 | 1 | 1 | 1 | 1 | 1 | 1 | 1 | 1 | 1 | 1 | 1 | 1 | 1 |
| 79 | No action needed | 1 | 1 | 1 | 1 | 1 | 1 | 1 | 1 | 1 | 1 | 1 | 1 | 1 | 1 | 1 | 1 | 1 | 1 | 1 | 1 |
| 80 | No action needed | 1 | 1 | 1 | 1 | 1 | 1 | 1 | 1 | 1 | 1 | 1 | 1 | 1 | 1 | 1 | 1 | 1 | 1 | 1 | 1 |
| 81 | No action needed | 1 | 1 | 1 | 1 | 1 | 1 | 1 | 1 | 1 | 1 | 1 | 1 | 1 | 1 | 1 | 1 | 1 | 1 | 1 | 1 |
| 82 | No action needed | 1 | 1 | 1 | 1 | 1 | 1 | 1 | 1 | 1 | 1 | 1 | 1 | 1 | 1 | 1 | 1 | 1 | 1 | 1 | 1 |
| 83 | No action needed | 1 | 1 | 1 | 1 | 1 | 1 | 1 | 1 | 1 | 1 | 1 | 1 | 1 | 1 | 1 | 1 | 1 | 1 | 1 | 1 |
| 84 | No action needed | 1 | 1 | 1 | 1 | 1 | 1 | 1 | 1 | 1 | 1 | 1 | 1 | 1 | 1 | 1 | 1 | 1 | 1 | 1 | 1 |
| 85 | No action needed | 1 | 1 | 1 | 1 | 1 | 1 | 1 | 1 | 1 | 1 | 1 | 1 | 1 | 1 | 1 | 1 | 1 | 1 | 1 | 1 |
| 86 | No action needed | 1 | 1 | 1 | 1 | 1 | 1 | 1 | 1 | 1 | 1 | 1 | 1 | 1 | 1 | 1 | 1 | 1 | 1 | 1 | 1 |
| 87 | No action needed | 1 | 1 | 1 | 1 | 1 | 1 | 1 | 1 | 1 | 1 | 1 | 1 | 1 | 1 | 1 | 1 | 1 | 1 | 1 | 1 |
| 88 | No action needed | 1 | 1 | 1 | 1 | 1 | 1 | 1 | 1 | 1 | 1 | 1 | 1 | 1 | 1 | 1 | 1 | 1 | 1 | 1 | 1 |
| 89 | No action needed | 1 | 1 | 1 | 1 | 1 | 1 | 1 | 1 | 1 | 1 | 1 | 1 | 1 | 1 | 1 | 1 | 1 | 1 | 1 | 1 |
| 90 | No action needed | 1 | 1 | 1 | 1 | 1 | 1 | 1 | 1 | 1 | 1 | 1 | 1 | 1 | 1 | 1 | 1 | 1 | 1 | 1 | 1 |
| 91 | No action needed | 1 | 1 | 1 | 1 | 1 | 1 | 1 | 1 | 1 | 1 | 1 | 1 | 1 | 1 | 1 | 1 | 1 | 1 | 1 | 1 |
| 92 | No action needed | 1 | 1 | 1 | 1 | 1 | 1 | 1 | 1 | 1 | 1 | 1 | 1 | 1 | 1 | 1 | 1 | 1 | 1 | 1 | 1 |
| 93 | No action needed | 1 | 1 | 1 | 1 | 1 | 1 | 1 | 1 | 1 | 1 | 1 | 1 | 1 | 1 | 1 | 1 | 1 | 1 | 1 | 1 |
| 94 | No action needed | 1 | 1 | 1 | 1 | 1 | 1 | 1 | 1 | 1 | 1 | 1 | 1 | 1 | 1 | 1 | 1 | 1 | 1 | 1 | 1 |
| 95 | No action needed | 1 | 1 | 1 | 1 | 1 | 1 | 1 | 1 | 1 | 1 | 1 | 1 | 1 | 1 | 1 | 1 | 1 | 1 | 1 | 1 |
| 96 | No action needed | 1 | 1 | 1 | 1 | 1 | 1 | 1 | 1 | 1 | 1 | 1 | 1 | 1 | 1 | 1 | 1 | 1 | 1 | 1 | 1 |
| 97 | No action needed | 1 | 1 | 1 | 1 | 1 | 1 | 1 | 1 | 1 | 1 | 1 | 1 | 1 | 1 | 1 | 1 | 1 | 1 | 1 | 1 |
| 98 | No action needed | 1 | 1 | 1 | 1 | 1 | 1 | 1 | 1 | 1 | 1 | 1 | 1 | 1 | 1 | 1 | 1 | 1 | 1 | 1 | 1 |
| 99 | No action needed | 1 | 1 | 1 | 1 | 1 | 1 | 1 | 1 | 1 | 1 | 1 | 1 | 1 | 1 | 1 | 1 | 1 | 1 | 1 | 1 |
| 100 | No action needed | 1 | 1 | 1 | 1 | 1 | 1 | 1 | 1 | 1 | 1 | 1 | 1 | 1 | 1 | 1 | 1 | 1 | 1 | 1 | 1 |
| 101 | No action needed | 1 | 1 | 1 | 1 | 1 | 1 | 1 | 1 | 1 | 1 | 1 | 1 | 1 | 1 | 1 | 1 | 1 | 1 | 1 | 1 |
| 102 | No action needed | 1 | 1 | 1 | 1 | 1 | 1 | 1 | 1 | 1 | 1 | 1 | 1 | 1 | 1 | 1 | 1 | 1 | 1 | 1 | 1 |
| 103 | No action needed. | 1 | 1 | 1 | 1 | 1 | 1 | 1 | 1 | 1 | 1 | 1 | 1 | 1 | 1 | 1 | 1 | 1 | 1 | 1 | 1 |
| 104 | No action needed | 1 | 1 | 1 | 1 | 1 | 1 | 1 | 1 | 1 | 1 | 1 | 1 | 1 | 1 | 1 | 1 | 1 | 1 | 1 | 1 |
| 105 | Change medication | 1 | 1 | 1 | 1 | 1 | 1 | 1 | 1 | 1 | 1 | 1 | 1 | 1 | 1 | 1 | 1 | 1 | 1 | 1 | 1 |
| 106 | Change medication | 1 | 1 | 1 | 1 | 1 | 1 | 1 | 1 | 1 | 1 | 1 | 1 | 1 | 1 | 1 | 1 | 1 | 1 | 1 | 1 |
| 107 | No action needed | 1 | 1 | 1 | 1 | 1 | 1 | 1 | 1 | 1 | 1 | 1 | 1 | 1 | 1 | 1 | 1 | 1 | 1 | 1 | 1 |
| 108 | No action needed. | 1 | 1 | 1 | 1 | 1 | 1 | 1 | 1 | 1 | 1 | 1 | 1 | 1 | 1 | 1 | 1 | 1 | 1 | 1 | 1 |
| 109 | No action needed | 1 | 1 | 1 | 1 | 1 | 1 | 1 | 1 | 1 | 1 | 1 | 1 | 1 | 1 | 1 | 1 | 1 | 1 | 1 | 1 |
| 110 | No action needed | 1 | 1 | 1 | 1 | 1 | 1 | 1 | 1 | 1 | 1 | 1 | 1 | 1 | 1 | 1 | 1 | 1 | 1 | 1 | 1 |
| 111 | No action needed | 1 | 1 | 1 | 1 | 1 | 1 | 1 | 1 | 1 | 1 | 1 | 1 | 1 | 1 | 1 | 1 | 1 | 1 | 1 | 1 |
| 112 | No action needed. | 1 | 1 | 1 | 1 | 1 | 1 | 1 | 1 | 1 | 1 | 1 | 1 | 1 | 1 | 1 | 1 | 1 | 1 | 1 | 1 |
| 113 | No action needed | 1 | 1 | 1 | 1 | 1 | 1 | 1 | 1 | 1 | 1 | 1 | 1 | 1 | 1 | 1 | 1 | 1 | 1 | 1 | 1 |
| 114 | No action needed | 1 | 1 | 1 | 1 | 1 | 1 | 1 | 1 | 1 | 1 | 1 | 1 | 1 | 1 | 1 | 1 | 1 | 1 | 1 | 1 |
| 115 | No action needed | 1 | 1 | 1 | 1 | 1 | 1 | 1 | 1 | 1 | 1 | 1 | 1 | 1 | 1 | 1 | 1 | 1 | 1 | 1 | 1 |
| 116 | No action needed | 1 | 1 | 1 | 1 | 1 | 1 | 1 | 1 | 1 | 1 | 1 | 1 | 1 | 1 | 1 | 1 | 1 | 1 | 1 | 1 |
| 117 | No action needed | 1 | 1 | 1 | 1 | 1 | 1 | 1 | 1 | 1 | 1 | 1 | 1 | 1 | 1 | 1 | 1 | 1 | 1 | 1 | 1 |
| 118 | No action needed | 1 | 1 | 1 | 1 | 1 | 1 | 1 | 1 | 1 | 1 | 1 | 1 | 1 | 1 | 1 | 1 | 1 | 1 | 1 | 1 |
| 119 | No action needed | 1 | 1 | 1 | 1 | 1 | 1 | 1 | 1 | 1 | 1 | 1 | 1 | 1 | 1 | 1 | 1 | 1 | 1 | 1 | 1 |
| 120 | No action needed | 1 | 1 | 1 | 1 | 1 | 1 | 1 | 1 | 1 | 1 | 1 | 1 | 1 | 1 | 1 | 1 | 1 | 1 | 1 | 1 |
| 121 | No action needed | 1 | 1 | 1 | 1 | 1 | 1 | 1 | 1 | 1 | 1 | 1 | 1 | 1 | 1 | 1 | 1 | 1 | 1 | 1 | 1 |
| 122 | No action needed | 1 | 1 | 1 | 1 | 1 | 1 | 1 | 1 | 1 | 1 | 1 | 1 | 1 | 1 | 1 | 1 | 1 | 1 | 1 | 1 |
| 123 | No action needed | 1 | 1 | 1 | 1 | 1 | 1 | 1 | 1 | 1 | 1 | 1 | 1 | 1 | 1 | 1 | 1 | 1 | 1 | 1 | 1 |
| 124 | No action needed | 1 | 1 | 1 | 1 | 1 | 1 | 1 | 1 | 1 | 1 | 1 | 1 | 1 | 1 | 1 | 1 | 1 | 1 | 1 | 1 |
| 125 | No action needed | 1 | 1 | 1 | 1 | 1 | 1 | 1 | 1 | 1 | 1 | 1 | 1 | 1 | 1 | 1 | 1 | 1 | 1 | 1 | 1 |
| 126 | Consider dosage modification | 1 | 0 | 0 | 1 | 1 | 1 | 0 | 1 | 1 | 0 | 1 | 0 | 1 | 0 | 1 | 0 | 1 | 0 | 1 | 1 |
| 127 | Change medication | 0 | 1 | 1 | 1 | 1 | 1 | 1 | 1 | 1 | 1 | 1 | 1 | 1 | 0 | 0 | 0 | 0 | 1 | 0 | 1 |
| 128 | No action needed | 1 | 1 | 1 | 1 | 1 | 1 | 1 | 1 | 1 | 1 | 1 | 1 | 1 | 1 | 1 | 1 | 1 | 1 | 1 | 1 |
| 129 | No action needed | 1 | 1 | 1 | 1 | 1 | 1 | 1 | 1 | 1 | 1 | 1 | 1 | 1 | 1 | 1 | 1 | 1 | 1 | 1 | 1 |
| 130 | No action needed | 1 | 1 | 1 | 1 | 1 | 1 | 1 | 1 | 1 | 1 | 1 | 1 | 1 | 1 | 1 | 1 | 1 | 1 | 1 | 1 |
| 131 | No action needed | 1 | 1 | 1 | 1 | 1 | 1 | 1 | 1 | 1 | 1 | 1 | 1 | 1 | 1 | 1 | 1 | 1 | 1 | 1 | 1 |
| 132 | No action needed | 1 | 1 | 1 | 1 | 1 | 1 | 1 | 1 | 1 | 1 | 1 | 1 | 1 | 1 | 1 | 1 | 1 | 1 | 1 | 1 |
| 133 | No action needed | 1 | 1 | 1 | 1 | 1 | 1 | 1 | 1 | 1 | 1 | 1 | 1 | 1 | 1 | 1 | 1 | 1 | 1 | 1 | 1 |
| 134 | No action needed | 1 | 1 | 1 | 1 | 1 | 1 | 1 | 1 | 1 | 1 | 1 | 1 | 1 | 1 | 1 | 1 | 1 | 1 | 1 | 1 |
| 135 | No action needed | 1 | 1 | 1 | 1 | 1 | 1 | 1 | 1 | 1 | 1 | 1 | 1 | 1 | 1 | 1 | 1 | 1 | 1 | 1 | 1 |
| 136 | No action needed | 1 | 1 | 1 | 1 | 1 | 1 | 1 | 1 | 1 | 1 | 1 | 1 | 1 | 1 | 1 | 1 | 1 | 1 | 1 | 1 |
| 137 | No action needed | 1 | 1 | 1 | 1 | 1 | 1 | 1 | 1 | 1 | 1 | 1 | 1 | 1 | 1 | 1 | 1 | 1 | 1 | 1 | 1 |
| 138 | No action needed | 1 | 1 | 1 | 1 | 1 | 1 | 1 | 1 | 1 | 1 | 1 | 1 | 1 | 1 | 1 | 1 | 1 | 1 | 1 | 1 |
| 139 | No action needed | 1 | 1 | 1 | 1 | 1 | 1 | 1 | 1 | 1 | 1 | 1 | 1 | 1 | 1 | 1 | 1 | 1 | 1 | 1 | 1 |
| 140 | No action needed | 1 | 1 | 1 | 1 | 1 | 1 | 1 | 1 | 1 | 1 | 1 | 1 | 1 | 1 | 1 | 1 | 1 | 1 | 1 | 1 |
| 141 | No action needed | 1 | 1 | 1 | 1 | 1 | 1 | 1 | 1 | 1 | 1 | 1 | 1 | 1 | 1 | 1 | 1 | 1 | 1 | 1 | 1 |
| 142 | No action needed | 1 | 1 | 1 | 1 | 1 | 1 | 1 | 1 | 1 | 1 | 1 | 1 | 1 | 1 | 1 | 1 | 1 | 1 | 1 | 1 |
| 143 | No action needed | 1 | 1 | 1 | 1 | 1 | 1 | 1 | 1 | 1 | 1 | 1 | 1 | 1 | 1 | 1 | 1 | 1 | 1 | 1 | 1 |
| 144 | No action needed | 1 | 1 | 1 | 1 | 1 | 1 | 1 | 1 | 1 | 1 | 1 | 1 | 1 | 1 | 1 | 1 | 1 | 1 | 1 | 1 |
| 145 | No action needed | 1 | 1 | 1 | 1 | 1 | 1 | 1 | 1 | 1 | 1 | 1 | 1 | 1 | 1 | 1 | 1 | 1 | 1 | 1 | 1 |
| 146 | No action needed | 1 | 1 | 1 | 1 | 1 | 1 | 1 | 1 | 1 | 1 | 1 | 1 | 1 | 1 | 1 | 1 | 1 | 1 | 1 | 1 |
| 147 | No action needed | 1 | 1 | 1 | 1 | 1 | 1 | 1 | 1 | 1 | 1 | 1 | 1 | 1 | 1 | 1 | 1 | 1 | 1 | 1 | 1 |
| 148 | No action needed | 1 | 1 | 1 | 1 | 1 | 1 | 1 | 1 | 1 | 1 | 1 | 1 | 1 | 1 | 1 | 1 | 1 | 1 | 1 | 1 |
| 149 | No action needed. | 1 | 1 | 1 | 1 | 1 | 1 | 1 | 1 | 1 | 1 | 1 | 1 | 1 | 1 | 1 | 1 | 1 | 1 | 1 | 1 |
| 150 | No action needed | 1 | 1 | 1 | 1 | 1 | 1 | 1 | 1 | 1 | 1 | 1 | 1 | 1 | 1 | 1 | 1 | 1 | 1 | 1 | 1 |
| 151 | No action needed | 1 | 1 | 1 | 1 | 1 | 1 | 1 | 1 | 1 | 1 | 1 | 1 | 1 | 1 | 1 | 1 | 1 | 1 | 1 | 1 |
| 152 | No action needed | 1 | 1 | 1 | 1 | 1 | 1 | 1 | 1 | 1 | 1 | 1 | 1 | 1 | 1 | 1 | 1 | 1 | 1 | 1 | 1 |
| 153 | No action needed. | 1 | 1 | 1 | 1 | 1 | 1 | 1 | 1 | 1 | 1 | 1 | 1 | 1 | 1 | 1 | 1 | 1 | 1 | 1 | 1 |
| 154 | No action needed | 1 | 1 | 1 | 1 | 1 | 1 | 1 | 1 | 1 | 1 | 1 | 1 | 1 | 1 | 1 | 1 | 1 | 1 | 1 | 1 |
| 155 | No action needed | 1 | 1 | 1 | 1 | 1 | 1 | 1 | 1 | 1 | 1 | 1 | 1 | 1 | 1 | 1 | 1 | 1 | 1 | 1 | 1 |
| 156 | No action needed | 1 | 1 | 1 | 1 | 1 | 1 | 1 | 1 | 1 | 1 | 1 | 1 | 1 | 1 | 1 | 1 | 1 | 1 | 1 | 1 |
| 157 | Consider dosage modification | 1 | 1 | 1 | 1 | 1 | 1 | 1 | 1 | 1 | 1 | 1 | 1 | 1 | 1 | 1 | 1 | 1 | 1 | 1 | 1 |
| 158 | Consider dosage modification | 1 | 1 | 1 | 1 | 1 | 1 | 1 | 1 | 1 | 1 | 1 | 1 | 1 | 1 | 1 | 1 | 1 | 1 | 1 | 1 |
| 159 | Consider dosage modification | 1 | 1 | 1 | 1 | 1 | 1 | 1 | 1 | 1 | 1 | 1 | 1 | 1 | 1 | 1 | 1 | 1 | 1 | 1 | 1 |
| 160 | Consider dosage modification | 1 | 1 | 1 | 1 | 1 | 1 | 1 | 1 | 1 | 1 | 1 | 1 | 1 | 1 | 1 | 1 | 1 | 1 | 1 | 1 |
| 161 | No action needed | 1 | 1 | 1 | 1 | 1 | 1 | 1 | 1 | 1 | 1 | 1 | 1 | 1 | 1 | 1 | 1 | 1 | 1 | 1 | 1 |
| 162 | No action needed | 1 | 1 | 1 | 1 | 1 | 1 | 1 | 1 | 1 | 1 | 1 | 1 | 1 | 1 | 1 | 1 | 1 | 1 | 1 | 1 |
| 163 | No action needed | 1 | 1 | 1 | 1 | 1 | 1 | 1 | 1 | 1 | 1 | 1 | 1 | 1 | 1 | 1 | 1 | 1 | 1 | 1 | 1 |
| 164 | No action needed | 1 | 1 | 1 | 1 | 1 | 1 | 1 | 1 | 1 | 1 | 1 | 1 | 1 | 1 | 1 | 1 | 1 | 1 | 1 | 1 |
| 165 | No action needed | 1 | 1 | 1 | 1 | 1 | 1 | 1 | 1 | 1 | 1 | 1 | 1 | 1 | 1 | 1 | 1 | 1 | 1 | 1 | 1 |
| 166 | No action needed | 1 | 1 | 1 | 1 | 1 | 1 | 1 | 1 | 1 | 1 | 1 | 1 | 1 | 1 | 1 | 1 | 1 | 1 | 1 | 1 |
| 167 | No action needed | 1 | 1 | 1 | 1 | 1 | 1 | 1 | 1 | 1 | 1 | 1 | 1 | 1 | 1 | 1 | 1 | 1 | 1 | 1 | 1 |
| 168 | No action needed | 1 | 1 | 1 | 1 | 1 | 1 | 1 | 1 | 1 | 1 | 1 | 1 | 1 | 1 | 1 | 1 | 1 | 1 | 1 | 1 |
| 169 | No action needed | 1 | 1 | 1 | 1 | 1 | 1 | 1 | 1 | 1 | 1 | 1 | 1 | 1 | 1 | 1 | 1 | 1 | 1 | 1 | 1 |
| 170 | No action needed | 1 | 1 | 1 | 1 | 1 | 1 | 1 | 1 | 1 | 1 | 1 | 1 | 1 | 1 | 1 | 1 | 1 | 1 | 1 | 1 |
| 171 | No action needed | 1 | 1 | 1 | 1 | 1 | 1 | 1 | 1 | 1 | 1 | 1 | 1 | 1 | 1 | 1 | 1 | 1 | 1 | 1 | 1 |
| 172 | Consider dosage modification | 1 | 1 | 0 | 1 | 1 | 0 | 1 | 0 | 1 | 0 | 1 | 1 | 1 | 1 | 1 | 1 | 1 | 1 | 1 | 0 |
| 173 | No action needed | 1 | 1 | 1 | 1 | 1 | 1 | 1 | 1 | 1 | 1 | 1 | 1 | 1 | 1 | 1 | 1 | 1 | 1 | 1 | 1 |
| 174 | No action needed | 1 | 1 | 1 | 1 | 1 | 1 | 1 | 1 | 1 | 1 | 1 | 1 | 1 | 1 | 1 | 1 | 1 | 1 | 1 | 1 |
| 175 | No action needed | 1 | 1 | 1 | 1 | 1 | 1 | 1 | 1 | 1 | 1 | 1 | 1 | 1 | 1 | 1 | 1 | 1 | 1 | 1 | 1 |
| 176 | No action needed | 1 | 1 | 1 | 1 | 1 | 1 | 1 | 1 | 1 | 1 | 1 | 1 | 1 | 1 | 1 | 1 | 1 | 1 | 1 | 1 |
| 177 | No action needed | 1 | 1 | 1 | 1 | 1 | 1 | 1 | 1 | 1 | 1 | 1 | 1 | 1 | 1 | 1 | 1 | 1 | 1 | 1 | 1 |
| 178 | No action needed | 1 | 1 | 1 | 1 | 1 | 1 | 1 | 1 | 1 | 1 | 1 | 1 | 1 | 1 | 1 | 1 | 1 | 1 | 1 | 1 |
| 179 | Consider dosage modification | 1 | 1 | 1 | 1 | 1 | 1 | 1 | 1 | 1 | 1 | 1 | 1 | 1 | 1 | 1 | 1 | 1 | 1 | 1 | 1 |
| 180 | Consider dosage modification | 1 | 1 | 1 | 1 | 1 | 0 | 0 | 1 | 1 | 1 | 0 | 1 | 1 | 1 | 1 | 1 | 0 | 1 | 1 | 1 |
| 181 | Change medication | 1 | 0 | 0 | 0 | 1 | 0 | 0 | 0 | 1 | 1 | 1 | 0 | 1 | 1 | 0 | 1 | 1 | 1 | 1 | 1 |
| 182 | Change medication | 1 | 1 | 1 | 1 | 1 | 1 | 1 | 1 | 1 | 1 | 1 | 1 | 1 | 1 | 1 | 1 | 1 | 1 | 1 | 1 |
| 183 | Change medication | 1 | 1 | 1 | 1 | 1 | 1 | 1 | 1 | 1 | 1 | 1 | 1 | 1 | 1 | 1 | 1 | 1 | 1 | 1 | 1 |
| 184 | Change medication | 1 | 1 | 1 | 1 | 1 | 1 | 1 | 1 | 1 | 1 | 1 | 1 | 1 | 1 | 1 | 1 | 1 | 1 | 1 | 1 |
| 185 | Change medication | 1 | 1 | 1 | 1 | 1 | 1 | 1 | 1 | 1 | 1 | 1 | 1 | 1 | 1 | 1 | 1 | 1 | 1 | 1 | 1 |
| 186 | Change medication | 1 | 1 | 1 | 1 | 1 | 1 | 1 | 1 | 1 | 1 | 1 | 1 | 1 | 1 | 1 | 1 | 1 | 1 | 1 | 1 |
| 187 | Change medication | 1 | 1 | 1 | 1 | 1 | 1 | 1 | 1 | 1 | 1 | 1 | 1 | 1 | 1 | 1 | 1 | 1 | 1 | 1 | 1 |
| 188 | Change medication | 1 | 1 | 1 | 1 | 1 | 1 | 1 | 1 | 1 | 1 | 1 | 1 | 1 | 1 | 1 | 1 | 1 | 1 | 1 | 1 |
| 189 | Change medication | 1 | 1 | 1 | 1 | 1 | 1 | 1 | 1 | 1 | 1 | 1 | 1 | 1 | 1 | 1 | 1 | 1 | 1 | 1 | 1 |
| 190 | Change medication | 1 | 1 | 1 | 1 | 1 | 1 | 1 | 1 | 1 | 1 | 1 | 1 | 1 | 1 | 1 | 1 | 1 | 1 | 1 | 1 |
| 191 | Change medication | 1 | 1 | 1 | 1 | 1 | 1 | 1 | 1 | 1 | 1 | 1 | 1 | 1 | 1 | 1 | 1 | 1 | 1 | 1 | 1 |
| 192 | Change medication | 1 | 1 | 1 | 1 | 1 | 1 | 1 | 1 | 1 | 1 | 1 | 1 | 1 | 1 | 1 | 1 | 1 | 1 | 1 | 1 |
| 193 | Change medication | 1 | 1 | 1 | 1 | 1 | 1 | 1 | 1 | 1 | 1 | 1 | 1 | 1 | 1 | 1 | 1 | 1 | 1 | 1 | 1 |
| 194 | Change medication | 1 | 1 | 1 | 1 | 1 | 1 | 1 | 1 | 1 | 1 | 1 | 1 | 1 | 1 | 1 | 1 | 1 | 1 | 1 | 1 |
| 195 | Change medication | 1 | 1 | 1 | 1 | 1 | 1 | 1 | 1 | 1 | 1 | 1 | 1 | 1 | 1 | 1 | 1 | 1 | 1 | 1 | 1 |
| 196 | Change medication | 1 | 1 | 1 | 1 | 1 | 1 | 1 | 1 | 1 | 1 | 1 | 1 | 1 | 1 | 1 | 1 | 1 | 1 | 1 | 1 |
| 197 | No action needed | 1 | 1 | 1 | 1 | 1 | 1 | 1 | 1 | 1 | 1 | 1 | 1 | 1 | 1 | 1 | 1 | 1 | 1 | 1 | 1 |
| 198 | Consider dosage modification | 1 | 1 | 1 | 1 | 1 | 1 | 1 | 1 | 1 | 1 | 0 | 1 | 1 | 1 | 1 | 0 | 1 | 1 | 1 | 1 |
| 199 | Consider dosage modification | 1 | 1 | 1 | 1 | 0 | 1 | 0 | 0 | 0 | 1 | 1 | 1 | 1 | 1 | 1 | 1 | 1 | 0 | 1 | 1 |
| 200 | No action needed | 1 | 1 | 1 | 1 | 1 | 1 | 1 | 1 | 1 | 1 | 1 | 1 | 1 | 1 | 1 | 1 | 1 | 1 | 1 | 1 |
| 201 | Change medication | 1 | 1 | 1 | 1 | 0 | 1 | 1 | 1 | 1 | 1 | 1 | 1 | 1 | 1 | 1 | 1 | 1 | 1 | 1 | 1 |
| 202 | Consider dosage modification | 1 | 1 | 1 | 1 | 1 | 1 | 1 | 1 | 1 | 1 | 1 | 1 | 1 | 1 | 1 | 1 | 1 | 1 | 1 | 1 |
| 203 | Change medication | 1 | 1 | 1 | 1 | 1 | 1 | 1 | 1 | 1 | 1 | 1 | 1 | 1 | 1 | 1 | 1 | 1 | 1 | 1 | 1 |
| 204 | Consider dosage modification | 1 | 1 | 1 | 1 | 1 | 1 | 1 | 1 | 1 | 1 | 1 | 1 | 1 | 1 | 1 | 1 | 1 | 1 | 1 | 1 |
| 205 | Change medication | 0 | 1 | 1 | 1 | 1 | 0 | 1 | 0 | 1 | 1 | 1 | 1 | 1 | 1 | 1 | 0 | 1 | 1 | 1 | 1 |
| 206 | Change medication | 1 | 1 | 1 | 1 | 1 | 1 | 1 | 1 | 1 | 1 | 1 | 1 | 1 | 1 | 1 | 1 | 1 | 1 | 1 | 1 |
| 207 | Change medication | 1 | 1 | 1 | 1 | 1 | 1 | 1 | 1 | 1 | 1 | 1 | 1 | 1 | 1 | 1 | 1 | 1 | 1 | 1 | 1 |
| 208 | Change medication | 1 | 1 | 1 | 1 | 1 | 1 | 1 | 1 | 1 | 1 | 1 | 1 | 1 | 1 | 1 | 1 | 1 | 1 | 1 | 1 |
| 209 | Consider dosage modification | 1 | 1 | 1 | 1 | 1 | 1 | 1 | 1 | 1 | 1 | 1 | 1 | 1 | 1 | 1 | 1 | 1 | 1 | 1 | 1 |
| 210 | Consider dosage modification | 1 | 1 | 1 | 1 | 1 | 1 | 1 | 1 | 1 | 1 | 1 | 1 | 1 | 1 | 0 | 1 | 1 | 1 | 1 | 0 |
| 211 | Change medication | 0 | 0 | 1 | 1 | 1 | 0 | 0 | 0 | 1 | 0 | 1 | 1 | 1 | 1 | 1 | 1 | 0 | 1 | 0 | 1 |
| 212 | No action needed | 1 | 1 | 1 | 1 | 1 | 1 | 1 | 1 | 1 | 1 | 1 | 1 | 1 | 1 | 1 | 1 | 1 | 1 | 1 | 1 |
| 213 | No action needed | 1 | 1 | 1 | 1 | 1 | 1 | 1 | 1 | 1 | 1 | 1 | 1 | 1 | 1 | 1 | 1 | 1 | 1 | 1 | 1 |
| 214 | No action needed | 1 | 1 | 1 | 1 | 1 | 1 | 1 | 1 | 1 | 1 | 1 | 1 | 1 | 1 | 1 | 1 | 1 | 1 | 1 | 1 |
| 215 | No action needed | 1 | 1 | 1 | 1 | 1 | 1 | 1 | 1 | 1 | 1 | 1 | 1 | 1 | 1 | 1 | 1 | 1 | 1 | 1 | 1 |
| 216 | No action needed | 1 | 1 | 1 | 1 | 1 | 1 | 1 | 1 | 1 | 1 | 1 | 1 | 1 | 1 | 1 | 1 | 1 | 1 | 1 | 1 |
| 217 | No action needed | 1 | 1 | 1 | 1 | 1 | 1 | 1 | 1 | 1 | 1 | 1 | 1 | 1 | 1 | 1 | 1 | 1 | 1 | 1 | 1 |
| 218 | No action needed | 1 | 1 | 1 | 1 | 1 | 1 | 1 | 1 | 1 | 1 | 1 | 1 | 1 | 1 | 1 | 1 | 1 | 1 | 1 | 1 |
| 219 | No action needed. | 1 | 1 | 1 | 1 | 1 | 1 | 1 | 1 | 1 | 1 | 1 | 1 | 1 | 1 | 1 | 1 | 1 | 1 | 1 | 1 |
| 220 | No action needed | 1 | 1 | 1 | 1 | 1 | 1 | 1 | 1 | 1 | 1 | 1 | 1 | 1 | 1 | 1 | 1 | 1 | 1 | 1 | 1 |
| 221 | No action needed | 1 | 1 | 1 | 1 | 1 | 1 | 1 | 1 | 1 | 1 | 1 | 1 | 1 | 1 | 1 | 1 | 1 | 1 | 1 | 1 |
| 222 | Consider dosage modification | 1 | 1 | 1 | 1 | 1 | 1 | 1 | 1 | 1 | 1 | 1 | 1 | 1 | 1 | 1 | 1 | 1 | 1 | 1 | 1 |
| 223 | Consider dosage modification | 1 | 1 | 1 | 1 | 1 | 1 | 1 | 1 | 1 | 1 | 1 | 1 | 1 | 1 | 1 | 1 | 1 | 1 | 1 | 1 |
| 224 | Consider dosage modification | 1 | 1 | 1 | 1 | 1 | 1 | 1 | 1 | 1 | 1 | 1 | 1 | 1 | 1 | 1 | 1 | 1 | 1 | 1 | 1 |
| 225 | Consider dosage modification | 1 | 1 | 1 | 1 | 1 | 1 | 1 | 1 | 1 | 1 | 1 | 1 | 1 | 1 | 1 | 1 | 1 | 1 | 1 | 1 |
| 226 | Consider dosage modification | 1 | 1 | 1 | 1 | 1 | 1 | 1 | 1 | 1 | 1 | 1 | 1 | 1 | 1 | 1 | 1 | 1 | 1 | 1 | 1 |
| 227 | Consider dosage modification | 1 | 1 | 1 | 1 | 1 | 1 | 1 | 0 | 1 | 1 | 1 | 1 | 1 | 1 | 1 | 1 | 0 | 1 | 1 | 1 |
| 228 | Consider dosage modification | 1 | 1 | 1 | 1 | 1 | 1 | 1 | 1 | 1 | 1 | 1 | 1 | 1 | 1 | 1 | 1 | 1 | 1 | 1 | 1 |
| 229 | Consider dosage modification | 1 | 1 | 0 | 1 | 1 | 1 | 0 | 1 | 1 | 0 | 0 | 1 | 1 | 0 | 1 | 1 | 0 | 1 | 0 | 1 |
| 230 | Change medication | 1 | 1 | 1 | 1 | 1 | 1 | 1 | 1 | 1 | 1 | 1 | 1 | 1 | 1 | 1 | 1 | 1 | 0 | 1 | 1 |
| 231 | No action needed | 1 | 1 | 1 | 1 | 1 | 1 | 1 | 1 | 1 | 1 | 1 | 1 | 1 | 1 | 1 | 1 | 1 | 1 | 1 | 1 |
| 232 | Change medication | 1 | 1 | 1 | 1 | 1 | 1 | 1 | 1 | 1 | 1 | 1 | 1 | 1 | 1 | 1 | 1 | 1 | 1 | 1 | 1 |
| 233 | Change medication | 1 | 1 | 1 | 1 | 1 | 1 | 1 | 1 | 1 | 1 | 1 | 1 | 1 | 1 | 1 | 1 | 1 | 1 | 1 | 1 |
| 234 | Change medication | 1 | 1 | 1 | 1 | 1 | 1 | 1 | 1 | 1 | 1 | 1 | 1 | 1 | 1 | 1 | 1 | 1 | 1 | 1 | 1 |
| 235 | Consider dosage modification | 1 | 1 | 1 | 0 | 0 | 1 | 1 | 1 | 1 | 1 | 1 | 0 | 1 | 1 | 1 | 1 | 1 | 1 | 1 | 1 |
| 236 | Consider dosage modification | 1 | 0 | 1 | 1 | 1 | 1 | 1 | 1 | 1 | 1 | 1 | 1 | 1 | 1 | 1 | 1 | 1 | 1 | 1 | 1 |
| 237 | Consider dosage modification | 1 | 0 | 1 | 1 | 0 | 0 | 1 | 1 | 0 | 0 | 1 | 0 | 1 | 1 | 1 | 0 | 1 | 1 | 1 | 0 |
| 238 | Change medication | 1 | 1 | 1 | 1 | 1 | 1 | 1 | 1 | 1 | 1 | 1 | 1 | 1 | 1 | 1 | 1 | 1 | 1 | 1 | 1 |
| 239 | Consider dosage modification | 1 | 1 | 1 | 1 | 1 | 1 | 1 | 1 | 1 | 1 | 1 | 1 | 1 | 1 | 1 | 1 | 1 | 1 | 1 | 1 |
| 240 | No action needed | 1 | 1 | 1 | 1 | 1 | 1 | 1 | 1 | 1 | 1 | 1 | 1 | 1 | 1 | 1 | 1 | 1 | 1 | 1 | 1 |
| 241 | Consider dosage modification | 1 | 1 | 1 | 1 | 1 | 1 | 1 | 1 | 1 | 1 | 1 | 1 | 1 | 1 | 1 | 1 | 1 | 1 | 1 | 1 |
| 242 | No action needed | 1 | 1 | 1 | 1 | 1 | 1 | 1 | 1 | 1 | 1 | 1 | 1 | 1 | 1 | 1 | 1 | 1 | 1 | 1 | 1 |
| 243 | Consider dosage modification | 1 | 1 | 1 | 1 | 1 | 1 | 1 | 1 | 1 | 1 | 1 | 1 | 1 | 1 | 1 | 1 | 1 | 1 | 1 | 1 |
| 244 | Consider dosage modification | 1 | 1 | 1 | 1 | 0 | 1 | 1 | 1 | 1 | 1 | 1 | 1 | 1 | 1 | 1 | 1 | 1 | 1 | 1 | 1 |
| 245 | Consider dosage modification | 1 | 1 | 0 | 1 | 1 | 1 | 1 | 1 | 1 | 1 | 1 | 1 | 1 | 1 | 1 | 1 | 1 | 1 | 1 | 0 |
| 246 | Consider dosage modification | 1 | 1 | 1 | 1 | 1 | 1 | 1 | 1 | 1 | 1 | 1 | 1 | 1 | 1 | 1 | 1 | 1 | 1 | 1 | 1 |
| 247 | Consider dosage modification | 1 | 1 | 1 | 1 | 1 | 1 | 1 | 1 | 1 | 1 | 1 | 1 | 1 | 1 | 1 | 1 | 1 | 1 | 1 | 1 |
| 248 | Consider dosage modification | 1 | 1 | 1 | 1 | 1 | 1 | 1 | 1 | 1 | 1 | 1 | 1 | 1 | 1 | 1 | 1 | 1 | 1 | 1 | 1 |
| 249 | Change medication | 1 | 1 | 1 | 1 | 1 | 1 | 1 | 1 | 1 | 1 | 1 | 1 | 1 | 1 | 1 | 1 | 1 | 1 | 1 | 1 |
| 250 | Change medication | 1 | 1 | 1 | 1 | 1 | 1 | 1 | 1 | 1 | 1 | 1 | 1 | 1 | 1 | 1 | 1 | 1 | 1 | 1 | 1 |
| 251 | Consider dosage modification | 1 | 1 | 1 | 1 | 1 | 1 | 1 | 1 | 1 | 1 | 1 | 1 | 1 | 1 | 0 | 1 | 1 | 1 | 1 | 1 |
| 252 | Change medication | 1 | 1 | 1 | 1 | 1 | 1 | 1 | 1 | 1 | 1 | 1 | 1 | 1 | 1 | 1 | 1 | 1 | 1 | 1 | 1 |
| 253 | Change medication | 1 | 1 | 1 | 1 | 0 | 0 | 1 | 1 | 0 | 1 | 1 | 1 | 1 | 1 | 0 | 1 | 1 | 1 | 0 | 1 |
| 254 | Consider dosage modification | 1 | 1 | 0 | 1 | 1 | 0 | 1 | 0 | 1 | 1 | 1 | 0 | 1 | 0 | 0 | 0 | 1 | 1 | 1 | 1 |
| 255 | Consider dosage modification | 1 | 1 | 1 | 1 | 1 | 1 | 1 | 1 | 1 | 1 | 1 | 1 | 1 | 1 | 1 | 1 | 1 | 1 | 1 | 1 |
| 256 | Consider dosage modification | 1 | 1 | 1 | 1 | 1 | 1 | 1 | 1 | 1 | 1 | 1 | 1 | 1 | 1 | 1 | 1 | 1 | 1 | 1 | 1 |
| 257 | Change medication | 1 | 1 | 1 | 1 | 1 | 1 | 1 | 1 | 1 | 1 | 1 | 1 | 1 | 1 | 1 | 1 | 1 | 1 | 1 | 1 |
| 258 | Change medication | 1 | 1 | 1 | 1 | 1 | 1 | 1 | 1 | 1 | 1 | 1 | 1 | 1 | 1 | 1 | 1 | 1 | 1 | 1 | 1 |
| 259 | Change medication | 1 | 1 | 1 | 1 | 1 | 1 | 1 | 1 | 1 | 1 | 1 | 1 | 1 | 1 | 1 | 1 | 1 | 1 | 1 | 1 |
| 260 | Change medication | 1 | 1 | 1 | 1 | 1 | 1 | 1 | 1 | 1 | 1 | 1 | 1 | 1 | 1 | 1 | 1 | 1 | 1 | 1 | 1 |
| 261 | Change medication | 1 | 1 | 1 | 1 | 1 | 1 | 1 | 1 | 1 | 1 | 1 | 1 | 1 | 1 | 1 | 1 | 1 | 1 | 1 | 1 |
| 262 | Consider dosage modification | 1 | 1 | 1 | 1 | 1 | 1 | 1 | 1 | 1 | 1 | 1 | 1 | 1 | 1 | 1 | 1 | 1 | 1 | 1 | 1 |
| 263 | Consider dosage modification | 1 | 1 | 1 | 1 | 1 | 1 | 1 | 1 | 1 | 1 | 1 | 1 | 1 | 1 | 1 | 1 | 1 | 1 | 1 | 1 |
| 264 | Consider dosage modification | 1 | 1 | 1 | 1 | 1 | 1 | 1 | 1 | 1 | 1 | 1 | 1 | 1 | 1 | 1 | 1 | 1 | 1 | 1 | 1 |
| 265 | Change medication | 1 | 1 | 1 | 1 | 1 | 1 | 1 | 1 | 1 | 1 | 1 | 1 | 1 | 1 | 1 | 1 | 1 | 1 | 1 | 1 |
| 266 | Monitor adverse effects | 1 | 1 | 1 | 1 | 1 | 1 | 1 | 1 | 1 | 1 | 1 | 1 | 1 | 1 | 1 | 1 | 1 | 1 | 1 | 1 |
| 267 | Change medication | 1 | 1 | 1 | 1 | 1 | 1 | 1 | 1 | 1 | 1 | 1 | 1 | 1 | 1 | 1 | 1 | 1 | 1 | 1 | 1 |
| 268 | No action needed | 1 | 1 | 1 | 1 | 1 | 1 | 1 | 1 | 1 | 1 | 1 | 1 | 1 | 1 | 1 | 1 | 1 | 1 | 1 | 1 |
| 269 | Consider dosage modification | 1 | 1 | 1 | 1 | 1 | 1 | 1 | 1 | 1 | 1 | 1 | 1 | 1 | 1 | 1 | 1 | 1 | 1 | 1 | 1 |
| 270 | Change medication | 1 | 1 | 1 | 1 | 1 | 1 | 1 | 1 | 1 | 1 | 1 | 1 | 1 | 1 | 1 | 1 | 1 | 1 | 1 | 1 |
| 271 | Change medication | 1 | 1 | 1 | 1 | 1 | 1 | 1 | 1 | 1 | 1 | 1 | 1 | 1 | 1 | 1 | 1 | 1 | 1 | 1 | 1 |
| 272 | Change medication | 1 | 1 | 1 | 1 | 1 | 1 | 1 | 1 | 1 | 1 | 1 | 1 | 1 | 1 | 1 | 1 | 1 | 1 | 1 | 1 |
| 273 | Consider dosage modification | 1 | 1 | 1 | 1 | 1 | 1 | 1 | 1 | 1 | 1 | 1 | 1 | 1 | 1 | 1 | 1 | 1 | 1 | 1 | 1 |
| 274 | Change medication | 1 | 1 | 1 | 1 | 1 | 1 | 1 | 1 | 1 | 1 | 1 | 1 | 1 | 1 | 1 | 1 | 1 | 1 | 1 | 1 |
| 275 | Consider dosage modification | 1 | 1 | 1 | 1 | 1 | 1 | 1 | 1 | 1 | 1 | 1 | 1 | 1 | 1 | 1 | 1 | 1 | 1 | 1 | 1 |
| 276 | Consider dosage modification | 1 | 1 | 1 | 1 | 1 | 1 | 1 | 1 | 1 | 1 | 1 | 1 | 1 | 1 | 1 | 1 | 1 | 1 | 1 | 1 |
| 277 | Monitor adverse effects | 1 | 1 | 1 | 1 | 1 | 1 | 1 | 1 | 1 | 1 | 1 | 1 | 1 | 1 | 1 | 1 | 1 | 1 | 1 | 1 |
| 278 | Monitor adverse effects | 0 | 1 | 0 | 1 | 1 | 1 | 1 | 1 | 0 | 0 | 0 | 1 | 1 | 1 | 1 | 0 | 1 | 0 | 1 | 0 |
| 279 | Monitor adverse effects | 1 | 1 | 1 | 1 | 1 | 0 | 1 | 1 | 1 | 1 | 0 | 0 | 1 | 0 | 1 | 1 | 1 | 1 | 0 | 1 |
| 280 | Monitor adverse effects | 0 | 1 | 1 | 1 | 1 | 1 | 1 | 1 | 1 | 1 | 1 | 1 | 1 | 1 | 1 | 1 | 1 | 0 | 0 | 1 |
| 281 | Monitor adverse effects | 1 | 1 | 1 | 1 | 0 | 1 | 1 | 1 | 1 | 1 | 1 | 1 | 1 | 1 | 1 | 1 | 1 | 0 | 1 | 1 |
| 282 | Monitor adverse effects | 1 | 1 | 0 | 1 | 1 | 1 | 1 | 1 | 1 | 1 | 1 | 1 | 1 | 1 | 0 | 1 | 1 | 1 | 1 | 1 |
| 283 | Consider dosage modification | 1 | 1 | 1 | 1 | 1 | 1 | 1 | 1 | 1 | 1 | 1 | 1 | 1 | 1 | 1 | 1 | 1 | 1 | 1 | 1 |
| 284 | Consider dosage modification | 1 | 1 | 1 | 1 | 1 | 1 | 1 | 1 | 1 | 1 | 1 | 1 | 1 | 1 | 1 | 1 | 1 | 1 | 1 | 1 |
| 285 | Change medication | 1 | 1 | 1 | 1 | 1 | 1 | 1 | 1 | 1 | 1 | 1 | 1 | 1 | 1 | 1 | 1 | 1 | 1 | 1 | 1 |
| 286 | No action needed | 1 | 1 | 1 | 1 | 1 | 1 | 1 | 1 | 1 | 1 | 1 | 1 | 1 | 1 | 1 | 1 | 1 | 1 | 1 | 1 |
| 287 | Change medication | 1 | 1 | 1 | 1 | 1 | 1 | 1 | 1 | 1 | 0 | 1 | 1 | 1 | 1 | 1 | 1 | 1 | 1 | 1 | 1 |
| 288 | No action needed | 1 | 1 | 1 | 1 | 1 | 1 | 1 | 1 | 1 | 1 | 1 | 1 | 1 | 1 | 1 | 1 | 1 | 1 | 1 | 1 |
| 289 | No action needed | 1 | 1 | 1 | 1 | 1 | 1 | 1 | 1 | 1 | 1 | 1 | 1 | 1 | 1 | 1 | 1 | 1 | 1 | 1 | 1 |
| 290 | Consider dosage modification | 1 | 1 | 1 | 1 | 1 | 1 | 1 | 1 | 1 | 1 | 1 | 1 | 1 | 1 | 1 | 1 | 1 | 1 | 1 | 1 |
| 291 | Consider dosage modification | 1 | 1 | 1 | 1 | 1 | 1 | 1 | 1 | 1 | 1 | 1 | 1 | 1 | 1 | 1 | 1 | 1 | 1 | 1 | 1 |
| 292 | Consider dosage modification | 1 | 1 | 1 | 1 | 1 | 1 | 1 | 1 | 1 | 1 | 1 | 1 | 1 | 1 | 1 | 1 | 1 | 1 | 1 | 1 |
| 293 | No action needed | 1 | 1 | 1 | 1 | 1 | 1 | 1 | 1 | 1 | 1 | 1 | 1 | 1 | 1 | 1 | 1 | 1 | 1 | 1 | 1 |
| 294 | Change medication | 1 | 1 | 1 | 1 | 1 | 1 | 1 | 1 | 1 | 1 | 1 | 1 | 1 | 1 | 1 | 1 | 1 | 1 | 1 | 1 |
| 295 | No action needed | 1 | 1 | 1 | 1 | 1 | 1 | 1 | 1 | 1 | 1 | 1 | 1 | 1 | 1 | 1 | 1 | 1 | 1 | 1 | 1 |
| 296 | Change medication | 1 | 1 | 1 | 1 | 1 | 1 | 1 | 1 | 1 | 1 | 1 | 1 | 1 | 1 | 1 | 1 | 1 | 1 | 1 | 1 |
| 297 | Change medication | 1 | 1 | 1 | 1 | 1 | 1 | 1 | 1 | 1 | 1 | 1 | 1 | 1 | 1 | 1 | 1 | 1 | 1 | 1 | 1 |
| 298 | Change medication | 1 | 1 | 1 | 1 | 1 | 1 | 1 | 1 | 1 | 1 | 0 | 1 | 1 | 1 | 1 | 1 | 1 | 1 | 1 | 0 |
| 299 | Consider dosage modification | 1 | 1 | 1 | 1 | 1 | 1 | 1 | 1 | 1 | 1 | 1 | 1 | 1 | 1 | 1 | 1 | 1 | 1 | 1 | 1 |
| 300 | Change medication | 1 | 1 | 1 | 1 | 1 | 1 | 1 | 1 | 1 | 1 | 1 | 1 | 1 | 1 | 1 | 1 | 1 | 1 | 1 | 1 |
| 301 | Change medication | 1 | 1 | 1 | 1 | 1 | 1 | 1 | 1 | 1 | 1 | 1 | 1 | 1 | 1 | 1 | 1 | 1 | 1 | 1 | 1 |
| 302 | No action needed | 1 | 1 | 1 | 1 | 1 | 1 | 1 | 1 | 1 | 1 | 1 | 1 | 1 | 1 | 1 | 1 | 1 | 1 | 1 | 1 |
| 303 | No action needed | 1 | 1 | 1 | 1 | 1 | 1 | 1 | 1 | 1 | 1 | 1 | 1 | 1 | 1 | 1 | 1 | 1 | 1 | 1 | 1 |
| 304 | Change medication | 1 | 1 | 1 | 1 | 1 | 1 | 0 | 1 | 1 | 1 | 1 | 1 | 0 | 1 | 1 | 1 | 1 | 1 | 1 | 1 |
| 305 | No action needed | 1 | 1 | 1 | 1 | 1 | 1 | 1 | 1 | 1 | 1 | 1 | 1 | 1 | 1 | 1 | 1 | 1 | 1 | 1 | 1 |
| 306 | No action needed | 1 | 1 | 1 | 1 | 1 | 1 | 1 | 1 | 1 | 1 | 1 | 1 | 1 | 1 | 1 | 1 | 1 | 1 | 1 | 1 |
| 307 | Consider dosage modification | 0 | 1 | 1 | 1 | 1 | 1 | 1 | 1 | 1 | 1 | 1 | 1 | 1 | 1 | 1 | 1 | 1 | 1 | 1 | 1 |
| 308 | No action needed | 1 | 1 | 1 | 1 | 1 | 1 | 1 | 1 | 1 | 1 | 1 | 1 | 1 | 1 | 1 | 1 | 1 | 1 | 1 | 1 |
| 309 | Change medication | 1 | 1 | 1 | 1 | 0 | 1 | 1 | 0 | 1 | 1 | 1 | 0 | 1 | 1 | 0 | 1 | 1 | 1 | 1 | 0 |
| 310 | No action needed. | 1 | 1 | 1 | 1 | 1 | 1 | 1 | 1 | 1 | 1 | 1 | 1 | 1 | 1 | 1 | 1 | 1 | 1 | 1 | 1 |
| 311 | No action needed | 1 | 1 | 1 | 1 | 1 | 1 | 1 | 1 | 1 | 1 | 1 | 1 | 1 | 1 | 1 | 1 | 1 | 1 | 1 | 1 |
| 312 | Change medication | 1 | 1 | 1 | 1 | 1 | 1 | 1 | 1 | 1 | 1 | 1 | 1 | 1 | 1 | 1 | 1 | 1 | 1 | 1 | 1 |
| 313 | Consider dosage modification | 1 | 1 | 1 | 1 | 1 | 1 | 1 | 1 | 1 | 1 | 1 | 1 | 1 | 1 | 1 | 1 | 1 | 1 | 1 | 1 |
| 314 | Change medication | 1 | 1 | 1 | 1 | 1 | 1 | 1 | 1 | 1 | 1 | 0 | 1 | 1 | 1 | 1 | 1 | 1 | 1 | 1 | 0 |
| 315 | No action needed | 1 | 1 | 1 | 1 | 1 | 1 | 1 | 1 | 1 | 1 | 1 | 1 | 1 | 1 | 1 | 1 | 1 | 1 | 1 | 1 |
| 316 | Change medication | 1 | 1 | 1 | 1 | 1 | 1 | 0 | 1 | 1 | 1 | 1 | 1 | 1 | 1 | 1 | 1 | 1 | 1 | 1 | 1 |
| 317 | Consider dosage modification | 1 | 1 | 1 | 1 | 1 | 1 | 1 | 1 | 1 | 1 | 1 | 1 | 1 | 1 | 1 | 1 | 1 | 1 | 1 | 1 |
| 318 | Change medication | 1 | 1 | 1 | 1 | 1 | 1 | 1 | 1 | 1 | 1 | 1 | 1 | 1 | 1 | 1 | 1 | 1 | 1 | 1 | 1 |
| 319 | Change medication | 1 | 1 | 1 | 1 | 1 | 1 | 0 | 1 | 1 | 1 | 0 | 1 | 1 | 1 | 1 | 1 | 1 | 1 | 1 | 1 |
| 320 | No action needed | 1 | 1 | 1 | 1 | 1 | 1 | 1 | 1 | 1 | 1 | 1 | 1 | 1 | 1 | 1 | 1 | 1 | 1 | 1 | 1 |
| 321 | No action needed | 1 | 1 | 1 | 1 | 1 | 1 | 1 | 1 | 1 | 1 | 1 | 1 | 1 | 1 | 1 | 1 | 1 | 1 | 1 | 1 |
| 322 | No action needed | 1 | 1 | 1 | 1 | 1 | 1 | 1 | 1 | 1 | 1 | 1 | 1 | 1 | 1 | 1 | 1 | 1 | 1 | 1 | 1 |
| 323 | No action needed | 1 | 1 | 1 | 1 | 1 | 1 | 1 | 1 | 1 | 1 | 1 | 1 | 1 | 1 | 1 | 1 | 1 | 1 | 1 | 1 |
| 324 | No action needed | 1 | 1 | 1 | 1 | 1 | 1 | 1 | 1 | 1 | 1 | 1 | 1 | 1 | 1 | 1 | 1 | 1 | 1 | 1 | 1 |
| 325 | No action needed | 1 | 1 | 1 | 1 | 1 | 1 | 1 | 1 | 1 | 1 | 1 | 1 | 1 | 1 | 1 | 1 | 1 | 1 | 1 | 1 |
| 326 | No action needed | 1 | 1 | 1 | 1 | 1 | 1 | 1 | 1 | 1 | 1 | 1 | 1 | 1 | 1 | 1 | 1 | 1 | 1 | 1 | 1 |
| 327 | No action needed | 1 | 1 | 1 | 1 | 1 | 1 | 1 | 1 | 1 | 1 | 1 | 1 | 1 | 1 | 1 | 1 | 1 | 1 | 1 | 1 |
| 328 | No action needed. | 1 | 1 | 1 | 1 | 1 | 1 | 1 | 1 | 1 | 1 | 1 | 1 | 1 | 1 | 1 | 1 | 1 | 1 | 1 | 1 |
| 329 | No action needed | 1 | 1 | 1 | 1 | 1 | 1 | 1 | 1 | 1 | 1 | 1 | 1 | 1 | 1 | 1 | 1 | 1 | 1 | 1 | 1 |
| 330 | Consider dosage modification | 1 | 1 | 1 | 1 | 1 | 1 | 1 | 1 | 1 | 1 | 1 | 1 | 1 | 1 | 1 | 1 | 1 | 1 | 1 | 1 |
| 331 | Consider dosage modification | 1 | 1 | 1 | 1 | 1 | 1 | 1 | 1 | 1 | 1 | 1 | 1 | 1 | 1 | 1 | 1 | 1 | 1 | 1 | 1 |
| 332 | Consider dosage modification | 1 | 1 | 1 | 1 | 1 | 1 | 1 | 1 | 1 | 1 | 1 | 1 | 1 | 1 | 1 | 1 | 1 | 1 | 1 | 1 |
| 333 | Consider dosage modification | 1 | 1 | 1 | 1 | 1 | 1 | 1 | 1 | 1 | 1 | 1 | 1 | 1 | 1 | 1 | 1 | 1 | 1 | 1 | 1 |
| 334 | Change medication | 1 | 1 | 1 | 1 | 1 | 1 | 1 | 1 | 1 | 1 | 1 | 1 | 1 | 1 | 1 | 1 | 1 | 1 | 1 | 1 |
| 335 | Consider dosage modification | 1 | 1 | 1 | 1 | 1 | 1 | 1 | 1 | 1 | 1 | 1 | 1 | 1 | 1 | 1 | 1 | 1 | 1 | 1 | 1 |
| 336 | Consider dosage modification | 1 | 1 | 1 | 1 | 1 | 1 | 1 | 1 | 1 | 1 | 1 | 1 | 1 | 1 | 1 | 1 | 1 | 1 | 1 | 1 |
| 337 | Consider dosage modification | 1 | 1 | 1 | 1 | 1 | 1 | 1 | 1 | 1 | 1 | 1 | 1 | 1 | 1 | 1 | 1 | 1 | 1 | 1 | 1 |
| 338 | No action needed | 1 | 1 | 1 | 1 | 1 | 1 | 1 | 1 | 1 | 1 | 1 | 1 | 1 | 1 | 1 | 1 | 1 | 1 | 1 | 1 |
| 339 | No action needed | 1 | 1 | 1 | 1 | 1 | 1 | 1 | 1 | 1 | 1 | 1 | 1 | 1 | 1 | 1 | 1 | 1 | 1 | 1 | 1 |
| 340 | No action needed | 1 | 1 | 1 | 1 | 1 | 1 | 1 | 1 | 1 | 1 | 1 | 1 | 1 | 1 | 1 | 1 | 1 | 1 | 1 | 1 |
| 341 | No action needed | 1 | 1 | 1 | 1 | 1 | 1 | 1 | 1 | 1 | 1 | 1 | 1 | 1 | 1 | 1 | 1 | 1 | 1 | 1 | 1 |
| 342 | Consider dosage modification | 1 | 1 | 1 | 1 | 1 | 1 | 1 | 1 | 1 | 1 | 1 | 1 | 1 | 1 | 1 | 1 | 1 | 1 | 1 | 1 |
| 343 | No action needed | 1 | 1 | 1 | 1 | 1 | 1 | 1 | 1 | 1 | 1 | 1 | 1 | 1 | 1 | 1 | 1 | 1 | 1 | 1 | 1 |
| 344 | No action needed | 1 | 1 | 1 | 1 | 1 | 1 | 1 | 1 | 1 | 1 | 1 | 1 | 1 | 1 | 1 | 1 | 1 | 1 | 1 | 1 |
| 345 | Consider dosage modification | 1 | 1 | 1 | 1 | 1 | 1 | 1 | 1 | 1 | 1 | 1 | 1 | 1 | 1 | 1 | 1 | 1 | 1 | 1 | 1 |
| 346 | No action needed | 1 | 1 | 1 | 1 | 1 | 1 | 1 | 1 | 1 | 1 | 1 | 1 | 1 | 1 | 1 | 1 | 1 | 1 | 1 | 1 |
| 347 | No action needed | 1 | 1 | 1 | 1 | 1 | 1 | 1 | 1 | 1 | 1 | 1 | 1 | 1 | 1 | 1 | 1 | 1 | 1 | 1 | 1 |
| 348 | Consider dosage modification | 1 | 1 | 1 | 1 | 1 | 1 | 1 | 1 | 1 | 1 | 1 | 1 | 1 | 1 | 1 | 1 | 1 | 1 | 1 | 1 |
| 349 | Change medication | 1 | 1 | 1 | 1 | 1 | 1 | 1 | 1 | 1 | 1 | 1 | 1 | 1 | 1 | 1 | 1 | 1 | 1 | 1 | 1 |
| 350 | No action needed | 1 | 1 | 1 | 1 | 1 | 1 | 1 | 1 | 1 | 1 | 1 | 1 | 1 | 1 | 1 | 1 | 1 | 1 | 1 | 1 |
| 351 | No action needed | 1 | 1 | 1 | 1 | 1 | 1 | 1 | 1 | 1 | 1 | 1 | 1 | 1 | 1 | 1 | 1 | 1 | 1 | 1 | 1 |
| 352 | Consider dosage modification | 1 | 1 | 1 | 1 | 1 | 1 | 1 | 1 | 1 | 1 | 1 | 1 | 1 | 1 | 1 | 1 | 1 | 1 | 1 | 1 |
| 353 | Change medication | 1 | 1 | 1 | 1 | 1 | 1 | 1 | 1 | 1 | 1 | 1 | 1 | 1 | 1 | 1 | 1 | 1 | 1 | 1 | 1 |
| 354 | No action needed | 1 | 1 | 1 | 1 | 1 | 1 | 1 | 1 | 1 | 1 | 1 | 1 | 1 | 1 | 1 | 1 | 1 | 1 | 1 | 1 |
| 355 | Consider dosage modification | 1 | 1 | 1 | 1 | 1 | 1 | 1 | 1 | 1 | 1 | 1 | 1 | 1 | 1 | 1 | 1 | 1 | 1 | 1 | 1 |
| 356 | Consider dosage modification | 1 | 1 | 1 | 1 | 1 | 1 | 1 | 1 | 1 | 1 | 1 | 1 | 1 | 1 | 1 | 1 | 1 | 1 | 1 | 1 |
| 357 | Change medication | 1 | 1 | 1 | 1 | 1 | 1 | 1 | 1 | 1 | 1 | 1 | 1 | 1 | 1 | 1 | 1 | 1 | 1 | 1 | 1 |
| 358 | Consider dosage modification | 1 | 1 | 1 | 1 | 1 | 1 | 1 | 1 | 1 | 1 | 1 | 1 | 1 | 1 | 1 | 1 | 1 | 1 | 1 | 1 |
| 359 | No action needed | 1 | 1 | 1 | 1 | 1 | 1 | 1 | 1 | 1 | 1 | 1 | 1 | 1 | 1 | 1 | 1 | 1 | 1 | 1 | 1 |
| 360 | Consider dosage modification | 1 | 1 | 1 | 1 | 1 | 0 | 1 | 1 | 1 | 0 | 1 | 1 | 0 | 0 | 1 | 1 | 1 | 1 | 1 | 1 |
| 361 | No action needed | 1 | 1 | 1 | 1 | 1 | 1 | 1 | 1 | 1 | 1 | 1 | 1 | 1 | 1 | 1 | 1 | 1 | 1 | 1 | 1 |
| 362 | Change medication | 1 | 1 | 1 | 1 | 1 | 1 | 1 | 1 | 1 | 1 | 1 | 1 | 1 | 0 | 1 | 1 | 1 | 1 | 1 | 1 |
| 363 | No action needed | 1 | 1 | 1 | 1 | 1 | 1 | 1 | 1 | 1 | 1 | 1 | 1 | 1 | 1 | 1 | 1 | 1 | 1 | 1 | 1 |
| 364 | No action needed | 1 | 1 | 1 | 1 | 1 | 1 | 1 | 1 | 1 | 1 | 1 | 1 | 1 | 1 | 1 | 1 | 1 | 1 | 1 | 1 |
| 365 | No action needed | 1 | 1 | 1 | 1 | 1 | 1 | 1 | 1 | 1 | 1 | 1 | 1 | 1 | 1 | 1 | 1 | 1 | 1 | 1 | 1 |
| 366 | Change medication | 0 | 0 | 1 | 1 | 1 | 0 | 0 | 1 | 1 | 1 | 0 | 0 | 1 | 1 | 0 | 1 | 1 | 1 | 0 | 1 |
| 367 | Consider dosage modification | 1 | 1 | 1 | 1 | 1 | 1 | 1 | 1 | 1 | 1 | 1 | 1 | 1 | 1 | 1 | 1 | 1 | 1 | 1 | 1 |
| 368 | No action needed | 1 | 1 | 1 | 1 | 1 | 1 | 1 | 1 | 1 | 1 | 1 | 1 | 1 | 1 | 1 | 1 | 1 | 1 | 1 | 1 |
| 369 | Change medication | 1 | 1 | 1 | 1 | 1 | 1 | 1 | 1 | 1 | 1 | 1 | 1 | 1 | 1 | 1 | 1 | 1 | 1 | 1 | 1 |
| 370 | Consider dosage modification | 1 | 1 | 1 | 1 | 1 | 1 | 1 | 1 | 1 | 1 | 1 | 1 | 1 | 1 | 1 | 1 | 1 | 1 | 1 | 1 |
| 371 | Consider dosage modification | 1 | 1 | 1 | 1 | 1 | 1 | 1 | 1 | 1 | 1 | 1 | 1 | 1 | 1 | 1 | 1 | 1 | 1 | 1 | 1 |
| 372 | No action needed | 0 | 0 | 1 | 0 | 1 | 1 | 1 | 1 | 1 | 1 | 1 | 0 | 0 | 0 | 1 | 1 | 0 | 0 | 1 | 0 |
| 373 | Consider dosage modification | 1 | 1 | 1 | 1 | 1 | 1 | 1 | 1 | 1 | 1 | 1 | 1 | 1 | 1 | 1 | 1 | 1 | 1 | 1 | 1 |
| 374 | Change medication | 1 | 1 | 1 | 1 | 1 | 1 | 1 | 1 | 1 | 1 | 1 | 1 | 1 | 1 | 1 | 1 | 1 | 1 | 1 | 1 |
| 375 | Consider dosage modification | 1 | 1 | 1 | 1 | 1 | 1 | 1 | 1 | 1 | 1 | 1 | 1 | 1 | 1 | 1 | 1 | 1 | 1 | 1 | 1 |
| 376 | Consider dosage modification | 1 | 1 | 1 | 1 | 1 | 1 | 1 | 1 | 1 | 1 | 1 | 1 | 1 | 1 | 1 | 1 | 1 | 1 | 1 | 1 |
| 377 | No action needed | 1 | 1 | 1 | 1 | 1 | 1 | 1 | 1 | 1 | 1 | 1 | 1 | 1 | 1 | 1 | 1 | 1 | 1 | 1 | 1 |
| 378 | No action needed. | 1 | 1 | 1 | 1 | 1 | 1 | 1 | 1 | 1 | 1 | 1 | 1 | 1 | 1 | 1 | 1 | 1 | 1 | 1 | 1 |
| 379 | Consider dosage modification | 1 | 0 | 1 | 1 | 1 | 1 | 1 | 1 | 1 | 1 | 0 | 1 | 1 | 1 | 1 | 1 | 1 | 1 | 0 | 1 |
| 380 | Consider dosage modification | 1 | 1 | 1 | 1 | 1 | 0 | 1 | 1 | 0 | 1 | 1 | 1 | 1 | 1 | 1 | 1 | 1 | 1 | 1 | 1 |
| 381 | Consider dosage modification | 1 | 1 | 1 | 1 | 1 | 1 | 1 | 1 | 1 | 1 | 1 | 1 | 1 | 1 | 1 | 1 | 1 | 1 | 1 | 1 |
| 382 | Consider dosage modification | 1 | 1 | 1 | 1 | 1 | 1 | 1 | 1 | 1 | 1 | 1 | 1 | 1 | 1 | 1 | 1 | 1 | 1 | 1 | 1 |
| 383 | Consider dosage modification | 1 | 1 | 1 | 1 | 1 | 1 | 1 | 1 | 1 | 1 | 1 | 1 | 1 | 1 | 1 | 1 | 1 | 1 | 1 | 1 |
| 384 | Consider dosage modification | 1 | 1 | 1 | 1 | 1 | 1 | 1 | 1 | 1 | 1 | 1 | 1 | 1 | 1 | 1 | 1 | 1 | 1 | 1 | 1 |
| 385 | Change medication | 1 | 1 | 1 | 1 | 1 | 1 | 1 | 1 | 1 | 1 | 1 | 1 | 1 | 1 | 1 | 1 | 1 | 1 | 1 | 1 |

1. Base Value: The most frequent classification result observed across 20 tests.
2. For GPT4o, classifications that agreed with base value data were coded as 1, while disagreements were coded as 0.

**Table S3. Prediction Stability of Gemini 1.5-Pro Across 20 Iterations**

| **No.** | **Base value ^a^** | **Individual results of 20 predictions by** **Germini 1.5-Pro ^b^** | | | | | | | | | | | | | | | | | | | |
| --- | --- | --- | --- | --- | --- | --- | --- | --- | --- | --- | --- | --- | --- | --- | --- | --- | --- | --- | --- | --- | --- |
|  |  | **1** | **2** | **3** | **4** | **5** | **6** | **7** | **8** | **9** | **10** | **11** | **12** | **13** | **14** | **15** | **16** | **17** | **18** | **19** | **20** |
| 1 | No action needed | 1 | 1 | 1 | 1 | 1 | 1 | 1 | 1 | 1 | 1 | 1 | 1 | 1 | 1 | 1 | 1 | 1 | 1 | 1 | 1 |
| 2 | No action needed | 1 | 1 | 1 | 1 | 1 | 1 | 1 | 1 | 1 | 1 | 1 | 1 | 1 | 1 | 1 | 1 | 1 | 1 | 1 | 1 |
| 3 | No action needed | 1 | 1 | 1 | 1 | 1 | 1 | 1 | 1 | 1 | 1 | 1 | 1 | 1 | 1 | 1 | 1 | 1 | 1 | 1 | 1 |
| 4 | No action needed | 1 | 1 | 1 | 1 | 1 | 1 | 1 | 1 | 1 | 1 | 1 | 1 | 1 | 1 | 1 | 1 | 1 | 1 | 1 | 1 |
| 5 | No action needed | 1 | 1 | 1 | 1 | 1 | 1 | 1 | 1 | 1 | 1 | 1 | 1 | 1 | 1 | 1 | 1 | 1 | 1 | 1 | 1 |
| 6 | No action needed | 1 | 1 | 1 | 1 | 1 | 1 | 1 | 1 | 1 | 1 | 1 | 1 | 1 | 1 | 1 | 1 | 1 | 1 | 1 | 1 |
| 7 | No action needed | 1 | 1 | 1 | 1 | 1 | 1 | 1 | 1 | 1 | 1 | 1 | 1 | 1 | 1 | 1 | 1 | 1 | 1 | 1 | 1 |
| 8 | No action needed | 1 | 1 | 1 | 1 | 1 | 1 | 1 | 1 | 1 | 1 | 1 | 1 | 1 | 1 | 1 | 1 | 1 | 1 | 1 | 1 |
| 9 | No action needed | 1 | 1 | 1 | 1 | 1 | 1 | 1 | 1 | 1 | 1 | 1 | 1 | 1 | 1 | 1 | 1 | 1 | 1 | 1 | 1 |
| 10 | No action needed | 1 | 1 | 1 | 1 | 1 | 1 | 1 | 1 | 1 | 1 | 1 | 1 | 1 | 1 | 1 | 1 | 1 | 1 | 1 | 1 |
| 11 | No action needed | 1 | 1 | 1 | 1 | 1 | 1 | 1 | 1 | 1 | 1 | 1 | 1 | 1 | 1 | 1 | 1 | 1 | 1 | 1 | 1 |
| 12 | No action needed | 1 | 1 | 1 | 1 | 1 | 1 | 1 | 1 | 1 | 1 | 1 | 1 | 1 | 1 | 1 | 1 | 1 | 1 | 1 | 1 |
| 13 | No action needed | 1 | 1 | 1 | 1 | 1 | 1 | 1 | 1 | 1 | 1 | 1 | 1 | 1 | 1 | 1 | 1 | 1 | 1 | 1 | 1 |
| 14 | No action needed | 1 | 1 | 1 | 1 | 1 | 1 | 1 | 1 | 1 | 1 | 1 | 1 | 1 | 1 | 1 | 1 | 1 | 1 | 1 | 1 |
| 15 | No action needed | 1 | 1 | 1 | 1 | 1 | 1 | 1 | 1 | 1 | 1 | 1 | 1 | 1 | 1 | 1 | 1 | 1 | 1 | 1 | 1 |
| 16 | No action needed | 1 | 1 | 1 | 1 | 1 | 1 | 1 | 1 | 1 | 1 | 1 | 1 | 1 | 1 | 1 | 1 | 1 | 1 | 1 | 1 |
| 17 | No action needed | 1 | 1 | 1 | 1 | 1 | 1 | 1 | 1 | 1 | 1 | 1 | 1 | 1 | 1 | 1 | 1 | 1 | 1 | 1 | 1 |
| 18 | No action needed | 1 | 1 | 1 | 1 | 1 | 1 | 1 | 1 | 1 | 1 | 1 | 1 | 1 | 1 | 1 | 1 | 1 | 1 | 1 | 1 |
| 19 | No action needed | 1 | 1 | 1 | 1 | 1 | 1 | 1 | 1 | 1 | 1 | 1 | 1 | 1 | 1 | 1 | 1 | 1 | 1 | 1 | 1 |
| 20 | No action needed | 1 | 1 | 1 | 1 | 1 | 1 | 1 | 1 | 1 | 1 | 1 | 1 | 1 | 1 | 1 | 1 | 1 | 1 | 1 | 1 |
| 21 | No action needed | 1 | 1 | 1 | 1 | 1 | 1 | 1 | 1 | 1 | 1 | 1 | 1 | 1 | 1 | 1 | 1 | 1 | 1 | 1 | 1 |
| 22 | No action needed | 1 | 1 | 1 | 1 | 1 | 1 | 1 | 1 | 1 | 1 | 1 | 1 | 1 | 1 | 1 | 1 | 1 | 1 | 1 | 1 |
| 23 | No action needed | 1 | 1 | 1 | 1 | 1 | 1 | 1 | 1 | 1 | 1 | 1 | 1 | 1 | 1 | 1 | 1 | 1 | 1 | 1 | 1 |
| 24 | No action needed | 1 | 1 | 1 | 1 | 1 | 1 | 1 | 1 | 1 | 1 | 1 | 1 | 1 | 1 | 1 | 1 | 1 | 1 | 1 | 1 |
| 25 | Change medication | 1 | 1 | 1 | 1 | 1 | 1 | 1 | 1 | 1 | 1 | 1 | 1 | 1 | 1 | 1 | 1 | 1 | 1 | 1 | 1 |
| 26 | Change medication | 1 | 1 | 1 | 1 | 1 | 1 | 1 | 1 | 1 | 1 | 1 | 1 | 1 | 1 | 1 | 1 | 1 | 1 | 1 | 1 |
| 27 | Change medication | 1 | 1 | 1 | 1 | 1 | 1 | 1 | 1 | 1 | 1 | 1 | 1 | 1 | 1 | 1 | 1 | 1 | 1 | 1 | 1 |
| 28 | Change medication | 1 | 1 | 1 | 1 | 1 | 1 | 1 | 1 | 1 | 1 | 1 | 1 | 1 | 1 | 1 | 1 | 1 | 1 | 1 | 1 |
| 29 | Consider dosage modification | 1 | 1 | 1 | 1 | 1 | 1 | 1 | 1 | 1 | 1 | 1 | 1 | 1 | 1 | 1 | 1 | 1 | 1 | 1 | 1 |
| 30 | Consider dosage modification | 1 | 1 | 1 | 1 | 1 | 1 | 1 | 1 | 1 | 1 | 1 | 1 | 1 | 1 | 1 | 1 | 1 | 1 | 1 | 1 |
| 31 | Change medication | 1 | 1 | 1 | 1 | 1 | 1 | 1 | 1 | 1 | 1 | 1 | 1 | 1 | 1 | 1 | 1 | 1 | 1 | 1 | 1 |
| 32 | Change medication | 1 | 1 | 1 | 1 | 1 | 1 | 1 | 1 | 1 | 1 | 1 | 1 | 1 | 1 | 1 | 1 | 1 | 1 | 1 | 1 |
| 33 | Change medication | 1 | 1 | 1 | 1 | 1 | 1 | 1 | 1 | 1 | 1 | 1 | 1 | 1 | 1 | 1 | 1 | 1 | 1 | 1 | 1 |
| 34 | Change medication | 1 | 1 | 1 | 1 | 1 | 1 | 1 | 1 | 1 | 1 | 1 | 1 | 1 | 1 | 1 | 1 | 1 | 1 | 1 | 1 |
| 35 | Change medication | 1 | 1 | 1 | 1 | 1 | 1 | 1 | 1 | 1 | 1 | 1 | 1 | 1 | 1 | 1 | 1 | 1 | 1 | 1 | 1 |
| 36 | Change medication | 1 | 1 | 1 | 1 | 1 | 1 | 1 | 1 | 1 | 1 | 1 | 1 | 1 | 1 | 1 | 1 | 1 | 1 | 1 | 1 |
| 37 | Change medication | 1 | 1 | 1 | 1 | 1 | 1 | 1 | 1 | 1 | 1 | 1 | 1 | 1 | 1 | 1 | 1 | 1 | 1 | 1 | 1 |
| 38 | Change medication | 1 | 1 | 1 | 1 | 1 | 1 | 1 | 1 | 1 | 1 | 1 | 1 | 1 | 1 | 1 | 1 | 1 | 1 | 1 | 1 |
| 39 | Change medication | 1 | 1 | 1 | 1 | 1 | 1 | 1 | 1 | 1 | 1 | 1 | 1 | 1 | 1 | 1 | 1 | 1 | 1 | 1 | 1 |
| 40 | Change medication | 1 | 1 | 1 | 1 | 1 | 1 | 1 | 1 | 1 | 1 | 1 | 1 | 1 | 1 | 1 | 1 | 1 | 1 | 1 | 1 |
| 41 | Change medication | 1 | 1 | 1 | 1 | 1 | 1 | 1 | 1 | 1 | 1 | 1 | 1 | 1 | 1 | 1 | 1 | 1 | 1 | 1 | 1 |
| 42 | Change medication | 1 | 1 | 1 | 1 | 1 | 1 | 1 | 1 | 1 | 1 | 1 | 1 | 1 | 1 | 1 | 1 | 1 | 1 | 1 | 1 |
| 43 | Consider dosage modification | 1 | 1 | 1 | 1 | 1 | 1 | 1 | 1 | 1 | 1 | 1 | 1 | 1 | 1 | 1 | 1 | 1 | 1 | 1 | 1 |
| 44 | Consider dosage modification | 1 | 1 | 1 | 1 | 1 | 1 | 1 | 1 | 1 | 1 | 1 | 1 | 1 | 1 | 1 | 1 | 1 | 1 | 1 | 1 |
| 45 | No action needed | 1 | 1 | 1 | 1 | 1 | 1 | 1 | 1 | 1 | 1 | 1 | 1 | 1 | 1 | 1 | 1 | 1 | 1 | 1 | 1 |
| 46 | No action needed | 1 | 1 | 1 | 1 | 1 | 1 | 1 | 1 | 1 | 1 | 1 | 1 | 1 | 1 | 1 | 1 | 1 | 1 | 1 | 1 |
| 47 | No action needed | 1 | 1 | 1 | 1 | 1 | 1 | 1 | 1 | 1 | 1 | 1 | 1 | 1 | 1 | 1 | 1 | 1 | 1 | 1 | 1 |
| 48 | No action needed | 1 | 1 | 1 | 1 | 1 | 1 | 1 | 1 | 1 | 1 | 1 | 1 | 1 | 1 | 1 | 1 | 1 | 1 | 1 | 1 |
| 49 | No action needed | 1 | 1 | 1 | 1 | 1 | 1 | 1 | 1 | 1 | 1 | 1 | 1 | 1 | 1 | 1 | 1 | 1 | 1 | 1 | 1 |
| 50 | No action needed | 1 | 1 | 1 | 1 | 1 | 1 | 1 | 1 | 1 | 1 | 1 | 1 | 1 | 1 | 1 | 1 | 1 | 1 | 1 | 1 |
| 51 | No action needed | 1 | 1 | 1 | 1 | 1 | 1 | 1 | 1 | 1 | 1 | 1 | 1 | 1 | 1 | 1 | 1 | 1 | 1 | 1 | 1 |
| 52 | No action needed | 1 | 1 | 1 | 1 | 1 | 1 | 1 | 1 | 1 | 1 | 1 | 1 | 1 | 1 | 1 | 1 | 1 | 1 | 1 | 1 |
| 53 | No action needed | 1 | 1 | 1 | 1 | 1 | 1 | 1 | 1 | 1 | 1 | 1 | 1 | 1 | 1 | 1 | 1 | 1 | 1 | 1 | 1 |
| 54 | No action needed | 1 | 1 | 1 | 1 | 1 | 1 | 1 | 1 | 1 | 1 | 1 | 1 | 1 | 1 | 1 | 1 | 1 | 1 | 1 | 1 |
| 55 | No action needed | 1 | 1 | 1 | 1 | 1 | 1 | 1 | 1 | 1 | 1 | 1 | 1 | 1 | 1 | 1 | 1 | 1 | 1 | 1 | 1 |
| 56 | No action needed | 1 | 1 | 1 | 1 | 1 | 1 | 1 | 1 | 1 | 1 | 1 | 1 | 1 | 1 | 1 | 1 | 1 | 1 | 1 | 1 |
| 57 | No action needed | 1 | 1 | 1 | 1 | 1 | 1 | 1 | 1 | 1 | 1 | 1 | 1 | 1 | 1 | 1 | 1 | 1 | 1 | 1 | 1 |
| 58 | No action needed | 1 | 1 | 1 | 1 | 1 | 1 | 1 | 1 | 1 | 1 | 1 | 1 | 1 | 1 | 1 | 1 | 1 | 1 | 1 | 1 |
| 59 | No action needed | 1 | 1 | 1 | 1 | 1 | 1 | 1 | 1 | 1 | 1 | 1 | 1 | 1 | 1 | 1 | 1 | 1 | 1 | 1 | 1 |
| 60 | No action needed | 1 | 1 | 1 | 1 | 1 | 1 | 1 | 1 | 1 | 1 | 1 | 1 | 1 | 1 | 1 | 1 | 1 | 1 | 1 | 1 |
| 61 | No action needed | 1 | 1 | 1 | 1 | 1 | 1 | 1 | 1 | 1 | 1 | 1 | 1 | 1 | 1 | 1 | 1 | 1 | 1 | 1 | 1 |
| 62 | No action needed | 1 | 1 | 1 | 1 | 1 | 1 | 1 | 1 | 1 | 1 | 1 | 1 | 1 | 1 | 1 | 1 | 1 | 1 | 1 | 1 |
| 63 | No action needed | 1 | 1 | 1 | 1 | 1 | 1 | 1 | 1 | 1 | 1 | 1 | 1 | 1 | 1 | 1 | 1 | 1 | 1 | 1 | 1 |
| 64 | No action needed | 1 | 1 | 1 | 1 | 1 | 1 | 1 | 1 | 1 | 1 | 1 | 1 | 1 | 1 | 1 | 1 | 1 | 1 | 1 | 1 |
| 65 | No action needed | 1 | 1 | 1 | 1 | 1 | 1 | 1 | 1 | 1 | 1 | 1 | 1 | 1 | 1 | 1 | 1 | 1 | 1 | 1 | 1 |
| 66 | No action needed | 1 | 1 | 1 | 1 | 1 | 1 | 1 | 1 | 1 | 1 | 1 | 1 | 1 | 1 | 1 | 1 | 1 | 1 | 1 | 1 |
| 67 | No action needed | 1 | 1 | 1 | 1 | 1 | 1 | 1 | 1 | 1 | 1 | 1 | 1 | 1 | 1 | 1 | 1 | 1 | 1 | 1 | 1 |
| 68 | No action needed | 1 | 1 | 1 | 1 | 1 | 1 | 1 | 1 | 1 | 1 | 1 | 1 | 1 | 1 | 1 | 1 | 1 | 1 | 1 | 1 |
| 69 | No action needed | 1 | 1 | 1 | 1 | 1 | 1 | 1 | 1 | 1 | 1 | 1 | 1 | 1 | 1 | 1 | 1 | 1 | 1 | 1 | 1 |
| 70 | No action needed | 1 | 1 | 1 | 1 | 1 | 1 | 1 | 1 | 1 | 1 | 1 | 1 | 1 | 1 | 1 | 1 | 1 | 1 | 1 | 1 |
| 71 | No action needed | 1 | 1 | 1 | 1 | 1 | 1 | 1 | 1 | 1 | 1 | 1 | 1 | 1 | 1 | 1 | 1 | 1 | 1 | 1 | 1 |
| 72 | No action needed | 1 | 1 | 1 | 1 | 1 | 1 | 1 | 1 | 1 | 1 | 1 | 1 | 1 | 1 | 1 | 1 | 1 | 1 | 1 | 1 |
| 73 | No action needed | 1 | 1 | 1 | 1 | 1 | 1 | 1 | 1 | 1 | 1 | 1 | 1 | 1 | 1 | 1 | 1 | 1 | 1 | 1 | 1 |
| 74 | No action needed | 1 | 1 | 1 | 1 | 1 | 1 | 1 | 1 | 1 | 1 | 1 | 1 | 1 | 1 | 1 | 1 | 1 | 1 | 1 | 1 |
| 75 | No action needed | 1 | 1 | 1 | 1 | 1 | 1 | 1 | 1 | 1 | 1 | 1 | 1 | 1 | 1 | 1 | 1 | 1 | 1 | 1 | 1 |
| 76 | No action needed | 1 | 1 | 1 | 1 | 1 | 1 | 1 | 1 | 1 | 1 | 1 | 1 | 1 | 1 | 1 | 1 | 1 | 1 | 1 | 1 |
| 77 | No action needed | 1 | 1 | 1 | 1 | 1 | 1 | 1 | 1 | 1 | 1 | 1 | 1 | 1 | 1 | 1 | 1 | 1 | 1 | 1 | 1 |
| 78 | No action needed | 1 | 1 | 1 | 1 | 1 | 1 | 1 | 1 | 1 | 1 | 1 | 1 | 1 | 1 | 1 | 1 | 1 | 1 | 1 | 1 |
| 79 | No action needed | 1 | 1 | 1 | 1 | 1 | 1 | 1 | 1 | 1 | 1 | 1 | 1 | 1 | 1 | 1 | 1 | 1 | 1 | 1 | 1 |
| 80 | No action needed | 1 | 1 | 1 | 1 | 1 | 1 | 1 | 1 | 1 | 1 | 1 | 1 | 1 | 1 | 1 | 1 | 1 | 1 | 1 | 1 |
| 81 | No action needed | 1 | 1 | 1 | 1 | 1 | 1 | 1 | 1 | 1 | 1 | 1 | 1 | 1 | 1 | 1 | 1 | 1 | 1 | 1 | 1 |
| 82 | No action needed | 1 | 1 | 1 | 1 | 1 | 1 | 1 | 1 | 1 | 1 | 1 | 1 | 1 | 1 | 1 | 1 | 1 | 1 | 1 | 1 |
| 83 | No action needed | 1 | 1 | 1 | 1 | 1 | 1 | 1 | 1 | 1 | 1 | 1 | 1 | 1 | 1 | 1 | 1 | 1 | 1 | 1 | 1 |
| 84 | No action needed | 1 | 1 | 1 | 1 | 1 | 1 | 1 | 1 | 1 | 1 | 1 | 1 | 1 | 1 | 1 | 1 | 1 | 1 | 1 | 1 |
| 85 | No action needed | 1 | 1 | 1 | 1 | 1 | 1 | 1 | 1 | 1 | 1 | 1 | 1 | 1 | 1 | 1 | 1 | 1 | 1 | 1 | 1 |
| 86 | No action needed | 1 | 1 | 1 | 1 | 1 | 1 | 1 | 1 | 1 | 1 | 1 | 1 | 1 | 1 | 1 | 1 | 1 | 1 | 1 | 1 |
| 87 | No action needed | 1 | 1 | 1 | 1 | 1 | 1 | 1 | 1 | 1 | 1 | 1 | 1 | 1 | 1 | 1 | 1 | 1 | 1 | 1 | 1 |
| 88 | No action needed | 1 | 1 | 1 | 1 | 1 | 1 | 1 | 1 | 1 | 1 | 1 | 1 | 1 | 1 | 1 | 1 | 1 | 1 | 1 | 1 |
| 89 | No action needed | 1 | 1 | 1 | 1 | 1 | 1 | 1 | 1 | 1 | 1 | 1 | 1 | 1 | 1 | 1 | 1 | 1 | 1 | 1 | 1 |
| 90 | No action needed | 1 | 1 | 1 | 1 | 1 | 1 | 1 | 1 | 1 | 1 | 1 | 1 | 1 | 1 | 1 | 1 | 1 | 1 | 1 | 1 |
| 91 | No action needed | 1 | 1 | 1 | 1 | 1 | 1 | 1 | 1 | 1 | 1 | 1 | 1 | 1 | 1 | 1 | 1 | 1 | 1 | 1 | 1 |
| 92 | No action needed | 1 | 1 | 1 | 1 | 1 | 1 | 1 | 1 | 1 | 1 | 1 | 1 | 1 | 1 | 1 | 1 | 1 | 1 | 1 | 1 |
| 93 | No action needed | 1 | 1 | 1 | 1 | 1 | 1 | 1 | 1 | 1 | 1 | 1 | 1 | 1 | 1 | 1 | 1 | 1 | 1 | 1 | 1 |
| 94 | No action needed | 1 | 1 | 1 | 1 | 1 | 1 | 1 | 1 | 1 | 1 | 1 | 1 | 1 | 1 | 1 | 1 | 1 | 1 | 1 | 1 |
| 95 | No action needed | 1 | 1 | 1 | 1 | 1 | 1 | 1 | 1 | 1 | 1 | 1 | 1 | 1 | 1 | 1 | 1 | 1 | 1 | 1 | 1 |
| 96 | No action needed | 1 | 1 | 1 | 1 | 1 | 1 | 1 | 1 | 1 | 1 | 1 | 1 | 1 | 1 | 1 | 1 | 1 | 1 | 1 | 1 |
| 97 | No action needed | 1 | 1 | 1 | 1 | 1 | 1 | 1 | 1 | 1 | 1 | 1 | 1 | 1 | 1 | 1 | 1 | 1 | 1 | 1 | 1 |
| 98 | No action needed | 1 | 1 | 1 | 1 | 1 | 1 | 1 | 1 | 1 | 1 | 1 | 1 | 1 | 1 | 1 | 1 | 1 | 1 | 1 | 1 |
| 99 | No action needed | 1 | 1 | 1 | 1 | 1 | 1 | 1 | 1 | 1 | 1 | 1 | 1 | 1 | 1 | 1 | 1 | 1 | 1 | 1 | 1 |
| 100 | No action needed | 1 | 1 | 1 | 1 | 1 | 1 | 1 | 1 | 1 | 1 | 1 | 1 | 1 | 1 | 1 | 1 | 1 | 1 | 1 | 1 |
| 101 | No action needed | 1 | 1 | 1 | 1 | 1 | 1 | 1 | 1 | 1 | 1 | 1 | 1 | 1 | 1 | 1 | 1 | 1 | 1 | 1 | 1 |
| 102 | No action needed | 1 | 1 | 1 | 1 | 1 | 1 | 1 | 1 | 1 | 1 | 1 | 1 | 1 | 1 | 1 | 1 | 1 | 1 | 1 | 1 |
| 103 | No action needed | 1 | 1 | 1 | 1 | 1 | 1 | 1 | 1 | 1 | 1 | 1 | 1 | 1 | 1 | 1 | 1 | 1 | 1 | 1 | 1 |
| 104 | No action needed | 1 | 1 | 1 | 1 | 1 | 1 | 1 | 1 | 1 | 1 | 1 | 1 | 1 | 1 | 1 | 1 | 1 | 1 | 1 | 1 |
| 105 | Change medication | 1 | 1 | 1 | 1 | 1 | 1 | 1 | 1 | 1 | 1 | 1 | 1 | 1 | 1 | 1 | 1 | 1 | 1 | 1 | 1 |
| 106 | Change medication | 1 | 1 | 1 | 1 | 1 | 1 | 1 | 1 | 1 | 1 | 1 | 1 | 1 | 1 | 1 | 1 | 1 | 1 | 1 | 1 |
| 107 | No action needed | 1 | 1 | 1 | 1 | 1 | 1 | 1 | 1 | 1 | 1 | 1 | 1 | 1 | 1 | 1 | 1 | 1 | 1 | 1 | 1 |
| 108 | No action needed | 1 | 1 | 1 | 1 | 1 | 1 | 1 | 1 | 1 | 1 | 1 | 1 | 1 | 1 | 1 | 1 | 1 | 1 | 1 | 1 |
| 109 | No action needed | 1 | 1 | 1 | 1 | 1 | 1 | 1 | 1 | 1 | 1 | 1 | 1 | 1 | 1 | 1 | 1 | 1 | 1 | 1 | 1 |
| 110 | No action needed | 1 | 1 | 1 | 1 | 1 | 1 | 1 | 1 | 1 | 1 | 1 | 1 | 1 | 1 | 1 | 1 | 1 | 1 | 1 | 1 |
| 111 | No action needed | 1 | 1 | 1 | 1 | 1 | 1 | 1 | 1 | 1 | 1 | 1 | 1 | 1 | 1 | 1 | 1 | 1 | 1 | 1 | 1 |
| 112 | No action needed | 1 | 1 | 1 | 1 | 1 | 1 | 1 | 1 | 1 | 1 | 1 | 1 | 1 | 1 | 1 | 1 | 1 | 1 | 1 | 1 |
| 113 | No action needed | 1 | 1 | 1 | 1 | 1 | 1 | 1 | 1 | 1 | 1 | 1 | 1 | 1 | 1 | 1 | 1 | 1 | 1 | 1 | 1 |
| 114 | No action needed | 1 | 1 | 1 | 1 | 1 | 1 | 1 | 1 | 1 | 1 | 1 | 1 | 1 | 1 | 1 | 1 | 1 | 1 | 1 | 1 |
| 115 | No action needed | 1 | 1 | 1 | 1 | 1 | 1 | 1 | 1 | 1 | 1 | 1 | 1 | 1 | 1 | 1 | 1 | 1 | 1 | 1 | 1 |
| 116 | No action needed | 1 | 1 | 1 | 1 | 1 | 1 | 1 | 1 | 1 | 1 | 1 | 1 | 1 | 1 | 1 | 1 | 1 | 1 | 1 | 1 |
| 117 | No action needed | 1 | 1 | 1 | 1 | 1 | 1 | 1 | 1 | 1 | 1 | 1 | 1 | 1 | 1 | 1 | 1 | 1 | 1 | 1 | 1 |
| 118 | No action needed | 1 | 1 | 1 | 1 | 1 | 1 | 1 | 1 | 1 | 1 | 1 | 1 | 1 | 1 | 1 | 1 | 1 | 1 | 1 | 1 |
| 119 | No action needed | 1 | 1 | 1 | 1 | 1 | 1 | 1 | 1 | 1 | 1 | 1 | 1 | 1 | 1 | 1 | 1 | 1 | 1 | 1 | 1 |
| 120 | No action needed | 1 | 1 | 1 | 1 | 1 | 1 | 1 | 1 | 1 | 1 | 1 | 1 | 1 | 1 | 1 | 1 | 1 | 1 | 1 | 1 |
| 121 | No action needed | 1 | 1 | 1 | 1 | 1 | 1 | 1 | 1 | 1 | 1 | 1 | 1 | 1 | 1 | 1 | 1 | 1 | 1 | 1 | 1 |
| 122 | No action needed | 1 | 1 | 1 | 1 | 1 | 1 | 1 | 1 | 1 | 1 | 1 | 1 | 1 | 1 | 1 | 1 | 1 | 1 | 1 | 1 |
| 123 | No action needed | 1 | 1 | 1 | 1 | 1 | 1 | 1 | 1 | 1 | 1 | 1 | 1 | 1 | 1 | 1 | 1 | 1 | 1 | 1 | 1 |
| 124 | No action needed | 1 | 1 | 1 | 1 | 1 | 1 | 1 | 1 | 1 | 1 | 1 | 1 | 1 | 1 | 1 | 1 | 1 | 1 | 1 | 1 |
| 125 | No action needed | 1 | 1 | 1 | 1 | 1 | 1 | 1 | 1 | 1 | 1 | 1 | 1 | 1 | 1 | 1 | 1 | 1 | 1 | 1 | 1 |
| 126 | Change medication | 1 | 1 | 1 | 1 | 1 | 1 | 1 | 1 | 1 | 1 | 1 | 1 | 1 | 1 | 1 | 1 | 1 | 1 | 1 | 1 |
| 127 | Change medication | 1 | 1 | 1 | 1 | 1 | 1 | 1 | 1 | 1 | 1 | 1 | 1 | 1 | 1 | 1 | 1 | 1 | 1 | 1 | 1 |
| 128 | No action needed | 1 | 1 | 1 | 1 | 1 | 1 | 1 | 1 | 1 | 1 | 1 | 1 | 1 | 1 | 1 | 1 | 1 | 1 | 1 | 1 |
| 129 | No action needed | 1 | 1 | 1 | 1 | 1 | 1 | 1 | 1 | 1 | 1 | 1 | 1 | 1 | 1 | 1 | 1 | 1 | 1 | 1 | 1 |
| 130 | No action needed | 1 | 1 | 1 | 1 | 1 | 1 | 1 | 1 | 1 | 1 | 1 | 1 | 1 | 1 | 1 | 1 | 1 | 1 | 1 | 1 |
| 131 | No action needed | 1 | 1 | 1 | 1 | 1 | 1 | 1 | 1 | 1 | 1 | 1 | 1 | 1 | 1 | 1 | 1 | 1 | 1 | 1 | 1 |
| 132 | No action needed | 1 | 1 | 1 | 1 | 1 | 1 | 1 | 1 | 1 | 1 | 1 | 1 | 1 | 1 | 1 | 1 | 1 | 1 | 1 | 1 |
| 133 | No action needed | 1 | 1 | 1 | 1 | 1 | 1 | 1 | 1 | 1 | 1 | 1 | 1 | 1 | 1 | 1 | 1 | 1 | 1 | 1 | 1 |
| 134 | No action needed | 1 | 1 | 1 | 1 | 1 | 1 | 1 | 1 | 1 | 1 | 1 | 1 | 1 | 1 | 1 | 1 | 1 | 1 | 1 | 1 |
| 135 | No action needed | 1 | 1 | 1 | 1 | 1 | 1 | 1 | 1 | 1 | 1 | 1 | 1 | 1 | 1 | 1 | 1 | 1 | 1 | 1 | 1 |
| 136 | No action needed | 1 | 1 | 1 | 1 | 1 | 1 | 1 | 1 | 1 | 1 | 1 | 1 | 1 | 1 | 1 | 1 | 1 | 1 | 1 | 1 |
| 137 | No action needed | 1 | 1 | 1 | 1 | 1 | 1 | 1 | 1 | 1 | 1 | 1 | 1 | 1 | 1 | 1 | 1 | 1 | 1 | 1 | 1 |
| 138 | No action needed | 1 | 1 | 1 | 1 | 1 | 1 | 1 | 1 | 1 | 1 | 1 | 1 | 1 | 1 | 1 | 1 | 1 | 1 | 1 | 1 |
| 139 | No action needed | 1 | 1 | 1 | 1 | 1 | 1 | 1 | 1 | 1 | 1 | 1 | 1 | 1 | 1 | 1 | 1 | 1 | 1 | 1 | 1 |
| 140 | No action needed | 1 | 1 | 1 | 1 | 1 | 1 | 1 | 1 | 1 | 1 | 1 | 1 | 1 | 1 | 1 | 1 | 1 | 1 | 1 | 1 |
| 141 | No action needed | 1 | 1 | 1 | 1 | 1 | 1 | 1 | 1 | 1 | 1 | 1 | 1 | 1 | 1 | 1 | 1 | 1 | 1 | 1 | 1 |
| 142 | No action needed | 1 | 1 | 1 | 1 | 1 | 1 | 1 | 1 | 1 | 1 | 1 | 1 | 1 | 1 | 1 | 1 | 1 | 1 | 1 | 1 |
| 143 | No action needed | 1 | 1 | 1 | 1 | 1 | 1 | 1 | 1 | 1 | 1 | 1 | 1 | 1 | 1 | 1 | 1 | 1 | 1 | 1 | 1 |
| 144 | No action needed | 1 | 1 | 1 | 1 | 1 | 1 | 1 | 1 | 1 | 1 | 1 | 1 | 1 | 1 | 1 | 1 | 1 | 1 | 1 | 1 |
| 145 | No action needed | 1 | 1 | 1 | 1 | 1 | 1 | 1 | 1 | 1 | 1 | 1 | 1 | 1 | 1 | 1 | 1 | 1 | 1 | 1 | 1 |
| 146 | No action needed | 1 | 1 | 1 | 1 | 1 | 1 | 1 | 1 | 1 | 1 | 1 | 1 | 1 | 1 | 1 | 1 | 1 | 1 | 1 | 1 |
| 147 | No action needed | 1 | 1 | 1 | 1 | 1 | 1 | 1 | 1 | 1 | 1 | 1 | 1 | 1 | 1 | 1 | 1 | 1 | 1 | 1 | 1 |
| 148 | No action needed | 1 | 1 | 1 | 1 | 1 | 1 | 1 | 1 | 1 | 1 | 1 | 1 | 1 | 1 | 1 | 1 | 1 | 1 | 1 | 1 |
| 149 | No action needed | 1 | 1 | 1 | 1 | 1 | 1 | 1 | 1 | 1 | 1 | 1 | 1 | 1 | 1 | 1 | 1 | 1 | 1 | 1 | 1 |
| 150 | No action needed | 1 | 1 | 1 | 1 | 1 | 1 | 1 | 1 | 1 | 1 | 1 | 1 | 1 | 1 | 1 | 1 | 1 | 1 | 1 | 1 |
| 151 | No action needed | 1 | 1 | 1 | 1 | 1 | 1 | 1 | 1 | 1 | 1 | 1 | 1 | 1 | 1 | 1 | 1 | 1 | 1 | 1 | 1 |
| 152 | No action needed | 1 | 1 | 1 | 1 | 1 | 1 | 1 | 1 | 1 | 1 | 1 | 1 | 1 | 1 | 1 | 1 | 1 | 1 | 1 | 1 |
| 153 | No action needed | 1 | 1 | 1 | 1 | 1 | 1 | 1 | 1 | 1 | 1 | 1 | 1 | 1 | 1 | 1 | 1 | 1 | 1 | 1 | 1 |
| 154 | No action needed | 1 | 1 | 1 | 1 | 1 | 1 | 1 | 1 | 1 | 1 | 1 | 1 | 1 | 1 | 1 | 1 | 1 | 1 | 1 | 1 |
| 155 | No action needed | 1 | 1 | 1 | 1 | 1 | 1 | 1 | 1 | 1 | 1 | 1 | 1 | 1 | 1 | 1 | 1 | 1 | 1 | 1 | 1 |
| 156 | No action needed | 1 | 1 | 1 | 1 | 1 | 1 | 1 | 1 | 1 | 1 | 1 | 1 | 1 | 1 | 1 | 1 | 1 | 1 | 1 | 1 |
| 157 | Consider dosage modification | 1 | 1 | 1 | 1 | 1 | 1 | 1 | 1 | 1 | 1 | 1 | 1 | 1 | 1 | 1 | 1 | 1 | 1 | 1 | 1 |
| 158 | Consider dosage modification | 1 | 1 | 1 | 1 | 1 | 1 | 1 | 1 | 1 | 1 | 1 | 1 | 1 | 1 | 1 | 1 | 1 | 1 | 1 | 1 |
| 159 | Consider dosage modification | 1 | 1 | 1 | 1 | 1 | 1 | 1 | 1 | 1 | 1 | 1 | 1 | 1 | 1 | 1 | 1 | 1 | 1 | 1 | 1 |
| 160 | Consider dosage modification | 1 | 1 | 1 | 1 | 1 | 1 | 1 | 1 | 1 | 1 | 1 | 1 | 1 | 1 | 1 | 1 | 1 | 1 | 1 | 1 |
| 161 | No action needed | 1 | 1 | 1 | 1 | 1 | 1 | 1 | 1 | 1 | 1 | 1 | 1 | 1 | 1 | 1 | 1 | 1 | 1 | 1 | 1 |
| 162 | No action needed | 1 | 1 | 1 | 1 | 1 | 1 | 1 | 1 | 1 | 1 | 1 | 1 | 1 | 1 | 1 | 1 | 1 | 1 | 1 | 1 |
| 163 | No action needed | 1 | 1 | 1 | 1 | 1 | 1 | 1 | 1 | 1 | 1 | 1 | 1 | 1 | 1 | 1 | 1 | 1 | 1 | 1 | 1 |
| 164 | No action needed | 1 | 1 | 1 | 1 | 1 | 1 | 1 | 1 | 1 | 1 | 1 | 1 | 1 | 1 | 1 | 1 | 1 | 1 | 1 | 1 |
| 165 | No action needed | 1 | 1 | 1 | 1 | 1 | 1 | 1 | 1 | 1 | 1 | 1 | 1 | 1 | 1 | 1 | 1 | 1 | 1 | 1 | 1 |
| 166 | No action needed | 1 | 1 | 1 | 1 | 1 | 1 | 1 | 1 | 1 | 1 | 1 | 1 | 1 | 1 | 1 | 1 | 1 | 1 | 1 | 1 |
| 167 | No action needed | 1 | 1 | 1 | 1 | 1 | 1 | 1 | 1 | 1 | 1 | 1 | 1 | 1 | 1 | 1 | 1 | 1 | 1 | 1 | 1 |
| 168 | No action needed | 1 | 1 | 1 | 1 | 1 | 1 | 1 | 1 | 1 | 1 | 1 | 1 | 1 | 1 | 1 | 1 | 1 | 1 | 1 | 1 |
| 169 | No action needed | 1 | 1 | 1 | 1 | 1 | 1 | 1 | 1 | 1 | 1 | 1 | 1 | 1 | 1 | 1 | 1 | 1 | 1 | 1 | 1 |
| 170 | No action needed | 1 | 1 | 1 | 1 | 1 | 1 | 1 | 1 | 1 | 1 | 1 | 1 | 1 | 1 | 1 | 1 | 1 | 1 | 1 | 1 |
| 171 | No action needed | 1 | 1 | 1 | 1 | 1 | 1 | 1 | 1 | 1 | 1 | 1 | 1 | 1 | 1 | 1 | 1 | 1 | 1 | 1 | 1 |
| 172 | Change medication | 1 | 1 | 1 | 1 | 1 | 1 | 1 | 1 | 1 | 1 | 1 | 1 | 1 | 1 | 1 | 1 | 1 | 1 | 1 | 1 |
| 173 | No action needed | 1 | 1 | 1 | 1 | 1 | 1 | 1 | 1 | 1 | 1 | 1 | 1 | 1 | 1 | 1 | 1 | 1 | 1 | 1 | 1 |
| 174 | No action needed | 1 | 1 | 1 | 1 | 1 | 1 | 1 | 1 | 1 | 1 | 1 | 1 | 1 | 1 | 1 | 1 | 1 | 1 | 1 | 1 |
| 175 | No action needed | 1 | 1 | 1 | 1 | 1 | 1 | 1 | 1 | 1 | 1 | 1 | 1 | 1 | 1 | 1 | 1 | 1 | 1 | 1 | 1 |
| 176 | No action needed | 1 | 1 | 1 | 1 | 1 | 1 | 1 | 1 | 1 | 1 | 1 | 1 | 1 | 1 | 1 | 1 | 1 | 1 | 1 | 1 |
| 177 | No action needed | 1 | 1 | 1 | 1 | 1 | 1 | 1 | 1 | 1 | 1 | 1 | 1 | 1 | 1 | 1 | 1 | 1 | 1 | 1 | 1 |
| 178 | No action needed | 1 | 1 | 1 | 1 | 1 | 1 | 1 | 1 | 1 | 1 | 1 | 1 | 1 | 1 | 1 | 1 | 1 | 1 | 1 | 1 |
| 179 | Consider dosage modification | 1 | 1 | 1 | 1 | 1 | 1 | 1 | 1 | 1 | 1 | 1 | 1 | 1 | 1 | 1 | 1 | 1 | 1 | 1 | 1 |
| 180 | Consider dosage modification | 1 | 1 | 1 | 1 | 1 | 1 | 1 | 1 | 1 | 1 | 1 | 1 | 1 | 1 | 1 | 1 | 1 | 1 | 1 | 1 |
| 181 | Change medication | 0 | 0 | 1 | 1 | 1 | 0 | 0 | 1 | 1 | 1 | 0 | 0 | 1 | 1 | 0 | 1 | 1 | 0 | 1 | 1 |
| 182 | Change medication | 1 | 1 | 1 | 1 | 1 | 1 | 1 | 1 | 1 | 1 | 1 | 1 | 1 | 1 | 1 | 1 | 1 | 1 | 1 | 1 |
| 183 | Change medication | 1 | 1 | 1 | 1 | 1 | 1 | 1 | 1 | 1 | 1 | 1 | 1 | 1 | 1 | 1 | 1 | 1 | 1 | 1 | 1 |
| 184 | Change medication | 1 | 1 | 1 | 1 | 1 | 1 | 1 | 1 | 1 | 1 | 1 | 1 | 1 | 1 | 1 | 1 | 1 | 1 | 1 | 1 |
| 185 | Change medication | 1 | 1 | 1 | 1 | 1 | 1 | 1 | 1 | 1 | 1 | 1 | 1 | 1 | 1 | 1 | 1 | 1 | 1 | 1 | 1 |
| 186 | Change medication | 1 | 1 | 1 | 1 | 1 | 1 | 1 | 1 | 1 | 1 | 1 | 1 | 1 | 1 | 1 | 1 | 1 | 1 | 1 | 1 |
| 187 | Change medication | 1 | 1 | 1 | 1 | 1 | 1 | 1 | 1 | 1 | 1 | 1 | 1 | 1 | 1 | 1 | 1 | 1 | 1 | 1 | 1 |
| 188 | Change medication | 1 | 1 | 1 | 1 | 1 | 1 | 1 | 1 | 1 | 1 | 1 | 1 | 1 | 1 | 1 | 1 | 1 | 1 | 1 | 1 |
| 189 | Change medication | 1 | 1 | 1 | 1 | 1 | 1 | 1 | 1 | 1 | 1 | 1 | 1 | 1 | 1 | 1 | 1 | 1 | 1 | 1 | 1 |
| 190 | Change medication | 1 | 1 | 1 | 1 | 1 | 1 | 1 | 1 | 1 | 1 | 1 | 1 | 1 | 1 | 1 | 1 | 1 | 1 | 1 | 1 |
| 191 | Change medication | 1 | 1 | 1 | 1 | 1 | 1 | 1 | 1 | 1 | 1 | 1 | 1 | 1 | 1 | 1 | 1 | 1 | 1 | 1 | 1 |
| 192 | Change medication | 1 | 1 | 1 | 1 | 1 | 1 | 1 | 1 | 1 | 1 | 1 | 1 | 1 | 1 | 1 | 1 | 1 | 1 | 1 | 1 |
| 193 | Change medication | 1 | 1 | 1 | 1 | 1 | 1 | 1 | 1 | 1 | 1 | 1 | 1 | 1 | 1 | 1 | 1 | 1 | 1 | 1 | 1 |
| 194 | Change medication | 1 | 1 | 1 | 1 | 1 | 1 | 1 | 1 | 1 | 1 | 1 | 1 | 1 | 1 | 1 | 1 | 1 | 1 | 1 | 1 |
| 195 | Change medication | 1 | 1 | 1 | 1 | 1 | 1 | 1 | 1 | 1 | 1 | 1 | 1 | 1 | 1 | 1 | 1 | 1 | 1 | 1 | 1 |
| 196 | Change medication | 1 | 1 | 1 | 1 | 1 | 1 | 1 | 1 | 1 | 1 | 1 | 1 | 1 | 1 | 1 | 1 | 1 | 1 | 1 | 1 |
| 197 | No action needed | 1 | 1 | 1 | 1 | 1 | 1 | 1 | 1 | 1 | 1 | 1 | 1 | 1 | 1 | 1 | 1 | 1 | 1 | 1 | 1 |
| 198 | Consider dosage modification | 1 | 1 | 1 | 1 | 1 | 1 | 1 | 1 | 1 | 1 | 1 | 1 | 1 | 1 | 1 | 1 | 1 | 1 | 1 | 1 |
| 199 | Consider dosage modification | 1 | 0 | 1 | 0 | 1 | 1 | 1 | 1 | 1 | 1 | 1 | 1 | 1 | 1 | 0 | 1 | 1 | 1 | 1 | 1 |
| 200 | No action needed | 1 | 1 | 1 | 1 | 1 | 1 | 1 | 1 | 1 | 1 | 1 | 1 | 1 | 1 | 1 | 1 | 1 | 1 | 1 | 1 |
| 201 | Change medication | 1 | 1 | 1 | 1 | 1 | 1 | 1 | 1 | 1 | 1 | 1 | 1 | 1 | 1 | 1 | 1 | 1 | 1 | 1 | 1 |
| 202 | Consider dosage modification | 1 | 1 | 1 | 1 | 1 | 1 | 1 | 1 | 1 | 1 | 1 | 1 | 1 | 1 | 1 | 1 | 1 | 1 | 1 | 1 |
| 203 | Change medication | 1 | 1 | 1 | 1 | 1 | 1 | 1 | 1 | 1 | 1 | 1 | 1 | 1 | 1 | 1 | 1 | 1 | 1 | 1 | 1 |
| 204 | Consider dosage modification | 1 | 1 | 1 | 1 | 1 | 1 | 1 | 1 | 1 | 1 | 1 | 1 | 1 | 1 | 1 | 1 | 1 | 1 | 1 | 1 |
| 205 | Change medication | 1 | 1 | 1 | 1 | 1 | 1 | 1 | 1 | 1 | 1 | 1 | 1 | 1 | 1 | 1 | 1 | 1 | 1 | 1 | 1 |
| 206 | Change medication | 1 | 1 | 1 | 1 | 1 | 1 | 1 | 1 | 1 | 1 | 1 | 1 | 1 | 1 | 1 | 1 | 1 | 1 | 1 | 1 |
| 207 | Change medication | 1 | 1 | 1 | 1 | 1 | 1 | 1 | 1 | 1 | 1 | 1 | 1 | 1 | 1 | 1 | 1 | 1 | 1 | 1 | 1 |
| 208 | Change medication | 1 | 1 | 1 | 1 | 1 | 1 | 1 | 1 | 1 | 1 | 1 | 1 | 1 | 1 | 1 | 1 | 1 | 1 | 1 | 1 |
| 209 | Consider dosage modification | 1 | 1 | 1 | 1 | 1 | 1 | 1 | 1 | 1 | 1 | 1 | 1 | 1 | 1 | 1 | 1 | 1 | 1 | 1 | 1 |
| 210 | Consider dosage modification | 1 | 1 | 1 | 1 | 1 | 1 | 1 | 1 | 1 | 1 | 1 | 1 | 1 | 1 | 1 | 1 | 1 | 1 | 1 | 1 |
| 211 | Consider dosage modification | 1 | 1 | 1 | 1 | 1 | 1 | 1 | 1 | 1 | 1 | 1 | 1 | 1 | 1 | 1 | 1 | 1 | 1 | 1 | 1 |
| 212 | Monitor adverse effects | 1 | 1 | 1 | 1 | 1 | 0 | 1 | 1 | 1 | 1 | 1 | 1 | 1 | 1 | 1 | 1 | 1 | 1 | 1 | 1 |
| 213 | No action needed | 1 | 1 | 1 | 1 | 1 | 1 | 1 | 1 | 1 | 1 | 1 | 1 | 1 | 1 | 1 | 1 | 1 | 1 | 1 | 1 |
| 214 | No action needed | 1 | 1 | 1 | 1 | 1 | 1 | 1 | 1 | 1 | 1 | 1 | 1 | 1 | 1 | 1 | 1 | 1 | 1 | 1 | 1 |
| 215 | No action needed | 1 | 1 | 1 | 1 | 1 | 1 | 1 | 1 | 1 | 1 | 1 | 1 | 1 | 1 | 1 | 1 | 1 | 1 | 1 | 1 |
| 216 | No action needed | 1 | 1 | 1 | 1 | 1 | 1 | 1 | 1 | 1 | 1 | 1 | 1 | 1 | 1 | 1 | 1 | 1 | 1 | 1 | 1 |
| 217 | No action needed | 1 | 1 | 1 | 1 | 1 | 1 | 1 | 1 | 1 | 1 | 1 | 1 | 1 | 1 | 1 | 1 | 1 | 1 | 1 | 1 |
| 218 | No action needed | 1 | 1 | 1 | 1 | 1 | 1 | 1 | 1 | 1 | 1 | 1 | 1 | 1 | 1 | 1 | 1 | 1 | 1 | 1 | 1 |
| 219 | No action needed | 1 | 1 | 1 | 1 | 1 | 1 | 1 | 1 | 1 | 1 | 1 | 1 | 1 | 1 | 1 | 1 | 1 | 1 | 1 | 1 |
| 220 | No action needed | 1 | 1 | 1 | 1 | 1 | 1 | 1 | 1 | 1 | 1 | 1 | 1 | 1 | 1 | 1 | 1 | 1 | 1 | 1 | 1 |
| 221 | No action needed | 1 | 1 | 1 | 1 | 1 | 1 | 1 | 1 | 1 | 1 | 1 | 1 | 1 | 1 | 1 | 1 | 1 | 1 | 1 | 1 |
| 222 | Consider dosage modification | 1 | 1 | 1 | 1 | 1 | 1 | 1 | 1 | 1 | 1 | 1 | 1 | 1 | 1 | 1 | 1 | 1 | 1 | 1 | 1 |
| 223 | Consider dosage modification | 1 | 1 | 1 | 1 | 1 | 1 | 1 | 1 | 1 | 1 | 1 | 1 | 1 | 1 | 1 | 1 | 1 | 1 | 1 | 1 |
| 224 | Consider dosage modification | 1 | 1 | 1 | 1 | 1 | 1 | 1 | 1 | 1 | 1 | 1 | 1 | 1 | 1 | 1 | 1 | 1 | 1 | 1 | 1 |
| 225 | Consider dosage modification | 1 | 1 | 1 | 1 | 1 | 1 | 1 | 1 | 1 | 1 | 1 | 1 | 1 | 1 | 1 | 1 | 1 | 1 | 1 | 1 |
| 226 | Consider dosage modification | 1 | 1 | 1 | 1 | 1 | 1 | 1 | 1 | 1 | 1 | 1 | 1 | 1 | 1 | 1 | 1 | 1 | 1 | 1 | 1 |
| 227 | Change medication | 1 | 0 | 1 | 1 | 1 | 0 | 1 | 1 | 1 | 0 | 1 | 1 | 1 | 1 | 1 | 1 | 1 | 1 | 1 | 1 |
| 228 | Change medication | 1 | 1 | 1 | 1 | 1 | 1 | 1 | 1 | 1 | 1 | 1 | 1 | 1 | 1 | 1 | 1 | 1 | 1 | 1 | 1 |
| 229 | Change medication | 1 | 1 | 1 | 1 | 1 | 1 | 1 | 1 | 1 | 1 | 1 | 1 | 1 | 1 | 1 | 1 | 1 | 1 | 1 | 1 |
| 230 | Consider dosage modification | 1 | 1 | 1 | 1 | 1 | 1 | 1 | 1 | 1 | 1 | 1 | 1 | 1 | 1 | 1 | 1 | 1 | 1 | 1 | 1 |
| 231 | Other | 1 | 1 | 1 | 1 | 1 | 1 | 1 | 1 | 1 | 1 | 1 | 1 | 1 | 1 | 1 | 1 | 1 | 1 | 1 | 1 |
| 232 | Change medication | 1 | 1 | 1 | 1 | 1 | 1 | 1 | 1 | 1 | 1 | 1 | 1 | 1 | 1 | 1 | 1 | 1 | 1 | 1 | 1 |
| 233 | Change medication | 1 | 1 | 1 | 1 | 1 | 1 | 1 | 1 | 1 | 1 | 1 | 1 | 1 | 1 | 1 | 1 | 1 | 1 | 1 | 1 |
| 234 | Change medication | 1 | 1 | 1 | 1 | 1 | 1 | 1 | 1 | 1 | 1 | 1 | 1 | 1 | 1 | 1 | 1 | 1 | 1 | 1 | 1 |
| 235 | Change medication | 1 | 1 | 1 | 1 | 1 | 1 | 1 | 1 | 1 | 1 | 1 | 1 | 1 | 1 | 1 | 1 | 1 | 1 | 1 | 1 |
| 236 | Consider dosage modification | 0 | 0 | 0 | 0 | 1 | 0 | 1 | 1 | 1 | 0 | 1 | 1 | 1 | 1 | 1 | 1 | 0 | 1 | 1 | 1 |
| 237 | Other | 1 | 1 | 1 | 1 | 1 | 1 | 1 | 1 | 1 | 1 | 1 | 1 | 1 | 1 | 1 | 1 | 1 | 0 | 1 | 1 |
| 238 | Change medication | 1 | 1 | 1 | 1 | 1 | 1 | 1 | 1 | 1 | 1 | 1 | 1 | 1 | 1 | 1 | 1 | 1 | 1 | 1 | 1 |
| 239 | Consider dosage modification | 1 | 1 | 1 | 1 | 1 | 1 | 1 | 1 | 1 | 1 | 1 | 1 | 1 | 1 | 1 | 1 | 1 | 1 | 1 | 1 |
| 240 | No action needed | 1 | 1 | 1 | 1 | 1 | 1 | 1 | 1 | 1 | 1 | 1 | 1 | 1 | 1 | 1 | 1 | 1 | 1 | 1 | 1 |
| 241 | Consider dosage modification | 1 | 1 | 1 | 1 | 1 | 1 | 1 | 1 | 1 | 1 | 1 | 1 | 1 | 1 | 1 | 1 | 1 | 1 | 1 | 1 |
| 242 | Monitor adverse effects | 1 | 1 | 1 | 1 | 1 | 1 | 1 | 1 | 1 | 1 | 1 | 1 | 1 | 1 | 1 | 1 | 1 | 1 | 1 | 1 |
| 243 | Consider dosage modification | 1 | 1 | 1 | 1 | 1 | 1 | 1 | 1 | 1 | 1 | 1 | 1 | 1 | 1 | 1 | 1 | 1 | 1 | 1 | 1 |
| 244 | Consider dosage modification | 1 | 1 | 1 | 1 | 1 | 1 | 1 | 1 | 1 | 1 | 1 | 1 | 1 | 1 | 1 | 1 | 1 | 1 | 1 | 1 |
| 245 | Change medication | 1 | 0 | 0 | 1 | 1 | 1 | 1 | 1 | 1 | 1 | 1 | 1 | 1 | 1 | 0 | 0 | 1 | 1 | 1 | 1 |
| 246 | Consider dosage modification | 1 | 1 | 1 | 1 | 1 | 1 | 1 | 1 | 1 | 1 | 1 | 1 | 1 | 1 | 1 | 1 | 1 | 1 | 1 | 1 |
| 247 | Consider dosage modification | 1 | 1 | 1 | 1 | 1 | 1 | 1 | 1 | 1 | 1 | 1 | 1 | 1 | 1 | 1 | 1 | 1 | 1 | 1 | 1 |
| 248 | Consider dosage modification | 1 | 1 | 1 | 1 | 1 | 1 | 1 | 1 | 1 | 1 | 1 | 1 | 1 | 1 | 1 | 1 | 1 | 1 | 1 | 1 |
| 249 | Change medication | 1 | 1 | 1 | 1 | 1 | 1 | 1 | 1 | 1 | 1 | 1 | 1 | 1 | 1 | 1 | 1 | 1 | 1 | 1 | 1 |
| 250 | Change medication | 1 | 1 | 1 | 1 | 1 | 1 | 1 | 1 | 1 | 1 | 1 | 1 | 1 | 1 | 1 | 1 | 1 | 1 | 1 | 1 |
| 251 | Change medication | 1 | 1 | 1 | 1 | 1 | 1 | 1 | 1 | 1 | 1 | 1 | 1 | 1 | 1 | 1 | 1 | 1 | 1 | 1 | 1 |
| 252 | Consider dosage modification | 1 | 1 | 1 | 1 | 1 | 1 | 1 | 1 | 1 | 1 | 1 | 1 | 1 | 1 | 1 | 1 | 1 | 1 | 1 | 1 |
| 253 | Consider dosage modification | 0 | 1 | 1 | 0 | 1 | 1 | 1 | 1 | 1 | 1 | 1 | 1 | 1 | 1 | 1 | 1 | 1 | 1 | 1 | 1 |
| 254 | Consider dosage modification | 1 | 1 | 1 | 1 | 0 | 1 | 1 | 1 | 1 | 1 | 1 | 1 | 1 | 0 | 1 | 1 | 1 | 1 | 1 | 1 |
| 255 | Consider dosage modification | 1 | 1 | 1 | 1 | 1 | 1 | 1 | 1 | 1 | 1 | 1 | 1 | 1 | 1 | 1 | 1 | 1 | 1 | 1 | 1 |
| 256 | Consider dosage modification | 1 | 1 | 1 | 1 | 1 | 1 | 1 | 1 | 1 | 1 | 1 | 1 | 1 | 1 | 1 | 1 | 1 | 1 | 1 | 1 |
| 257 | Change medication | 1 | 1 | 1 | 1 | 1 | 1 | 1 | 1 | 1 | 1 | 1 | 1 | 1 | 1 | 1 | 1 | 1 | 1 | 1 | 1 |
| 258 | Change medication | 1 | 1 | 1 | 1 | 1 | 1 | 1 | 1 | 1 | 1 | 1 | 1 | 1 | 1 | 1 | 1 | 1 | 1 | 1 | 1 |
| 259 | Change medication | 1 | 1 | 1 | 1 | 1 | 1 | 1 | 1 | 1 | 1 | 1 | 1 | 1 | 1 | 1 | 1 | 1 | 1 | 1 | 1 |
| 260 | Change medication | 1 | 1 | 1 | 1 | 1 | 1 | 1 | 1 | 1 | 1 | 1 | 1 | 1 | 1 | 1 | 1 | 1 | 1 | 1 | 1 |
| 261 | Change medication | 1 | 1 | 1 | 1 | 1 | 1 | 1 | 1 | 1 | 1 | 1 | 1 | 1 | 1 | 1 | 1 | 1 | 1 | 1 | 1 |
| 262 | Consider dosage modification | 1 | 1 | 1 | 1 | 1 | 1 | 1 | 1 | 1 | 1 | 1 | 1 | 1 | 1 | 1 | 1 | 1 | 1 | 1 | 1 |
| 263 | Consider dosage modification | 1 | 1 | 1 | 1 | 1 | 1 | 1 | 1 | 1 | 1 | 1 | 1 | 1 | 1 | 1 | 1 | 1 | 1 | 1 | 1 |
| 264 | Consider dosage modification | 1 | 1 | 1 | 1 | 1 | 1 | 1 | 1 | 1 | 1 | 1 | 1 | 1 | 1 | 1 | 1 | 1 | 1 | 1 | 1 |
| 265 | Change medication | 1 | 1 | 1 | 1 | 1 | 1 | 1 | 1 | 1 | 1 | 1 | 1 | 1 | 1 | 1 | 1 | 1 | 1 | 1 | 1 |
| 266 | Consider dosage modification | 1 | 1 | 1 | 1 | 1 | 1 | 1 | 1 | 1 | 1 | 1 | 1 | 1 | 1 | 1 | 1 | 1 | 1 | 1 | 1 |
| 267 | Change medication | 1 | 1 | 1 | 1 | 1 | 1 | 1 | 1 | 1 | 1 | 1 | 1 | 1 | 1 | 1 | 1 | 1 | 1 | 1 | 1 |
| 268 | Monitor adverse effects | 0 | 0 | 1 | 1 | 1 | 0 | 1 | 1 | 1 | 0 | 1 | 0 | 1 | 0 | 1 | 1 | 1 | 1 | 1 | 1 |
| 269 | Consider dosage modification | 1 | 1 | 1 | 1 | 1 | 1 | 1 | 1 | 1 | 1 | 1 | 1 | 1 | 1 | 1 | 1 | 1 | 1 | 1 | 1 |
| 270 | Change medication | 1 | 1 | 1 | 1 | 1 | 1 | 1 | 1 | 1 | 1 | 1 | 1 | 1 | 1 | 1 | 1 | 1 | 1 | 1 | 1 |
| 271 | Change medication | 1 | 1 | 1 | 1 | 1 | 1 | 1 | 1 | 1 | 1 | 1 | 1 | 1 | 1 | 1 | 1 | 1 | 1 | 1 | 1 |
| 272 | Change medication | 1 | 1 | 1 | 1 | 1 | 1 | 1 | 1 | 1 | 1 | 1 | 1 | 1 | 1 | 1 | 1 | 1 | 1 | 1 | 1 |
| 273 | Consider dosage modification | 1 | 1 | 1 | 1 | 1 | 1 | 1 | 1 | 1 | 1 | 1 | 1 | 1 | 1 | 1 | 1 | 1 | 1 | 1 | 1 |
| 274 | Change medication | 1 | 1 | 1 | 1 | 1 | 1 | 1 | 1 | 1 | 1 | 1 | 1 | 1 | 1 | 1 | 1 | 1 | 1 | 1 | 1 |
| 275 | Consider dosage modification | 1 | 1 | 1 | 1 | 1 | 1 | 1 | 1 | 1 | 1 | 1 | 1 | 1 | 1 | 1 | 1 | 1 | 1 | 1 | 1 |
| 276 | Consider dosage modification | 1 | 1 | 1 | 1 | 1 | 1 | 1 | 1 | 1 | 1 | 1 | 1 | 1 | 1 | 1 | 1 | 1 | 1 | 1 | 1 |
| 277 | Consider dosage modification | 1 | 1 | 1 | 1 | 1 | 1 | 1 | 1 | 1 | 1 | 1 | 1 | 1 | 1 | 1 | 1 | 1 | 1 | 1 | 1 |
| 278 | Consider dosage modification | 1 | 1 | 1 | 1 | 1 | 1 | 1 | 1 | 1 | 1 | 1 | 1 | 1 | 1 | 1 | 1 | 1 | 1 | 1 | 1 |
| 279 | Consider dosage modification | 1 | 1 | 1 | 1 | 1 | 1 | 1 | 1 | 1 | 1 | 1 | 1 | 1 | 1 | 1 | 1 | 1 | 1 | 1 | 1 |
| 280 | Consider dosage modification | 1 | 1 | 1 | 1 | 1 | 1 | 1 | 1 | 1 | 1 | 1 | 1 | 1 | 1 | 1 | 1 | 1 | 1 | 1 | 1 |
| 281 | Consider dosage modification | 1 | 1 | 1 | 1 | 1 | 1 | 1 | 1 | 1 | 1 | 1 | 1 | 1 | 1 | 1 | 1 | 1 | 1 | 1 | 1 |
| 282 | Consider dosage modification | 1 | 1 | 1 | 1 | 1 | 1 | 1 | 1 | 1 | 1 | 1 | 1 | 1 | 1 | 1 | 1 | 1 | 1 | 1 | 1 |
| 283 | Consider dosage modification | 1 | 1 | 1 | 1 | 1 | 1 | 1 | 1 | 1 | 1 | 1 | 1 | 1 | 1 | 1 | 1 | 1 | 1 | 1 | 1 |
| 284 | Consider dosage modification | 1 | 1 | 1 | 1 | 1 | 1 | 1 | 1 | 1 | 1 | 1 | 1 | 1 | 1 | 1 | 1 | 1 | 1 | 1 | 1 |
| 285 | Change medication | 1 | 1 | 1 | 1 | 1 | 1 | 1 | 1 | 1 | 1 | 1 | 1 | 1 | 1 | 1 | 1 | 1 | 1 | 1 | 1 |
| 286 | Monitor adverse effects | 1 | 1 | 1 | 1 | 1 | 1 | 1 | 1 | 1 | 1 | 1 | 1 | 1 | 1 | 1 | 1 | 1 | 1 | 1 | 1 |
| 287 | Change medication | 1 | 1 | 1 | 1 | 1 | 1 | 1 | 1 | 1 | 1 | 1 | 1 | 1 | 1 | 1 | 1 | 1 | 1 | 1 | 1 |
| 288 | No action needed | 1 | 1 | 1 | 1 | 1 | 1 | 1 | 1 | 1 | 1 | 1 | 1 | 1 | 1 | 1 | 1 | 1 | 1 | 1 | 1 |
| 289 | No action needed | 1 | 1 | 1 | 1 | 1 | 1 | 1 | 1 | 1 | 1 | 1 | 1 | 1 | 1 | 1 | 1 | 1 | 1 | 1 | 1 |
| 290 | Consider dosage modification | 1 | 1 | 1 | 0 | 1 | 1 | 1 | 1 | 1 | 1 | 1 | 1 | 1 | 1 | 1 | 1 | 1 | 0 | 0 | 1 |
| 291 | Consider dosage modification | 1 | 1 | 1 | 1 | 1 | 1 | 1 | 1 | 1 | 1 | 1 | 1 | 1 | 1 | 1 | 1 | 1 | 1 | 1 | 1 |
| 292 | Consider dosage modification | 1 | 1 | 1 | 1 | 1 | 1 | 1 | 1 | 1 | 1 | 1 | 1 | 1 | 1 | 1 | 1 | 1 | 1 | 1 | 1 |
| 293 | No action needed | 1 | 1 | 1 | 1 | 1 | 1 | 1 | 1 | 1 | 1 | 1 | 1 | 1 | 1 | 1 | 1 | 1 | 1 | 1 | 1 |
| 294 | Change medication | 1 | 1 | 1 | 1 | 1 | 1 | 1 | 1 | 1 | 1 | 1 | 1 | 1 | 1 | 1 | 1 | 1 | 1 | 1 | 1 |
| 295 | No action needed | 1 | 1 | 1 | 1 | 1 | 1 | 1 | 1 | 1 | 1 | 1 | 1 | 1 | 1 | 1 | 1 | 1 | 1 | 1 | 1 |
| 296 | Change medication | 1 | 1 | 1 | 1 | 1 | 1 | 1 | 1 | 1 | 1 | 1 | 1 | 1 | 1 | 1 | 1 | 1 | 1 | 1 | 1 |
| 297 | Change medication | 1 | 1 | 1 | 1 | 1 | 1 | 1 | 1 | 1 | 1 | 1 | 1 | 1 | 1 | 1 | 1 | 1 | 1 | 1 | 1 |
| 298 | Change medication | 1 | 1 | 1 | 1 | 1 | 1 | 1 | 1 | 1 | 1 | 1 | 1 | 1 | 1 | 1 | 1 | 1 | 1 | 1 | 1 |
| 299 | Consider dosage modification | 1 | 1 | 1 | 1 | 1 | 1 | 1 | 1 | 1 | 1 | 1 | 1 | 1 | 1 | 1 | 1 | 1 | 1 | 1 | 1 |
| 300 | Consider dosage modification | 1 | 1 | 1 | 1 | 1 | 1 | 1 | 1 | 1 | 1 | 1 | 1 | 1 | 1 | 1 | 1 | 1 | 1 | 1 | 1 |
| 301 | Change medication | 1 | 1 | 1 | 1 | 1 | 1 | 1 | 1 | 1 | 1 | 1 | 1 | 1 | 1 | 1 | 1 | 1 | 1 | 1 | 1 |
| 302 | No action needed | 1 | 1 | 1 | 1 | 1 | 1 | 1 | 1 | 1 | 1 | 1 | 1 | 1 | 1 | 1 | 1 | 1 | 1 | 1 | 1 |
| 303 | No action needed | 1 | 1 | 1 | 1 | 1 | 1 | 1 | 1 | 1 | 1 | 1 | 1 | 1 | 1 | 1 | 1 | 1 | 1 | 1 | 1 |
| 304 | Change medication | 1 | 1 | 1 | 0 | 1 | 1 | 1 | 0 | 1 | 0 | 1 | 1 | 1 | 1 | 0 | 1 | 1 | 1 | 0 | 0 |
| 305 | No action needed | 1 | 1 | 1 | 1 | 1 | 1 | 1 | 1 | 1 | 1 | 1 | 1 | 1 | 1 | 1 | 1 | 1 | 1 | 1 | 1 |
| 306 | Monitor adverse effects | 1 | 1 | 1 | 1 | 1 | 1 | 1 | 1 | 1 | 1 | 1 | 1 | 1 | 1 | 1 | 1 | 1 | 1 | 1 | 1 |
| 307 | Consider dosage modification | 1 | 1 | 1 | 1 | 1 | 1 | 1 | 1 | 1 | 1 | 1 | 1 | 1 | 1 | 1 | 1 | 1 | 1 | 1 | 1 |
| 308 | No action needed | 1 | 1 | 1 | 1 | 1 | 1 | 1 | 1 | 1 | 1 | 1 | 1 | 1 | 1 | 1 | 1 | 1 | 1 | 1 | 1 |
| 309 | Consider dosage modification | 1 | 1 | 1 | 1 | 1 | 1 | 1 | 1 | 1 | 1 | 1 | 1 | 1 | 1 | 1 | 1 | 1 | 1 | 1 | 1 |
| 310 | Consider dosage modification | 1 | 1 | 1 | 1 | 1 | 1 | 1 | 1 | 1 | 1 | 1 | 1 | 1 | 1 | 1 | 1 | 1 | 1 | 1 | 1 |
| 311 | No action needed | 1 | 1 | 1 | 1 | 1 | 1 | 1 | 1 | 1 | 1 | 1 | 1 | 1 | 1 | 1 | 1 | 1 | 1 | 1 | 1 |
| 312 | Change medication | 1 | 1 | 1 | 1 | 1 | 1 | 1 | 1 | 1 | 1 | 1 | 1 | 1 | 1 | 1 | 1 | 1 | 1 | 1 | 1 |
| 313 | Consider dosage modification | 1 | 1 | 1 | 1 | 1 | 1 | 1 | 1 | 1 | 1 | 1 | 1 | 1 | 1 | 1 | 1 | 1 | 1 | 1 | 1 |
| 314 | Consider dosage modification | 1 | 1 | 1 | 1 | 1 | 1 | 1 | 1 | 1 | 1 | 1 | 1 | 1 | 1 | 1 | 1 | 1 | 1 | 1 | 1 |
| 315 | No action needed | 1 | 1 | 1 | 1 | 1 | 1 | 1 | 1 | 1 | 1 | 1 | 1 | 1 | 1 | 1 | 1 | 1 | 1 | 1 | 1 |
| 316 | Change medication | 1 | 1 | 1 | 1 | 1 | 1 | 1 | 1 | 1 | 1 | 1 | 1 | 1 | 1 | 1 | 1 | 1 | 1 | 1 | 1 |
| 317 | Consider dosage modification | 1 | 1 | 1 | 1 | 1 | 1 | 1 | 1 | 1 | 1 | 1 | 1 | 1 | 1 | 1 | 1 | 1 | 1 | 1 | 1 |
| 318 | Change medication | 1 | 1 | 1 | 1 | 1 | 1 | 1 | 1 | 1 | 1 | 1 | 1 | 1 | 1 | 1 | 1 | 1 | 1 | 1 | 1 |
| 319 | Change medication | 1 | 1 | 1 | 1 | 1 | 1 | 1 | 1 | 1 | 1 | 1 | 1 | 1 | 1 | 1 | 1 | 1 | 1 | 1 | 1 |
| 320 | No action needed | 0 | 1 | 1 | 1 | 1 | 0 | 1 | 1 | 0 | 0 | 1 | 1 | 0 | 0 | 0 | 1 | 1 | 1 | 1 | 1 |
| 321 | No action needed | 1 | 1 | 1 | 1 | 1 | 1 | 1 | 1 | 1 | 1 | 1 | 1 | 1 | 1 | 1 | 1 | 1 | 1 | 1 | 1 |
| 322 | No action needed | 1 | 1 | 1 | 1 | 1 | 1 | 1 | 1 | 1 | 1 | 1 | 1 | 1 | 1 | 1 | 1 | 1 | 1 | 1 | 1 |
| 323 | No action needed | 1 | 1 | 1 | 1 | 1 | 1 | 1 | 1 | 1 | 1 | 1 | 1 | 1 | 1 | 1 | 1 | 1 | 1 | 1 | 1 |
| 324 | No action needed | 1 | 1 | 1 | 1 | 1 | 1 | 1 | 1 | 1 | 1 | 1 | 1 | 1 | 1 | 1 | 1 | 1 | 1 | 1 | 1 |
| 325 | No action needed | 1 | 1 | 1 | 1 | 1 | 1 | 1 | 1 | 1 | 1 | 1 | 1 | 1 | 1 | 1 | 1 | 1 | 1 | 1 | 1 |
| 326 | No action needed | 1 | 1 | 1 | 1 | 1 | 1 | 1 | 1 | 1 | 1 | 1 | 1 | 1 | 1 | 1 | 1 | 1 | 1 | 1 | 1 |
| 327 | No action needed | 1 | 1 | 1 | 1 | 1 | 1 | 1 | 1 | 1 | 1 | 1 | 1 | 1 | 1 | 1 | 1 | 1 | 1 | 1 | 1 |
| 328 | No action needed | 1 | 1 | 1 | 1 | 1 | 1 | 1 | 1 | 1 | 1 | 1 | 1 | 1 | 1 | 1 | 1 | 1 | 1 | 1 | 1 |
| 329 | Consider dosage modification | 1 | 1 | 1 | 1 | 1 | 1 | 1 | 1 | 1 | 1 | 1 | 1 | 1 | 1 | 1 | 1 | 1 | 1 | 1 | 1 |
| 330 | Consider dosage modification | 1 | 1 | 1 | 1 | 1 | 1 | 1 | 1 | 1 | 1 | 1 | 1 | 1 | 1 | 1 | 1 | 1 | 1 | 1 | 1 |
| 331 | Consider dosage modification | 1 | 1 | 1 | 1 | 1 | 1 | 1 | 1 | 1 | 1 | 1 | 1 | 1 | 1 | 1 | 1 | 1 | 1 | 1 | 1 |
| 332 | Consider dosage modification | 1 | 1 | 1 | 1 | 1 | 1 | 1 | 1 | 1 | 1 | 1 | 1 | 1 | 1 | 1 | 1 | 1 | 1 | 1 | 1 |
| 333 | Consider dosage modification | 1 | 1 | 1 | 1 | 1 | 1 | 1 | 1 | 1 | 1 | 1 | 1 | 1 | 1 | 1 | 1 | 1 | 1 | 1 | 1 |
| 334 | Change medication | 1 | 1 | 1 | 1 | 1 | 1 | 1 | 1 | 1 | 1 | 1 | 1 | 1 | 1 | 1 | 1 | 1 | 1 | 1 | 1 |
| 335 | Consider dosage modification | 1 | 1 | 1 | 1 | 1 | 1 | 1 | 1 | 1 | 1 | 1 | 1 | 1 | 1 | 1 | 1 | 1 | 1 | 1 | 1 |
| 336 | Consider dosage modification | 1 | 1 | 1 | 1 | 1 | 1 | 1 | 1 | 1 | 1 | 1 | 1 | 1 | 1 | 1 | 1 | 1 | 1 | 1 | 1 |
| 337 | Consider dosage modification | 1 | 1 | 1 | 1 | 1 | 1 | 1 | 1 | 1 | 1 | 1 | 1 | 1 | 1 | 1 | 1 | 1 | 1 | 1 | 1 |
| 338 | No action needed | 1 | 1 | 1 | 1 | 1 | 1 | 1 | 1 | 1 | 1 | 1 | 1 | 1 | 1 | 1 | 1 | 1 | 1 | 1 | 1 |
| 339 | No action needed | 1 | 1 | 1 | 1 | 1 | 1 | 1 | 1 | 1 | 1 | 1 | 1 | 1 | 1 | 1 | 1 | 1 | 1 | 1 | 1 |
| 340 | No action needed | 1 | 1 | 1 | 1 | 1 | 1 | 1 | 1 | 1 | 1 | 1 | 1 | 1 | 1 | 1 | 1 | 1 | 1 | 1 | 1 |
| 341 | No action needed | 1 | 1 | 1 | 1 | 1 | 1 | 1 | 1 | 1 | 1 | 1 | 1 | 1 | 1 | 1 | 1 | 1 | 1 | 1 | 1 |
| 342 | Consider dosage modification | 1 | 1 | 1 | 1 | 1 | 1 | 1 | 1 | 1 | 1 | 1 | 1 | 1 | 1 | 1 | 1 | 1 | 1 | 1 | 1 |
| 343 | No action needed | 1 | 1 | 1 | 1 | 1 | 1 | 1 | 1 | 1 | 1 | 1 | 1 | 1 | 1 | 1 | 1 | 1 | 1 | 1 | 1 |
| 344 | No action needed | 1 | 1 | 1 | 1 | 1 | 1 | 1 | 1 | 1 | 1 | 1 | 1 | 1 | 1 | 1 | 1 | 1 | 1 | 1 | 1 |
| 345 | Consider dosage modification | 1 | 1 | 1 | 1 | 1 | 1 | 1 | 1 | 1 | 1 | 1 | 1 | 1 | 1 | 1 | 1 | 1 | 1 | 1 | 1 |
| 346 | No action needed | 1 | 1 | 1 | 1 | 1 | 1 | 1 | 1 | 1 | 1 | 1 | 1 | 1 | 1 | 1 | 1 | 1 | 1 | 1 | 1 |
| 347 | No action needed | 1 | 1 | 1 | 1 | 1 | 1 | 1 | 1 | 1 | 1 | 1 | 1 | 1 | 1 | 1 | 1 | 1 | 1 | 1 | 1 |
| 348 | Consider dosage modification | 1 | 1 | 1 | 1 | 1 | 1 | 1 | 1 | 1 | 1 | 1 | 1 | 1 | 1 | 1 | 1 | 1 | 1 | 1 | 1 |
| 349 | Change medication | 1 | 1 | 1 | 1 | 1 | 1 | 1 | 1 | 1 | 1 | 1 | 1 | 1 | 1 | 1 | 1 | 1 | 1 | 1 | 1 |
| 350 | Consider dosage modification | 1 | 0 | 0 | 0 | 1 | 0 | 0 | 1 | 1 | 1 | 1 | 1 | 0 | 0 | 0 | 1 | 1 | 1 | 0 | 1 |
| 351 | No action needed | 1 | 1 | 1 | 1 | 1 | 1 | 1 | 1 | 1 | 1 | 1 | 1 | 1 | 1 | 1 | 1 | 1 | 1 | 1 | 1 |
| 352 | Consider dosage modification | 1 | 1 | 1 | 1 | 1 | 1 | 1 | 1 | 1 | 1 | 1 | 1 | 1 | 1 | 1 | 1 | 1 | 1 | 1 | 1 |
| 353 | Change medication | 1 | 1 | 1 | 1 | 1 | 1 | 1 | 1 | 1 | 1 | 1 | 1 | 1 | 1 | 1 | 1 | 1 | 1 | 1 | 1 |
| 354 | Consider dosage modification | 1 | 0 | 1 | 1 | 0 | 1 | 0 | 0 | 0 | 0 | 0 | 0 | 1 | 0 | 1 | 1 | 1 | 1 | 1 | 0 |
| 355 | Consider dosage modification | 1 | 1 | 1 | 1 | 1 | 1 | 1 | 1 | 1 | 1 | 1 | 1 | 1 | 1 | 1 | 1 | 1 | 1 | 1 | 1 |
| 356 | Consider dosage modification | 1 | 1 | 1 | 1 | 1 | 1 | 1 | 1 | 1 | 1 | 1 | 1 | 1 | 1 | 1 | 1 | 1 | 1 | 1 | 1 |
| 357 | Change medication | 1 | 1 | 1 | 1 | 1 | 1 | 1 | 1 | 1 | 1 | 1 | 1 | 1 | 1 | 1 | 1 | 1 | 1 | 1 | 1 |
| 358 | Consider dosage modification | 1 | 1 | 1 | 1 | 1 | 1 | 1 | 1 | 1 | 1 | 1 | 1 | 1 | 1 | 1 | 1 | 1 | 1 | 1 | 1 |
| 359 | No action needed | 1 | 1 | 1 | 1 | 1 | 1 | 1 | 1 | 1 | 1 | 1 | 1 | 1 | 1 | 1 | 1 | 1 | 1 | 1 | 1 |
| 360 | Consider dosage modification | 0 | 1 | 0 | 1 | 1 | 0 | 0 | 0 | 1 | 1 | 1 | 0 | 1 | 1 | 0 | 0 | 1 | 0 | 1 | 1 |
| 361 | No action needed | 1 | 1 | 1 | 1 | 1 | 1 | 1 | 1 | 1 | 1 | 1 | 1 | 1 | 1 | 1 | 1 | 1 | 1 | 1 | 1 |
| 362 | Change medication | 1 | 1 | 1 | 1 | 1 | 1 | 1 | 1 | 1 | 1 | 1 | 1 | 1 | 1 | 1 | 1 | 1 | 1 | 1 | 1 |
| 363 | Monitor adverse effects | 1 | 1 | 1 | 1 | 1 | 1 | 1 | 0 | 0 | 1 | 1 | 1 | 1 | 1 | 1 | 1 | 1 | 0 | 0 | 0 |
| 364 | No action needed | 1 | 1 | 1 | 1 | 1 | 1 | 1 | 1 | 1 | 1 | 1 | 1 | 1 | 1 | 1 | 1 | 1 | 1 | 1 | 1 |
| 365 | Consider dosage modification | 1 | 1 | 1 | 1 | 1 | 1 | 1 | 1 | 1 | 1 | 1 | 1 | 1 | 1 | 1 | 1 | 1 | 1 | 1 | 1 |
| 366 | Consider dosage modification | 1 | 1 | 1 | 1 | 1 | 1 | 1 | 1 | 1 | 1 | 1 | 1 | 1 | 1 | 1 | 1 | 1 | 1 | 1 | 1 |
| 367 | Consider dosage modification | 1 | 1 | 1 | 1 | 1 | 1 | 1 | 1 | 1 | 1 | 1 | 1 | 1 | 1 | 1 | 1 | 1 | 1 | 1 | 1 |
| 368 | No action needed | 1 | 1 | 1 | 1 | 1 | 1 | 1 | 1 | 1 | 1 | 1 | 1 | 1 | 1 | 1 | 1 | 1 | 1 | 1 | 1 |
| 369 | Change medication | 1 | 1 | 1 | 1 | 1 | 1 | 1 | 1 | 1 | 1 | 1 | 1 | 1 | 1 | 1 | 1 | 1 | 1 | 1 | 1 |
| 370 | Consider dosage modification | 1 | 1 | 1 | 1 | 1 | 1 | 1 | 1 | 1 | 1 | 1 | 1 | 1 | 1 | 1 | 1 | 1 | 1 | 1 | 1 |
| 371 | Consider dosage modification | 1 | 1 | 1 | 1 | 1 | 0 | 1 | 1 | 1 | 1 | 0 | 1 | 1 | 1 | 1 | 1 | 1 | 1 | 1 | 1 |
| 372 | Monitor adverse effects | 1 | 1 | 1 | 1 | 1 | 1 | 1 | 1 | 1 | 1 | 1 | 1 | 1 | 1 | 1 | 1 | 1 | 1 | 1 | 1 |
| 373 | Change medication | 1 | 1 | 1 | 1 | 1 | 1 | 1 | 1 | 1 | 1 | 1 | 1 | 1 | 1 | 1 | 1 | 1 | 1 | 1 | 1 |
| 374 | Change medication | 1 | 1 | 1 | 1 | 1 | 1 | 1 | 1 | 1 | 1 | 1 | 1 | 1 | 1 | 1 | 1 | 1 | 1 | 1 | 1 |
| 375 | Consider dosage modification | 1 | 1 | 1 | 1 | 1 | 1 | 1 | 1 | 1 | 1 | 1 | 1 | 1 | 1 | 1 | 1 | 1 | 1 | 1 | 1 |
| 376 | Consider dosage modification | 1 | 1 | 1 | 1 | 1 | 1 | 1 | 1 | 1 | 1 | 1 | 1 | 1 | 1 | 1 | 1 | 1 | 1 | 1 | 1 |
| 377 | No action needed | 1 | 1 | 1 | 1 | 1 | 1 | 1 | 1 | 1 | 1 | 1 | 1 | 1 | 1 | 1 | 1 | 1 | 1 | 1 | 1 |
| 378 | Monitor adverse effects | 1 | 1 | 1 | 1 | 1 | 1 | 1 | 1 | 1 | 1 | 1 | 1 | 1 | 1 | 1 | 1 | 1 | 1 | 1 | 1 |
| 379 | Change medication | 1 | 1 | 1 | 1 | 1 | 1 | 1 | 1 | 1 | 1 | 1 | 1 | 1 | 1 | 1 | 1 | 1 | 1 | 1 | 1 |
| 380 | Change medication | 1 | 1 | 1 | 1 | 1 | 1 | 1 | 1 | 1 | 1 | 1 | 1 | 1 | 1 | 1 | 1 | 1 | 1 | 1 | 1 |
| 381 | Consider dosage modification | 1 | 1 | 1 | 1 | 1 | 1 | 1 | 1 | 1 | 1 | 1 | 1 | 1 | 1 | 1 | 1 | 1 | 1 | 1 | 1 |
| 382 | Consider dosage modification | 1 | 1 | 1 | 1 | 1 | 1 | 1 | 1 | 1 | 1 | 1 | 1 | 1 | 1 | 1 | 1 | 1 | 1 | 1 | 1 |
| 383 | Consider dosage modification | 1 | 1 | 1 | 1 | 1 | 1 | 1 | 1 | 1 | 1 | 1 | 1 | 1 | 1 | 1 | 1 | 1 | 1 | 1 | 1 |
| 384 | Consider dosage modification | 1 | 1 | 1 | 1 | 1 | 1 | 1 | 1 | 1 | 1 | 1 | 1 | 1 | 1 | 1 | 1 | 1 | 1 | 1 | 1 |
| 385 | Change medication | 1 | 1 | 1 | 1 | 1 | 1 | 1 | 1 | 1 | 1 | 1 | 1 | 1 | 1 | 1 | 1 | 1 | 1 | 1 | 1 |

1. Base Value: The most frequent classification result observed across 20 tests.
2. For Germini 1.5-Pro, classifications that agreed with base value data were coded as 1, while disagreements were coded as 0.

**Table S4. Classification results by five pharmacists from different healthcare systems**

| **No.** | **Final Label ^a^** | **Individual results of predictions by 5 pharmacist ^b^** | | | | |
| --- | --- | --- | --- | --- | --- | --- |
|  |  | **H.Y.H** | **L.H.L** | **C.L.W** | **C.Y.L** | **C.K.L** |
| 1 | No action needed | 1 | 1 | 1 | 1 | 1 |
| 2 | No action needed | 1 | 1 | 1 | 1 | 1 |
| 3 | No action needed | 1 | 1 | 1 | 1 | 1 |
| 4 | No action needed | 1 | 1 | 1 | 1 | 1 |
| 5 | No action needed | 1 | 1 | 1 | 1 | 1 |
| 6 | No action needed | 1 | 1 | 1 | 1 | 1 |
| 7 | No action needed | 1 | 1 | 1 | 1 | 1 |
| 8 | No action needed | 1 | 1 | 1 | 1 | 1 |
| 9 | No action needed | 1 | 1 | 1 | 1 | 1 |
| 10 | No action needed | 1 | 1 | 1 | 1 | 1 |
| 11 | No action needed | 1 | 1 | 1 | 1 | 1 |
| 12 | No action needed | 1 | 1 | 1 | 1 | 1 |
| 13 | No action needed | 1 | 1 | 1 | 1 | 1 |
| 14 | No action needed | 1 | 1 | 1 | 1 | 1 |
| 15 | No action needed | 1 | 1 | 1 | 1 | 1 |
| 16 | No action needed | 1 | 1 | 1 | 1 | 1 |
| 17 | No action needed | 1 | 1 | 1 | 1 | 1 |
| 18 | No action needed | 1 | 1 | 1 | 1 | 1 |
| 19 | No action needed | 1 | 1 | 1 | 1 | 1 |
| 20 | No action needed | 1 | 1 | 1 | 1 | 1 |
| 21 | No action needed | 1 | 1 | 1 | 1 | 1 |
| 22 | No action needed | 1 | 1 | 1 | 1 | 1 |
| 23 | No action needed | 1 | 1 | 1 | 1 | 1 |
| 24 | No action needed | 1 | 1 | 1 | 1 | 1 |
| 25 | Change medication | 3 | 3 | 3 | 3 | 3 |
| 26 | Change medication | 3 | 3 | 3 | 2 | 3 |
| 27 | Change medication | 3 | 3 | 3 | 2 | 3 |
| 28 | Change medication | 3 | 3 | 3 | 3 | 3 |
| 29 | Consider dosage modification | 2 | 2 | 3 | 2 | 2 |
| 30 | Consider dosage modification | 2 | 2 | 3 | 2 | 2 |
| 31 | Change medication | 3 | 3 | 3 | 3 | 3 |
| 32 | Change medication | 3 | 3 | 3 | 3 | 3 |
| 33 | Change medication | 3 | 3 | 3 | 3 | 3 |
| 34 | Change medication | 3 | 3 | 3 | 3 | 3 |
| 35 | Change medication | 3 | 3 | 3 | 3 | 3 |
| 36 | Change medication | 3 | 3 | 3 | 3 | 3 |
| 37 | Change medication | 3 | 3 | 3 | 3 | 3 |
| 38 | Change medication | 3 | 3 | 3 | 3 | 3 |
| 39 | Change medication | 3 | 3 | 3 | 3 | 3 |
| 40 | Change medication | 3 | 3 | 3 | 3 | 3 |
| 41 | Change medication | 3 | 3 | 3 | 3 | 3 |
| 42 | Change medication | 3 | 3 | 3 | 3 | 3 |
| 43 | Consider dosage modification | 2 | 4 | 2 | 2 | 2 |
| 44 | Consider dosage modification | 2 | 2 | 2 | 2 | 2 |
| 45 | No action needed | 1 | 1 | 1 | 1 | 1 |
| 46 | No action needed | 1 | 1 | 1 | 1 | 1 |
| 47 | No action needed | 1 | 1 | 1 | 1 | 1 |
| 48 | No action needed | 1 | 1 | 1 | 1 | 1 |
| 49 | No action needed | 1 | 1 | 1 | 1 | 1 |
| 50 | No action needed | 1 | 1 | 1 | 1 | 1 |
| 51 | No action needed | 1 | 1 | 1 | 1 | 1 |
| 52 | No action needed | 1 | 1 | 1 | 1 | 1 |
| 53 | No action needed | 1 | 1 | 1 | 1 | 1 |
| 54 | No action needed | 1 | 1 | 1 | 1 | 1 |
| 55 | No action needed | 1 | 1 | 1 | 1 | 1 |
| 56 | No action needed | 1 | 1 | 1 | 1 | 1 |
| 57 | No action needed | 1 | 1 | 1 | 1 | 1 |
| 58 | No action needed | 1 | 1 | 1 | 1 | 1 |
| 59 | No action needed | 1 | 1 | 1 | 1 | 1 |
| 60 | No action needed | 1 | 1 | 1 | 1 | 1 |
| 61 | No action needed | 1 | 1 | 1 | 1 | 1 |
| 62 | No action needed | 1 | 1 | 1 | 1 | 1 |
| 63 | No action needed | 1 | 1 | 1 | 1 | 1 |
| 64 | No action needed | 1 | 1 | 1 | 1 | 1 |
| 65 | No action needed | 1 | 1 | 1 | 1 | 1 |
| 66 | No action needed | 1 | 1 | 1 | 1 | 1 |
| 67 | No action needed | 1 | 1 | 1 | 1 | 1 |
| 68 | No action needed | 1 | 1 | 1 | 1 | 1 |
| 69 | No action needed | 1 | 1 | 1 | 1 | 1 |
| 70 | No action needed | 1 | 1 | 1 | 1 | 1 |
| 71 | No action needed | 1 | 1 | 1 | 1 | 1 |
| 72 | No action needed | 1 | 1 | 1 | 1 | 1 |
| 73 | No action needed | 1 | 1 | 1 | 1 | 1 |
| 74 | No action needed | 1 | 1 | 1 | 1 | 1 |
| 75 | No action needed | 1 | 1 | 1 | 1 | 1 |
| 76 | No action needed | 1 | 1 | 1 | 1 | 1 |
| 77 | No action needed | 1 | 1 | 1 | 1 | 1 |
| 78 | No action needed | 1 | 1 | 1 | 1 | 1 |
| 79 | No action needed | 1 | 1 | 1 | 1 | 1 |
| 80 | No action needed | 1 | 1 | 1 | 1 | 1 |
| 81 | No action needed | 1 | 1 | 1 | 1 | 1 |
| 82 | No action needed | 1 | 1 | 1 | 1 | 1 |
| 83 | No action needed | 1 | 1 | 1 | 1 | 1 |
| 84 | No action needed | 1 | 1 | 1 | 1 | 1 |
| 85 | No action needed | 1 | 1 | 1 | 1 | 1 |
| 86 | No action needed | 1 | 1 | 1 | 1 | 1 |
| 87 | No action needed | 1 | 1 | 1 | 1 | 1 |
| 88 | No action needed | 1 | 1 | 1 | 1 | 1 |
| 89 | No action needed | 1 | 1 | 1 | 1 | 1 |
| 90 | No action needed | 1 | 1 | 1 | 1 | 1 |
| 91 | No action needed | 1 | 1 | 1 | 1 | 1 |
| 92 | No action needed | 1 | 1 | 1 | 1 | 1 |
| 93 | No action needed | 1 | 1 | 1 | 1 | 1 |
| 94 | No action needed | 1 | 1 | 1 | 1 | 1 |
| 95 | No action needed | 1 | 1 | 1 | 1 | 1 |
| 96 | No action needed | 1 | 1 | 1 | 1 | 1 |
| 97 | No action needed | 1 | 1 | 1 | 1 | 1 |
| 98 | No action needed | 1 | 1 | 1 | 1 | 1 |
| 99 | No action needed | 1 | 1 | 1 | 1 | 1 |
| 100 | No action needed | 1 | 1 | 1 | 1 | 1 |
| 101 | No action needed | 1 | 1 | 1 | 1 | 1 |
| 102 | No action needed | 1 | 1 | 1 | 1 | 1 |
| 103 | No action needed | 1 | 1 | 1 | 1 | 1 |
| 104 | No action needed | 1 | 1 | 1 | 1 | 1 |
| 105 | Change medication | 3 | 3 | 3 | 3 | 3 |
| 106 | Change medication | 3 | 3 | 3 | 3 | 3 |
| 107 | No action needed | 1 | 1 | 1 | 1 | 1 |
| 108 | No action needed | 1 | 1 | 1 | 1 | 1 |
| 109 | No action needed | 1 | 1 | 1 | 1 | 1 |
| 110 | No action needed | 1 | 1 | 1 | 1 | 1 |
| 111 | No action needed | 1 | 1 | 1 | 1 | 1 |
| 112 | No action needed | 1 | 1 | 1 | 1 | 1 |
| 113 | No action needed | 1 | 1 | 1 | 1 | 1 |
| 114 | No action needed | 1 | 1 | 1 | 1 | 1 |
| 115 | No action needed | 1 | 1 | 1 | 1 | 1 |
| 116 | No action needed | 1 | 1 | 1 | 1 | 1 |
| 117 | No action needed | 1 | 1 | 1 | 1 | 1 |
| 118 | No action needed | 1 | 1 | 1 | 1 | 1 |
| 119 | No action needed | 1 | 1 | 1 | 1 | 1 |
| 120 | No action needed | 1 | 1 | 1 | 1 | 1 |
| 121 | No action needed | 1 | 1 | 1 | 1 | 1 |
| 122 | No action needed | 1 | 1 | 1 | 1 | 1 |
| 123 | No action needed | 1 | 1 | 1 | 1 | 1 |
| 124 | No action needed | 1 | 1 | 1 | 1 | 1 |
| 125 | No action needed | 1 | 1 | 1 | 1 | 1 |
| 126 | Change medication | 3 | 3 | 3 | 2 | 3 |
| 127 | Change medication | 3 | 3 | 3 | 2 | 3 |
| 128 | No action needed | 1 | 1 | 1 | 1 | 1 |
| 129 | No action needed | 1 | 1 | 1 | 1 | 1 |
| 130 | No action needed | 1 | 1 | 1 | 1 | 1 |
| 131 | No action needed | 1 | 1 | 1 | 1 | 1 |
| 132 | No action needed | 1 | 1 | 1 | 1 | 1 |
| 133 | No action needed | 1 | 1 | 1 | 1 | 1 |
| 134 | No action needed | 1 | 1 | 1 | 1 | 1 |
| 135 | No action needed | 1 | 1 | 1 | 1 | 1 |
| 136 | No action needed | 1 | 1 | 1 | 1 | 1 |
| 137 | No action needed | 1 | 1 | 1 | 1 | 1 |
| 138 | No action needed | 1 | 1 | 1 | 1 | 1 |
| 139 | No action needed | 1 | 1 | 1 | 1 | 1 |
| 140 | No action needed | 1 | 1 | 1 | 1 | 1 |
| 141 | No action needed | 1 | 1 | 1 | 1 | 1 |
| 142 | No action needed | 1 | 1 | 1 | 1 | 1 |
| 143 | No action needed | 1 | 1 | 1 | 1 | 1 |
| 144 | No action needed | 1 | 1 | 1 | 1 | 1 |
| 145 | No action needed | 1 | 1 | 1 | 1 | 1 |
| 146 | No action needed | 1 | 1 | 1 | 1 | 1 |
| 147 | No action needed | 1 | 1 | 1 | 1 | 1 |
| 148 | No action needed | 1 | 1 | 1 | 1 | 1 |
| 149 | No action needed | 1 | 1 | 1 | 1 | 1 |
| 150 | No action needed | 1 | 1 | 1 | 1 | 1 |
| 151 | No action needed | 1 | 1 | 1 | 1 | 1 |
| 152 | No action needed | 1 | 1 | 1 | 1 | 1 |
| 153 | No action needed | 1 | 1 | 1 | 1 | 1 |
| 154 | No action needed | 1 | 1 | 1 | 1 | 1 |
| 155 | No action needed | 1 | 1 | 1 | 1 | 1 |
| 156 | No action needed | 1 | 1 | 1 | 1 | 1 |
| 157 | Consider dosage modification | 2 | 2 | 2 | 2 | 2 |
| 158 | Consider dosage modification | 2 | 2 | 2 | 2 | 2 |
| 159 | Consider dosage modification | 2 | 2 | 2 | 2 | 2 |
| 160 | Consider dosage modification | 2 | 2 | 2 | 2 | 2 |
| 161 | No action needed | 1 | 1 | 1 | 1 | 1 |
| 162 | No action needed | 1 | 1 | 1 | 1 | 1 |
| 163 | No action needed | 1 | 1 | 1 | 1 | 1 |
| 164 | No action needed | 1 | 1 | 1 | 1 | 1 |
| 165 | No action needed | 1 | 1 | 1 | 1 | 1 |
| 166 | No action needed | 1 | 1 | 1 | 1 | 1 |
| 167 | No action needed | 1 | 1 | 1 | 1 | 1 |
| 168 | No action needed | 1 | 1 | 1 | 1 | 1 |
| 169 | No action needed | 1 | 1 | 1 | 1 | 1 |
| 170 | No action needed | 1 | 1 | 1 | 1 | 1 |
| 171 | No action needed | 1 | 1 | 1 | 1 | 1 |
| 172 | Change medication | 3 | 3 | 3 | 2 | 3 |
| 173 | No action needed | 1 | 1 | 1 | 1 | 1 |
| 174 | No action needed | 1 | 1 | 1 | 1 | 1 |
| 175 | No action needed | 1 | 1 | 1 | 1 | 1 |
| 176 | No action needed | 1 | 1 | 1 | 1 | 1 |
| 177 | No action needed | 1 | 1 | 1 | 1 | 1 |
| 178 | No action needed | 1 | 1 | 1 | 1 | 1 |
| 179 | Consider dosage modification | 2 | 2 | 3 | 2 | 2 |
| 180 | Consider dosage modification | 2 | 3 | 3 | 2 | 2 |
| 181 | Change medication | 3 | 3 | 3 | 2 | 3 |
| 182 | Change medication | 3 | 3 | 3 | 3 | 3 |
| 183 | Change medication | 3 | 3 | 3 | 3 | 3 |
| 184 | Change medication | 3 | 3 | 3 | 3 | 3 |
| 185 | Change medication | 3 | 3 | 3 | 3 | 3 |
| 186 | Change medication | 3 | 3 | 3 | 3 | 3 |
| 187 | Change medication | 3 | 3 | 3 | 3 | 3 |
| 188 | Change medication | 3 | 3 | 3 | 3 | 3 |
| 189 | Change medication | 3 | 3 | 3 | 3 | 3 |
| 190 | Change medication | 3 | 3 | 3 | 3 | 3 |
| 191 | Change medication | 3 | 3 | 3 | 3 | 3 |
| 192 | Change medication | 3 | 3 | 3 | 3 | 3 |
| 193 | Change medication | 3 | 3 | 3 | 3 | 3 |
| 194 | Change medication | 3 | 3 | 3 | 3 | 3 |
| 195 | Change medication | 3 | 3 | 3 | 3 | 3 |
| 196 | Change medication | 3 | 3 | 3 | 3 | 3 |
| 197 | No action needed | 1 | 1 | 1 | 1 | 1 |
| 198 | Consider dosage modification | 2 | 2 | 3 | 2 | 2 |
| 199 | Consider dosage modification | 2 | 2 | 3 | 2 | 2 |
| 200 | No action needed | 1 | 1 | 1 | 1 | 1 |
| 201 | Change medication | 3 | 3 | 3 | 2 | 3 |
| 202 | Consider dosage modification | 2 | 2 | 3 | 2 | 2 |
| 203 | Change medication | 3 | 5 | 3 | 3 | 3 |
| 204 | Consider dosage modification | 2 | 2 | 2 | 2 | 2 |
| 205 | Change medication | 3 | 3 | 3 | 3 | 3 |
| 206 | Change medication | 3 | 3 | 3 | 3 | 3 |
| 207 | Change medication | 3 | 3 | 3 | 3 | 3 |
| 208 | Change medication | 3 | 3 | 3 | 3 | 3 |
| 209 | Consider dosage modification | 2 | 2 | 2 | 2 | 2 |
| 210 | Consider dosage modification | 3 | 3 | 3 | 2 | 2 |
| 211 | Consider dosage modification | 3 | 3 | 3 | 2 | 2 |
| 212 | Monitor adverse effects | 1 | 1 | 1 | 1 | 1 |
| 213 | No action needed | 1 | 1 | 1 | 1 | 1 |
| 214 | No action needed | 1 | 1 | 1 | 1 | 1 |
| 215 | No action needed | 1 | 1 | 1 | 1 | 1 |
| 216 | No action needed | 1 | 1 | 1 | 1 | 1 |
| 217 | No action needed | 1 | 1 | 1 | 1 | 1 |
| 218 | No action needed | 1 | 1 | 1 | 1 | 1 |
| 219 | No action needed | 1 | 1 | 1 | 1 | 1 |
| 220 | No action needed | 1 | 1 | 1 | 1 | 1 |
| 221 | No action needed | 1 | 1 | 1 | 1 | 1 |
| 222 | Consider dosage modification | 2 | 2 | 2 | 2 | 2 |
| 223 | Consider dosage modification | 2 | 2 | 2 | 2 | 2 |
| 224 | Consider dosage modification | 2 | 2 | 2 | 2 | 2 |
| 225 | Consider dosage modification | 2 | 2 | 3 | 2 | 2 |
| 226 | Consider dosage modification | 2 | 2 | 3 | 2 | 2 |
| 227 | Change medication | 3 | 2 | 3 | 2 | 3 |
| 228 | Change medication | 3 | 3 | 3 | 3 | 3 |
| 229 | Change medication | 3 | 3 | 3 | 3 | 3 |
| 230 | Consider dosage modification | 3 | 3 | 3 | 3 | 3 |
| 231 | Other | 5 | 5 | 5 | 5 | 5 |
| 232 | Change medication | 3 | 3 | 3 | 3 | 3 |
| 233 | Change medication | 3 | 3 | 3 | 3 | 3 |
| 234 | Change medication | 3 | 3 | 3 | 3 | 3 |
| 235 | Change medication | 3 | 5 | 3 | 3 | 3 |
| 236 | Consider dosage modification | 2 | 5 | 3 | 2 | 2 |
| 237 | Other | 5 | 5 | 5 | 2 | 5 |
| 238 | Change medication | 3 | 3 | 3 | 3 | 3 |
| 239 | Consider dosage modification | 2 | 2 | 2 | 2 | 2 |
| 240 | No action needed | 1 | 1 | 1 | 1 | 1 |
| 241 | Consider dosage modification | 2 | 2 | 2 | 2 | 2 |
| 242 | Monitor adverse effects | 1 | 1 | 1 | 1 | 1 |
| 243 | Consider dosage modification | 2 | 2 | 2 | 2 | 2 |
| 244 | Consider dosage modification | 2 | 3 | 2 | 2 | 2 |
| 245 | Change medication | 3 | 3 | 3 | 3 | 3 |
| 246 | Consider dosage modification | 2 | 2 | 2 | 2 | 2 |
| 247 | Consider dosage modification | 2 | 2 | 2 | 2 | 2 |
| 248 | Consider dosage modification | 2 | 2 | 2 | 2 | 2 |
| 249 | Change medication | 3 | 3 | 3 | 3 | 3 |
| 250 | Change medication | 3 | 3 | 3 | 3 | 3 |
| 251 | Change medication | 3 | 3 | 3 | 3 | 3 |
| 252 | Consider dosage modification | 3 | 3 | 3 | 3 | 3 |
| 253 | Consider dosage modification | 3 | 3 | 3 | 3 | 3 |
| 254 | Consider dosage modification | 2 | 3 | 3 | 2 | 2 |
| 255 | Consider dosage modification | 2 | 2 | 2 | 2 | 2 |
| 256 | Consider dosage modification | 2 | 2 | 2 | 2 | 2 |
| 257 | Change medication | 3 | 3 | 3 | 3 | 3 |
| 258 | Change medication | 3 | 3 | 3 | 3 | 3 |
| 259 | Change medication | 3 | 3 | 3 | 3 | 3 |
| 260 | Change medication | 3 | 3 | 3 | 3 | 3 |
| 261 | Change medication | 3 | 3 | 3 | 3 | 3 |
| 262 | Consider dosage modification | 2 | 2 | 2 | 2 | 2 |
| 263 | Consider dosage modification | 2 | 2 | 2 | 2 | 2 |
| 264 | Consider dosage modification | 2 | 2 | 2 | 2 | 2 |
| 265 | Change medication | 3 | 3 | 3 | 3 | 3 |
| 266 | Consider dosage modification | 4 | 4 | 4 | 3 | 4 |
| 267 | Change medication | 3 | 3 | 3 | 3 | 3 |
| 268 | Monitor adverse effects | 1 | 1 | 1 | 1 | 1 |
| 269 | Consider dosage modification | 2 | 2 | 2 | 2 | 2 |
| 270 | Change medication | 3 | 3 | 3 | 3 | 3 |
| 271 | Change medication | 3 | 3 | 3 | 3 | 3 |
| 272 | Change medication | 3 | 3 | 3 | 3 | 3 |
| 273 | Consider dosage modification | 2 | 2 | 2 | 2 | 2 |
| 274 | Change medication | 3 | 3 | 3 | 3 | 3 |
| 275 | Consider dosage modification | 2 | 5 | 2 | 2 | 2 |
| 276 | Consider dosage modification | 2 | 5 | 2 | 2 | 2 |
| 277 | Consider dosage modification | 5 | 5 | 4 | 2 | 5 |
| 278 | Consider dosage modification | 5 | 5 | 4 | 2 | 5 |
| 279 | Consider dosage modification | 5 | 5 | 4 | 2 | 5 |
| 280 | Consider dosage modification | 5 | 5 | 4 | 2 | 5 |
| 281 | Consider dosage modification | 5 | 5 | 4 | 2 | 5 |
| 282 | Consider dosage modification | 5 | 5 | 4 | 2 | 5 |
| 283 | Consider dosage modification | 2 | 2 | 2 | 2 | 2 |
| 284 | Consider dosage modification | 2 | 2 | 2 | 2 | 2 |
| 285 | Change medication | 3 | 3 | 3 | 3 | 3 |
| 286 | Monitor adverse effects | 1 | 1 | 1 | 1 | 1 |
| 287 | Change medication | 3 | 3 | 3 | 3 | 3 |
| 288 | No action needed | 1 | 1 | 1 | 1 | 1 |
| 289 | No action needed | 1 | 1 | 1 | 1 | 1 |
| 290 | Consider dosage modification | 2 | 3 | 3 | 2 | 2 |
| 291 | Consider dosage modification | 2 | 3 | 3 | 2 | 2 |
| 292 | Consider dosage modification | 2 | 2 | 2 | 2 | 2 |
| 293 | No action needed | 1 | 1 | 1 | 1 | 1 |
| 294 | Change medication | 3 | 3 | 3 | 3 | 3 |
| 295 | No action needed | 1 | 1 | 1 | 1 | 1 |
| 296 | Change medication | 3 | 3 | 3 | 3 | 3 |
| 297 | Change medication | 3 | 3 | 3 | 3 | 3 |
| 298 | Change medication | 3 | 3 | 3 | 3 | 3 |
| 299 | Consider dosage modification | 2 | 2 | 2 | 2 | 2 |
| 300 | Consider dosage modification | 3 | 3 | 2 | 3 | 3 |
| 301 | Change medication | 3 | 3 | 3 | 3 | 3 |
| 302 | No action needed | 1 | 1 | 1 | 1 | 1 |
| 303 | No action needed | 1 | 1 | 1 | 1 | 1 |
| 304 | Change medication | 3 | 3 | 3 | 3 | 3 |
| 305 | No action needed | 1 | 1 | 1 | 1 | 1 |
| 306 | Monitor adverse effects | 1 | 1 | 1 | 1 | 1 |
| 307 | Consider dosage modification | 2 | 2 | 2 | 2 | 2 |
| 308 | No action needed | 1 | 1 | 1 | 1 | 1 |
| 309 | Consider dosage modification | 2 | 2 | 2 | 2 | 2 |
| 310 | Consider dosage modification | 1 | 1 | 1 | 1 | 1 |
| 311 | No action needed | 1 | 1 | 1 | 1 | 1 |
| 312 | Change medication | 3 | 3 | 3 | 3 | 3 |
| 313 | Consider dosage modification | 2 | 2 | 2 | 2 | 2 |
| 314 | Consider dosage modification | 3 | 3 | 3 | 3 | 3 |
| 315 | No action needed | 1 | 1 | 1 | 1 | 1 |
| 316 | Change medication | 3 | 3 | 3 | 3 | 3 |
| 317 | Consider dosage modification | 2 | 2 | 2 | 2 | 2 |
| 318 | Change medication | 3 | 3 | 3 | 3 | 3 |
| 319 | Change medication | 3 | 3 | 3 | 3 | 3 |
| 320 | No action needed | 1 | 1 | 1 | 1 | 1 |
| 321 | No action needed | 1 | 1 | 1 | 1 | 1 |
| 322 | No action needed | 1 | 1 | 1 | 1 | 1 |
| 323 | No action needed | 1 | 1 | 1 | 1 | 1 |
| 324 | No action needed | 1 | 1 | 1 | 1 | 1 |
| 325 | No action needed | 1 | 1 | 1 | 1 | 1 |
| 326 | No action needed | 1 | 1 | 1 | 1 | 1 |
| 327 | No action needed | 1 | 1 | 1 | 1 | 1 |
| 328 | No action needed | 1 | 1 | 1 | 1 | 1 |
| 329 | Consider dosage modification | 1 | 1 | 1 | 1 | 1 |
| 330 | Consider dosage modification | 2 | 2 | 3 | 2 | 2 |
| 331 | Consider dosage modification | 2 | 2 | 2 | 2 | 2 |
| 332 | Consider dosage modification | 2 | 2 | 2 | 2 | 2 |
| 333 | Consider dosage modification | 2 | 2 | 2 | 2 | 2 |
| 334 | Change medication | 3 | 3 | 3 | 3 | 3 |
| 335 | Consider dosage modification | 2 | 2 | 2 | 2 | 2 |
| 336 | Consider dosage modification | 2 | 3 | 3 | 2 | 2 |
| 337 | Consider dosage modification | 2 | 3 | 3 | 2 | 2 |
| 338 | No action needed | 1 | 1 | 1 | 1 | 1 |
| 339 | No action needed | 1 | 1 | 1 | 1 | 1 |
| 340 | No action needed | 1 | 1 | 1 | 1 | 1 |
| 341 | No action needed | 1 | 1 | 1 | 1 | 1 |
| 342 | Consider dosage modification | 2 | 3 | 3 | 2 | 2 |
| 343 | No action needed | 1 | 1 | 1 | 1 | 1 |
| 344 | No action needed | 1 | 1 | 1 | 1 | 1 |
| 345 | Consider dosage modification | 2 | 2 | 3 | 2 | 2 |
| 346 | No action needed | 1 | 1 | 1 | 1 | 1 |
| 347 | No action needed | 1 | 1 | 1 | 1 | 1 |
| 348 | Consider dosage modification | 2 | 2 | 2 | 2 | 2 |
| 349 | Change medication | 3 | 3 | 3 | 3 | 3 |
| 350 | Consider dosage modification | 1 | 1 | 1 | 1 | 1 |
| 351 | No action needed | 1 | 1 | 1 | 1 | 1 |
| 352 | Consider dosage modification | 2 | 2 | 2 | 2 | 2 |
| 353 | Change medication | 3 | 3 | 3 | 3 | 3 |
| 354 | Consider dosage modification | 1 | 1 | 1 | 1 | 1 |
| 355 | Consider dosage modification | 2 | 2 | 2 | 2 | 2 |
| 356 | Consider dosage modification | 2 | 2 | 2 | 2 | 2 |
| 357 | Change medication | 3 | 3 | 3 | 3 | 3 |
| 358 | Consider dosage modification | 2 | 2 | 2 | 2 | 2 |
| 359 | No action needed | 1 | 1 | 1 | 1 | 1 |
| 360 | Consider dosage modification | 2 | 3 | 2 | 2 | 2 |
| 361 | No action needed | 1 | 1 | 1 | 1 | 1 |
| 362 | Change medication | 3 | 3 | 3 | 3 | 3 |
| 363 | Monitor adverse effects | 1 | 1 | 1 | 1 | 1 |
| 364 | No action needed | 1 | 1 | 1 | 1 | 1 |
| 365 | Consider dosage modification | 1 | 1 | 1 | 1 | 1 |
| 366 | Consider dosage modification | 2 | 3 | 2 | 2 | 2 |
| 367 | Consider dosage modification | 2 | 2 | 2 | 2 | 2 |
| 368 | No action needed | 1 | 1 | 1 | 1 | 1 |
| 369 | Change medication | 3 | 3 | 3 | 3 | 3 |
| 370 | Consider dosage modification | 2 | 3 | 2 | 2 | 2 |
| 371 | Consider dosage modification | 2 | 2 | 2 | 2 | 2 |
| 372 | Monitor adverse effects | 1 | 1 | 1 | 1 | 1 |
| 373 | Change medication | 3 | 3 | 3 | 3 | 3 |
| 374 | Change medication | 3 | 3 | 3 | 3 | 3 |
| 375 | Consider dosage modification | 2 | 3 | 3 | 2 | 2 |
| 376 | Consider dosage modification | 2 | 3 | 3 | 2 | 2 |
| 377 | No action needed | 1 | 1 | 1 | 1 | 1 |
| 378 | Monitor adverse effects | 1 | 1 | 1 | 1 | 1 |
| 379 | Change medication | 3 | 3 | 3 | 3 | 3 |
| 380 | Change medication | 3 | 3 | 3 | 3 | 3 |
| 381 | Consider dosage modification | 2 | 2 | 2 | 2 | 2 |
| 382 | Consider dosage modification | 2 | 2 | 2 | 2 | 2 |
| 383 | Consider dosage modification | 2 | 2 | 2 | 2 | 2 |
| 384 | Consider dosage modification | 2 | 2 | 2 | 2 | 2 |
| 385 | Change medication | 3 | 3 | 3 | 3 | 3 |

1. Final Label refers to the consensus decision reached for each pharmacogenomic guideline after reviewing the classifications made by five pharmacists from different healthcare systems (H.Y.H, L.H.L, C.L.W, C.Y.L, and C.K.L).
2. The classifications include: 1) No action needed, 2) Consider dosage modification, 3) Change medication, 4) Monitor adverse effects, and 5) Other.

**Table S5. Class-Wise Evaluation Metrics for GPT-4o and Gemini-1.5-Pro on Pharmacogenomic Guideline Classification**

- 1. **Evaluation Metrics for GPT-4o Performance**

| **Class** | **Precision** | **Recall** | **F1 Score** | **Predicted Count** | **Actual Count** |
| --- | --- | --- | --- | --- | --- |
| 1. No action needed | 0.9764 | 1.0000 | 0.9880 | 212 | 207 |
| 2. Consider dosage modification | 0.8372 | 1.0000 | 0.9115 | 86 | 72 |
| 3. Change medication | 1.0000 | 0.8660 | 0.9282 | 84 | 97 |
| 4. Monitor adverse effects | 0.3333 | 1.0000 | 0.5000 | 3 | 1 |
| 5. Other | 0.0000 | 0.0000 | 0.0000 | 0 | 8 |
| Macro Average | 0.6294 | 0.7732 | 0.6655 | - | - |
| Weighted Average | 0.9351 | 0.9351 | 0.9288 | 385 | 385 |

- 1. **Evaluation Metrics for Gemini-1.5-Pro Performance**

| **Class** | **Precision** | **Recall** | **F1 Score** | **Predicted Count** | **Actual Count** |
| --- | --- | --- | --- | --- | --- |
| 1. No action needed | 1.0000 | 0.9372 | 0.9676 | 194 | 207 |
| 2. Consider dosage modification | 0.7826 | 1.0000 | 0.8780 | 92 | 72 |
| 3. Change medication | 1.0000 | 0.9175 | 0.9570 | 89 | 97 |
| 4. Monitor adverse effects | 0.0000 | 0.0000 | 0.0000 | 0 | 1 |
| 5. Other | 0.8000 | 1.0000 | 0.8889 | 10 | 8 |
| Macro Average | 0.7165 | 0.7709 | 0.7383 | - | - |
| Weighted Average | 0.9455 | 0.9351 | 0.9350 | 385 | 385 |

**Table S6. Error analysis of 44 pharmacogenomic guideline items with classification discrepancies between GPT-4o and Gemini-1.5-Pro**

| **No.** | **Drug** | **Gene** | **GPT Prediction** | **Gemini Prediction** | **Final label assigned by human reviewers** | **Analysis of possible error causes** |
| --- | --- | --- | --- | --- | --- | --- |
| 277 | daunorubicin | RARG | monitor adverse effects | consider dosage modification | Consider dosage modification | For narrow therapeutic index drugs, dose adjustment should be carefully considered |
| 278 | daunorubicin | SLC28A3 | monitor adverse effects | consider dosage modification | Consider dosage modification |  |
| 279 | daunorubicin | UGT1A6 | monitor adverse effects | consider dosage modification | Consider dosage modification |  |
| 280 | doxorubicin | RARG | monitor adverse effects | consider dosage modification | Consider dosage modification |  |
| 281 | doxorubicin | SLC28A3 | monitor adverse effects | consider dosage modification | Consider dosage modification |  |
| 282 | doxorubicin | UGT1A6 | monitor adverse effects | consider dosage modification | Consider dosage modification |  |
| 310 | disopyramide | CYP2D6 | no action needed | consider dosage modification | Consider dosage modification |  |
| 329 | glyburide | CYP2C9 | no action needed | consider dosage modification | Consider dosage modification |  |
| 354 | phenprocoumon | CYP2C9 | no action needed | consider dosage modification | Consider dosage modification |  |
| 212 | diclofenac | CYP2C8 | no action needed | monitor adverse effects | Monitor adverse effects | Although not advised, adverse events should be monitored for safety. |
| 242 | methadone | CYP2B6 | no action needed | monitor adverse effects | Monitor adverse effects |  |
| 266 | cisplatin | TPMT | monitor adverse effects | consider dosage modification | Consider dosage modification |  |
| 268 | acenocoumarol | CYP2C9 | no action needed | monitor adverse effects | Monitor adverse effects |  |
| 286 | amiodarone | CYP2D6 | no action needed | monitor adverse effects | Monitor adverse effects |  |
| 306 | clozapine | CYP2D6 | no action needed | monitor adverse effects | Monitor adverse effects |  |
| 363 | quinidine | CYP2D6 | no action needed | monitor adverse effects | Monitor adverse effects |  |
| 372 | sotalol | CYP2D6 | no action needed | monitor adverse effects | Monitor adverse effects |  |
| 378 | tolbutamide | CYP2C9 | no action needed | monitor adverse effects | Monitor adverse effects |  |
| 26 | Amitriptyline | CYP2C19 | Consider dosage modification | Change medication | Change medication | Therapy varies with metabolic type |
| 27 | Amitriptyline | CYP2C19 | Consider dosage modification | Change medication | Change medication |  |
| 126 | citalopram | CYP2C19 | consider dosage modification | change medication | Change medication |  |
| 127 | escitalopram | CYP2C19 | consider dosage modification | change medication | Change medication |  |
| 172 | codeine | CYP2D6 | consider dosage modification | change medication | Change medication |  |
| 227 | nortriptyline | CYP2D6 | consider dosage modification | change medication | Change medication |  |
| 228 | fluvastatin | CYP2C9 | consider dosage modification | change medication | Change medication |  |
| 229 | fluvastatin | SLCO1B1 | consider dosage modification | change medication | Change medication |  |
| 245 | sertraline | CYP2C19 | consider dosage modification | change medication | Change medication |  |
| 251 | tramadol | CYP2D6 | consider dosage modification | change medication | Change medication |  |
| 252 | simvastatin | SLCO1B1 | change medication | consider dosage modification | Consider dosage modification |  |
| 253 | trimipramine | CYP2C19 | change medication | consider dosage modification | Consider dosage modification |  |
| 300 | clomipramine | CYP2D6 | change medication | consider dosage modification | Consider dosage modification |  |
| 309 | doxepin | CYP2D6 | change medication | consider dosage modification | Consider dosage modification |  |
| 314 | escitalopram | CYP2C19 | change medication | consider dosage modification | Consider dosage modification |  |
| 366 | risperidone | CYP2D6 | change medication | consider dosage modification | Consider dosage modification |  |
| 373 | tamoxifen | CYP2D6 | consider dosage modification | change medication | Change medication |  |
| 379 | tramadol | CYP2D6 | consider dosage modification | change medication | Change medication |  |
| 380 | venlafaxine | CYP2D6 | consider dosage modification | change medication | Change medication |  |
| 231 | ivacaftor | CFTR | no action needed | other | Other | Lack of sufficient clinical evidence |
| 235 | peginterferon alfa 2a | IFNL3 | consider dosage modification | change medication | Change medication |  |
| 237 | ribavirin | IFNL3 | consider dosage modification | other | Other |  |
| 350 | oxycodone | CYP2D6 | no action needed | consider dosage modification | Consider dosage modification |  |
| 365 | ribavirin | HLA | no action needed | consider dosage modification | Consider dosage modification |  |
| 211 | imipramine | CYP2D6 | change medication | consider dosage modification | Consider dosage modification | Avoid use; if necessary, adjust the dosage accordingly. |
| 230 | lovastatin | SLCO1B1 | change medication | consider dosage modification | Consider dosage modification |  |
